# Effect of multiple comorbidities on mortality in chronic obstructive pulmonary disease among Korean population: a nationwide cohort study

**DOI:** 10.1186/s12890-021-01424-7

**Published:** 2021-02-11

**Authors:** Youngmee Kim, Ye-Jee Kim, Won-Kyung Cho

**Affiliations:** 1 Red Cross College of Nursing, Chung-Ang University, Seoul, Korea; 2 Department of Clinical Epidemiology and Biostatistics, Asan Medical Center, University of Ulsan College of Medicine, Seoul, Korea; 3 International Healthcare Center, Department of Pulmonary and Critical Care Medicine, Asan Medical Center, University of Ulsan College of Medicine, 88, Olympic-ro 43-gil, Songpa-gu, Seoul, 05505 Korea

**Keywords:** COPD, Mortality, Comorbidity

## Abstract

**Background:**

The effects of comorbidities on chronic obstructive pulmonary disease (COPD) have been usually studied individually in the past. In this study, we aimed to investigate the comorbidities associated with mortality, the effect of multimorbidity on mortality and other factors associated with mortality among Korean COPD population.

**Methods:**

The Korean National Health Insurance Service-National Sample Cohort version 2.0, collected between 2002 and 2015, was used. Among COPD patients [entire cohort (EC), N = 12,779], 44% of the participants underwent additional health examination, and they were analysed separately [health-screening cohort (HSC), N = 5624]. Fifteen comorbidities previously reported as risk factors for mortality were studied using Cox proportional hazards regression models.

**Results:**

Total mortality rates were 38.6 per 1000 person-years (95% CI 37.32–40.01) and 27.4 per 1000 person-years (95% CI 25.68–29.22) in EC and HSC, respectively. The most common causes of death were disease progression, lung cancer, and pneumonia. Only some of the comorbidities had a direct impact on mortality. Multimorbidity, assessed by the number of comorbid diseases, was an independent risk factor of all-cause mortality in both cohorts and was a risk factor of respiratory mortality only in HSC. The Kaplan–Meier analysis showed significant differences in survival trajectories according to the number of comorbidities in all-cause mortality but not in respiratory mortality. Low BMI, old age and male sex were independent risk factors for both mortalities in both cohorts.

**Conclusions:**

The number of comorbidities might be an independent risk factor of COPD mortality. Multimorbidity contributes to all-cause mortality in COPD, but the effect of multimorbidity is less evident on respiratory mortality.

## Background

Chronic obstructive pulmonary disease (COPD) is characterised by chronic irreversible airflow limitation and is usually caused by cigarette smoking. COPD is a major cause of disability and death around the world [1]. Medical comorbidities are prevalent among COPD patients, and previous research has shown that comorbidities affect not only symptom burden and functional performance in patients with COPD but also the risk of exacerbation, hospitalisation and mortality [2, 3]. The majority of COPD patients have more than one comorbidity; however, in most of the studies that have addressed the effects of a comorbidity on COPD clinical outcomes, comorbidities have been studied individually [1, 3].

In the past, a few studies have tried to examine the effect of multiple comorbidities on COPD outcomes more systematically by using or developing the measurement instruments. For example, the Charlson comorbidity index (CCI) is one of the most widely used measurement tools by healthcare professionals to assess the burden of multiple comorbid diseases, and the CCI has been well-validated to predict the mortality in COPD patients [4]. However, CCI is not a disease-specific instrument, and few COPD-specific indices to assess the cumulative burdens of multiple comorbidities on COPD outcomes have been developed. For instance, the COPD-specific comorbidity test (COTE) index and the COMorbidities in COPD (COMCOLD) index were recently developed and validated to predict the mortality and general quality of life in COPD patients, respectively [5, 6]. In addition, Putcha et al. reported a simple scoring system, wherein the number of comorbidities could identify the susceptible COPD patient at the risk of developing exacerbation [7]. Intriguingly, a difference in comorbidity profile by race and ethnicity in COPD patients has been reported [8]. Therefore, it will be of interest to see whether differences in comorbidity profiles according to race and ethnicity lead to differences in disease outcomes.

With this background, this study was conducted to examine the following: first, the comorbidities associated with mortality; second, the number of comorbidities could be an independent risk factor to predict mortality and third, other factors associated with mortality among Korean COPD population using a nationwide population-based cohort.

## Methods

### Study subjects

The Korean National Health Insurance Service (KNHIS) has been implemented in 1963, covering approximately 97% of the population in South Korea. KNHIS has two components: mandatory social health insurance and medical aid. The medical aid program is a form of public assistance that uses government subsidies to provide low-income groups with healthcare services [9]. The KNHIS developed the National Health Insurance Service-National Sample Cohort (NHIS-NCS) for research purposes, containing all medical information related to insurance claims. The database comprises eligibility and demographic information regarding health insurance as well as data on medical aid beneficiaries, medical bill details, medical treatment, disease histories and prescriptions; such data were constructed after converting insurance claim information to the first day of medical treatment. The NHIS-NCS is a semi-dynamically constructed cohort database; the cohort has been followed-up to either the time of the participant’s disqualification from receiving health services due to death or emigration or until the end of the study period. The detailed structure and function of KNHIS is described elsewhere [10].

This study used the National Health Insurance Service-National Sample Cohort, version 2.0 (NHIS-NCS v2.0), sampled between 2002 and 2015 [9]. This database provides neither pulmonary function data nor participants’ physical symptoms that are necessary to diagnose COPD. Therefore, COPD patients were recruited according to their International Classification of Disease-Tenth Revision (ICD-10) and prescription history. Specifically, among 1,108,369 participants in this cohort, COPD patients with the following inclusion criteria were chosen first as previously used in one study [11]: ≥ 40 years of age; ICD-10 codes for COPD (J43-J44, except J430) and COPD medication use confirmed at least twice per year. COPD medications include long-acting muscarinic antagonist (LAMA), long-acting beta-2 agonist (LABA), inhaled corticosteroid (ICS), methylxanthines and systemic beta-agonists in this study. Among these, only the newly diagnosed COPD patients were selected by allowing a one-year pre-study period to avoid any potential diagnostic conflicts. Then the participants were followed up during the entire study period until the last year of qualification for those who were alive, or until the date of death for those who died. The cases of death within one year after diagnosis of COPD were excluded due to the possibility of the death being from uncertain cause. To ensure that each study participant had ≥ 1 year follow-up period, COPD patients diagnosed in the last year of the study period were excluded (entire cohort).

**Fig. 1:**
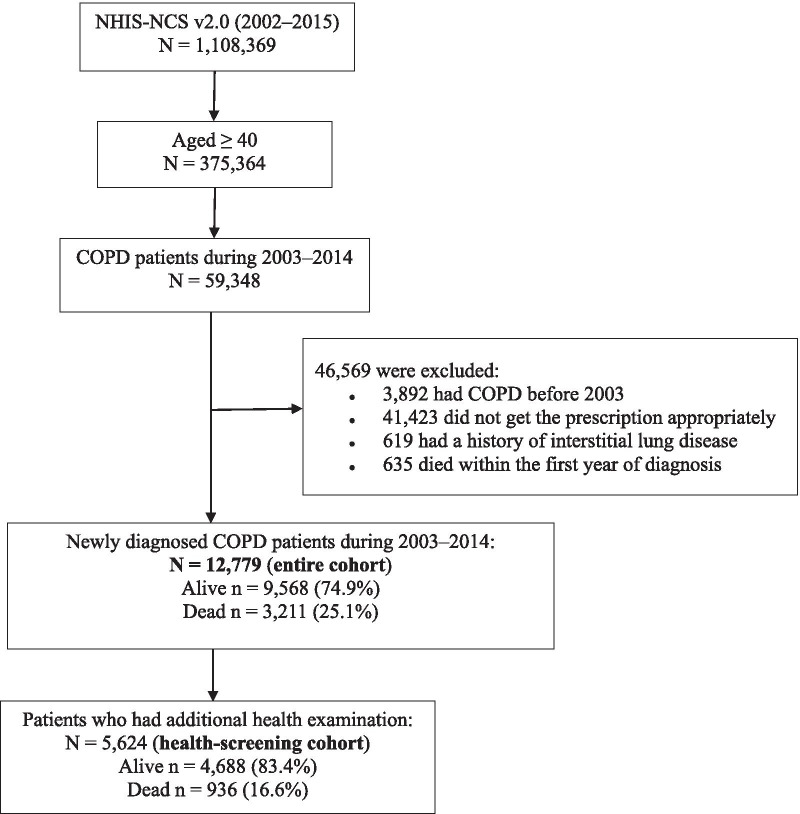
Flow diagram showing the study participants

The more detailed data, including laboratory data, were available for some patients who participated in national health screening, which was 44% of the entire cohort. We also analysed the data from these separately to identify additional factors associated with mortality (health screening cohort). Figure 1 demonstrates the selection process and the number of study participants.

### Definition of comorbidities and other parameters

The selection of target chronic comorbid diseases and other parameters was based on existing literature and data availability [1, 3, 5, 12–14]. The presence of comorbidity was screened during the one-year pre-study period using ICD-10 codes. Finally, fifteen comorbidities that were previously reported to be risk factors for mortality were included in this study. Notably, we counted different types of malignancies under one comorbidity, even though individual malignancies were studied independently in this study. In addition, malignancies that needed either diagnostic work-ups or active treatment were only included to exclude the remote history of malignancies.

Concerning other clinical parameters, body mass index (BMI; kg/m^2^) was classified as follows: low (< 18.5), normal (18.5–22.9), overweight (23–24.9) and obese (≥ 25) [15]. The reference ranges of blood tests, as determined by the cohort user manual [9], were as follows: the normal range of haemoglobin levels (g/dL) for men was 13.0–16.4 and that for women was 12.0–15.5; the normal range of fasting blood glucose (FBG) levels (mg/dL) was 100–125; the normal range of total cholesterol (TC) (mg/dL) levels was < 200 and the normal range of serum creatinine (mg/dL) levels was < 1.5 [9]. In this study, we assessed both all-cause mortality and respiratory mortality. Respiratory mortality was defined as any death from a respiratory cause except for the malignancy of bronchus and lung.

### Statistical analysis

All data are presented as means ± standard deviation (SD) for continuous variables or as frequency and proportions for categorical variables. We calculated the sex- and cause-specific mortality rates per 1000 person-years and 95% confidence intervals (CI), assuming a Poisson distribution of the data. Cox proportional hazards regression models were used to explore the associations between mortality and each variable. All variables for which the *p* value was < 0.1 in univariate analysis were included in multiple Cox regression analysis using the backward elimination method. The results are presented as estimated hazard ratios (HRs) with 95% confidence intervals. A 95% CI that did not span 1.0 was considered statistically significant.

In addition, the impact of the comorbidity count on the death of COPD patients was evaluated using the Kaplan–Meier method. We applied a multiple imputation procedure using a Markov Chain Monte Carlo (MCMC) method to impute missing values in the health-screening cohort. The multiple imputed data sets were analysed using the same analytical procedures, and the results from these analyses were combined to obtain an overall estimate. Data were analysed using SAS Enterprise Guide software version 7.1 (SAS Institute, Inc., Cary, NC, USA). A *p* value < 0.05 was considered statistically significant.

## Results

### Baseline characteristics of the entire and the health-screening cohorts

Table 1 shows the baseline characteristics of the entire and health-screening cohorts. In the entire group, the median follow-up period was 6.5 years. 3211 participants, 25.1% of the entire group, died during the follow-up period. Among the participants, 54.5% were men, and the mean age at diagnosis of COPD was 66.4 years. The mean number of comorbidities of the participants was 2.56, but 11.4% of the participants had no comorbidity. Among comorbidities, asthma, hypertension and dyslipidaemia were the most common diseases in the entire group.

**Table 1:** Baseline characteristics of the study participants

	Entire cohort	Health screening cohort
No. of participants	12,779	5624
Follow-up period (years)	6.5 ± 3.37	6.09 ± 3.26
No. of death	3211 (25.1)	936 (16.6)
**General characteristics**		
Mean age at recruitment (years)	66.40 ± 11.22	65.03 ± 10.46
Male	6966 (54.5)	3250 (57.8)
Health insurance type		
Medical aids	11,134 (87.1)	5540 (98.5)
Health insurance	1643 (12.9)	84 (1.5)
Household income		
1st quintile	1767 (13.8)	878 (15.6)
2nd quintile	1528 (12.0)	767 (13.6)
3rd quintile	1875 (14.7)	930 (16.5)
4th quintile	2536 (19.9)	1273 (22.6)
5th quintile	3317 (26.0)	1627 (28.9)
Missing	1754 (13.7)	149 (2.7)
**Comorbidities**		
No. of comorbid disease	2.56 ± 1.86	2.57 ± 1.83
(Range)	(0–11)	(0–9)
0	1454 (11.4)	615 (11.0)
1 or 2	5615 (44.0)	2461 (43.8)
3 or 4	3630 (28.4)	1633 (29.0)
≥ 5	2078 (16.3)	915 (16.3)
**Cardiovascular comorbidity**	7007 (54.8)	3041 (54.1)
Hypertension	5910 (46.3)	2541 (45.2)
Ischemic heart disease	1995 (15.6)	837 (14.9)
Cardiac arrhythmia	871 (6.8)	382 (6.8)
Heart failure	913 (7.2)	315 (5.6)
Cerebrovascular disease	1233 (9.7)	438 (7.8)
Peripheral vascular disease	1048 (8.2)	514 (9.1)
**Other respiratory comorbidity**	7083 (55.4)	3081 (54.8)
Asthma	6853 (53.6)	2970 (52.8)
Bronchiectasis	761 (6.0)	328 (5.8)
**Metabolic comorbidity**	5980 (46.8)	2695 (48.0)
Diabetes mellitus	3102 (24.3)	1260 (22.4)
Dyslipidaemia	3516 (27.5)	1694 (30.1)
Chronic kidney disease	141 (1.1)	43 (0.8)
Osteoporosis	1934 (15.1)	844 (15.0)
**Gastrointestinal comorbidity**	3830 (30.0)	2021 (35.9)
Gastro-oesophageal reflux disease	3419 (26.8)	1846 (32.8)
Chronic liver disease	679 (5.3)	321 (5.7)
**Malignancy comorbidity**	375 (2.9)	140 (2.5)
Lung cancer	135 (1.1)	50 (0.9)
Stomach cancer	45 (0.4)	17 (0.3)
Colorectal cancer	48 (0.4)	17 (0.3)
Liver cancer	37 (0.3)	6 (0.1)
Thyroid cancer	14 (0.1)	10 (0.2)
**Health examination data**		
BMI, kg/m^2^		23.79 ± 3.42
Haemoglobin, g/dL		13.67 ± 1.57
Fasting blood glucose, mg/dL		102.37 ± 31.33
Total Cholesterol, mg/dL		197.43 ± 49.35
Serum creatinine, mg/dL		1.00 ± 0.84

Values are n (%) or mean ± standard deviation (SD)

The median follow-up period was 6.09 years in the health-screening cohort. In this cohort, 936 participants died during the follow-up period, amounting to 16.6% death rate. In the health-screening cohort, 57.8% of the participants were men, and the mean age at diagnosis of COPD was 65.03 years. The mean number of comorbidities per participant was 2.57, but 11.0% of the participants had no comorbidity. Asthma, hypertension and GERD were the most common diseases.

### Mortality rates and cause of death

Table 2 shows the mortality rates by the cause of death in both cohorts. Total mortality rates were 38.6 per 1000 person-years (95% CI 37.32–40.01) and 27.3 per 1000 person-years (95% CI 25.59–29.11) in the entire cohort and the health-screening cohort, respectively. Three major common categories of the cause of deaths in both cohorts were respiratory disorders, malignancy, and cardiovascular disorders. The most common causes of death in both cohorts were the disease progression of COPD, lung cancer, pneumonia and acute myocardial infarction. Overall, participants died of non-respiratory causes more than of respiratory causes. More detailed information about the mortality rates according to age and sex in both cohorts is presented in Additional file 1: Table S1.

**Table 2:** Mortality rates by the cause of death

Causes of death	Entire cohort	Health screening cohort
	MR (95% CI)	MR (95% CI)
Total	38.6 (37.32–40.01)	27.3 (25.59–29.11)
*Respiratory system (J00-98)*	8.7 (8.03–9.31)	5.7 (4.97–6.61)
J44	4.1 (3.66–4.54)	2.6 (2.09 –3.20)
J12, J15, J16, J18	1.9 (1.66–2.27)	1.4 (1.03–1.86)
J45	0.9 (0.69–1.10)	0.4 (0.24–0.72)
J84	0.7 (0.50–0.86)	0.7 (0.45–1.04)
Others	1.1 (0.87–1.33)	0.6 (0.38–0.94)
*Malignancy (C00-97)*	10.3 (9.58–10.97)	9.4 (8.40–10.48)
C34	4.4 (3.98–4.89)	4.2 (3.57–4.98)
C22	0.9 (0.74–1.17)	0.6 (0.36–0.90)
C16	0.9 (0.72–1.14)	0.8 (0.57–1.22)
C18	0.5 (0.34–0.66)	0.3 (0.16–0.57)
Others	3.5 (3.11–3.93)	
*Cardiovascular system (I00-99)*	8.1 (7.45–8.69)	5.0 (4.27–5.80)
I21	1.5 (1.25–1.79)	1.1 (0.78–1.52)
I63	1.1 (0.91–1.38)	0.7 (0.47–1.08)
I69	1 (0.76–1.20)	0.4 (0.22–0.69)
I50	0.7 (0.53–0.90)	0.3 (0.14–0.54)
I11	0.5 (0.38–0.71)	0.3 (0.14–0.54)
I61	0.5 (0.34–0.66)	0.4 (0.22–0.69)
Others	2.7 (2.40–3.12)	1.8 (1.34–2.25)
*Other causes*	11.4 (10.66–12.12)	7.1 (6.28–8.10)
*Missing*	0.3 (0.20–0.46)	0.0 (0.00–0.16)

MR, Mortality rate per 1000 person-years; CI, confidence interval. *ICD-10 codes*:: C34: malignant neoplasm of bronchus and lung; C22: liver cell carcinoma; C16: malignant neoplasm of the stomach; C18: malignant neoplasm of the colon; J44: other chronic obstructive pulmonary diseases; J12, J15, J16 and J18: pneumonia; J45: asthma; J84: other interstitial pulmonary diseases; I21: acute myocardial infarction; I63: cerebral infarction; I69: sequelae of cerebrovascular disease; I11: hypertensive heart disease; I61: intracerebral haemorrhage; I50: heart failure

### Factors associated with all-cause and respiratory mortalities

**Fig. 2:**
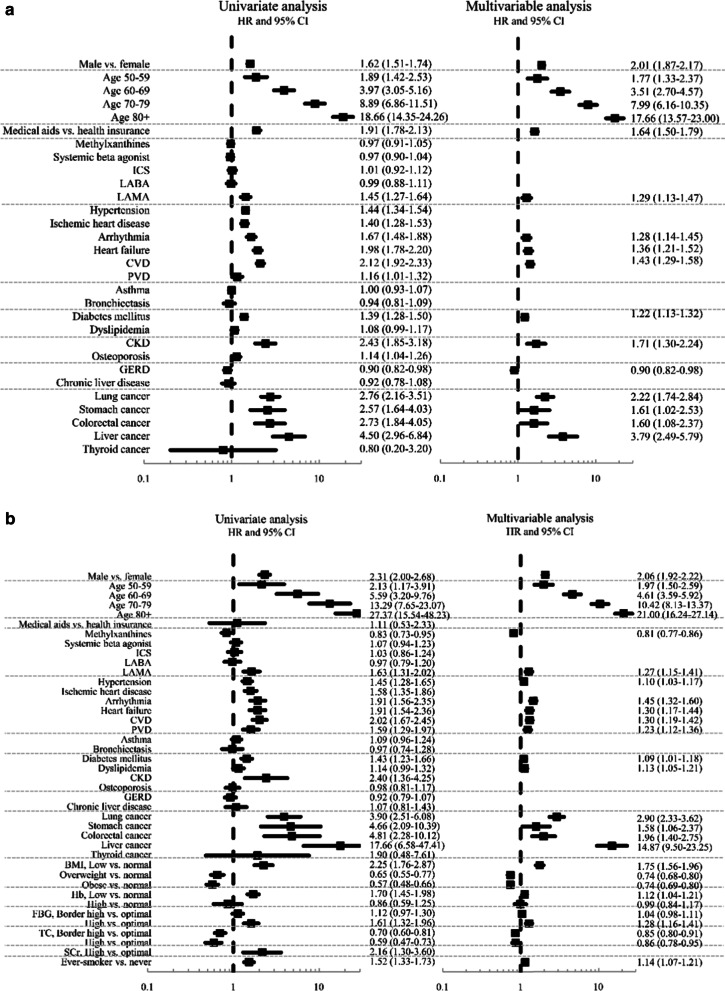
Factors associated with all-cause mortality in the entire cohort (**a**) and the health screening cohort (**b**). *Note:* The final multivariable Cox regression model was constructed by applying backward elimination method after including all variables for which the *p* value was < 0.1 in the univariate analysis. HR, hazard ratios; CI, confidence interval; ICS, inhaled corticosteroid; LABA, long-acting beta agonist; LAMA, long-acting muscarinic agonist; CVD, cerebrovascular disease; PVD, peripheral vascular disease; CKD, chronic kidney disease; GERD, gastro-oesophageal reflux disease; Ever-smoker includes both current (65.2%) and former smoking (34.8%)

**Fig. 3:**
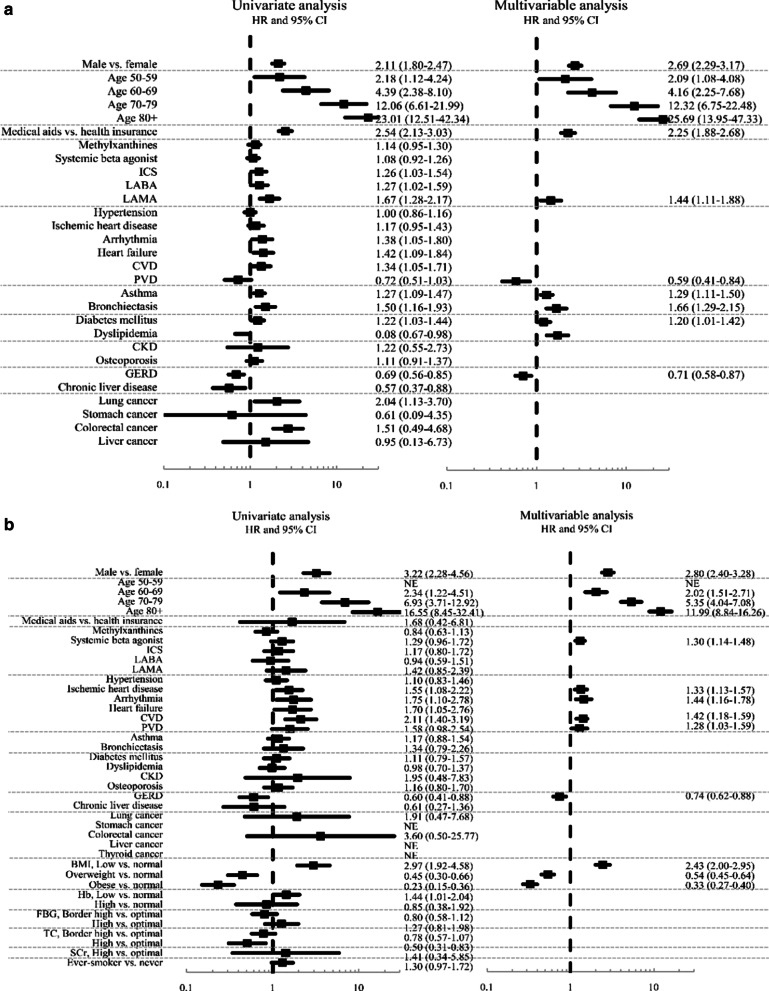
Factors associated with respiratory mortality in the entire cohort (**a**) and health-screening cohort (**b**). *Note:* The final multivariable Cox regression model was constructed applying backward elimination method after including all variables for which the *p* value was < 0.1 in the univariate analysis. ICS, inhaled corticosteroid; LABA, long-acting beta agonist; LAMA, long-acting muscarinic agonist; CVD, cerebrovascular disease; PVD, peripheral vascular disease; CKD, chronic kidney disease; GERD, gastro-oesophageal reflux disease; Ever-smoker includes both current (65.2%) and former smoking (34.8%)

Figures 2 and 3 show univariate and multivariable analyses for the factors associated with mortalities in both cohorts. Male sex, old age, having medical aids suggesting poor economic status and using LAMA at the time of initial enrolment were associated with all-cause mortality in the multivariable analysis of the entire cohort. Among comorbidities, having cardiac arrhythmia, heart failure, cerebrovascular disease, DM, CKD and any malignancy except thyroid cancer were associated with all-cause mortality in the multivariable analysis of the entire cohort (Fig. 2a). In the health-screening cohort, male sex, old age and using LAMA at the time of enrolment were associated with all-cause mortality in the multivariable analysis. Having hypertension, cardiac arrhythmia, heart failure, cerebrovascular disease, peripheral vascular disease, DM, dyslipidaemia, malignancies of lung, stomach and liver, colorectal malignancies, low BMI, high fasting blood glucose and smoking history were associated with all-cause mortality, whereas taking methylxanthine and having high BMI and cholesterol were associated with lower all-cause mortality in the multivariable analysis of the health-screening cohort (Fig. 2b).

Regarding the factors associated with respiratory mortality, male sex, old age, having medical aids and using LAMA were associated with respiratory mortality among the entire cohort. Having asthma, bronchiectasis, DM and dyslipidaemia were also associated with respiratory mortality in the multivariable analysis of the entire cohort (Fig. 3a). In the health-screening cohort, male sex, old age and taking systemic beta-agonist, having ischemic heart disease, cardiac arrhythmia, cerebrovascular disease, peripheral vascular disease and lower BMI were associated with respiratory mortality. In addition, having high BMI was associated with lower respiratory mortality (Fig. 3b). Besides, the Kaplan–Meier survival curves to show the impact of each comorbidity on the death of our study participants are displayed in Additional file 2: Fig. S1 and Additional file 3: Fig. S2.

### Mortality relative to the number of comorbid diseases

**Fig. 4:**
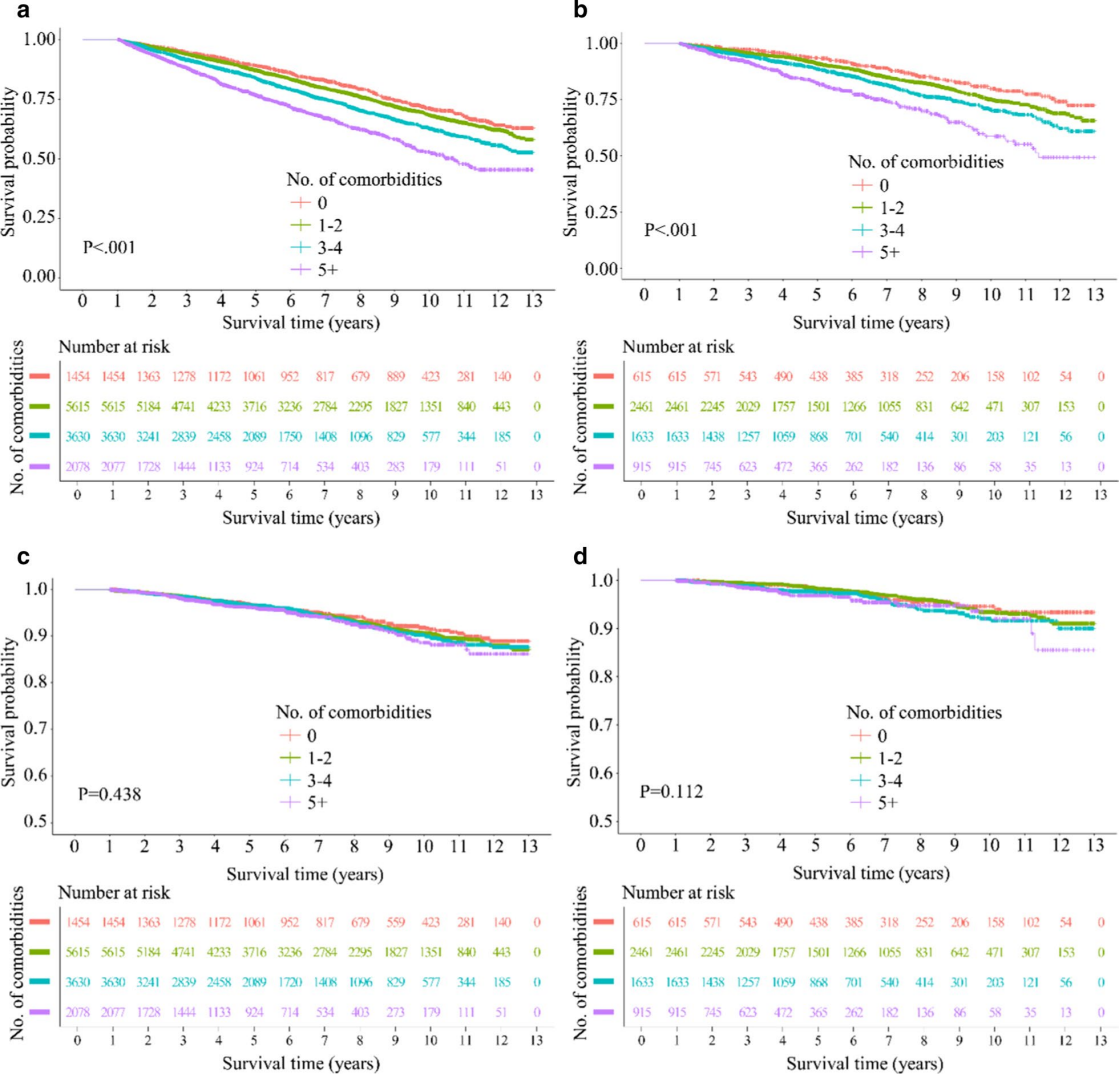
Kaplan–Meier survival curves comparing survival trajectories according to the number of comorbid diseases. **a** Entire cohort (all-cause); **b** Health-screening cohort (all-cause); **c** Entire cohort (respiratory); **d** Health-screening cohort (respiratory)

We examined the effect of multimorbidity on COPD mortality using two different methods. First, the impact of the number of comorbidities on mortality was analysed using the Kaplan–Meier method (Fig. 4). In both cohorts, significant differences in the survival trajectories according to the number of comorbidities were observed only with all-cause mortality and not with respiratory mortality. Second, a multivariable Cox regression analysis was performed to address this further. Again, a significant association between the number of comorbid diseases and all-cause mortality of COPD patients was observed in both cohorts. For instance, patients with more than five comorbidities had hazard ratios of 1.52 (95% CI 1.33–1.75) and 2.14 (95% CI 1.90–2.41) in the entire cohort and health-screening cohort, respectively. In addition, patients with more than five comorbidities had a hazard ratio of 1.87 (95% CI 1.45–2.40) in the health-screening cohort with regard to respiratory mortality. However, the comorbidity number was not associated with respiratory mortality in the entire cohort (Table 3).

**Table 3:** Cox proportional hazard analysis for mortalities focused on the number of comorbidities

No. of comorbid diseases	No. of death	No. of patients	% of death	Univariate analysis		Multivariable analysis	
				HR (95% CI)	*p*	HR (95% CI)	*p*
All-cause in entire cohort*							
0	341	1454	23.45	1.00	< 0.001	1.00	< 0.001
1 or 2	1355	5615	24.13	1.14 (1.01–1.28)		1.02 (0.91–1.15)	
3 or 4	931	3630	25.65	1.42 (1.26–1.61)		1.20 (1.06–1.36)	
5–11	584	2078	28.10	2.00 (1.75–2.29)		1.52 (1.33–1.75)	
All-cause in health-screening cohort**							
0	88	615	14.31	1.00	< 0.001	1.00	0.001
1 or 2	391	2461	15.89	1.29 (1.02–1.62)		1.25 (1.12–1.38)	
3 or 4	278	1633	17.02	1.64 (1.29–2.08)		1.49 (1.33–1.66)	
5–9	179	915	19.56	2.50 (1.93–3.23)		2.14 (1.90–2.41)	
Respiratory cause in entire cohort***							
0	89	1454	6.12	1.00	0.438	1.00	0.944
1 or 2	344	5615	6.13	1.11 (0.88–1.40)		0.97 (0.77–1.23)	
3 or 4	192	3630	5.29	1.14 (0.89–1.47)		0.94 (0.73–1.21)	
5–11	94	2078	4.52	1.27 (0.95–1.70)		0.92 (0.69–1.25)	
Respiratory cause in health-screening cohort****							
0	23	615	(3.74)	1.00	0.112	1.00	< 0.001
1 or 2	84	2461	(3.41)	1.07 (0.67–1.70)		1.10 (0.89–1.35)	
3 or 4	61	1633	(3.74)	1.41 (0.87–2.28)		1.55 (1.25–1.93)	
5–9	29	915	(3.17)	1.63 (0.94–2.83)		1.87 (1.45–2.40)	

^*^Adjusted for sex, age, health insurance type and COPD medication (LAMA); **Adjusted for sex, age, health insurance type, BMI, haemoglobin, total cholesterol, smoking status and COPD medicines (LAMA, Methylxathines); ***Adjusted for sex, age, health insurance type and COPD medicines (LAMA, ICS); ****Adjusted for sex, age, BMI and COPD medicines (systemic beta-agonist)

## Discussion

In this study, we investigated the comorbidities associated with mortality, the effect of multimorbidity on mortality and other factors associated with mortality among physician-diagnosed Korean COPD patients by using two different cohorts derived from a nationwide population-based cohort. Our study has found the following.

Regarding the characteristics of our study participants, most of them had comorbidities, and the average numbers of comorbidities were 2.56 and 2.57 in the entire cohort and health-screening cohort, respectively. Our patients had a relatively fewer comorbidities than compared by other studies [5, 12, 16–18]. However, a direct comparison between studies is challenging due to the expected heterogeneity of comorbidity definitions used in different studies. The most prevalent comorbidities were hypertension, asthma and dyslipidaemia and GERD in both cohorts (Table 1). These findings are similar to what has been reported previously. Notably, most studies have found cardiovascular comorbidities as the most prevalent comorbidities in COPD patients [17, 19, 20].

Total mortality rates were 38.6 per 1000 person-years (95% CI 37.32–40.01) and 27.4 per 1000 person-years (95% CI 25.68–29.22) in the entire cohort and health-screening cohort, respectively. Although the number of comorbid diseases was similar, the difference in all-cause mortality was quite significant between the two cohorts. A potential selection bias for the national health screening cohort can account for this difference. The participants in this cohort seemed to have been more interested in their health in that they wanted more detailed examinations, and this seems to have decreased the mortality rate. The most common causes of death in both cohorts were the disease progression of COPD, lung cancer and acute myocardial infarction. These findings are in agreement with previous reports (Table 2) [3, 5, 13, 14, 21]. Among the respiratory causes of death, deaths from pneumonia could simply represent deaths from COPD exacerbation associated with disease progression. If so, the most common cause of death in our study participants would be the disease progression of COPD. Some studies have reported that the leading causes of death in mild or moderate COPD are lung cancer and cardiovascular diseases, but respiratory failure from disease progression becomes the predominant cause of death in more advanced COPD [3, 14]. From that standpoint, our study participants may have had advanced COPD. However, due to the lack of detailed clinical data with regard to disease severity, it will not be easy to interpret these findings.

We also examined the factors associated with all-cause and respiratory mortalities in both cohorts. The most robust findings are that male sex, old age and low BMI were universal risk factors associated with all-cause and respiratory mortalities in both cohorts. Further, high BMI was also associated with lower all-cause and respiratory mortalities in both cohorts. Low BMI is reportedly a risk factor of all-cause mortality in people with COPD [22, 23]. Although the role of sex as a determinant of clinical outcome of COPD has been controversial [24, 25], male sex turned out to be an obvious risk factor of death in our study (Figs. 2 and 3).

Among comorbidities, arrhythmia, cerebrovascular disease, heart failure, DM and malignancies, including lung cancer, were associated with all-cause mortality in both cohorts. Intriguingly, unlike all-cause mortality, comorbidities associated with respiratory mortality were quite different between the two cohorts. In addition, in both cohorts, fewer comorbid diseases were associated with respiratory mortality than with all-cause mortalities (Figs. 2 and 3).

Next, we investigated whether multimorbidity, assessed by the number of comorbidities, could be a valuable tool to identify the individuals susceptible to death from COPD (Table 3). Consequently, we observed a significant association between the number of comorbid diseases and all-cause mortality of COPD patients in both cohorts. However, the association between the number of comorbidities and respiratory mortality did not seem to be strong in our study participants; this is based on the following. First, there were no significant differences in survival trajectories according to the number of comorbid diseases concerning respiratory mortality in both cohorts (Fig. 4). Second, multivariable Cox regression analysis revealed a significant association between the number of comorbid diseases and respiratory mortality only in the health-screening cohort and not in the entire cohort (Table 3). Therefore, there seems to be a difference with regard to the effect of multimorbidity on mortality between COPD patients who died of disease progression and COPD patients who died of the non-respiratory diseases in our study. We currently do not know how to accurately interpret these findings; however, to speculate, the patients who died of respiratory causes might have had more advanced COPD; consequently, the effect of comorbidities on mortality may mitigate with COPD progression.

Notably, the use of certain medications, particularly LAMA, at the time of diagnosis was associated with increased mortality. Moreover, methylxanthine decreased mortality. We speculate that this is related to the reimbursement criteria of Korean health insurance. LAMA is permitted only in patients with at least moderate airflow limitation. However, there are no strict reimbursement criteria for methylxanthine. Thus, it is likely that LAMA was prescribed in more severe patients and methylxanthine in less severe patients. However, given the lack of pulmonary function data and the detailed history of patient condition, it is difficult to interpret this finding more concretely.

Overall, our study findings can be summarised as follows. First, some comorbid conditions can have a direct impact on COPD mortality; second, comorbid diseases that do not have a direct effect on mortality might still contribute to mortality, probably by intensifying the total burden of comorbidities; third, low BMI, age and male sex are durable risk factors for death and fourth, the number of comorbidities might be an independent risk factor of COPD mortality. And the effect of multimorbidity is more evident on all-cause mortality than on respiratory mortality among Korean COPD population.

We assessed the effect of multimorbidity on COPD mortality by simply counting the number of comorbidities. By doing so, we may have oversimplified the impact of multimorbidity in this study. Previously, a few researchers had developed the measurement instruments to assess the effect of multiple comorbidities on various COPD outcomes [4–7]. Recently, Divo et al. developed the COPD-specific comorbidity test (COTE) index, a disease-specific comorbidity index to predict mortality [5, 6]. In their study, 12 comorbidities associated with increased mortality were first identified out of 79 comorbidities, and the strength of the association of each comorbidity with COPD mortality was assessed by performing multivariate analyses using Cox proportional hazards regression. Scale value points in the range of 1–6 points were assigned to each comorbidity in proportion to its hazard ratio. By summating the points, they were able to assess multimorbidity effect on COPD death. Needless to say, the COTE index is an invaluable tool; however, its utility needs to be further verified. For instance, the value of CCI, which is not a COPD-specific tool, to predict all‐cause mortality in COPD was higher than that of the COTE index in one study [4]. Recently, Putcha et al. assessed the burden of multimorbidity to predict the exacerbation risk of COPD by employing three different ways: (1) simple count, (2) weighted score and (3) weighted score based on statistical selection procedures. They found that the comorbidity count performs best in terms of quantifying the comorbidity burden [7]. The Putcha’s study, as well as ours, suggests that simple counting might be a reliable measure to assess the burden of multimorbidity on clinical outcomes.

There are a few limitations to our study. Differentiation between asthma and COPD in a real-life practice can sometimes be challenging. It is possible that some patients with asthma may have been just COPD patients, which could explain the high prevalence of asthma in our study population. However, we cannot be certain about this due to the lack of detailed clinical information and pulmonary function data.

It has been shown that some mental illnesses, such as depression or anxiety, are associated with an adverse clinical outcome of COPD [26]. However, these data were not available for this study, which can be another weakness of the study. Our analysis focused on the comorbidities that the patient had during the one-year pre-study period. The number and profile of comorbidities may have changed during the follow-up period. Even if it were possible, we believe that the implications of our study findings would not have changed.

Only physician-diagnosed COPD patients were included in our study. In addition, only physician-diagnosed comorbidities were examined, which could be a considerable strength of this study over many other studies that used self-reported comorbidities [5, 21, 27]. However, it is also possible that some comorbidities that are not routinely examined or monitored at our clinical practice, such as osteoporosis, could have been underestimated in this study.

In general, frailty and disability need to be evaluated separately to address the impact of comorbidities on clinical outcomes. As for the frailty factor, we lacked sufficient clinical information about several parameters (e.g., gait speed, grip strength, physical activity, and cognitive function) to assess frailty that are usually difficult to measure in healthcare claims database like ours [28]. Further, given that there were only 1.8% of participants with disabilities in the national sample cohort, the disability factor was not evaluated as it was expected to have minimal impact on the study results.

This study showed that simple comorbidity counting can be another way to assess comorbidity burden, but the study findings should be carefully interpreted as simple counting can oversimplify and misrepresent the impact of multimorbidity. Although our study findings are intriguing, further research is required to implement this knowledge into standard, patient-centred COPD care. In addition, a probable different effect of multimorbidity on mortality between patients who died of non-respiratory causes and who died of respiratory cause may need to be further explored in future studies.

## Conclusions

Our large population-based study showed that some comorbid conditions can have a direct impact on COPD mortality, and comorbid diseases that do not have a direct effect on mortality might still contribute to mortality, probably by intensifying the total burden of comorbidities, given that the number of comorbidities might be an independent risk factor of COPD mortality. In addition, multimorbidity contributes to all-cause mortality in COPD, but the effect of multimorbidity is less evident on respiratory mortality.

## Supplementary Information

**Additional file 1: Table S1.** Mortality rates by age and sex.

**Additional file 2: Fig. S1.** Kaplan-Meier curves comparing all-cause mortalities in entire cohort according to comorbidities and clinical variables.

**Additional file 3: Fig. S2.** Kaplan-Meier curves comparing respiratory mortalities in entire cohort according to comorbidities and clinical variables.

## Data Availability

All data generated or analysed during this study are included in this published article.

## Abbreviations

COPD: Chronic obstructive pulmonary disease
CCI: Charlson comorbidity index
COTE: COPD-specific comorbidity test
COMCOLD: COMorbidities in COPD
KNHIS: Korean National Health Insurance Service
NHIS-NCS: National Health Insurance Service-National Sample Cohort
ICD-10: International Classification of Disease-Tenth Revision
LAMA: Long-acting muscarinic antagonist
LABA: Long-acting beta-2 agonist
ICS: Inhaled corticosteroid
BMI: Body mass index
FBG: Fasting blood glucose
TC: Total cholesterol
SD: Standard deviation
CI: Confidence intervals
HRs: Hazard ratios
MCMC: Markov Chain Monte Carlo
