# Supplementary material for: Effect of multiple comorbidities on mortality in chronic obstructive pulmonary disease among Korean population: a nationwide cohort study
Source: BMC Pulm Med. 2021 Feb 11;21:56. doi: 10.1186/s12890-021-01424-7 (PMC7879613; doi:10.1186/s12890-021-01424-7)
Supplement: Supplementary file 1 — Additional file 1: Table S1. Mortality rates by age and sex. [file 12890_2021_1424_MOESM1_ESM.docx]

Supplementary Table 1. Mortality rates by age and sex

| **Age (years)** | **Male** | **Female** | **Total** |  |  | | |
| --- | --- | --- | --- | --- | --- | --- | --- |
| **All–cause MR: Entire cohort [MR (95% CI)]** | | | |  |  | | |
| 40–49 | 10.5 (7.69–14.02) | 3.3 (1.79–5.49) | 6.9 (5.29–8.92) |  |  |  |  |
| 50–59 | 18.3 (15.55–21.42) | 5.8 (4.11–7.90) | 12.8 (11.04–14.70) |  |  |  |  |
| 60–69 | 37.2 (34.27–40.39) | 13.6 (11.60–15.95) | 27.2 (25.27–29.23) |  |  |  |  |
| 70–79 | 71.7 (67.00–76.69) | 42.3 (38.74–46.10) | 56.8 (53.85–59.89) |  |  |  |  |
| 80+ | 133.3 (120.45–147.12) | 93.6 (85.09–102.72) | 108.8 (101.59–116.46) |  |  |  |  |
| Overall | 46.9 (44.85–48.95) | 29.6 (27.97–31.39) | 38.6 (37.32–40.01) |  |  |  |  |
| **All–cause MR: Health–screening cohort [MR (95% CI)]** | | | |  |  | |  |
| 40–49 | 7.0 (3.62–12.22) | 0.6 (0.02–3.31) | 3.8 (2.04–6.54) |  |  |  |  |
| 50–59 | 11.9 (8.66–15.99) | 3.2 (1.53–5.87) | 7.9 (5.94–10.32) |  |  |  |  |
| 60–69 | 28.8 (25.08–32.98) | 10.0 (7.43–13.12) | 21.1 (18.63–23.81) |  |  |  |  |
| 70–79 | 59.1 (52.82–65.99) | 29.6 (24.73–35.19) | 45.9 (41.79–50.41) |  |  |  |  |
| 80+ | 123.1 (100.69–149.04) | 62.0 (46.43–81.07) | 92.5 (78.65–108.11) |  |  |  |  |
| Overall | 36.4 (33.74–39.22) | 16.1 (14.16–18.27) | 27.4 (25.68–29.22) |  |  |  |  |
| **Respiratory MR: Entire cohort [MR (95% CI)]** | | | |  |  | |  |
| 40–49 | 2.1 (0.94–3.90) | 0.5 (0.06–1.69) | 1.3 (0.63–2.27) |  |  |  |  |
| 50–59 | 3.9 (2.67–5.44) | 1.2 (0.51–2.34) | 2.7 (1.93–3.64) |  |  |  |  |
| 60–69 | 8.1 (6.72–9.60) | 2.1 (1.33–3.08) | 5.5 (4.67–6.47) |  |  |  |  |
| 70–79 | 19.1 (16.67–21.70) | 8.8 (7.21–10.61) | 13.9 (12.41–15.42) |  |  |  |  |
| 80+ | 34.2 (27.83–41.52) | 16.9 (13.37–20.99) | 23.5 (20.21–27.19) |  |  |  |  |
| Overall | 11.4 (10.46–12.49) | 5.6 (4.89–6.39) | 8.7 (8.03–9.31) |  |  |  |  |
| **Respiratory MR: Health–screening cohort [MR (95% CI)]** | | | |  | |  |  |
| 50–59 | 2.4 (1.11–4.63) | 0.6 (0.08–2.31) | 1.6 (0.80–2.88) |  |  |  |  |
| 60–69 | 5.8 (4.23–7.88) | 1.0 (0.32–2.28) | 3.9 (2.84–5.11) |  |  |  |  |
| 70–79 | 13.7 (10.77–17.22) | 5.7 (3.71–8.47) | 10.2 (8.25–12.36) |  |  |  |  |
| 80+ | 36.3 (24.70–51.59) | 9.4 (4.04–18.43) | 22.8 (16.24–31.22) |  |  |  |  |
| Overall | 8.3 (7.02–9.66) | 2.6 (1.89–3.60) | 5.8 (4.99–6.63) |  |  |  |  |
