# Supplementary material for: Effect of multiple comorbidities on mortality in chronic obstructive pulmonary disease among Korean population: a nationwide cohort study
Source: BMC Pulm Med. 2021 Feb 11;21:56. doi: 10.1186/s12890-021-01424-7 (PMC7879613; doi:10.1186/s12890-021-01424-7)
Supplement: Supplementary file 2 — Additional file 2: Fig. S1. Kaplan-Meier curves comparing all-cause mortalities in entire cohort according to comorbidities and clinical variables. [file 12890_2021_1424_MOESM2_ESM.pptx]

## Slide 1
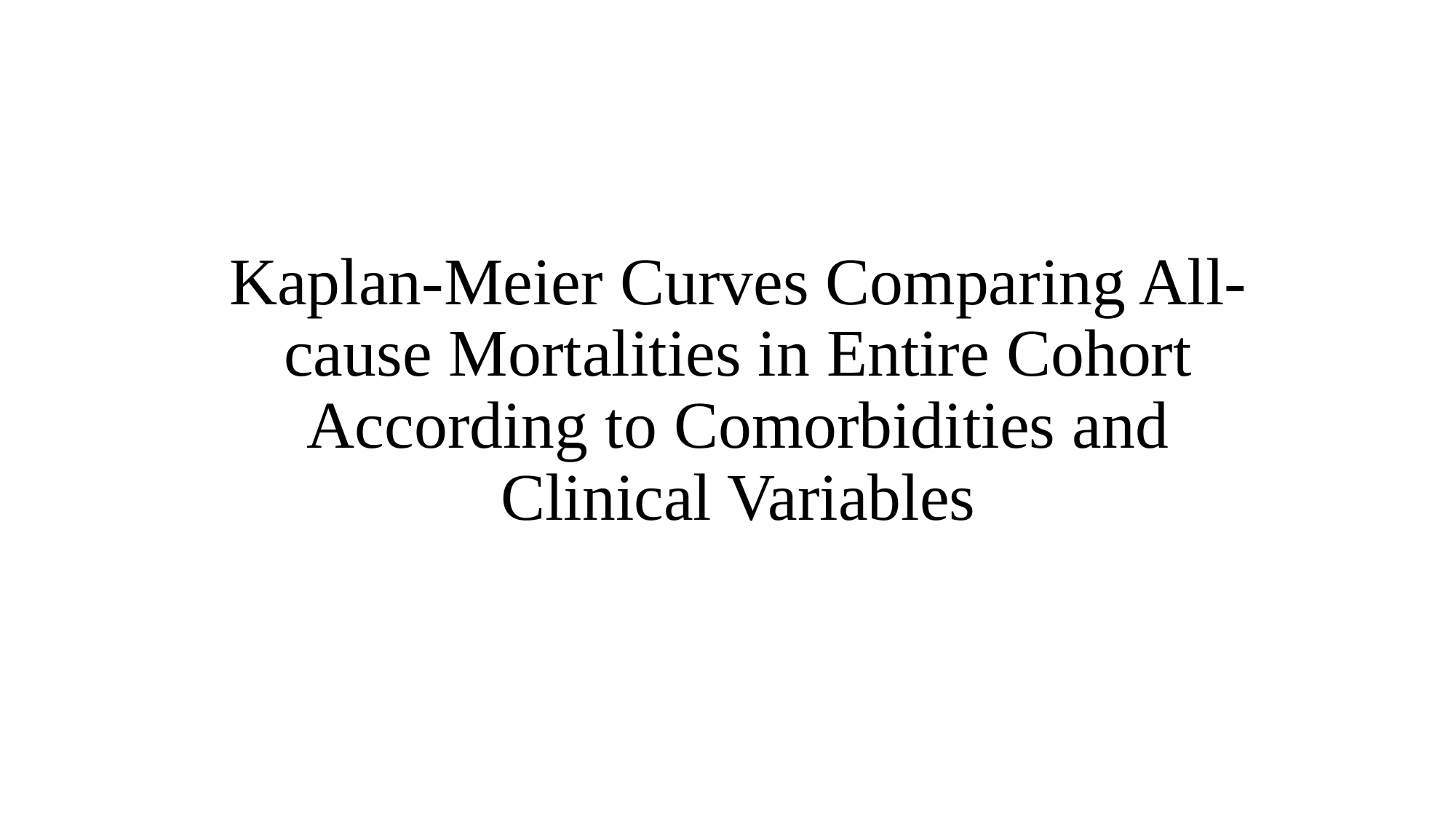

# Kaplan-Meier Curves Comparing All-cause Mortalities in Entire Cohort According to Comorbidities and Clinical Variables

## Slide 2
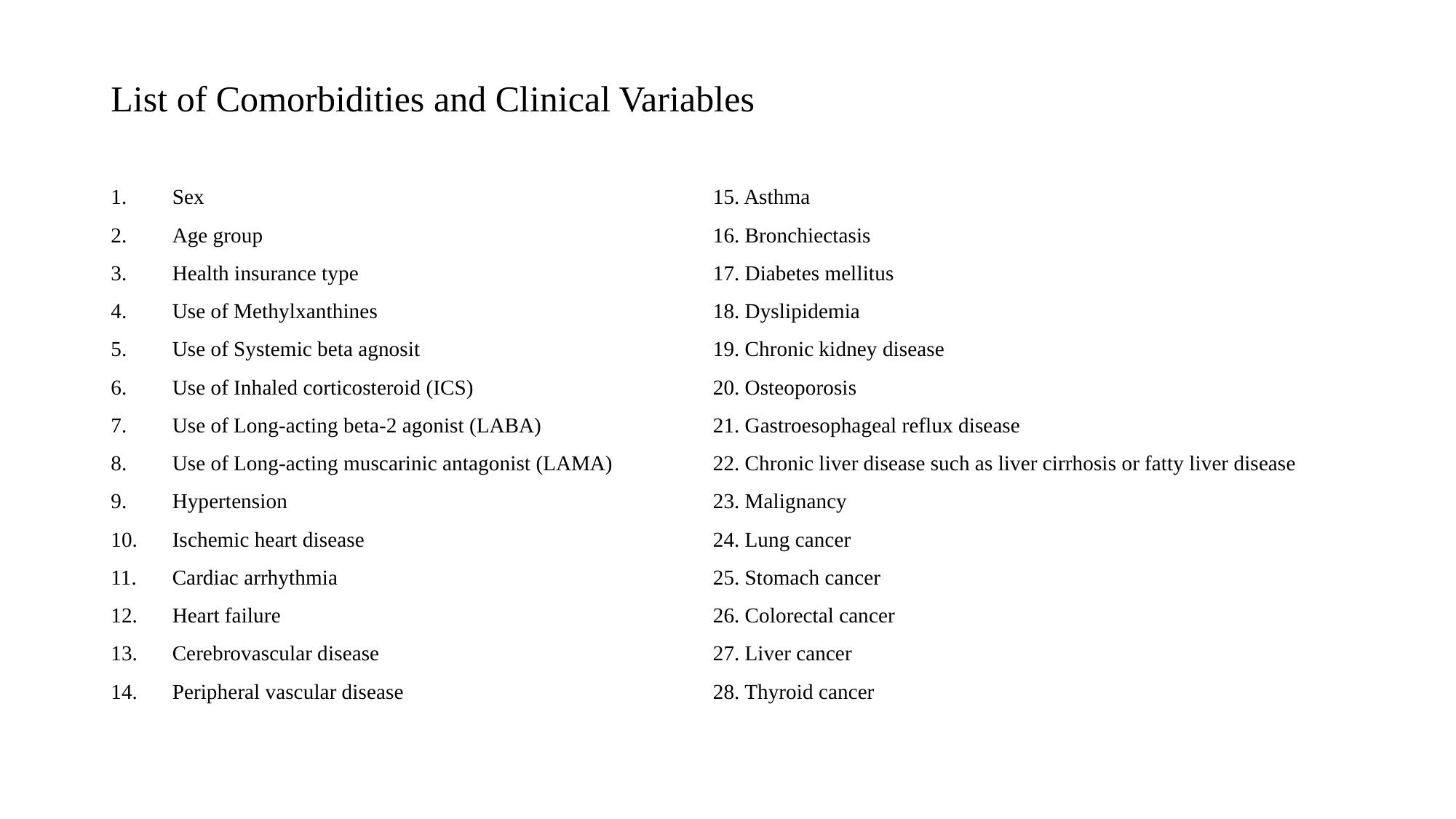

# List of Comorbidities and Clinical Variables
Sex
Age group
Health insurance type
Use of Methylxanthines
Use of Systemic beta agnosit
Use of Inhaled corticosteroid (ICS)
Use of Long-acting beta-2 agonist (LABA)
Use of Long-acting muscarinic antagonist (LAMA)
Hypertension
Ischemic heart disease
Cardiac arrhythmia
Heart failure
Cerebrovascular disease
Peripheral vascular disease
15. Asthma
16. Bronchiectasis
17. Diabetes mellitus
18. Dyslipidemia
19. Chronic kidney disease
20. Osteoporosis
21. Gastroesophageal reflux disease
22. Chronic liver disease such as liver cirrhosis or fatty liver disease
23. Malignancy
24. Lung cancer
25. Stomach cancer
26. Colorectal cancer
27. Liver cancer
28. Thyroid cancer

## Slide 3
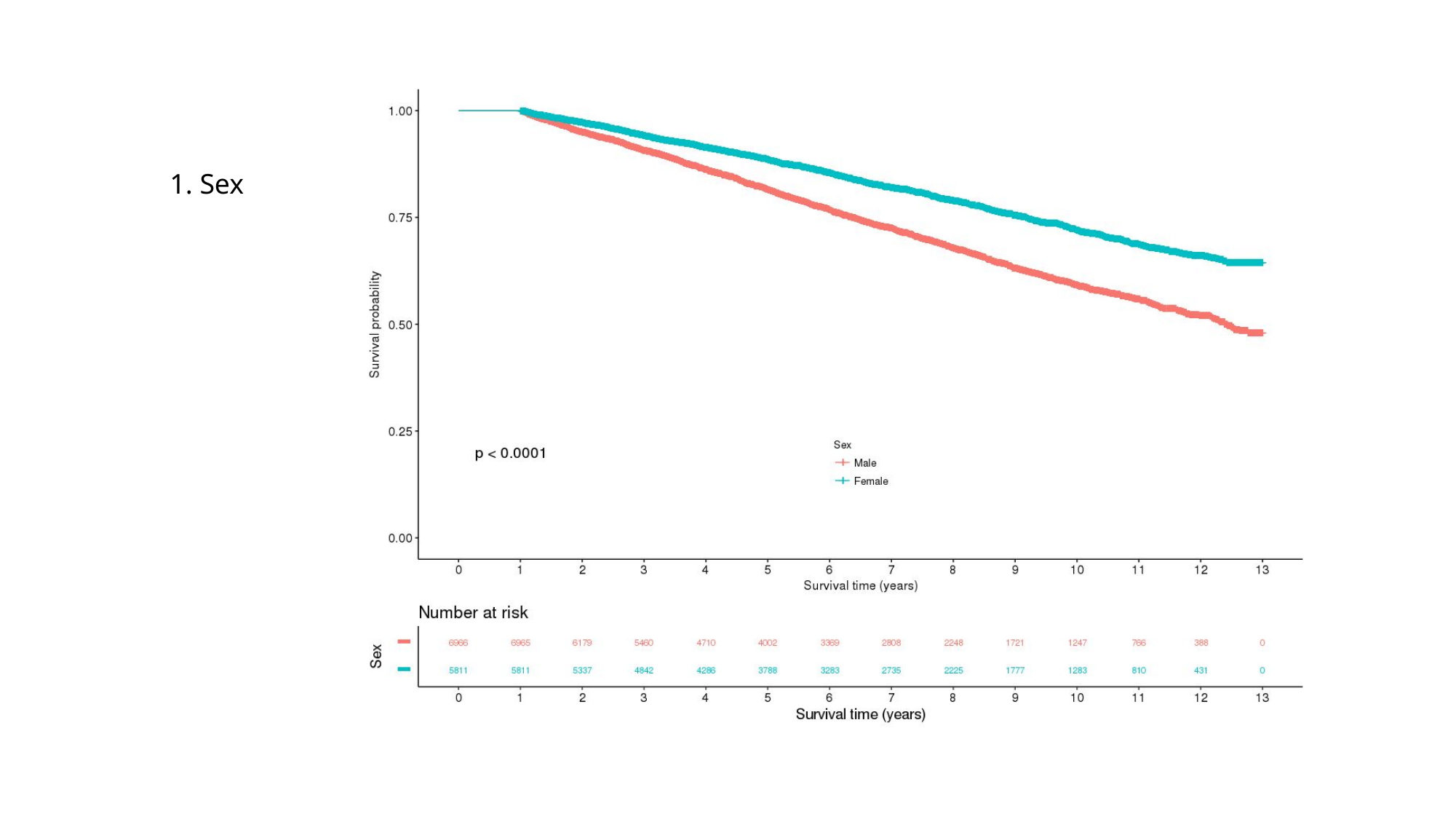

1. Sex

## Slide 4
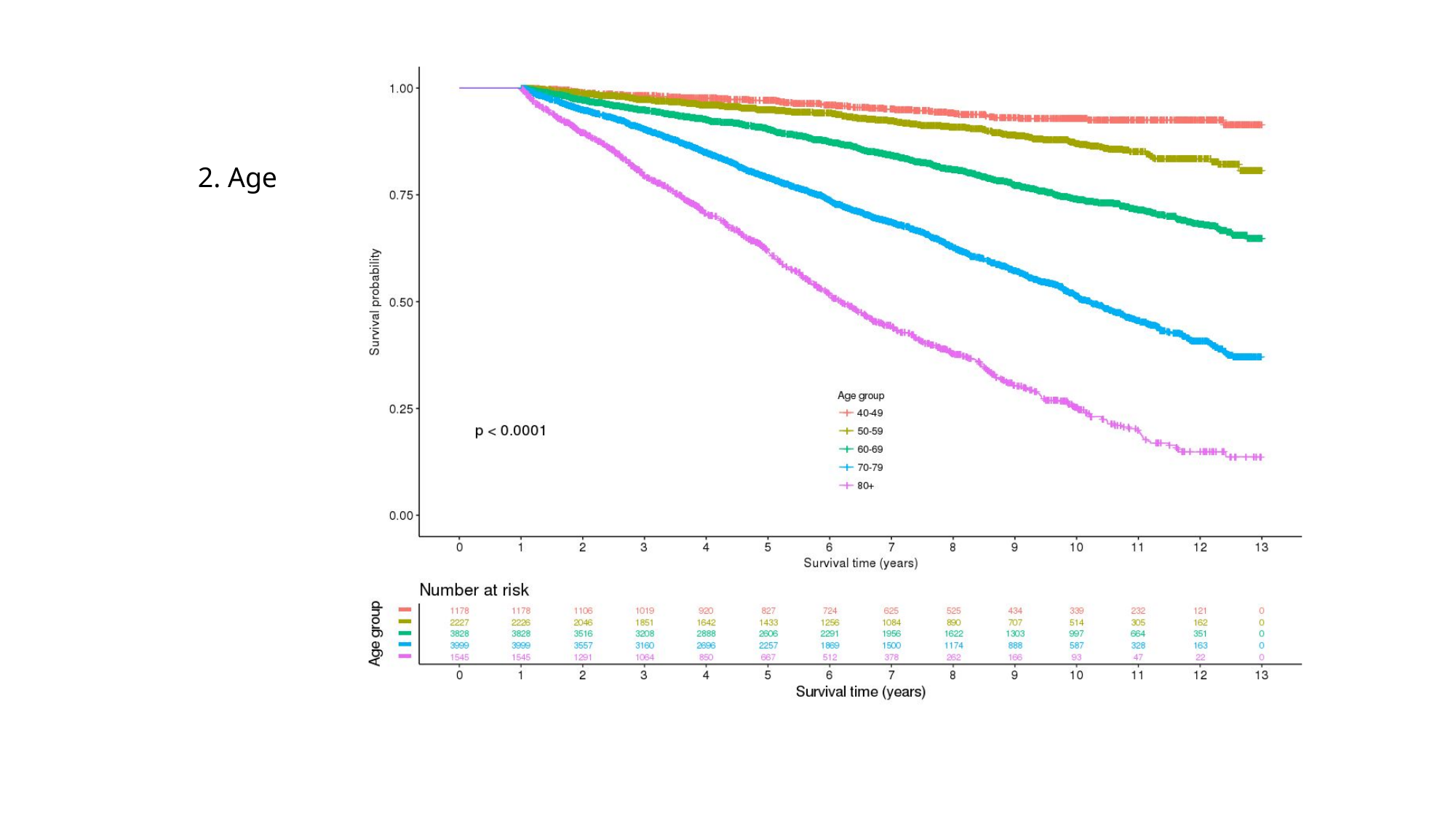

2. Age

## Slide 5
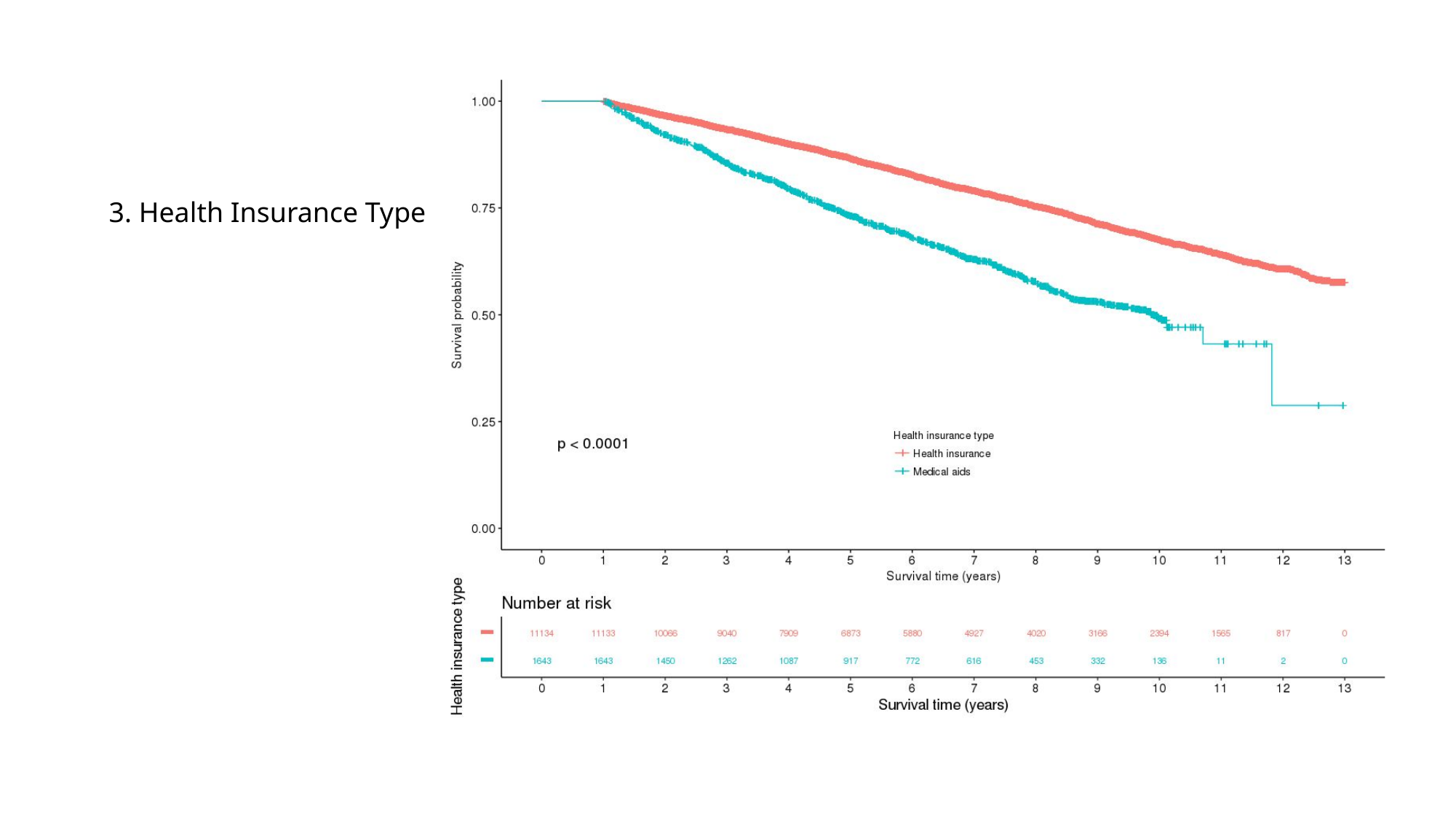

3. Health Insurance Type

## Slide 6
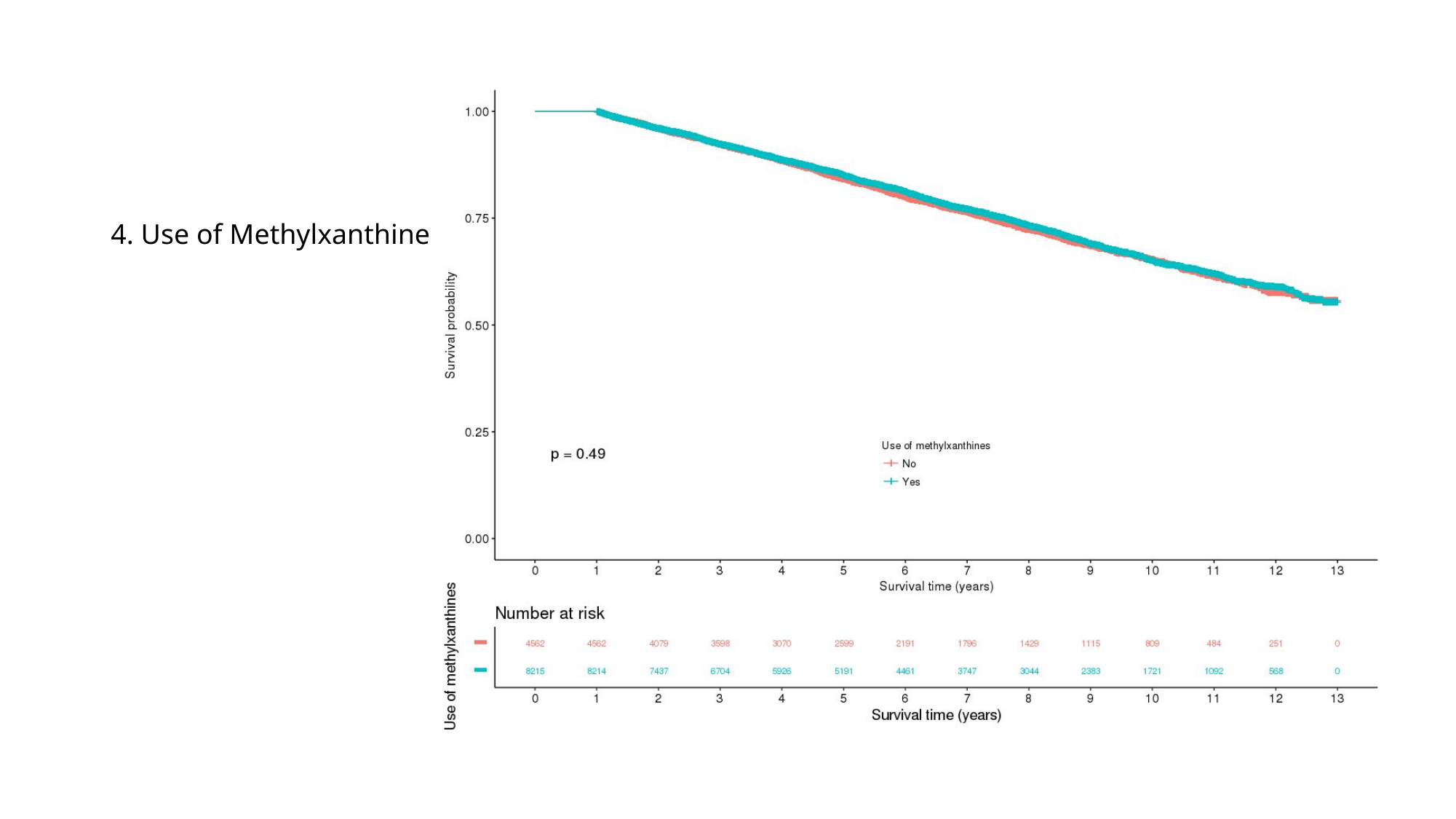

4. Use of Methylxanthine

## Slide 7
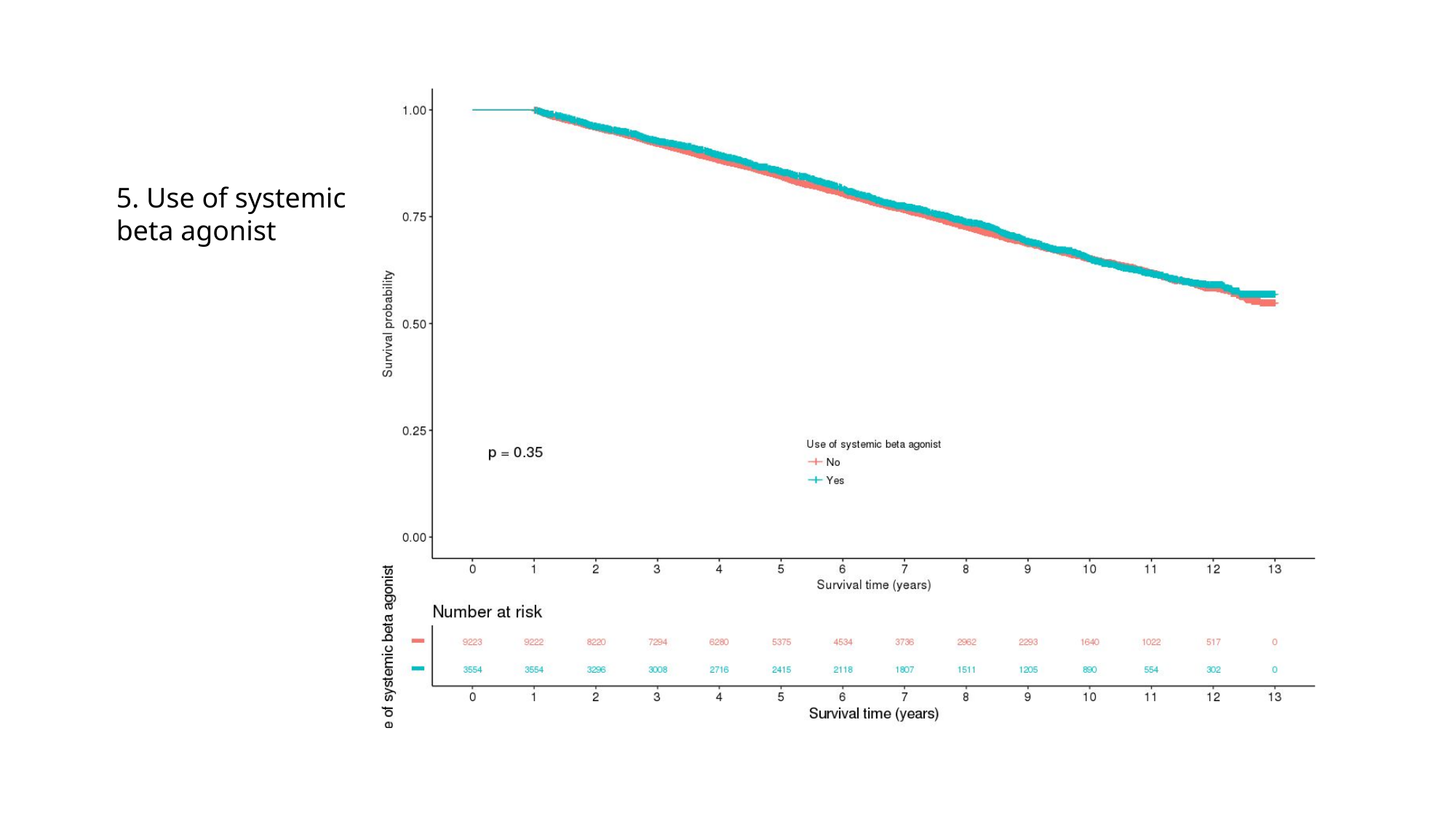

5. Use of systemic
beta agonist

## Slide 8
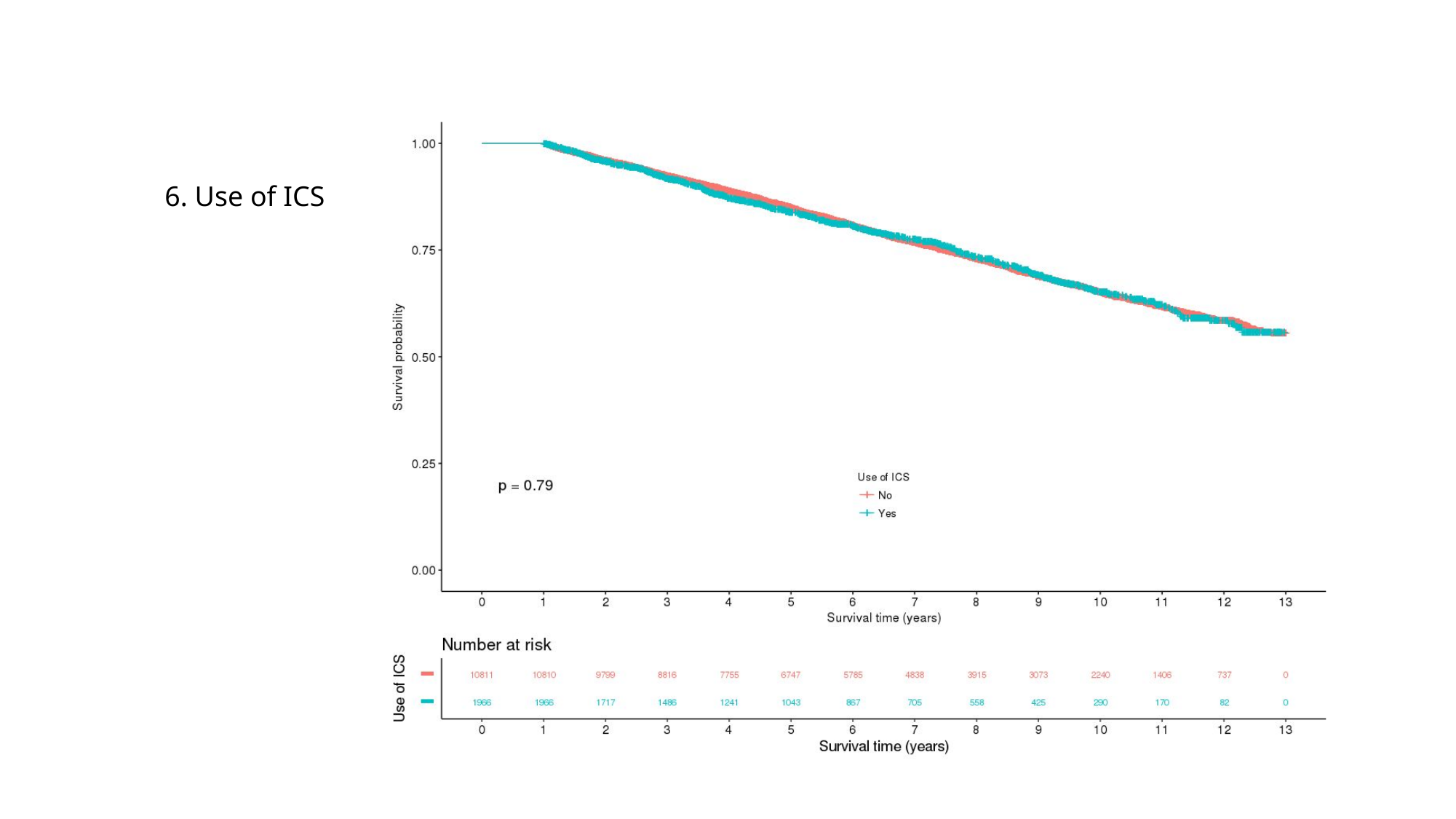

6. Use of ICS

## Slide 9
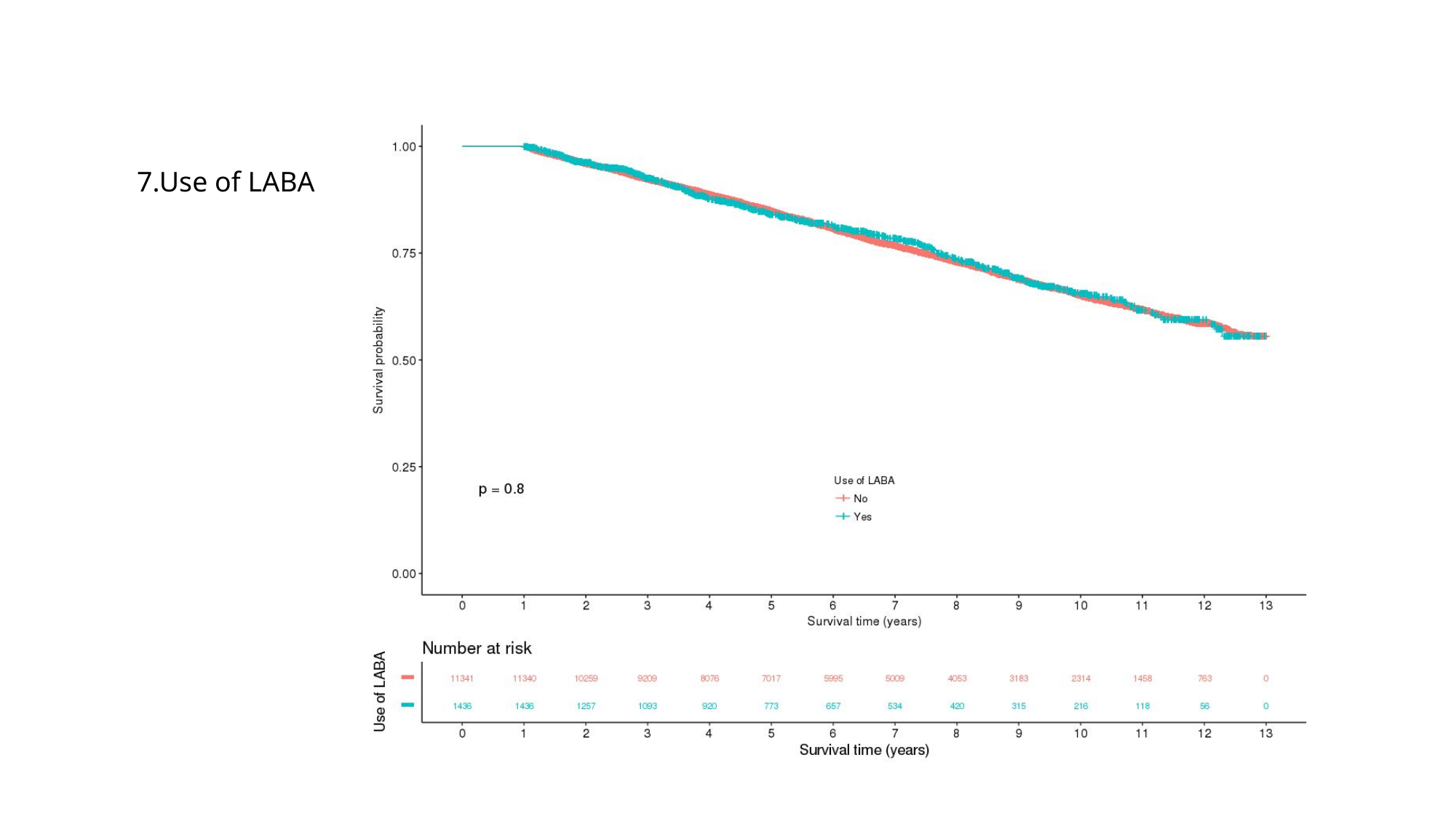

7.Use of LABA

## Slide 10
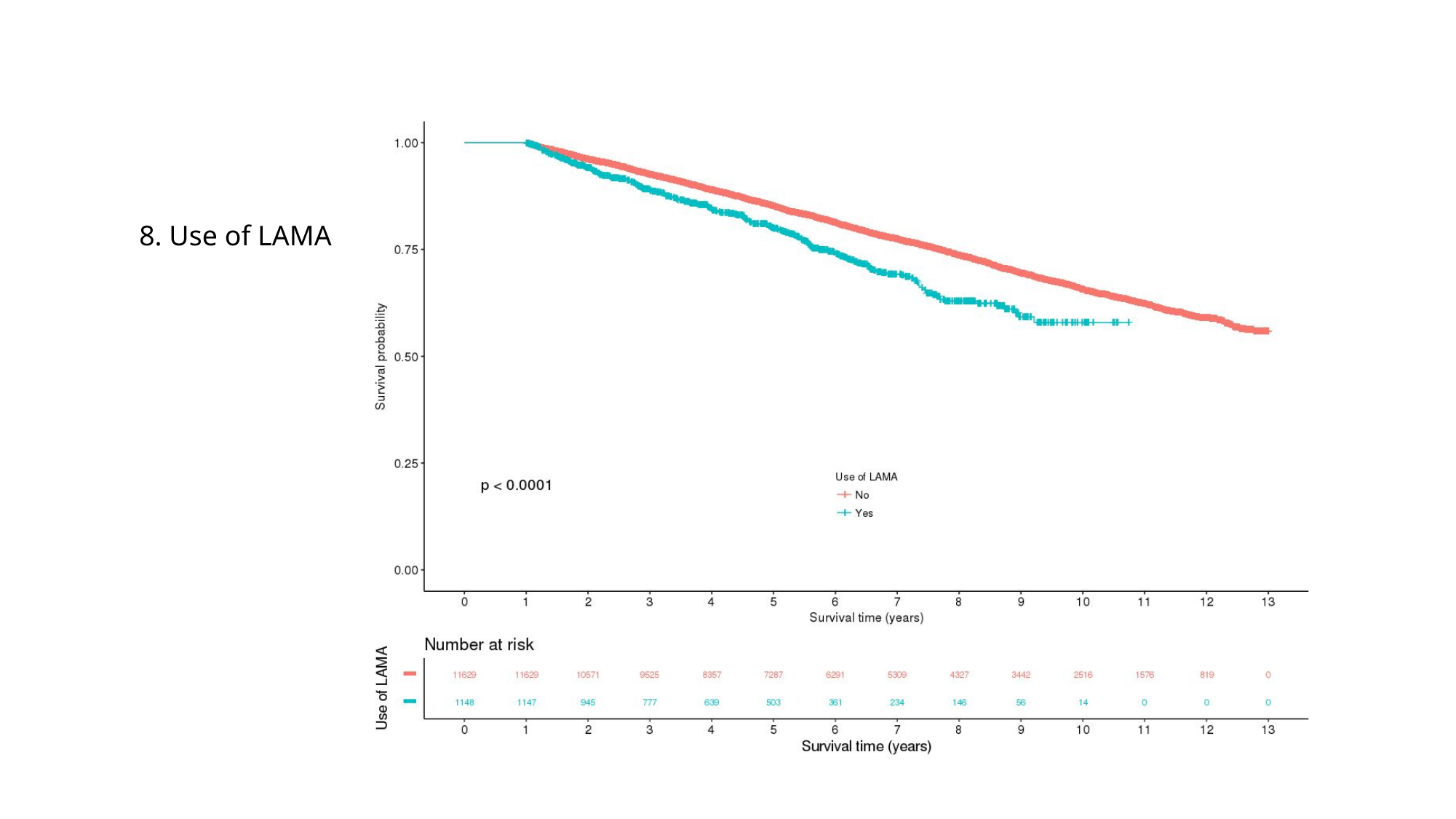

8. Use of LAMA

## Slide 11
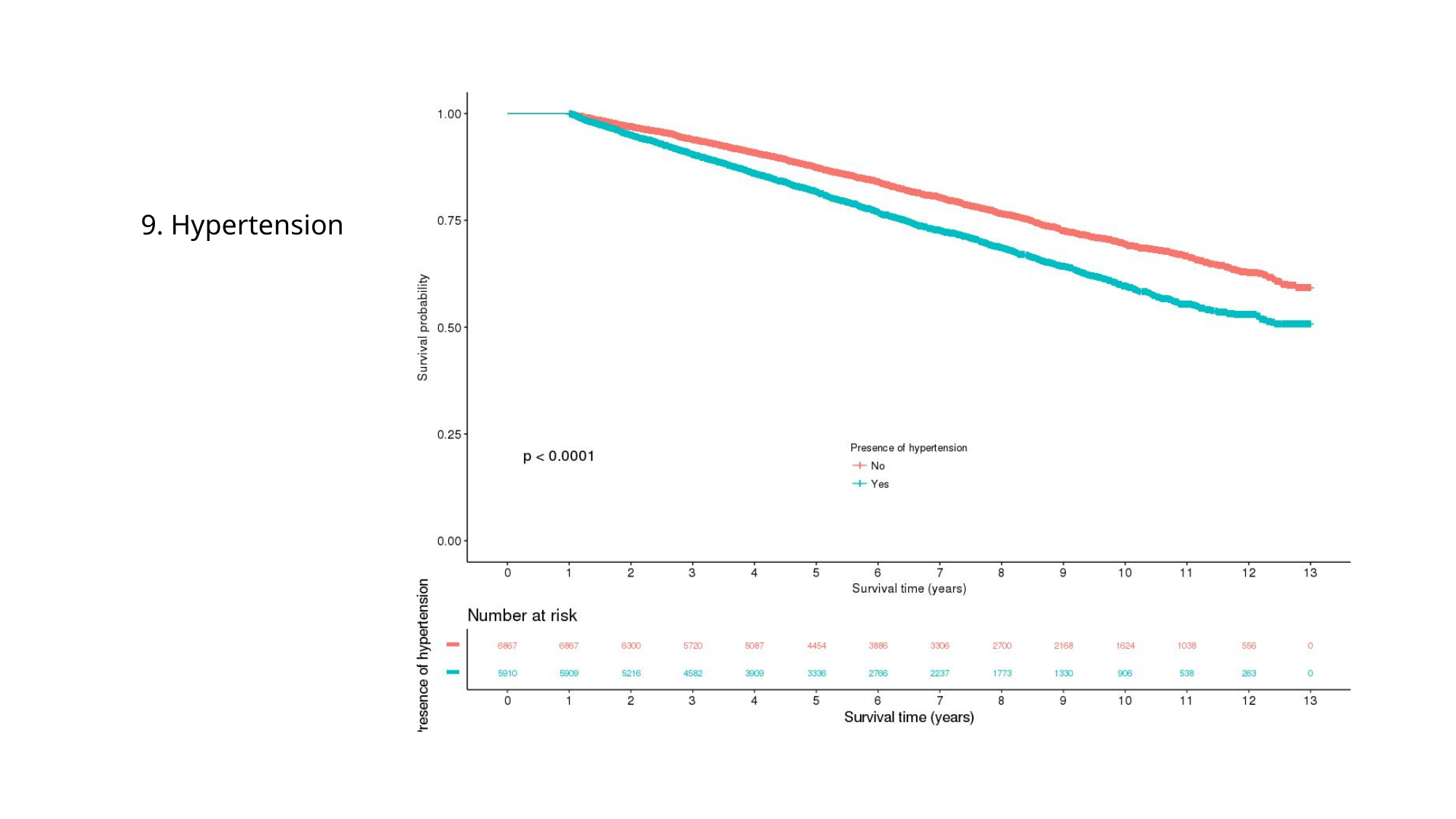

9. Hypertension

## Slide 12
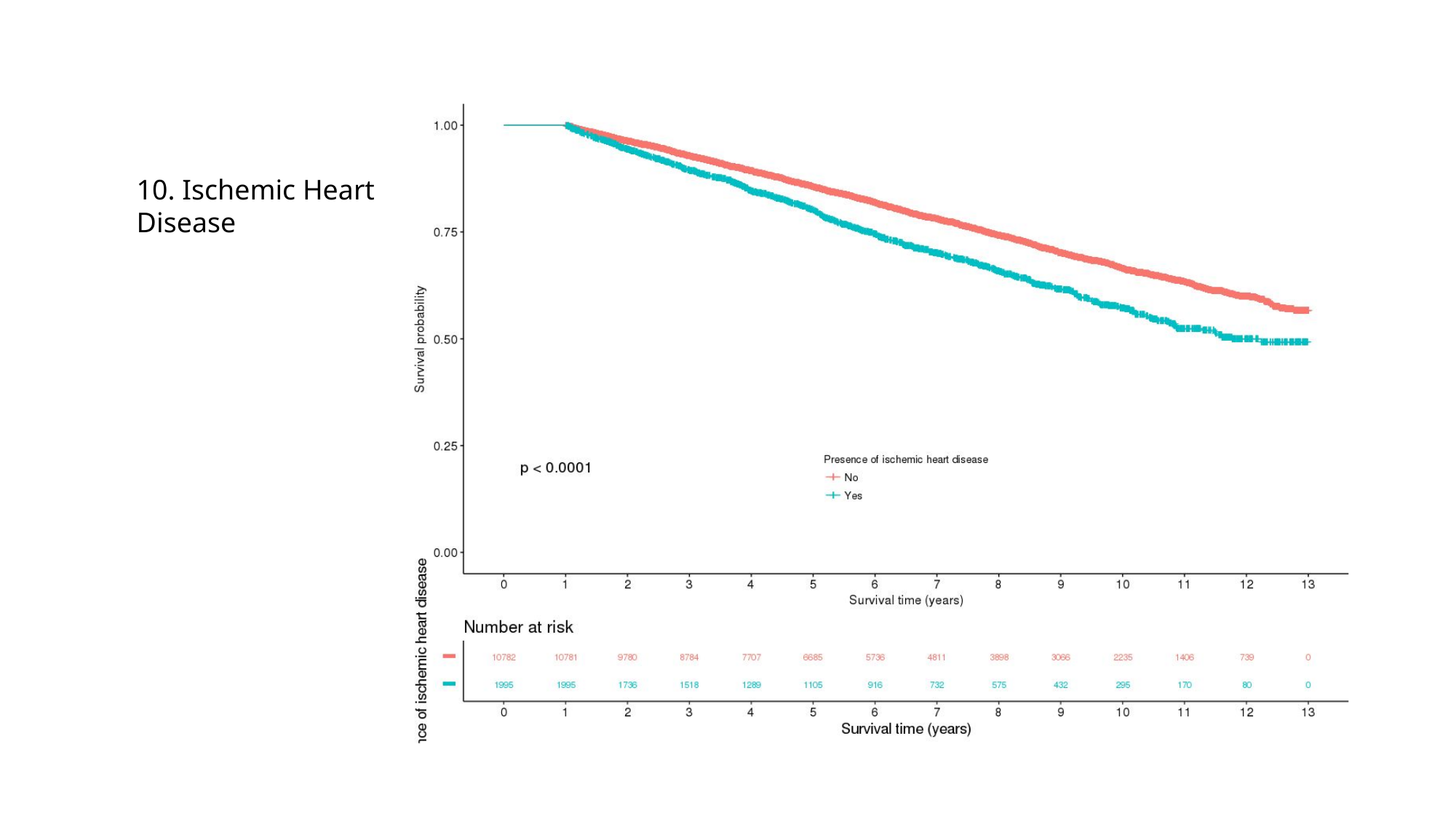

10. Ischemic Heart
Disease

## Slide 13
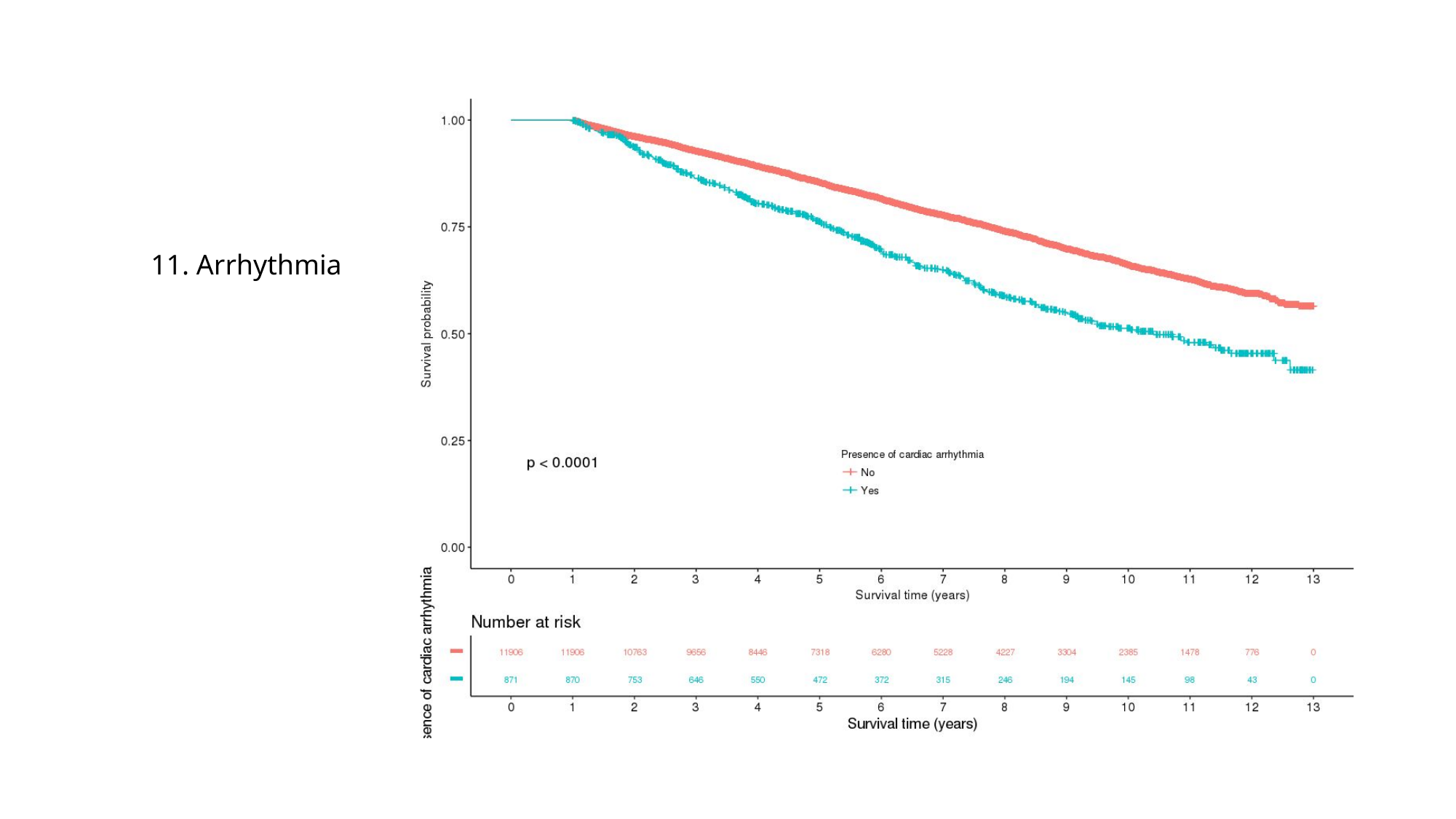

11. Arrhythmia

## Slide 14
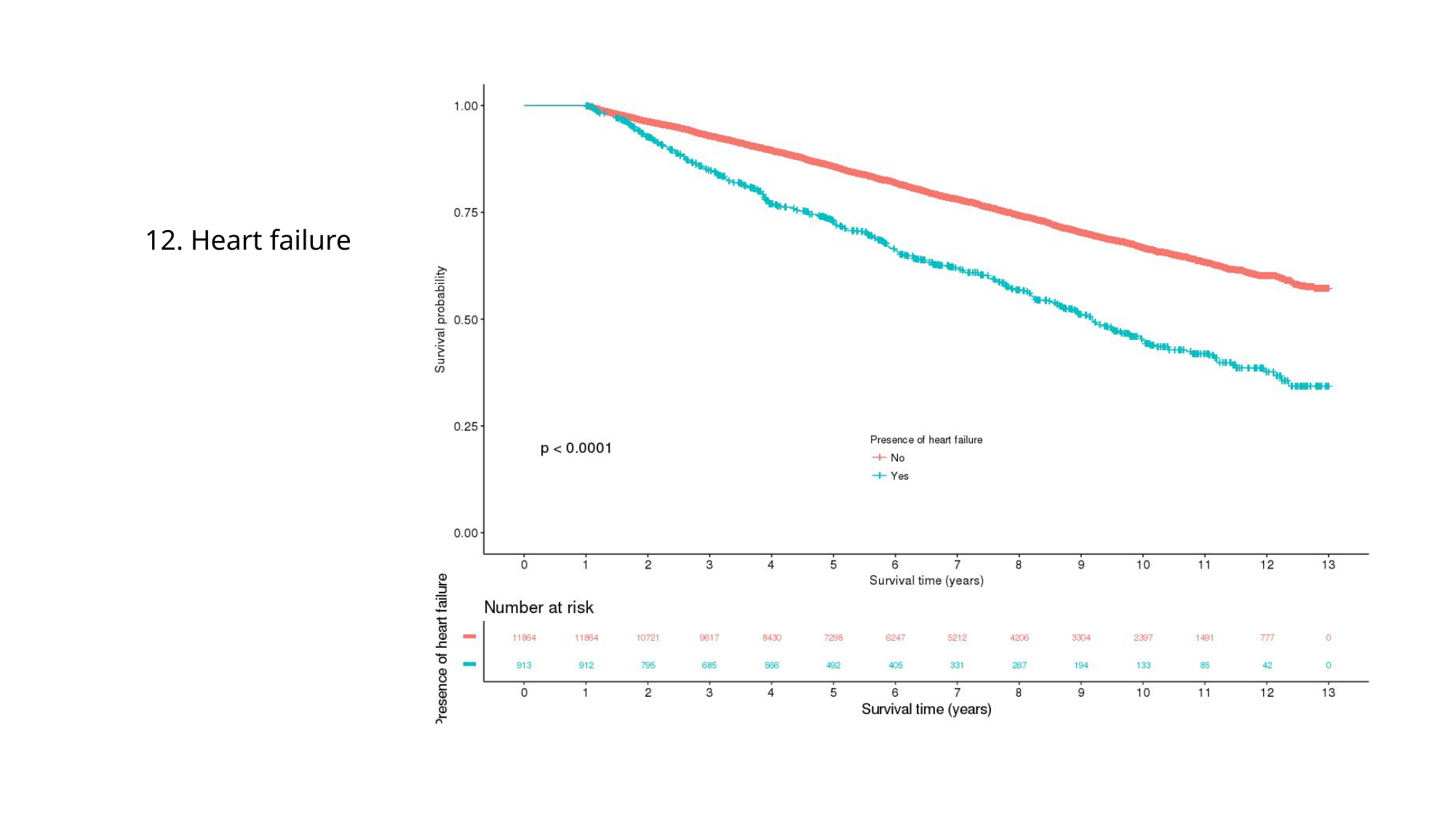

12. Heart failure

## Slide 15
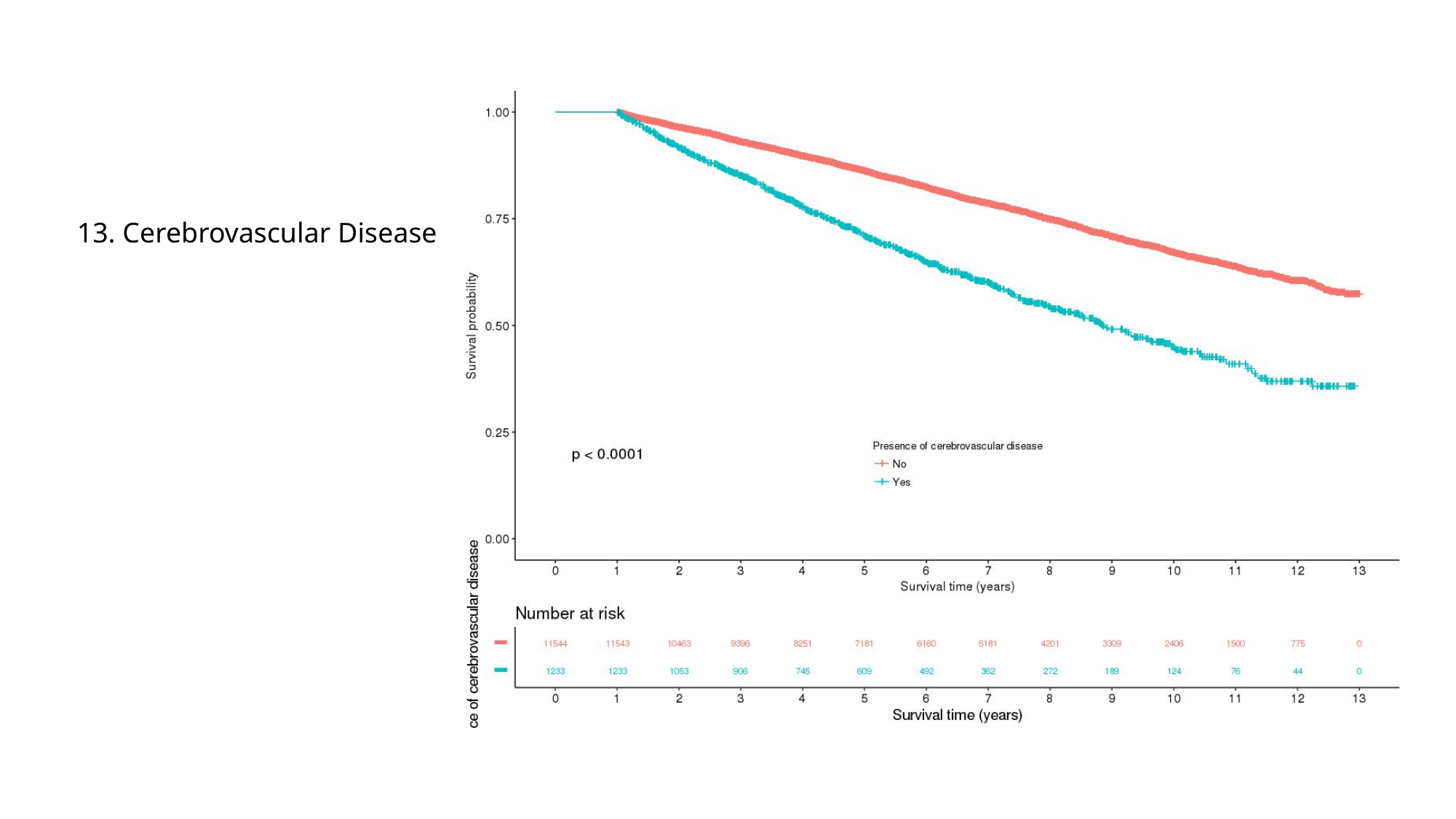

13. Cerebrovascular Disease

## Slide 16
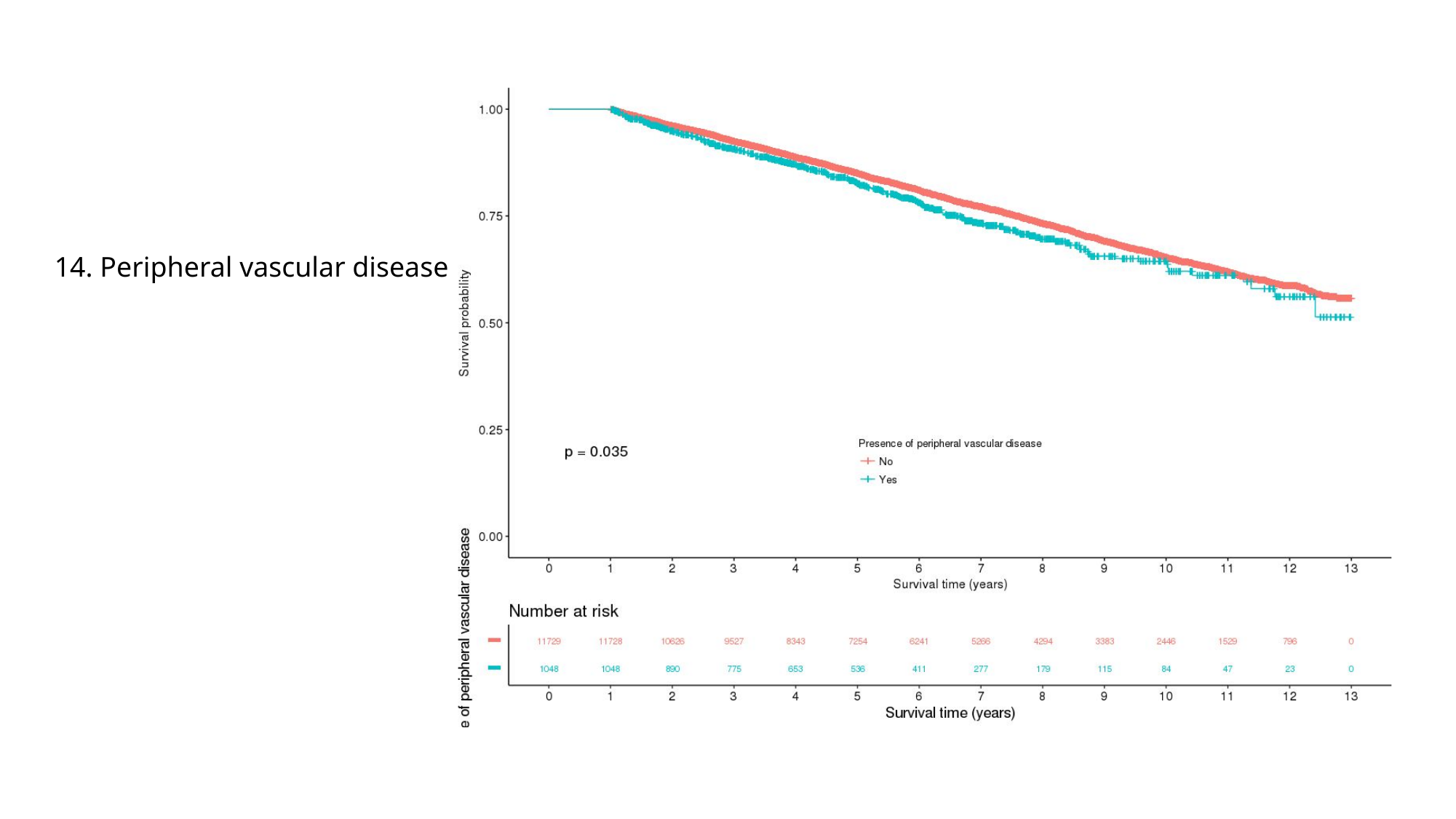

14. Peripheral vascular disease

## Slide 17
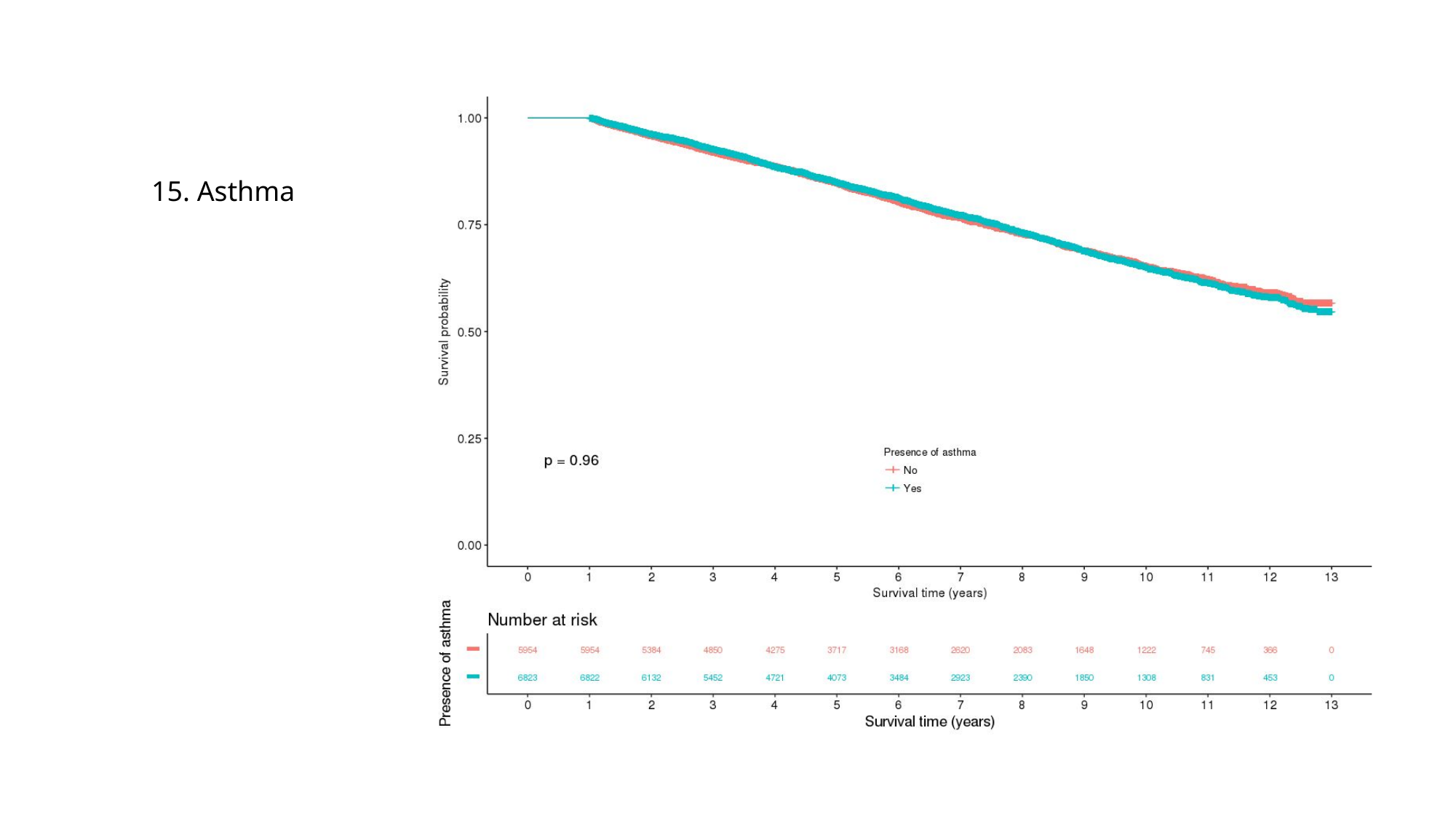

15. Asthma

## Slide 18
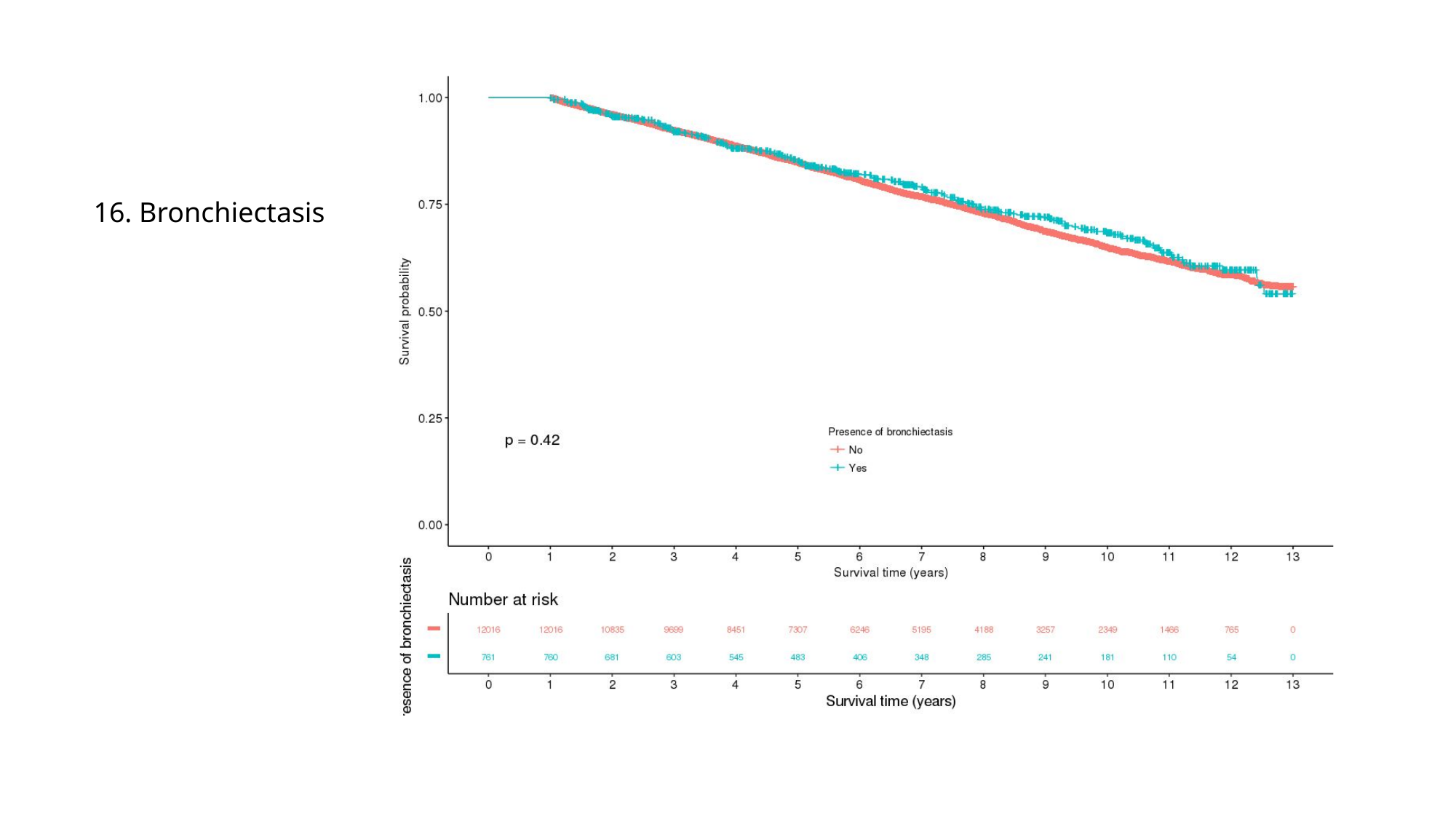

16. Bronchiectasis

## Slide 19
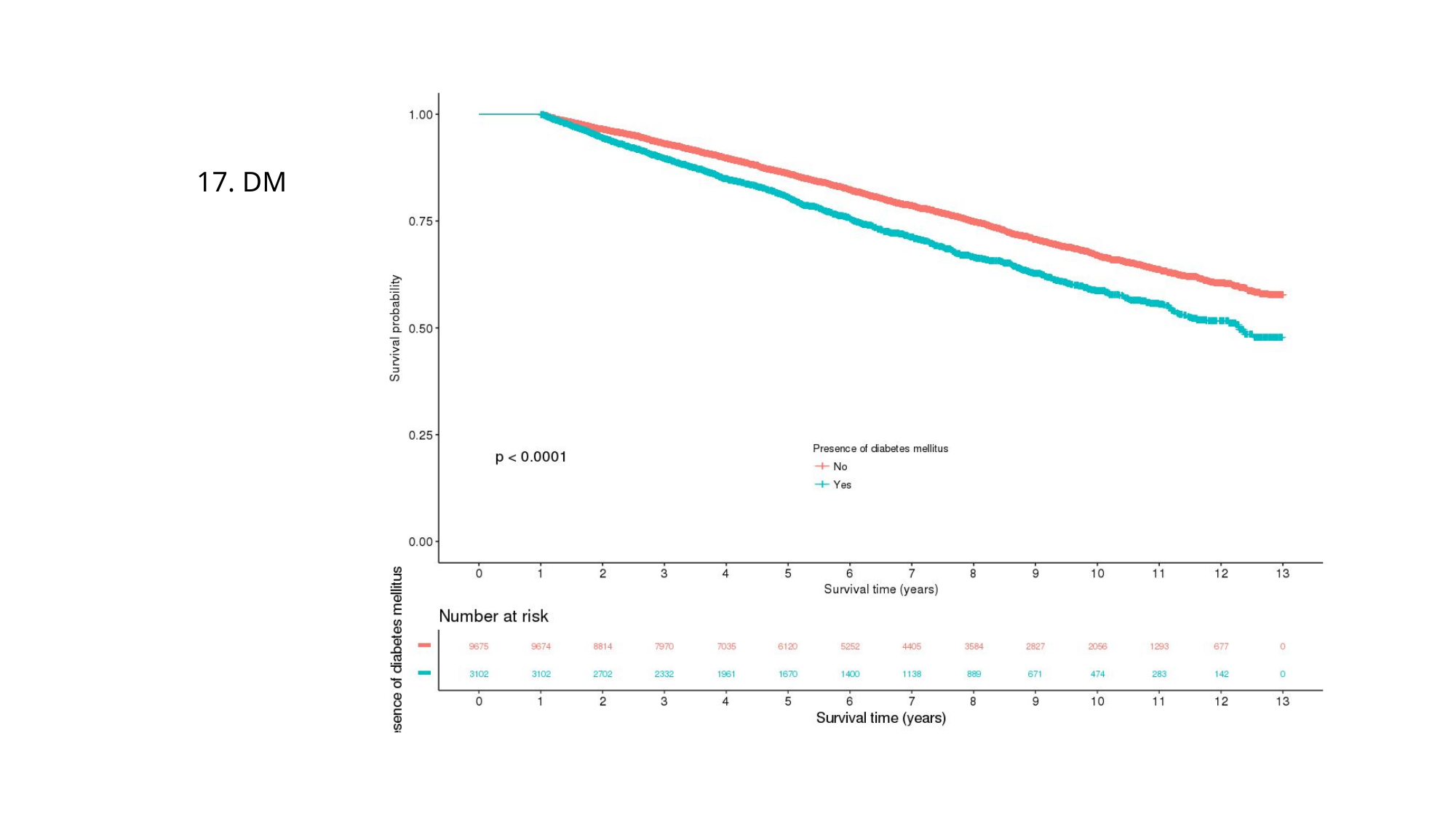

17. DM

## Slide 20
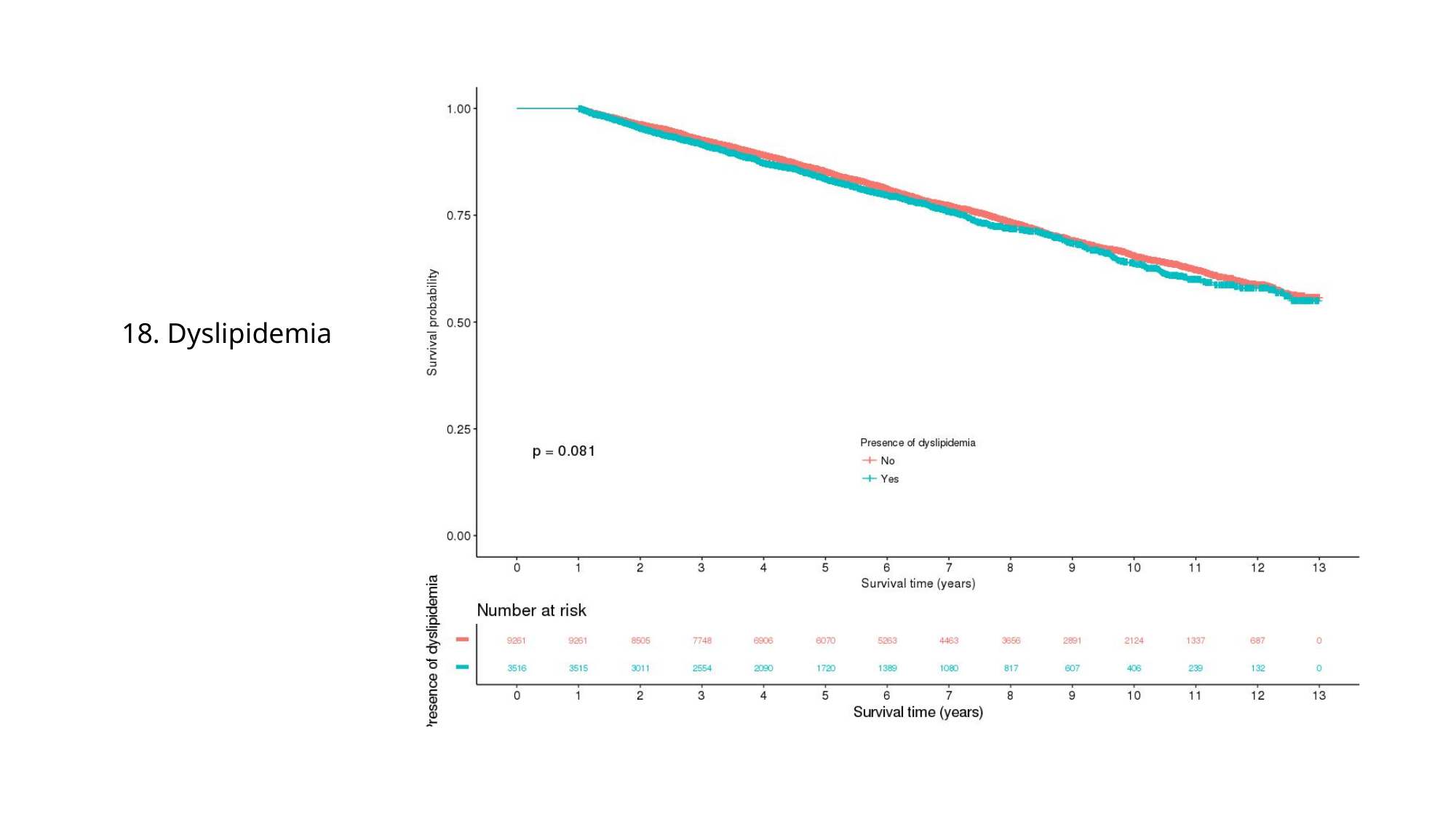

18. Dyslipidemia

## Slide 21
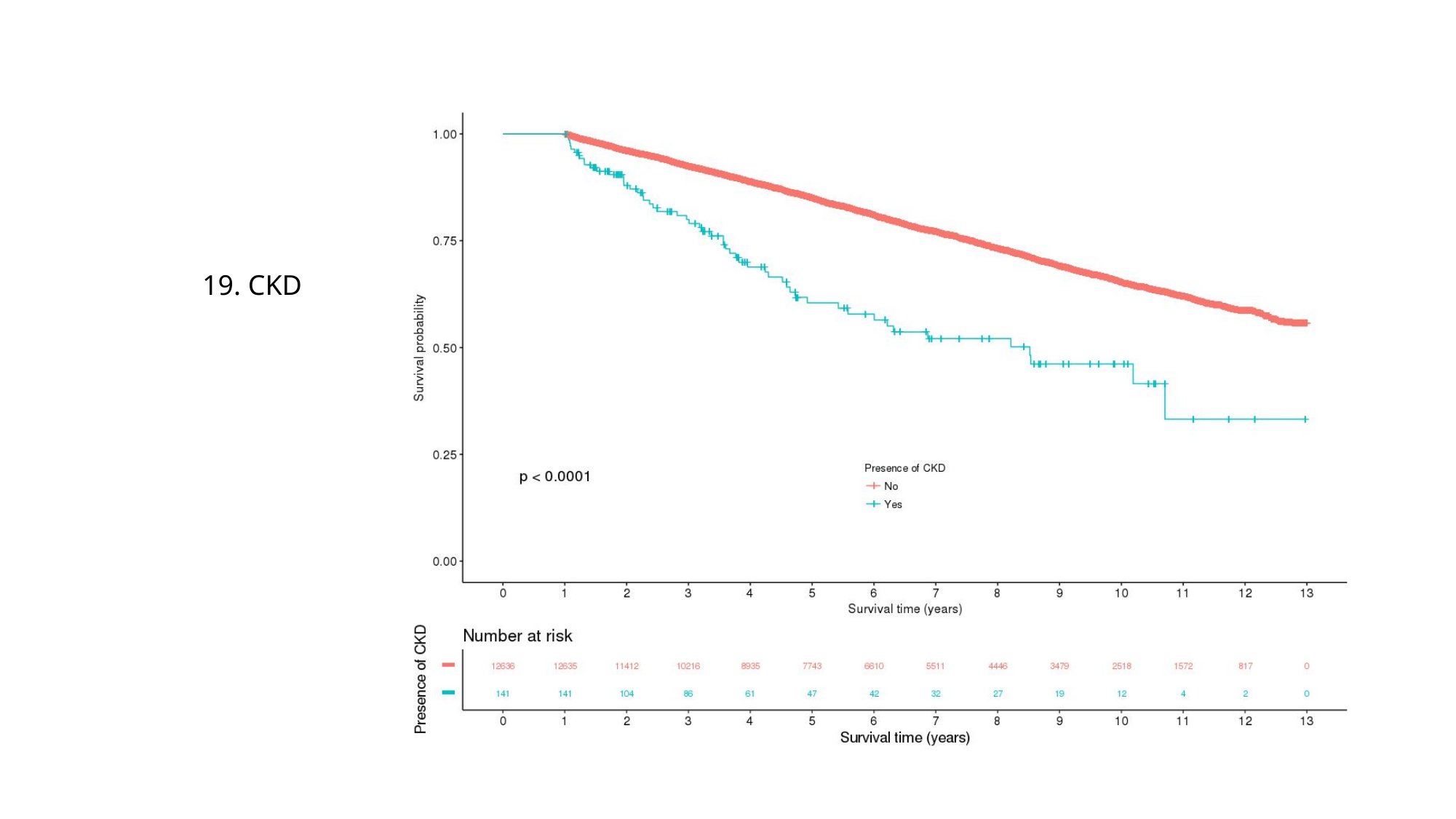

19. CKD

## Slide 22
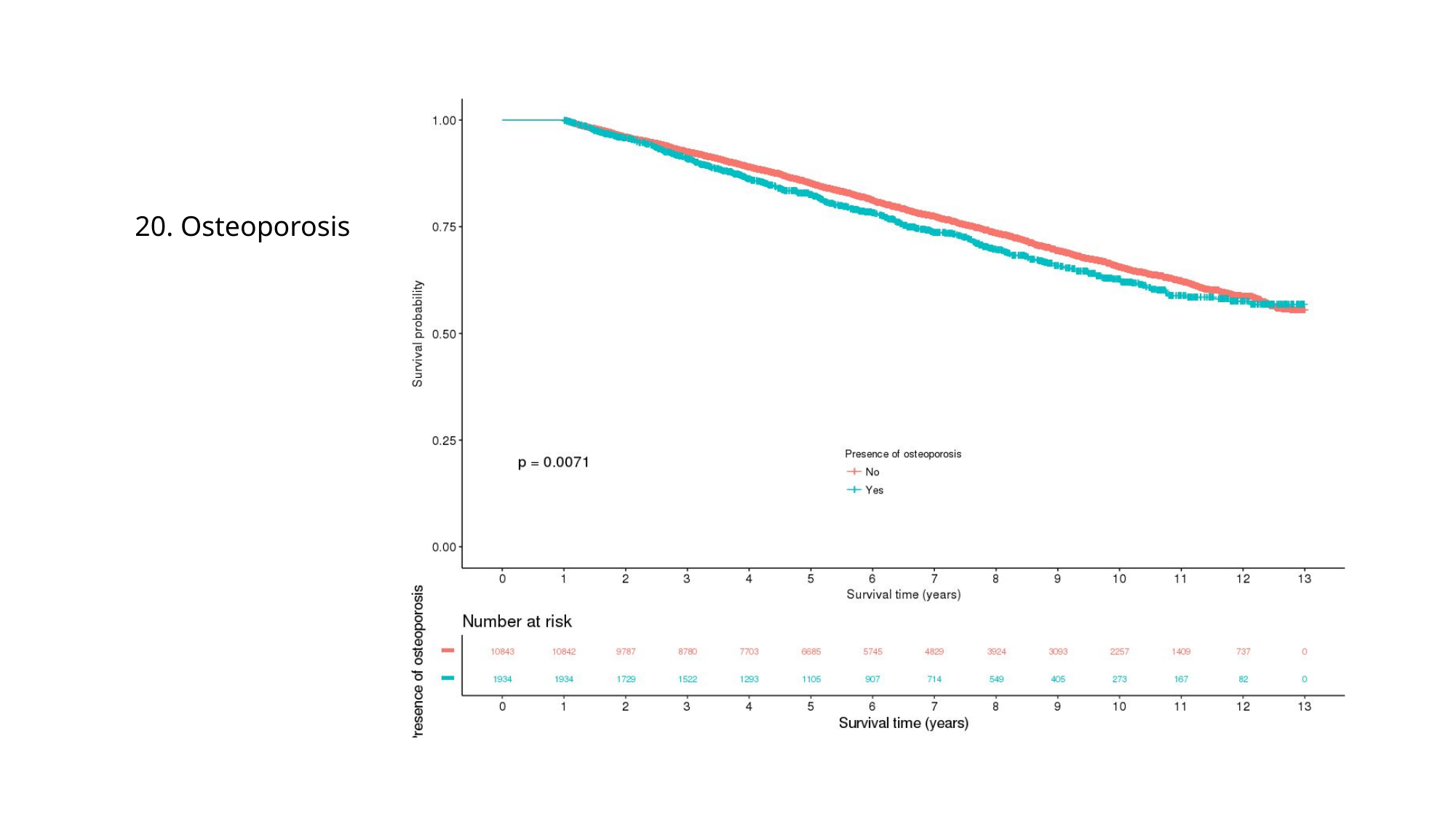

20. Osteoporosis

## Slide 23
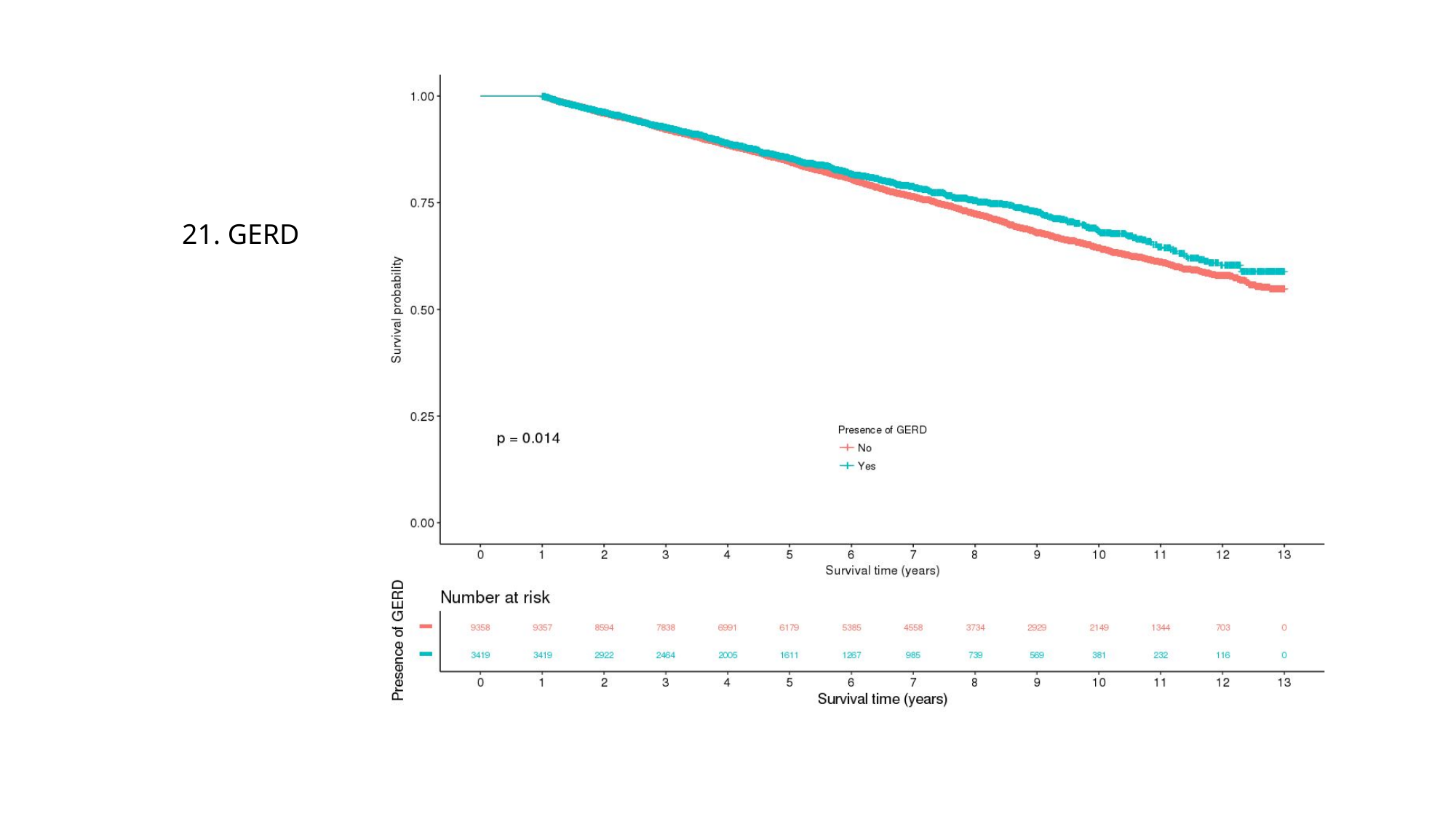

21. GERD

## Slide 24
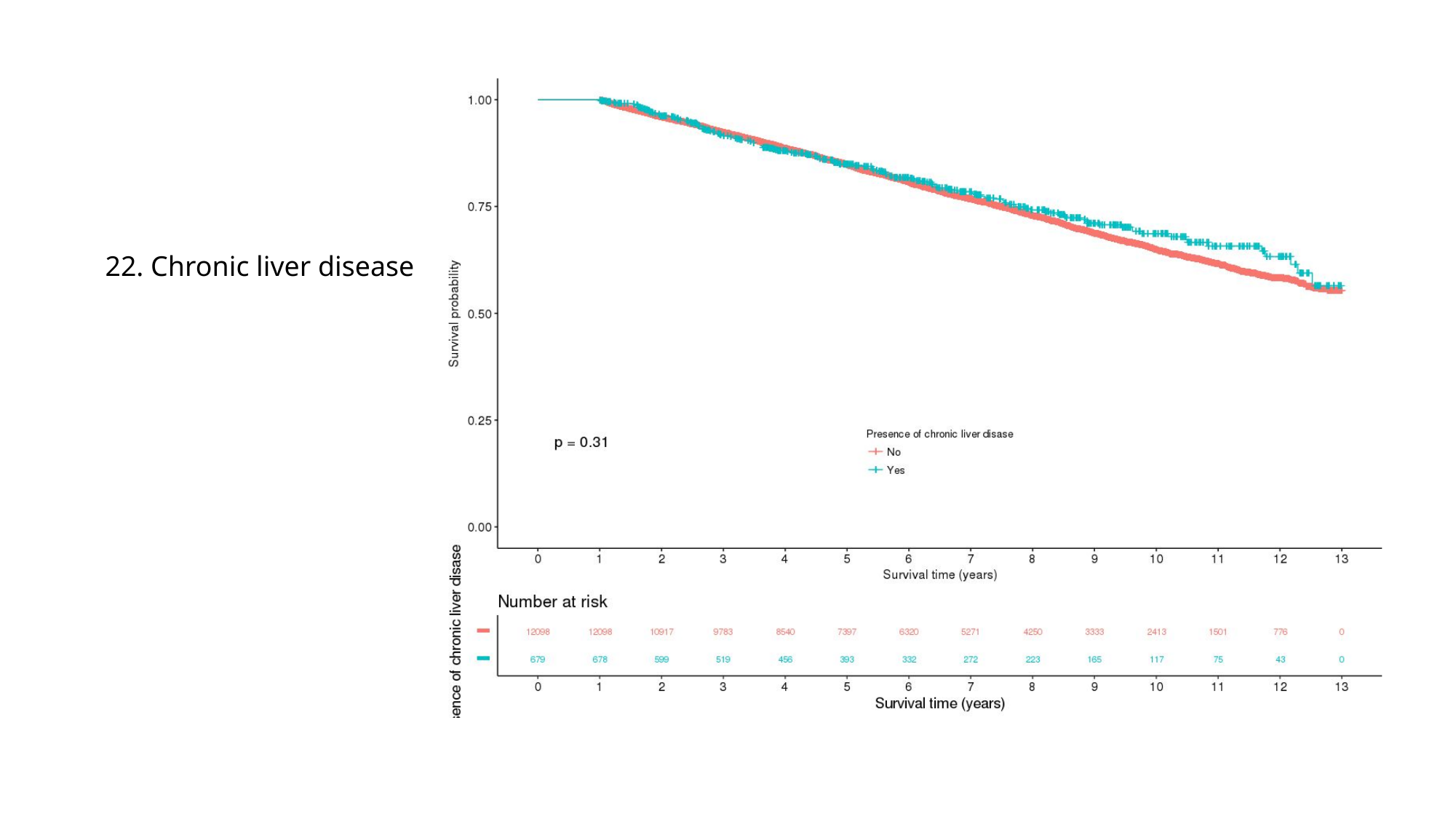

22. Chronic liver disease

## Slide 25
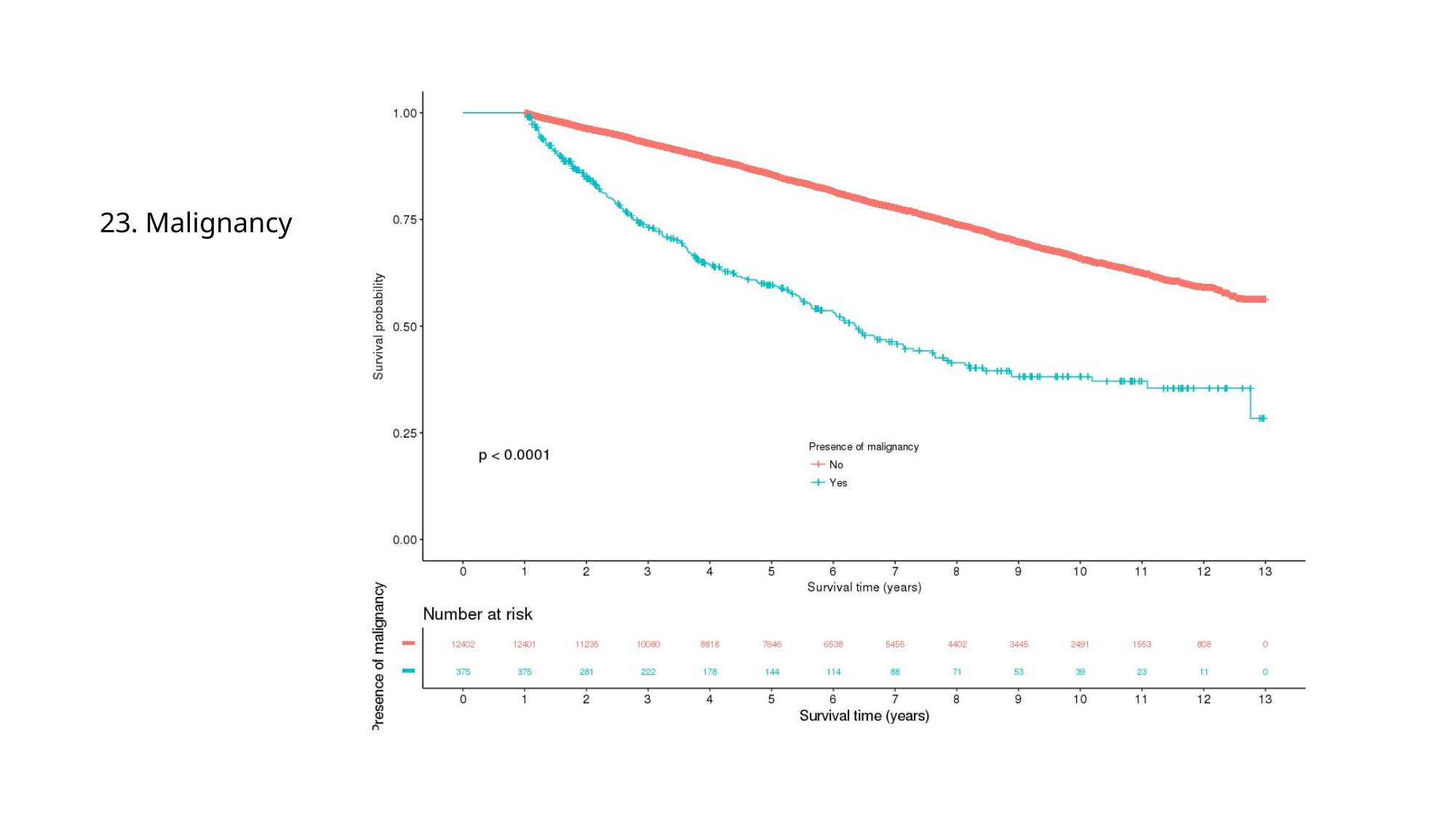

23. Malignancy

## Slide 26
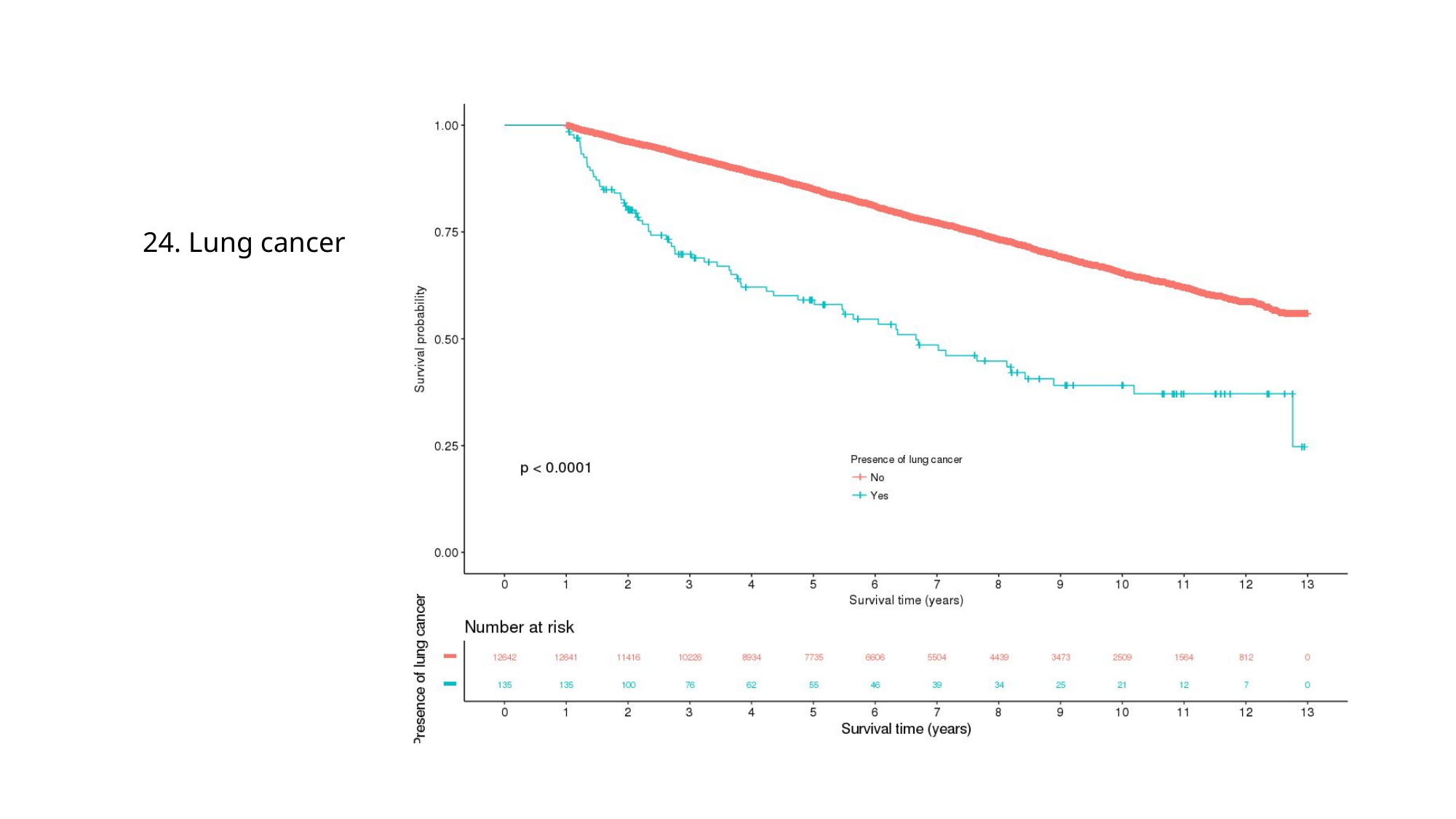

24. Lung cancer

## Slide 27
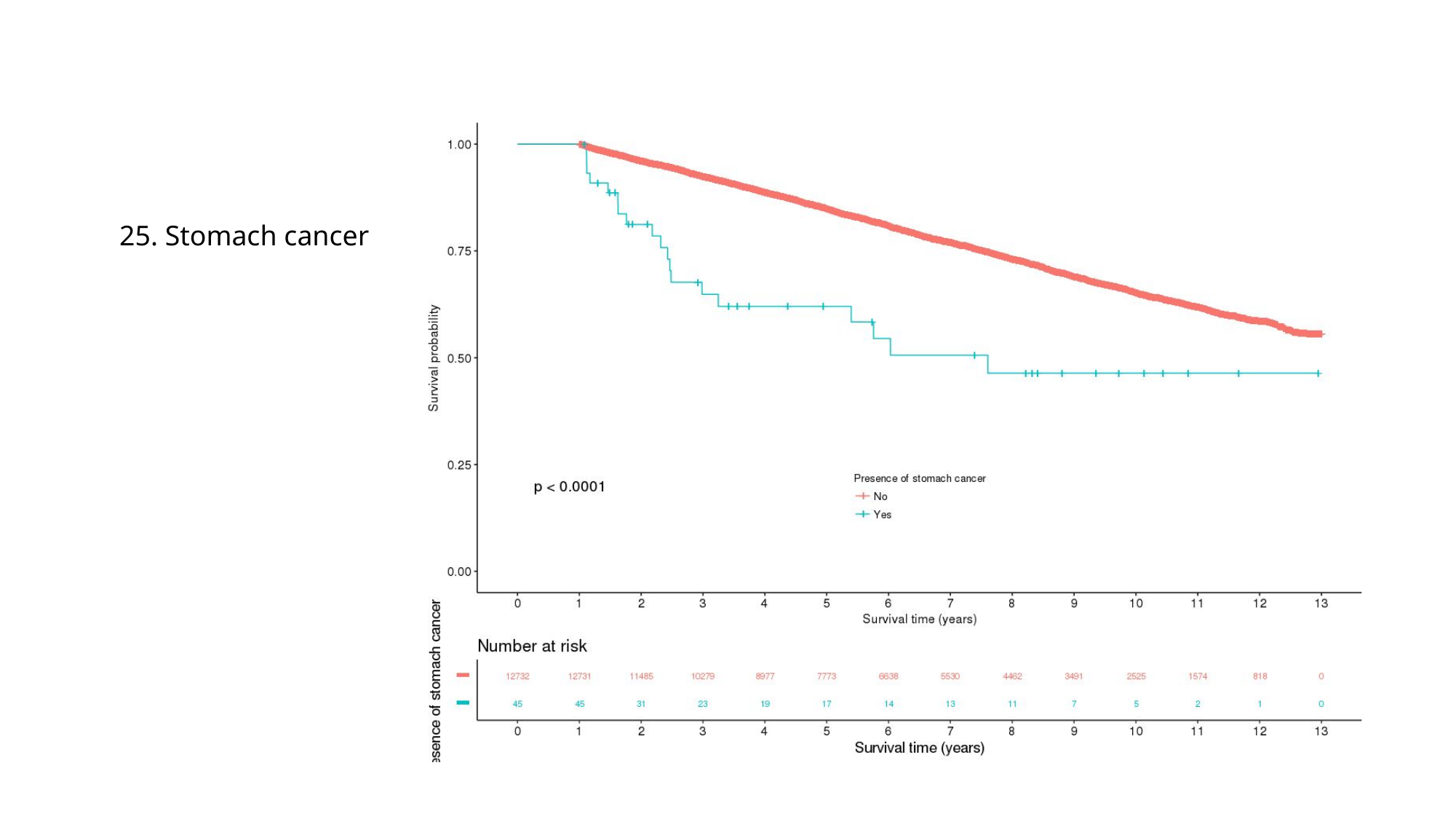

25. Stomach cancer

## Slide 28
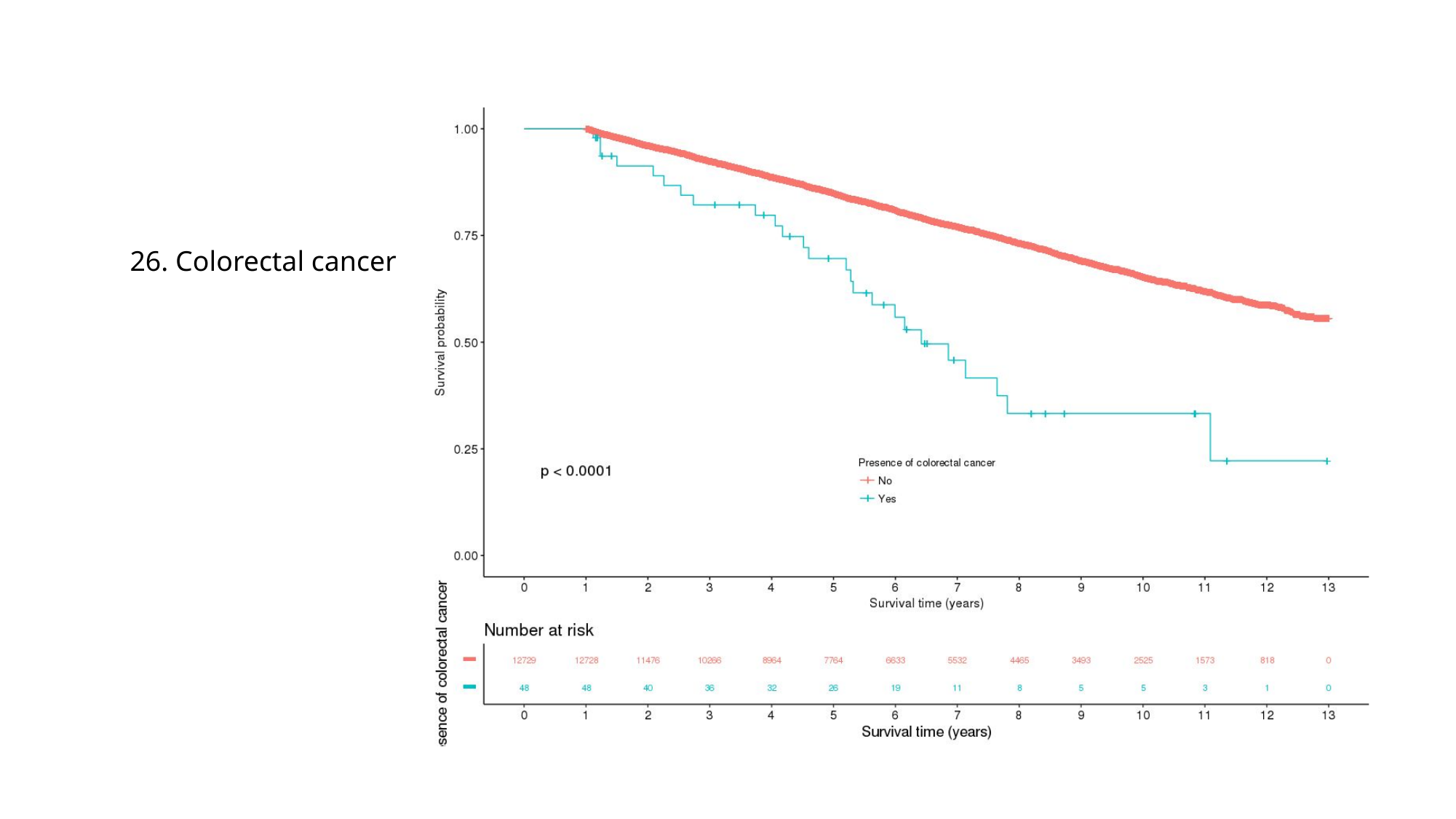

26. Colorectal cancer

## Slide 29
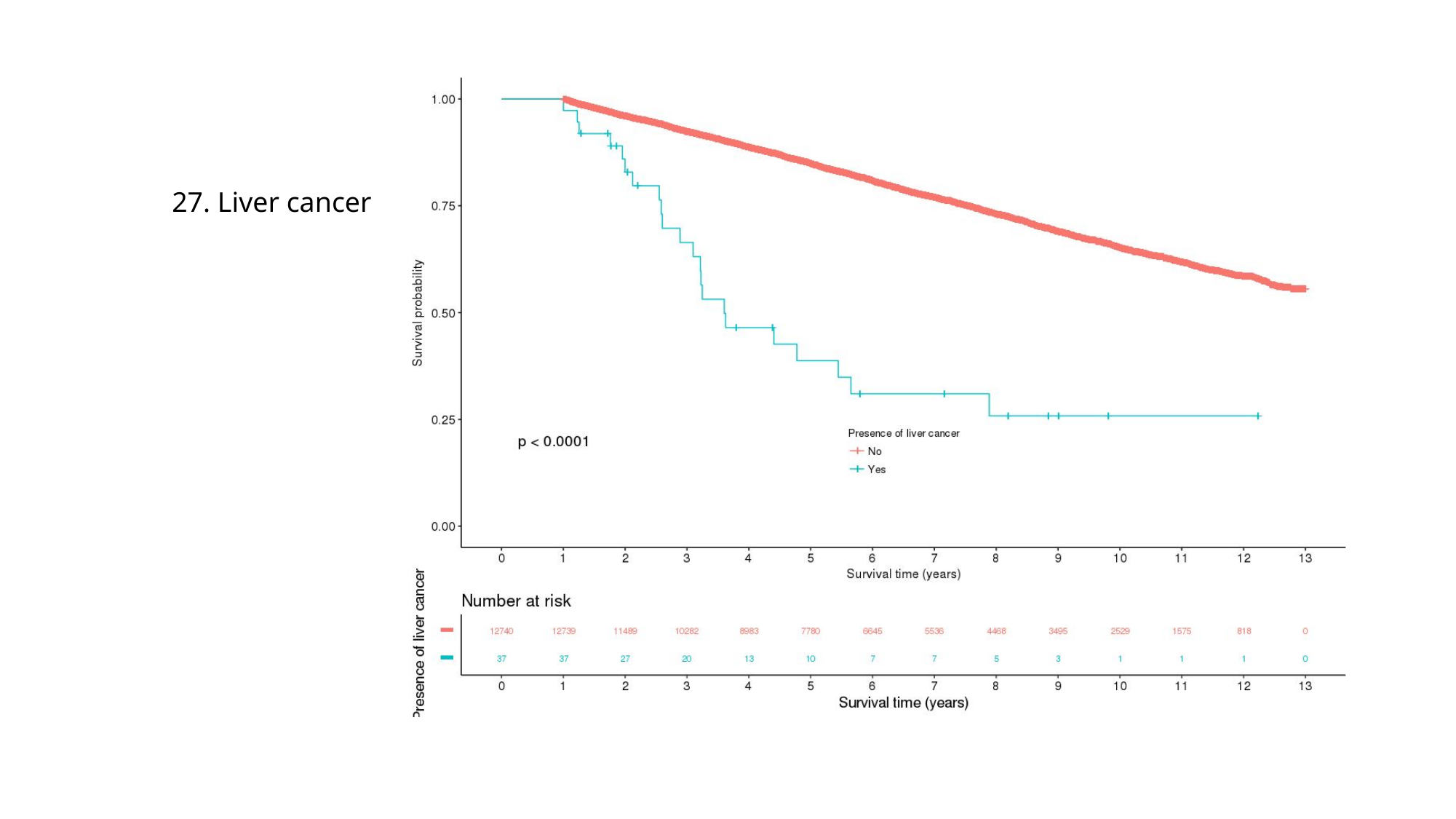

27. Liver cancer

## Slide 30
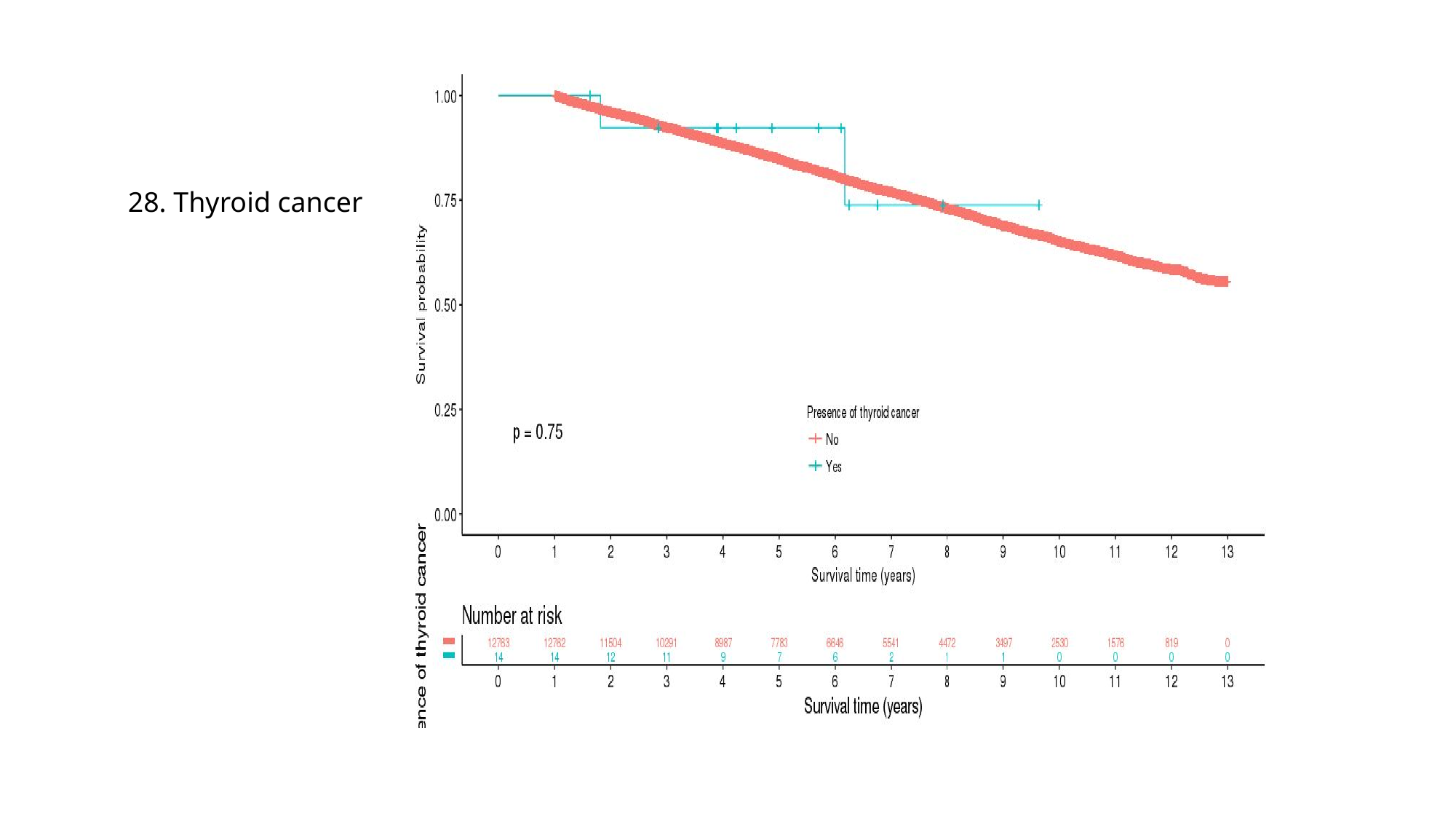

28. Thyroid cancer

## Slide 31
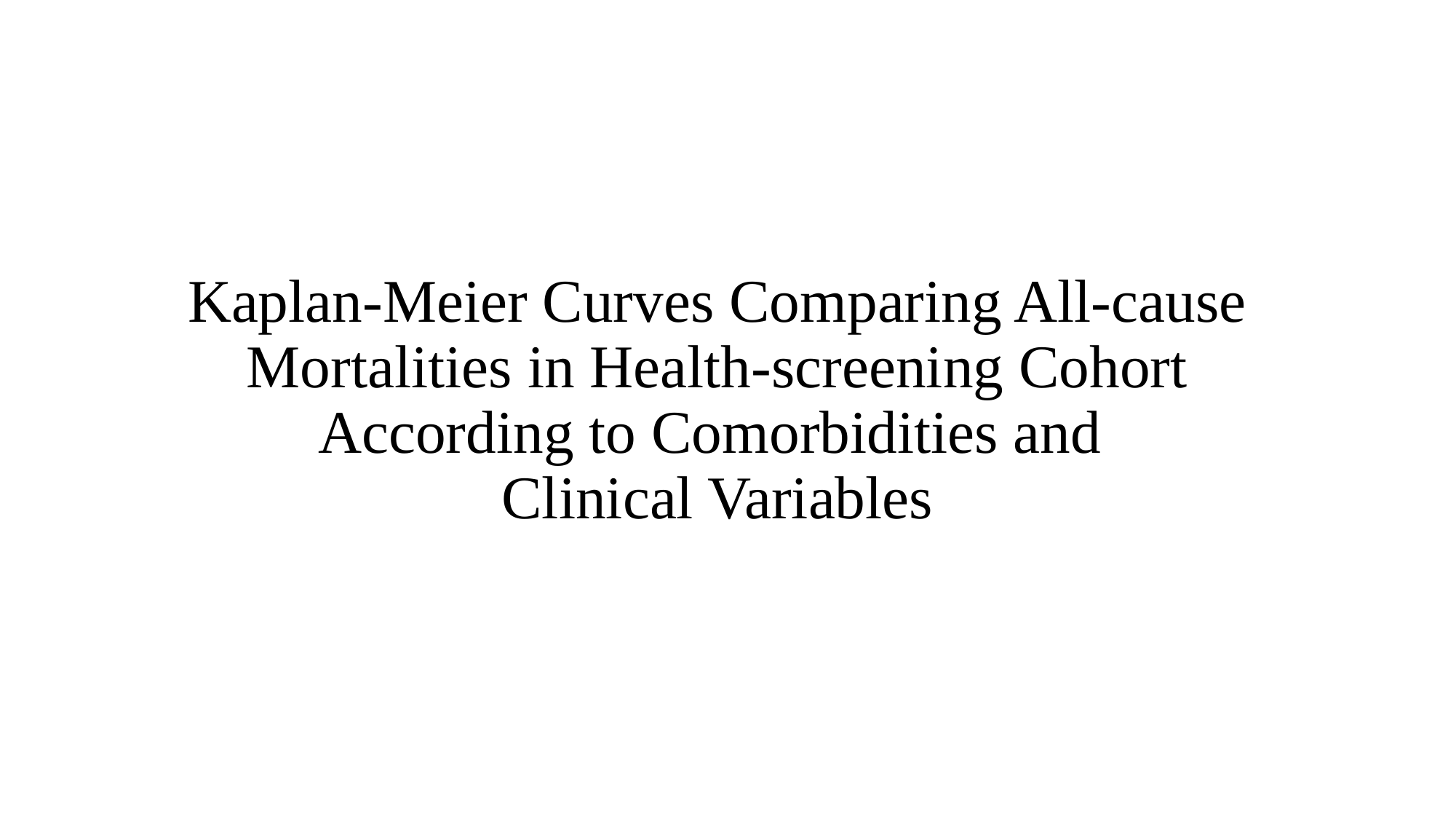

# Kaplan-Meier Curves Comparing All-cause Mortalities in Health-screening Cohort According to Comorbidities and Clinical Variables

## Slide 32
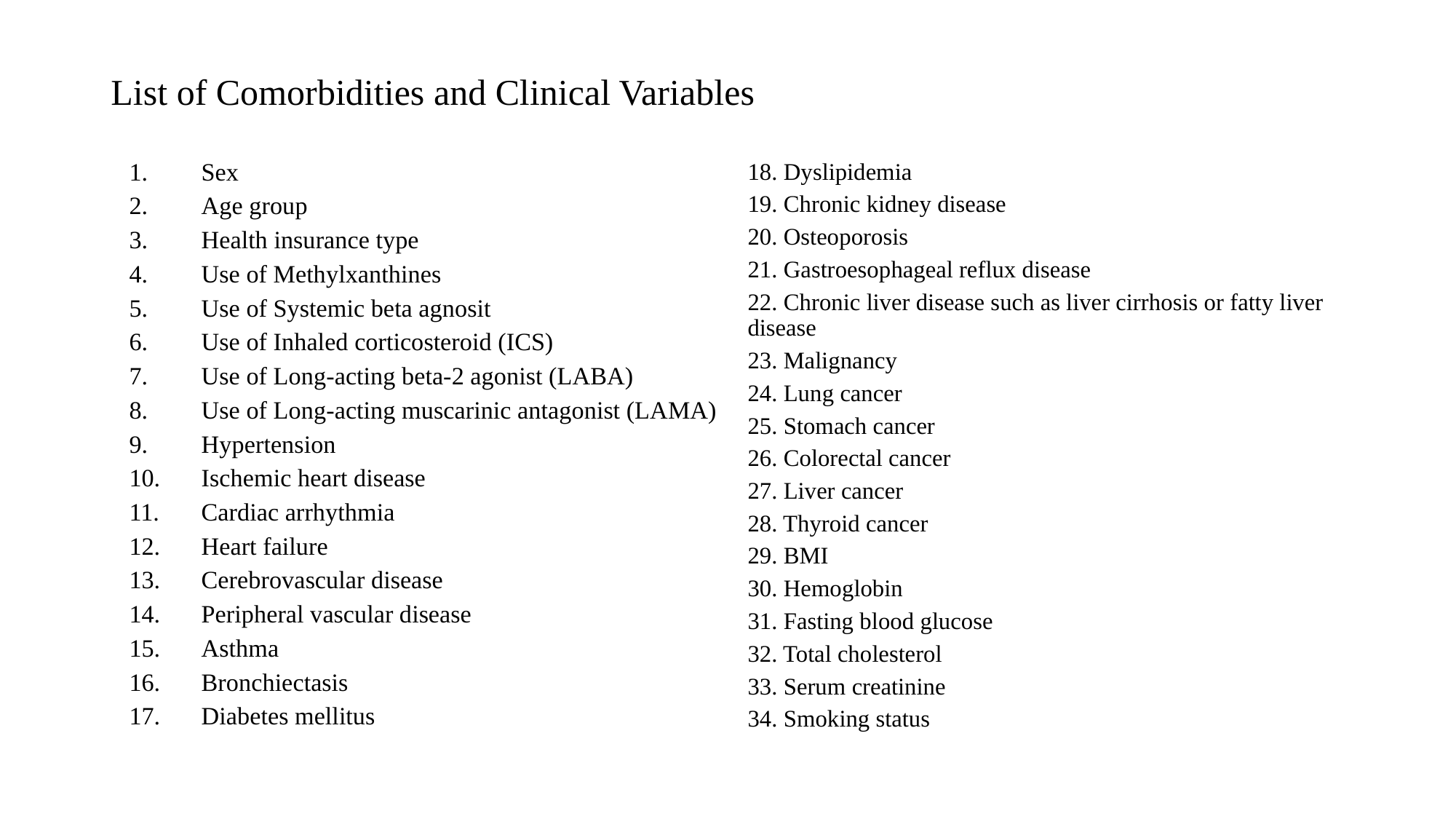

# List of Comorbidities and Clinical Variables
Sex
Age group
Health insurance type
Use of Methylxanthines
Use of Systemic beta agnosit
Use of Inhaled corticosteroid (ICS)
Use of Long-acting beta-2 agonist (LABA)
Use of Long-acting muscarinic antagonist (LAMA)
Hypertension
Ischemic heart disease
Cardiac arrhythmia
Heart failure
Cerebrovascular disease
Peripheral vascular disease
Asthma
Bronchiectasis
Diabetes mellitus
18. Dyslipidemia
19. Chronic kidney disease
20. Osteoporosis
21. Gastroesophageal reflux disease
22. Chronic liver disease such as liver cirrhosis or fatty liver disease
23. Malignancy
24. Lung cancer
25. Stomach cancer
26. Colorectal cancer
27. Liver cancer
28. Thyroid cancer
29. BMI
30. Hemoglobin
31. Fasting blood glucose
32. Total cholesterol
33. Serum creatinine
34. Smoking status

## Slide 33
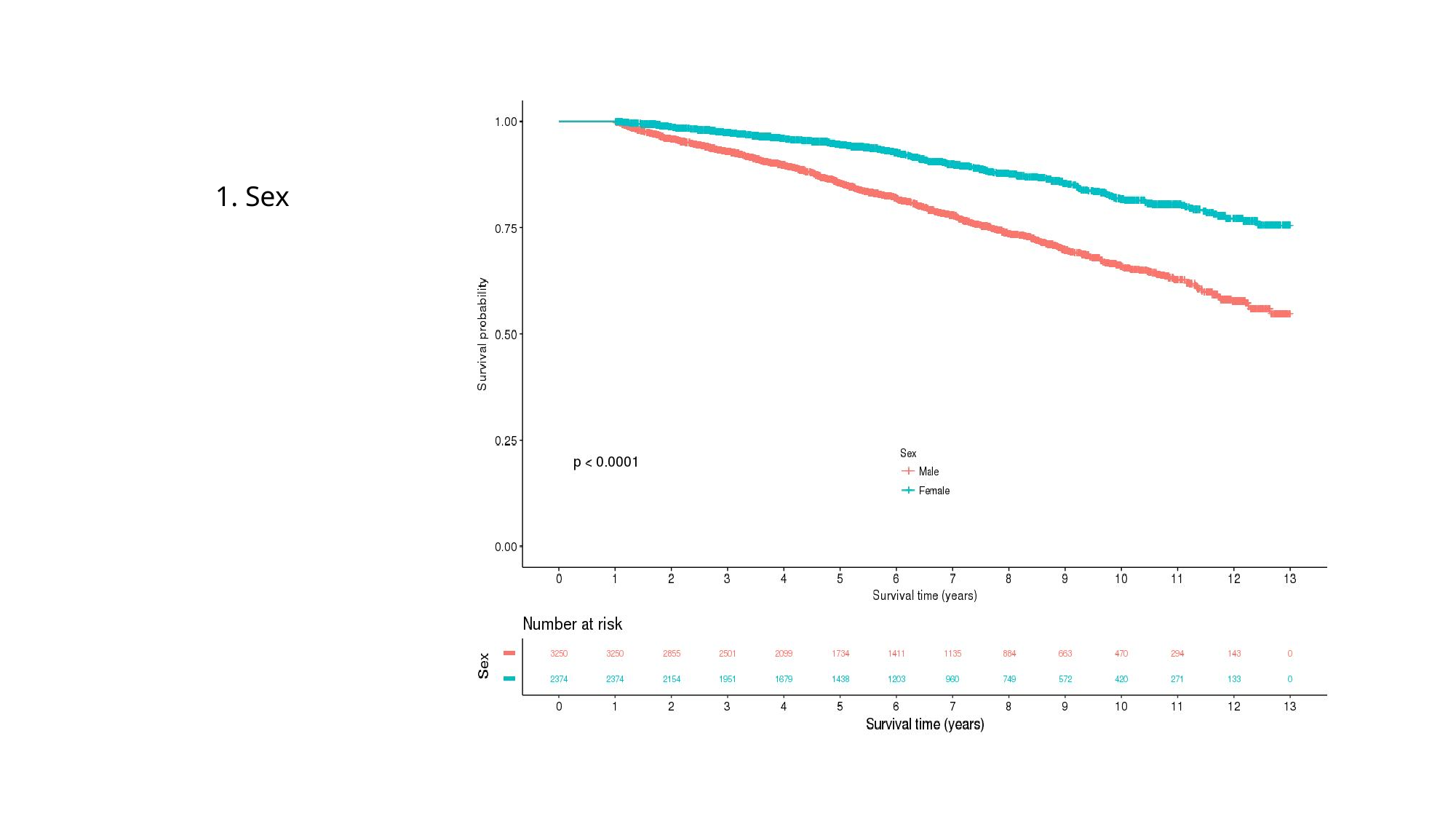

1. Sex

## Slide 34
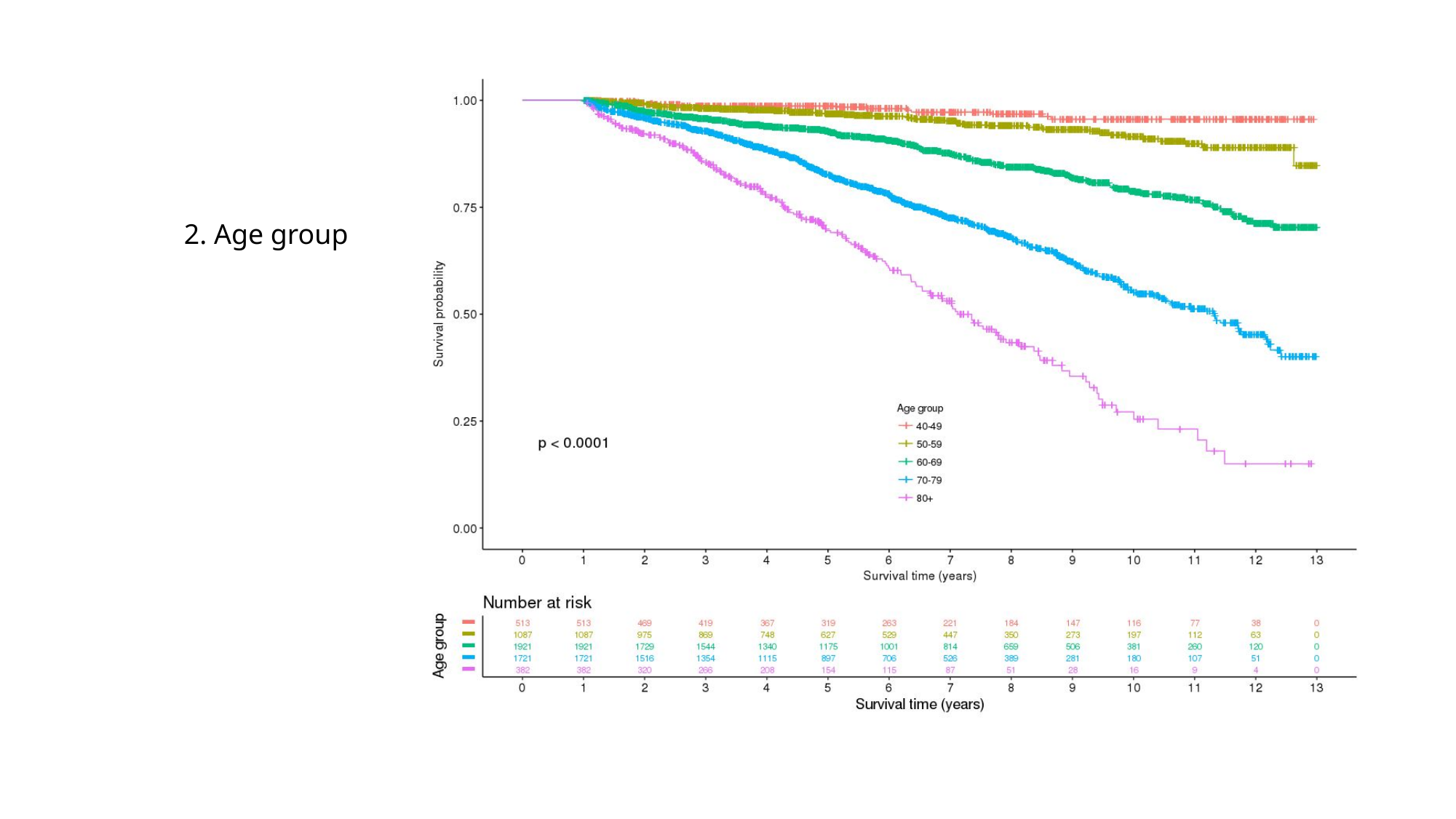

2. Age group

## Slide 35
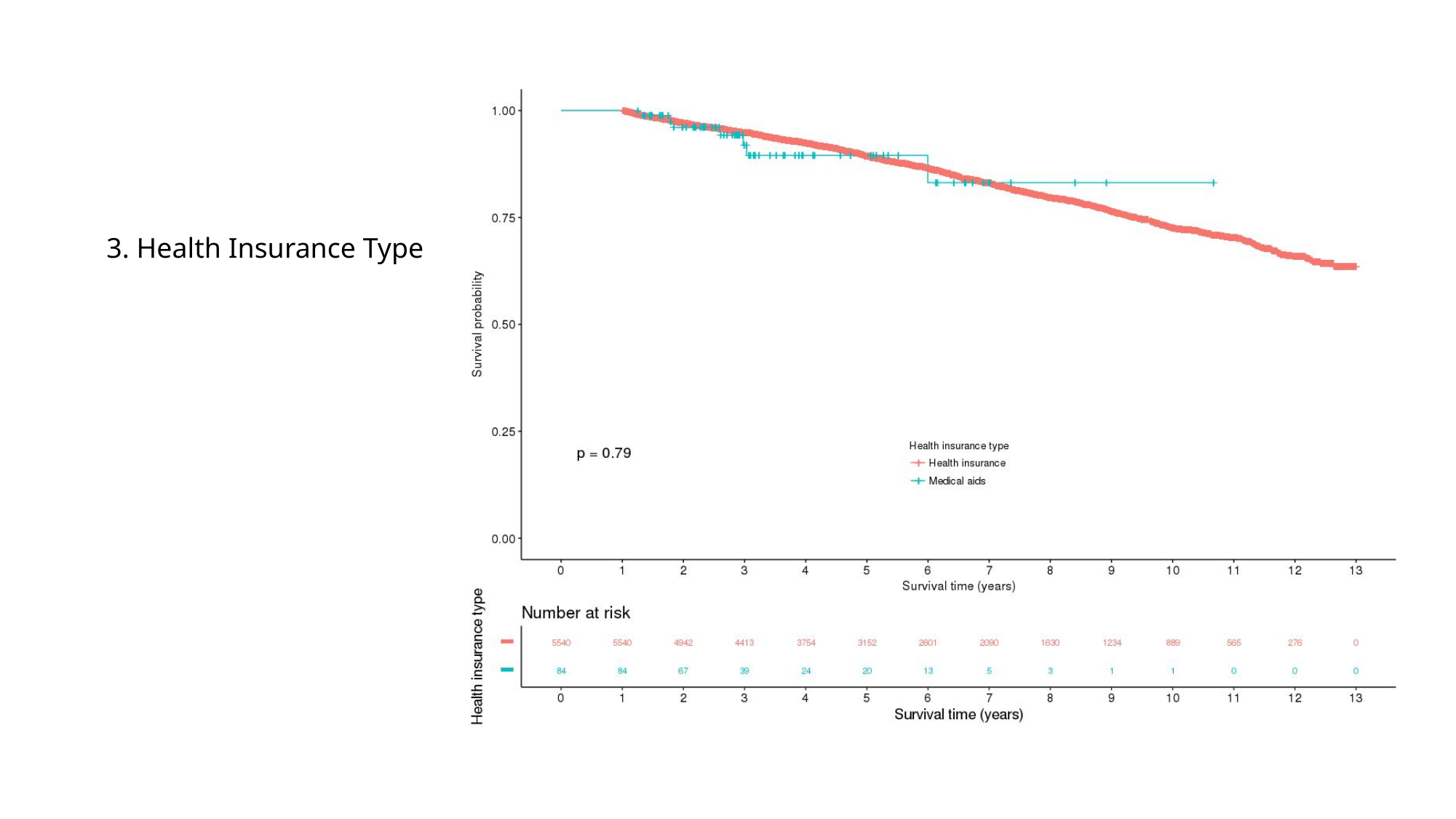

3. Health Insurance Type

## Slide 36
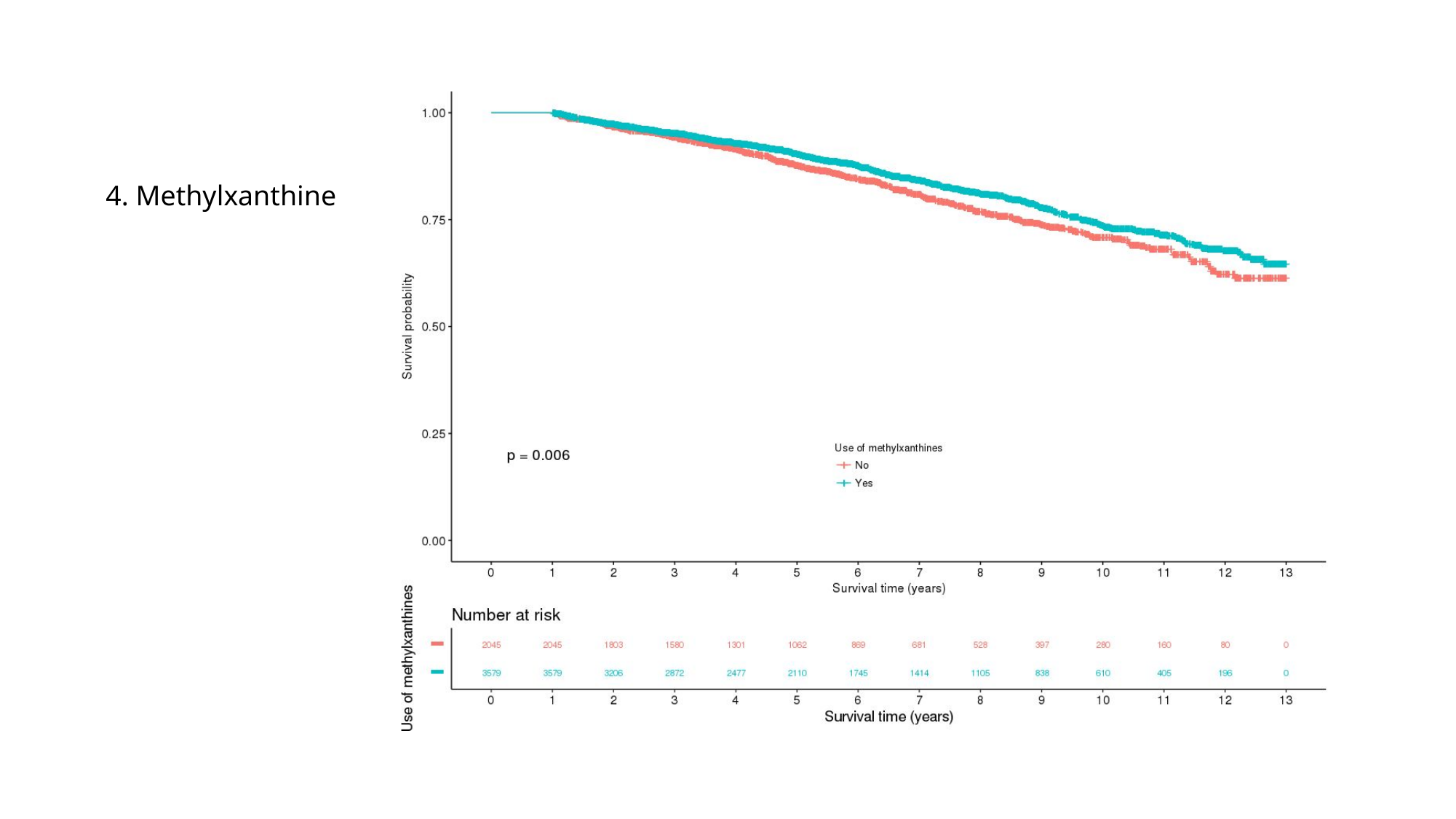

4. Methylxanthine

## Slide 37
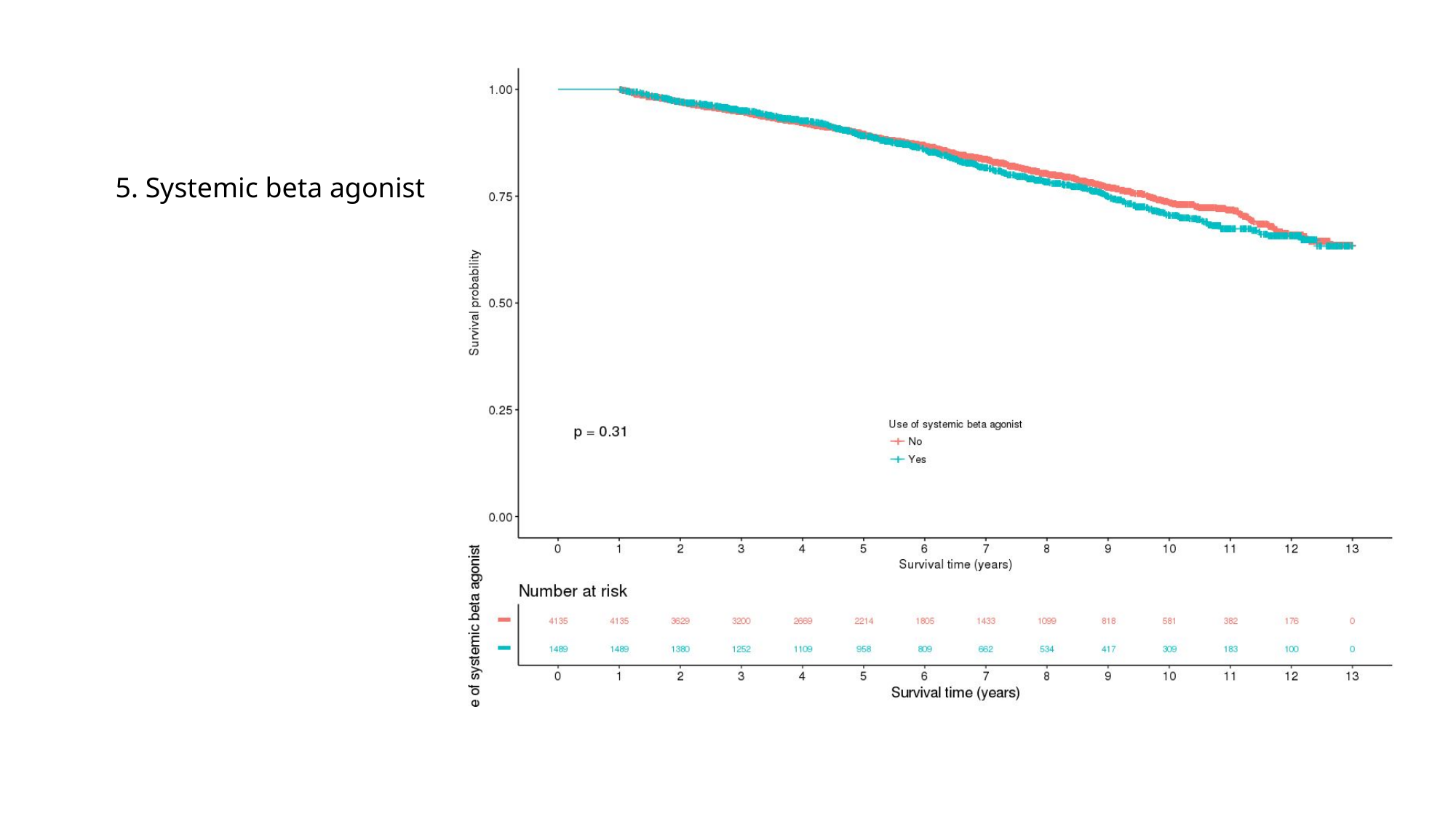

5. Systemic beta agonist

## Slide 38
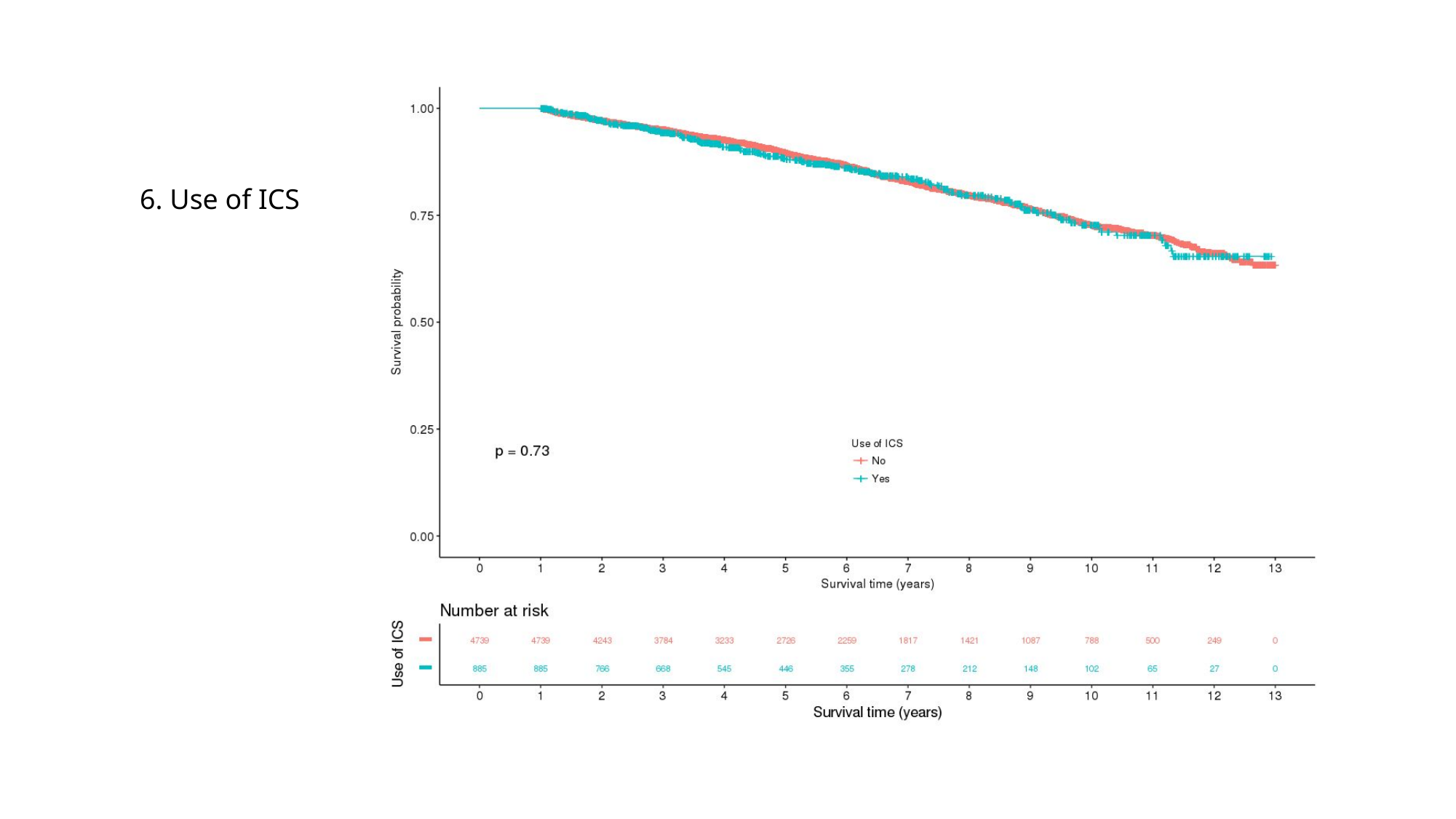

6. Use of ICS

## Slide 39
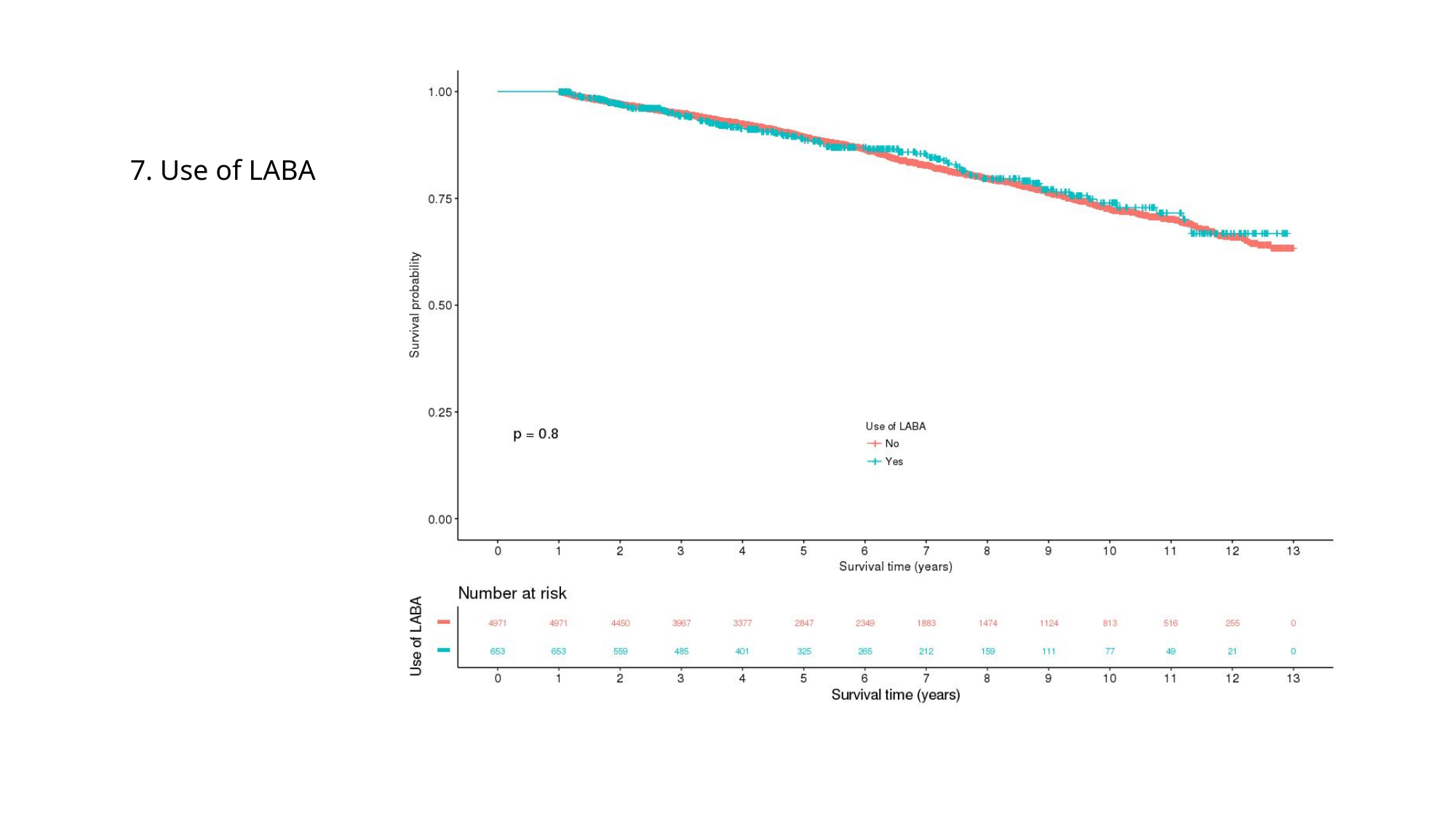

7. Use of LABA

## Slide 40
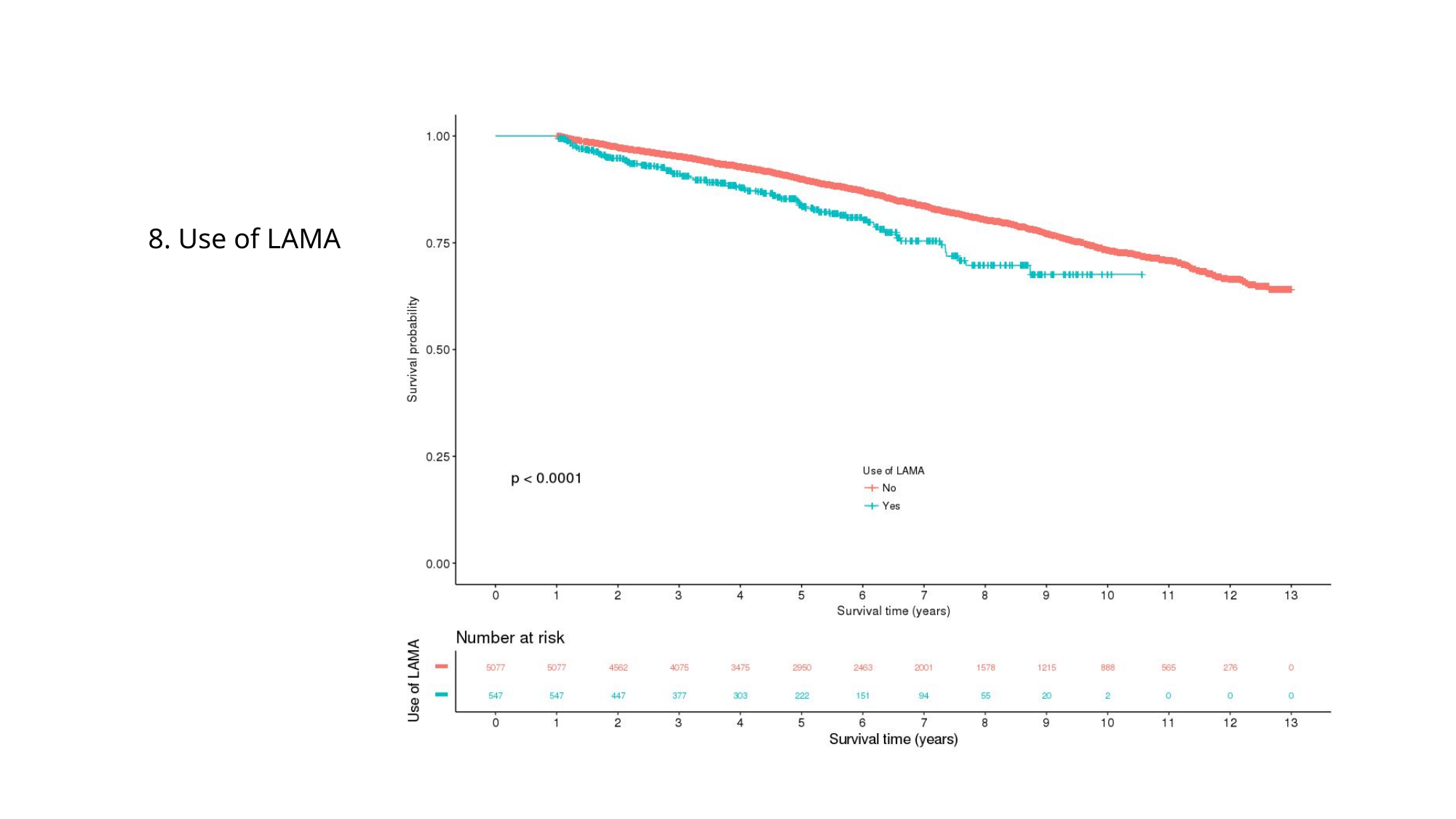

8. Use of LAMA

## Slide 41
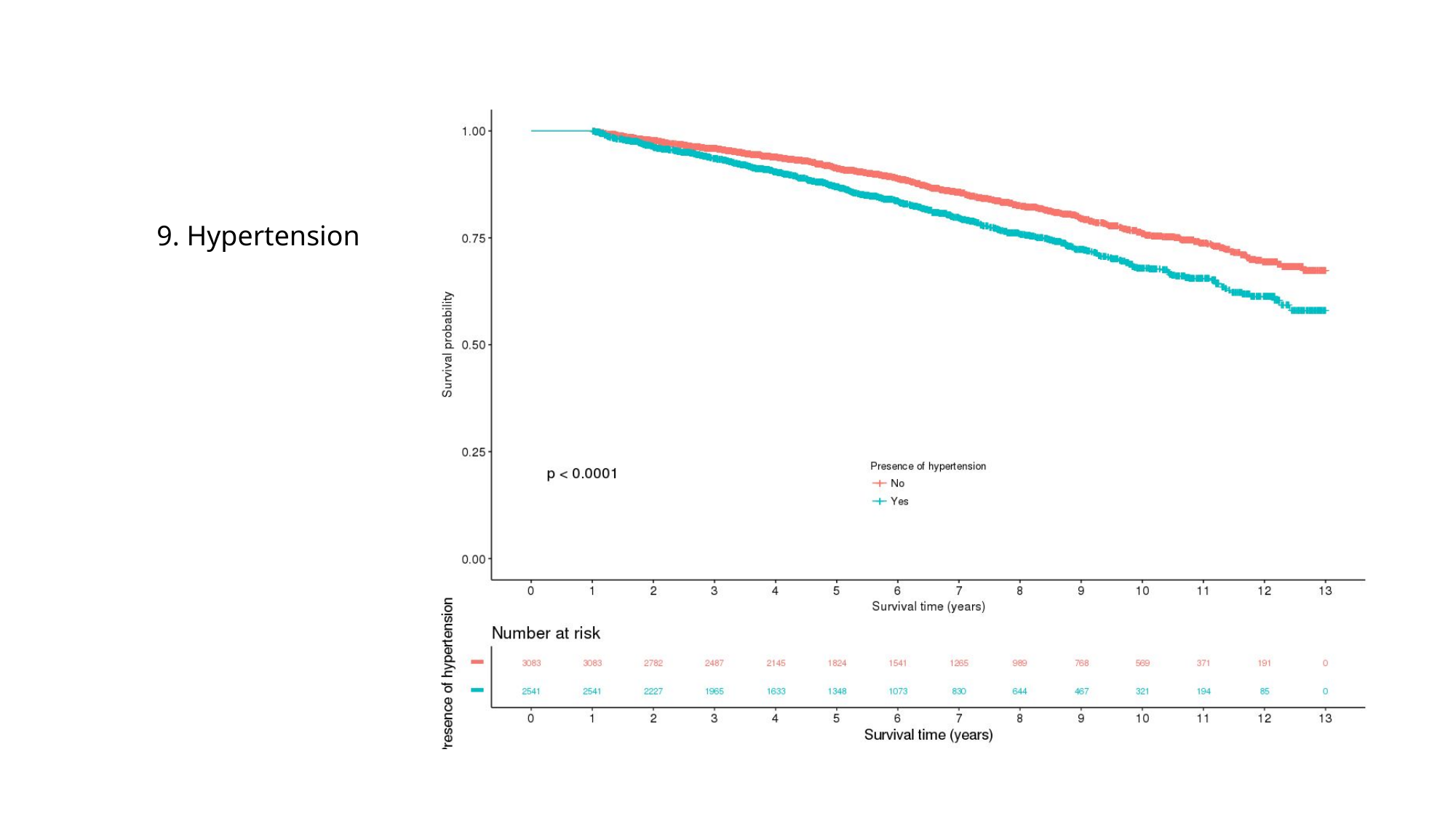

9. Hypertension

## Slide 42
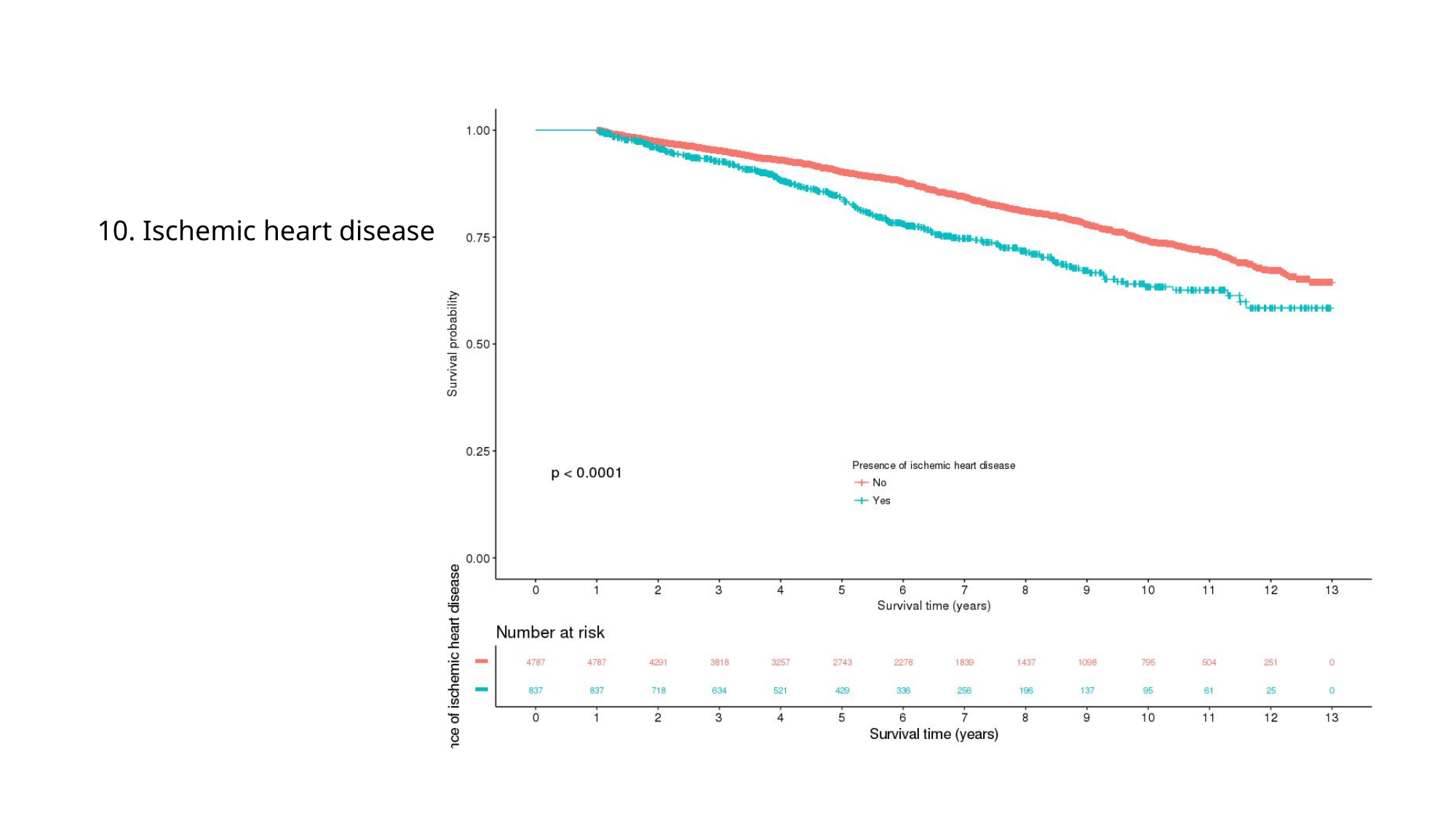

10. Ischemic heart disease

## Slide 43
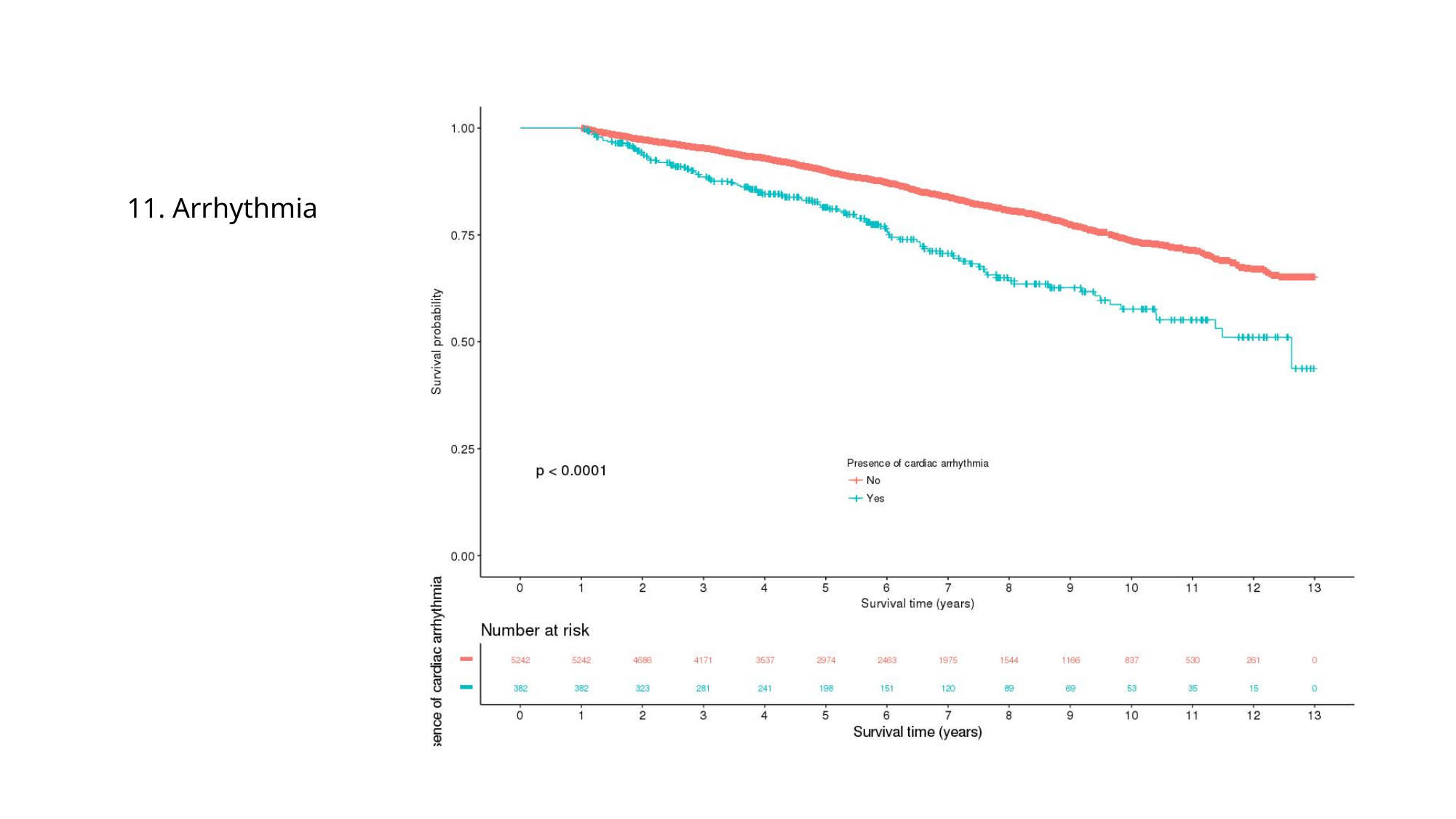

11. Arrhythmia

## Slide 44
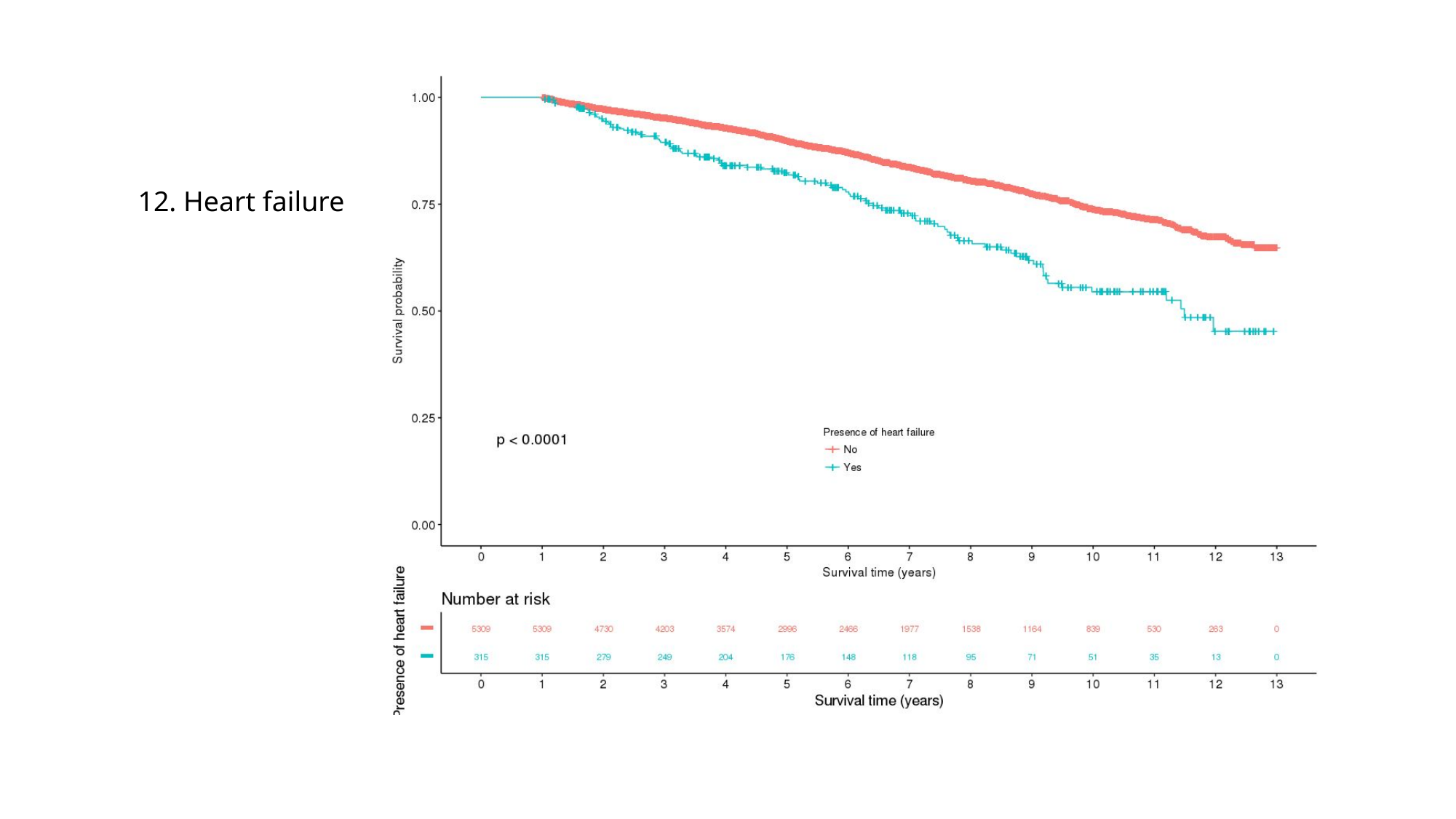

12. Heart failure

## Slide 45
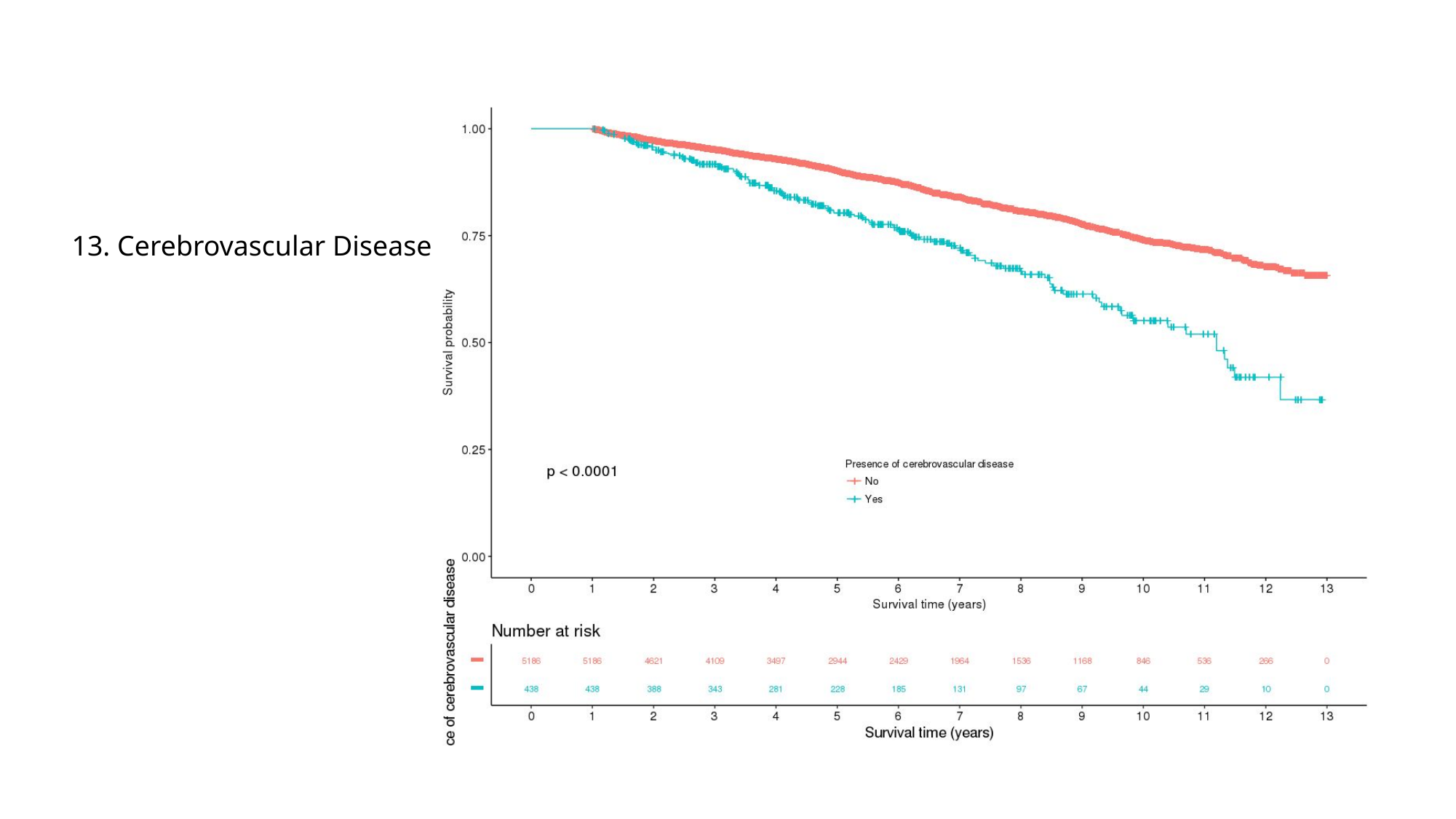

13. Cerebrovascular Disease

## Slide 46
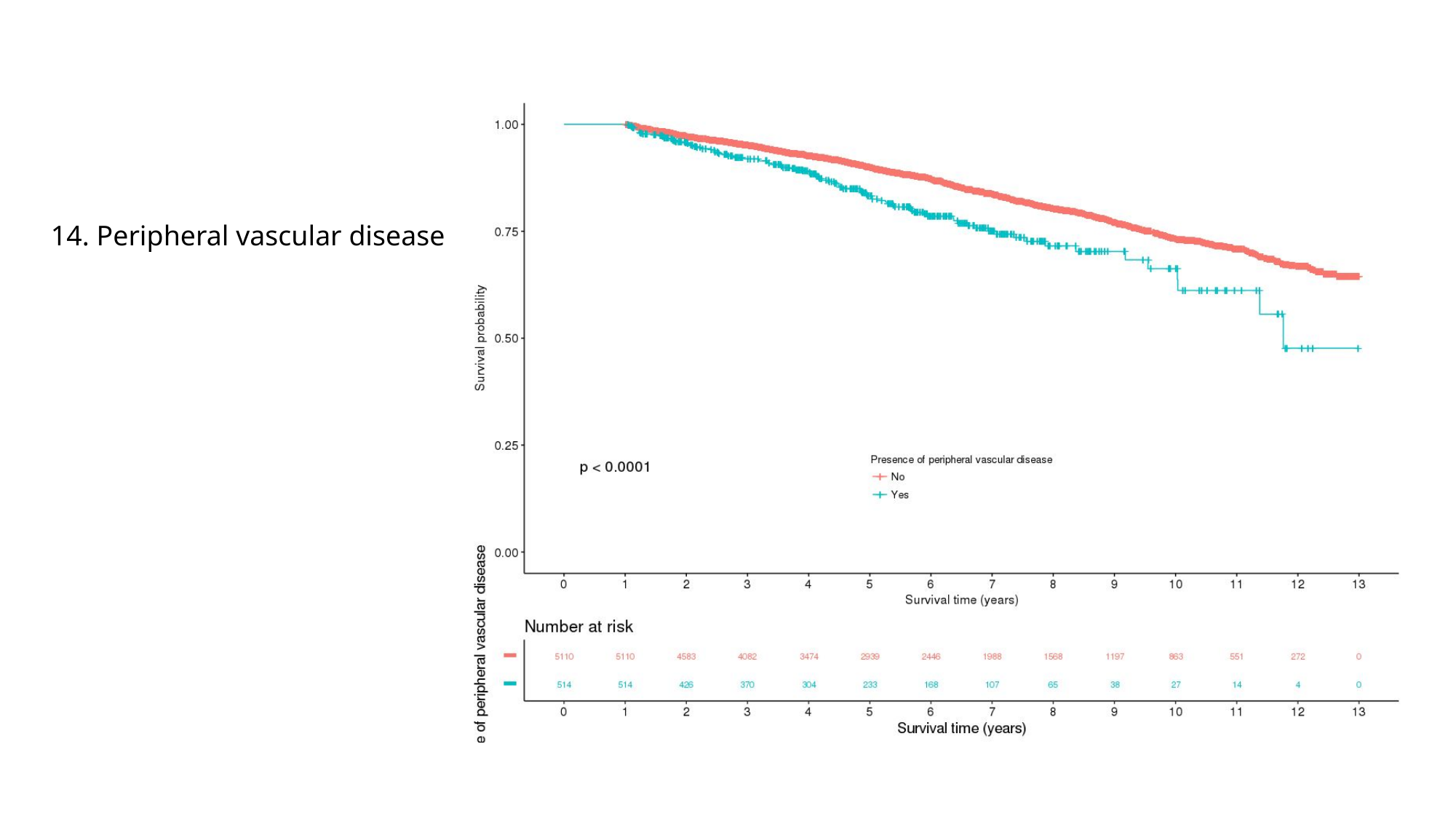

14. Peripheral vascular disease

## Slide 47
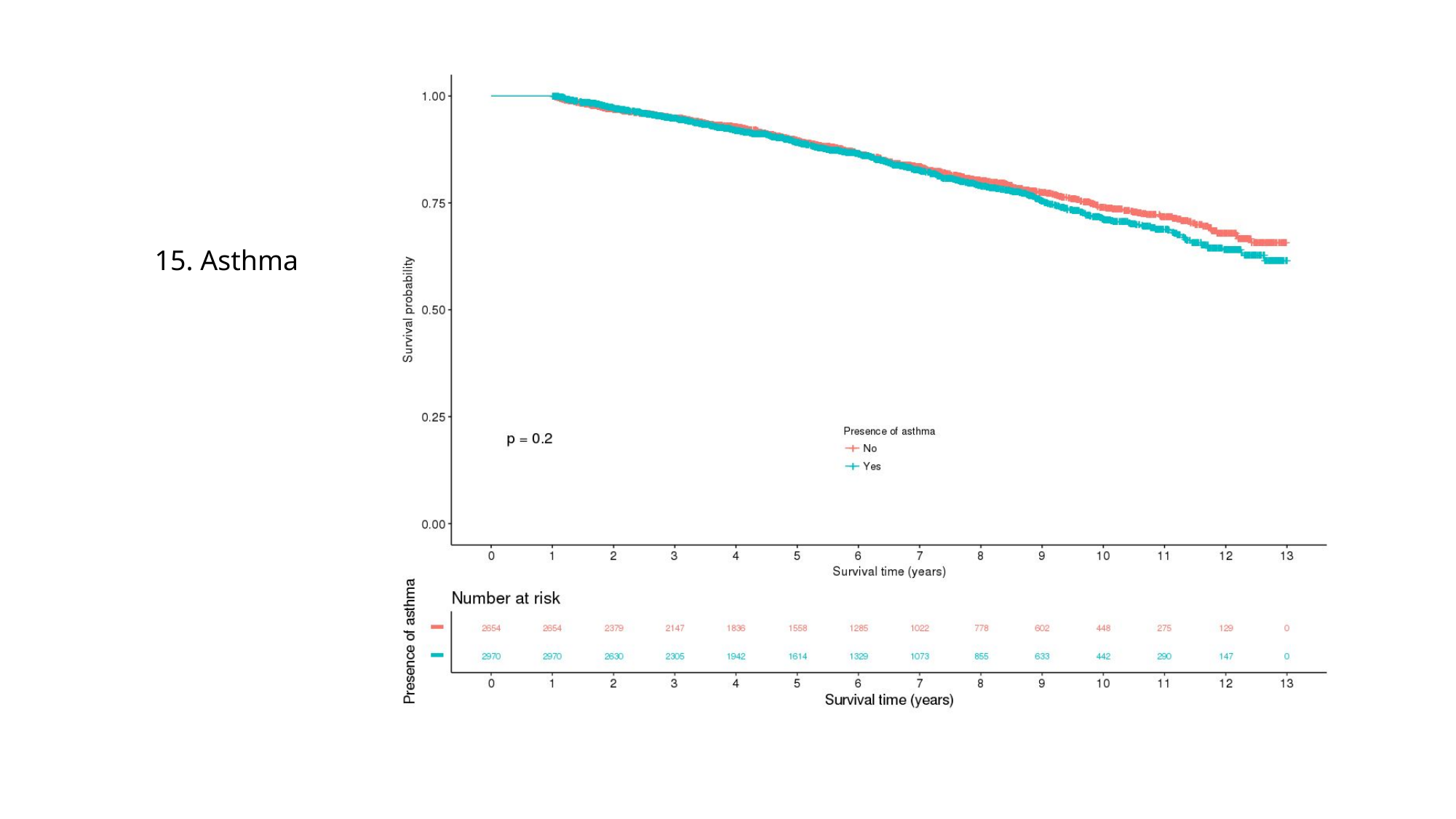

15. Asthma

## Slide 48
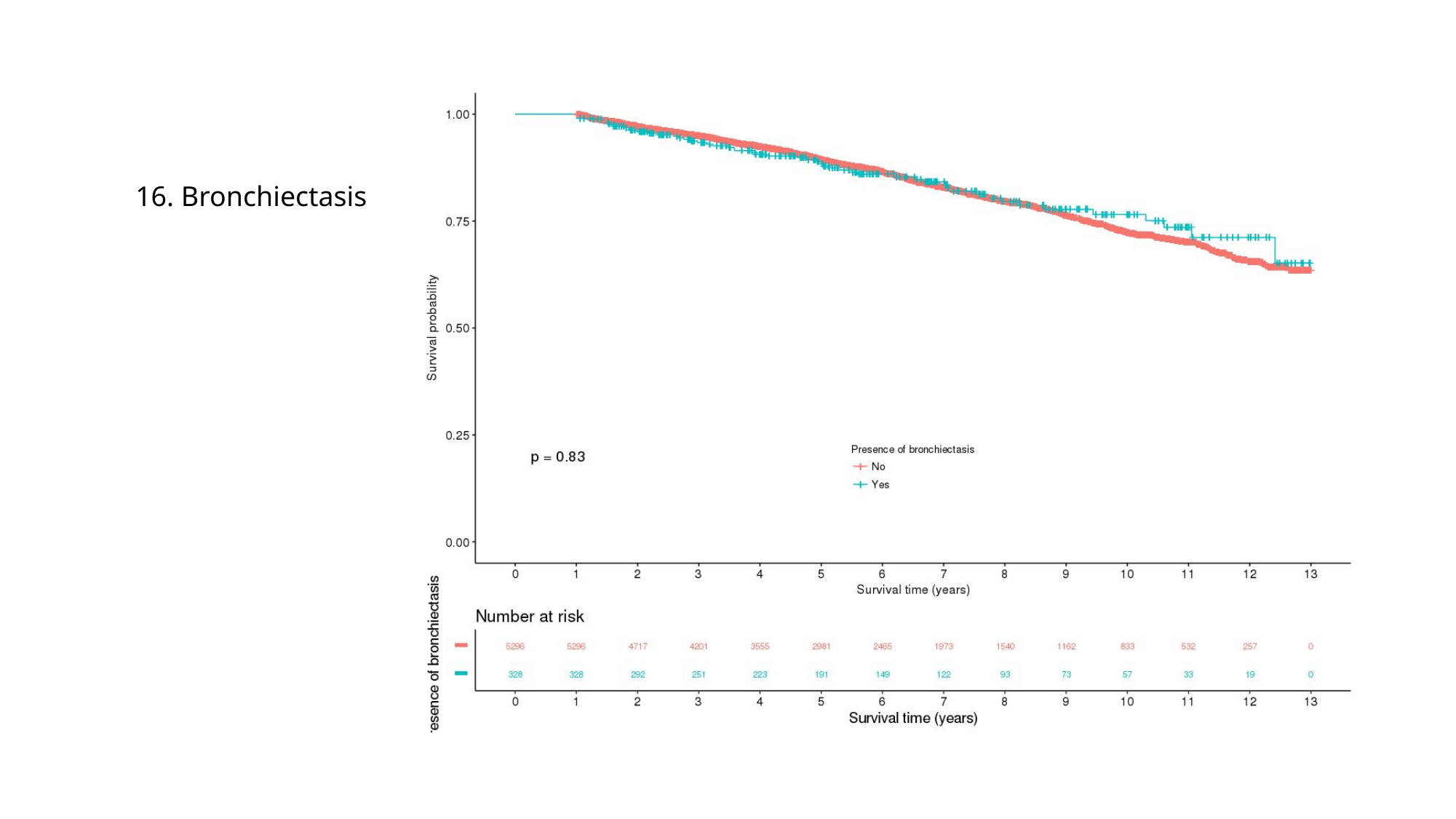

16. Bronchiectasis

## Slide 49
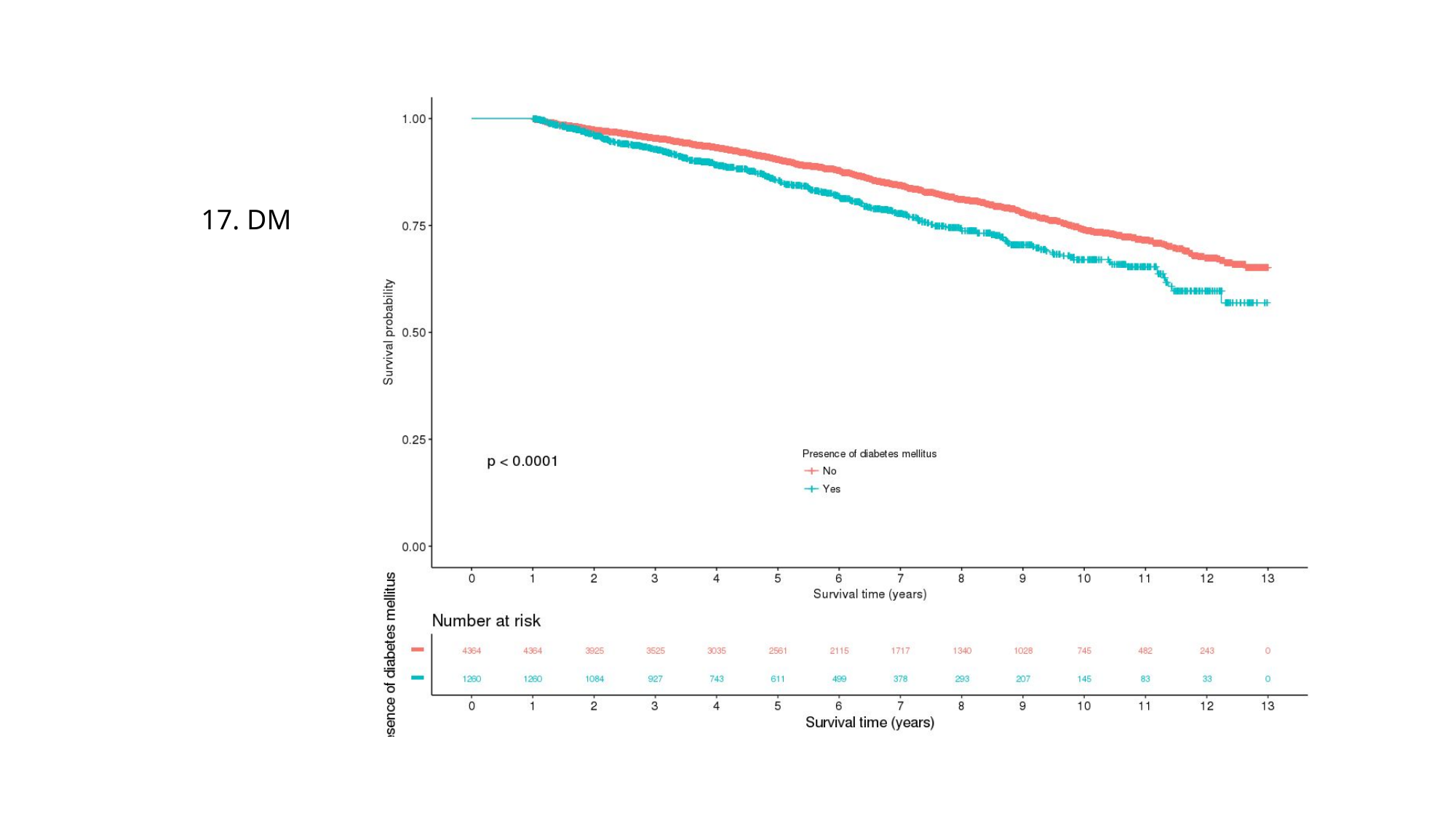

17. DM

## Slide 50
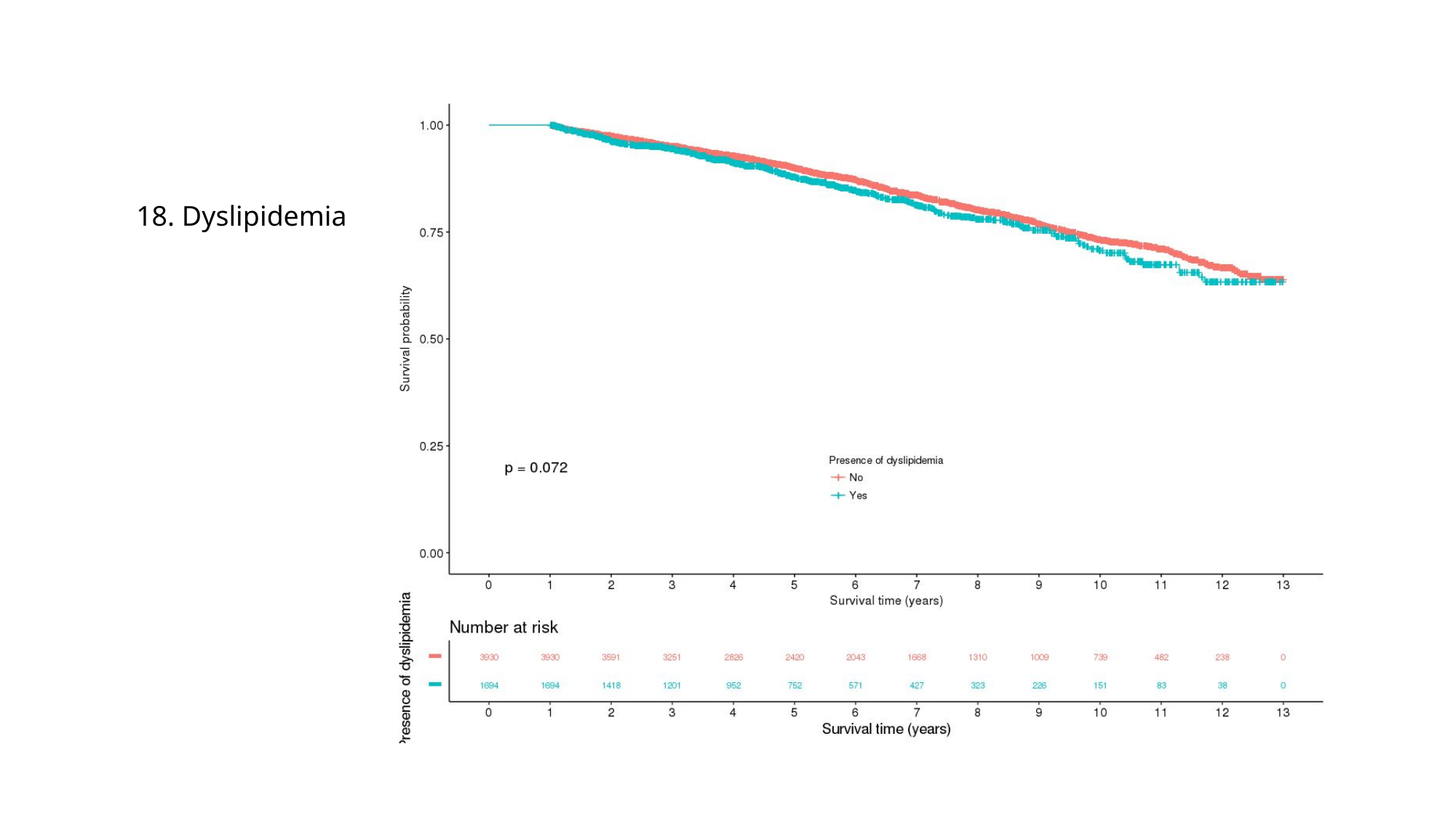

18. Dyslipidemia

## Slide 51
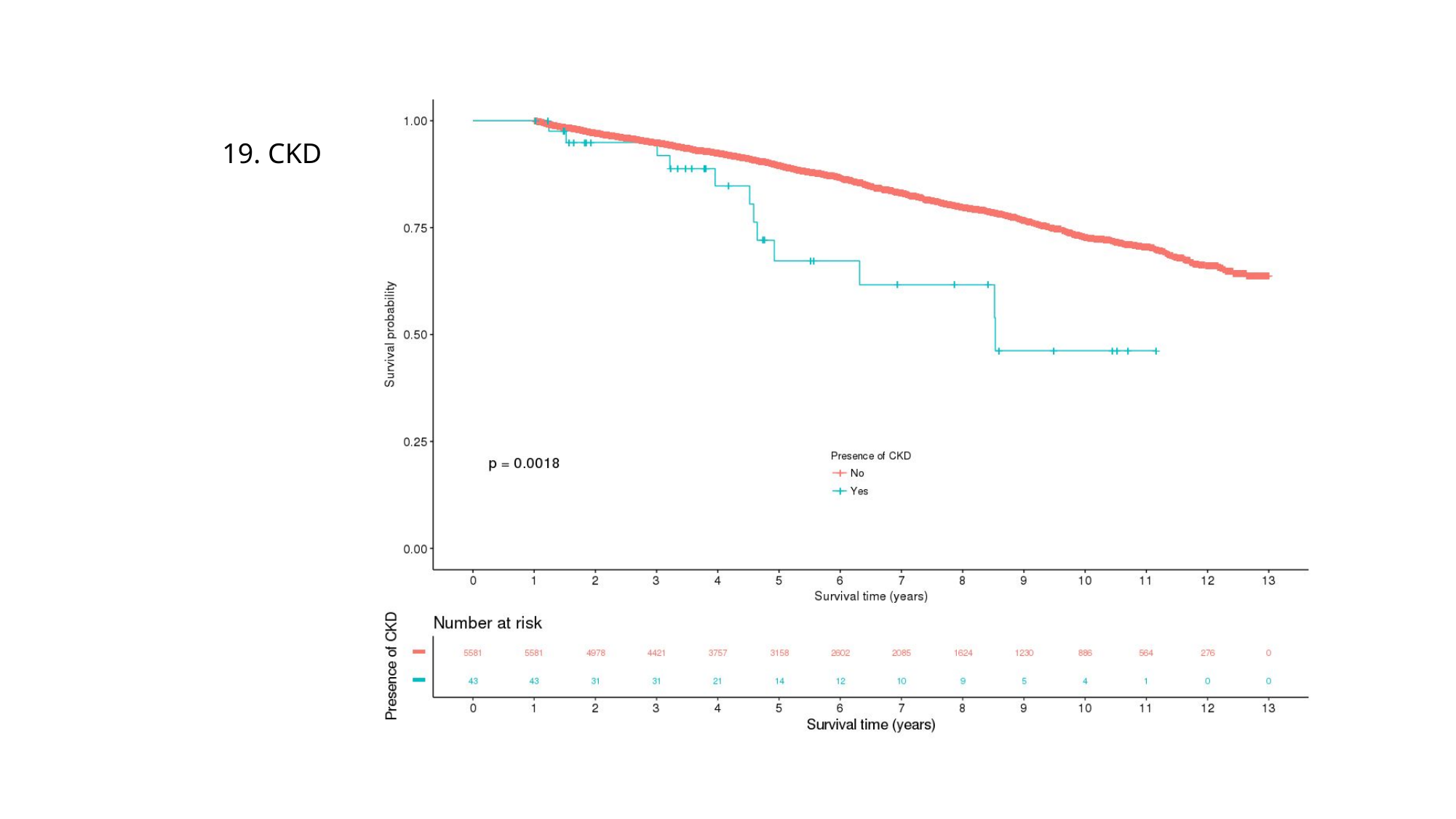

19. CKD

## Slide 52
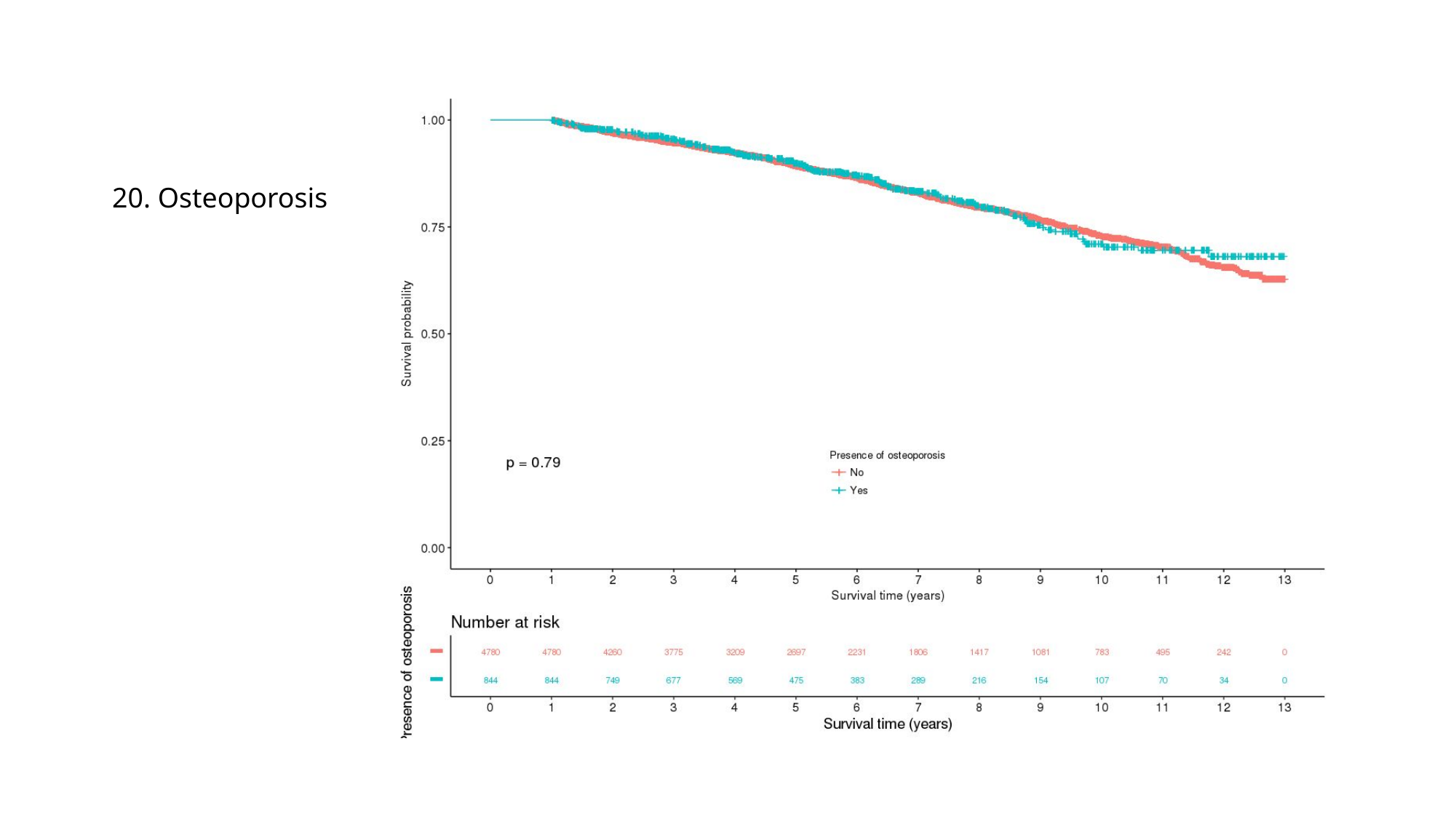

20. Osteoporosis

## Slide 53
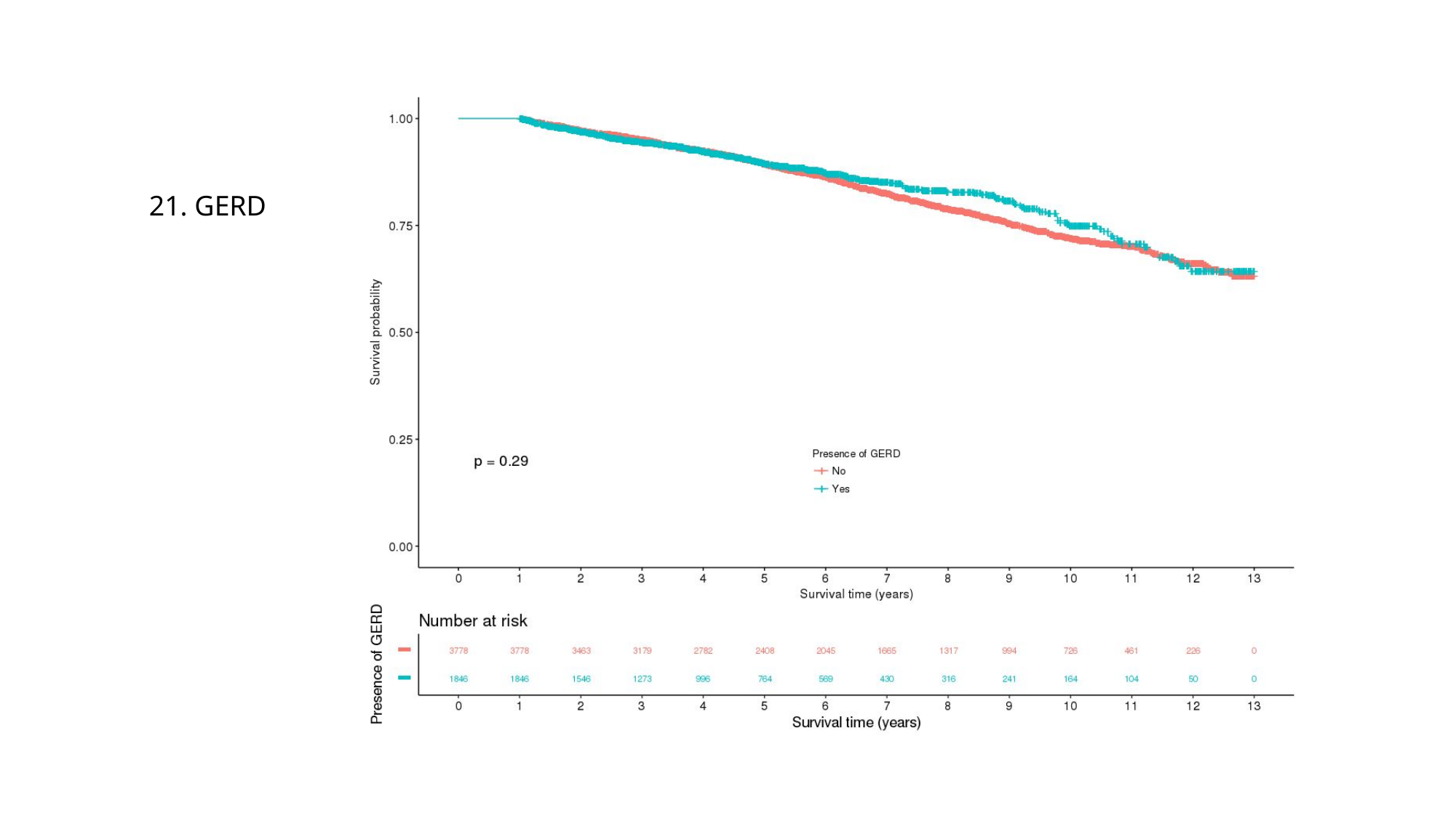

21. GERD

## Slide 54
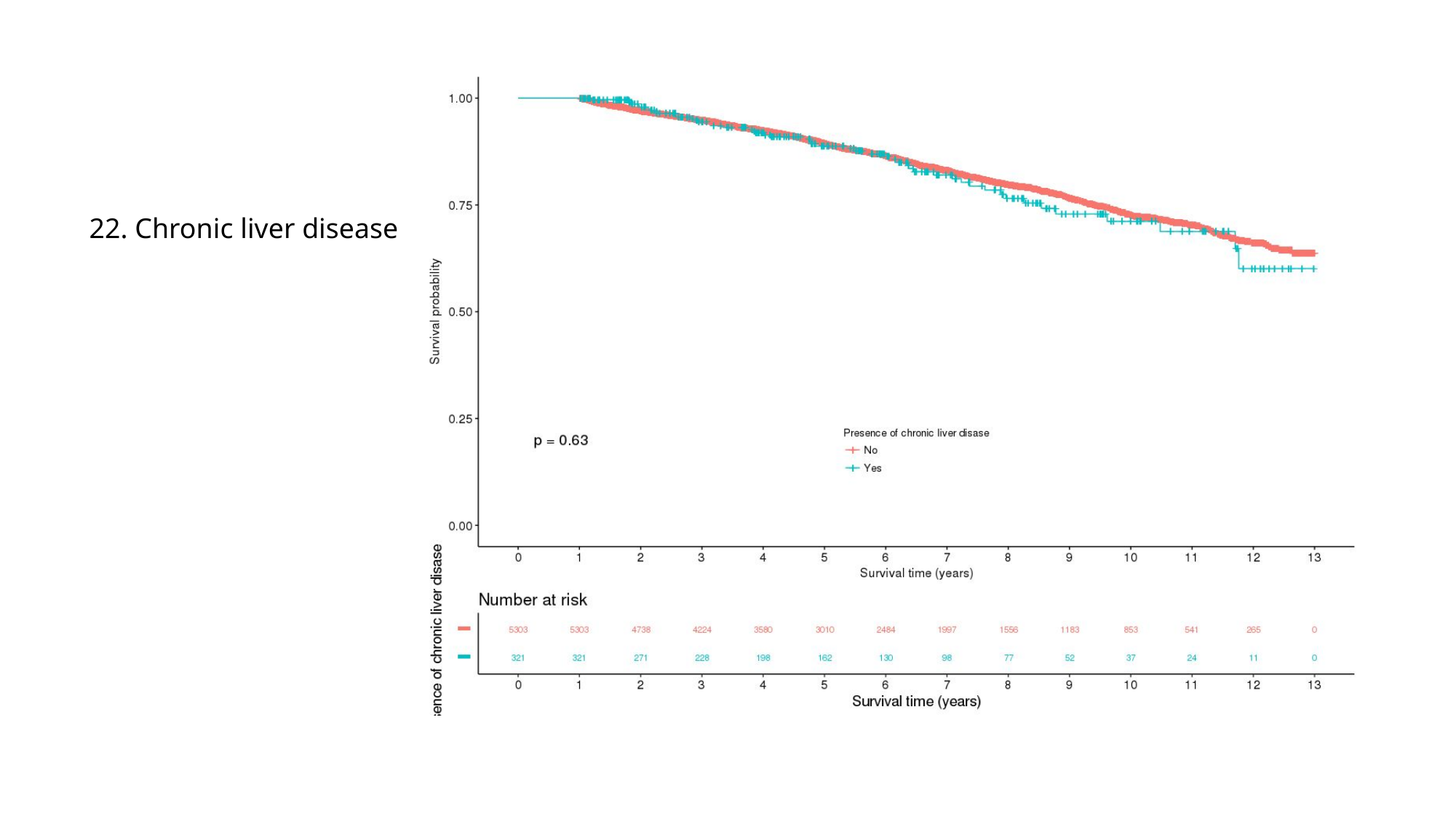

22. Chronic liver disease

## Slide 55
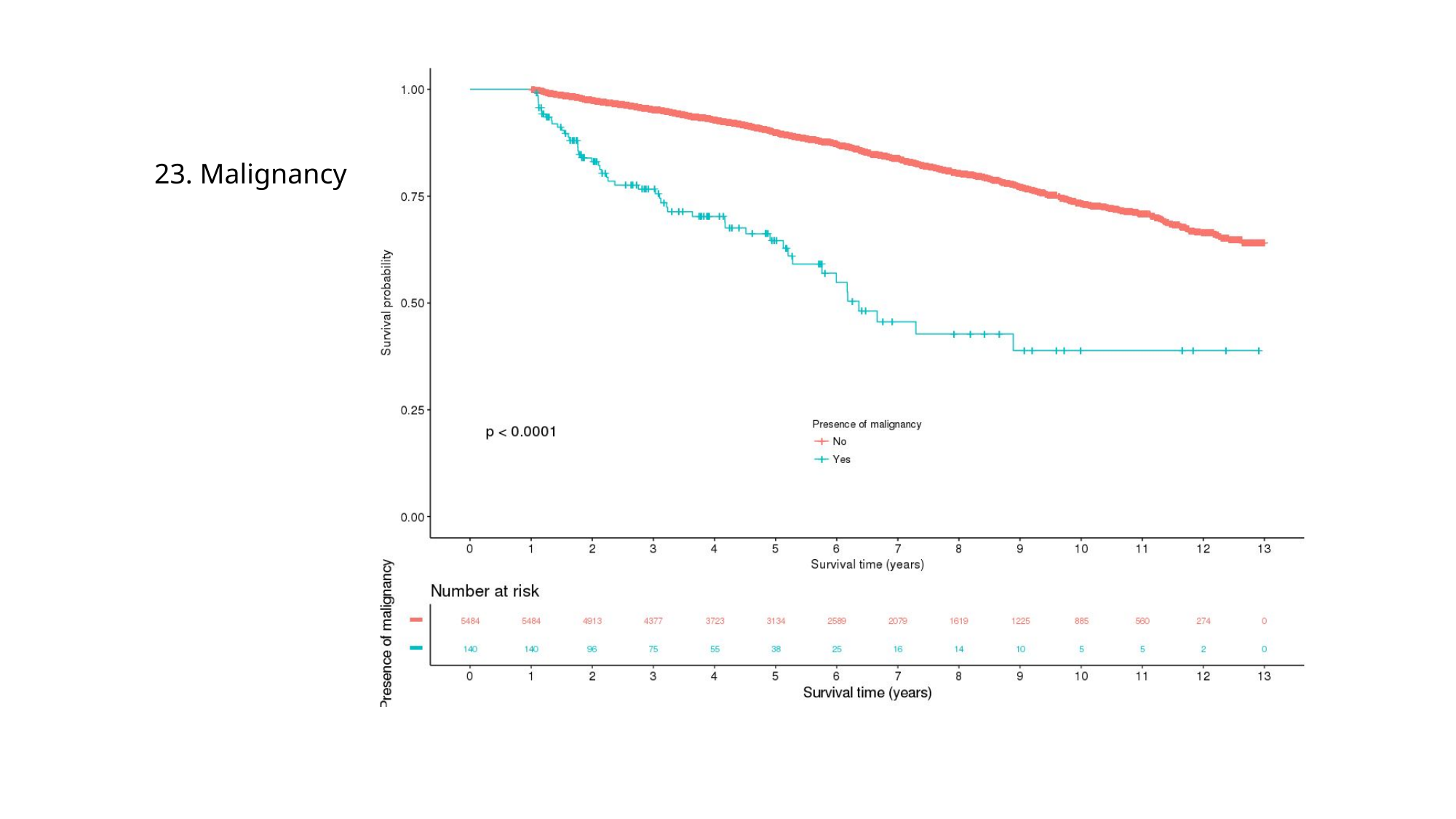

23. Malignancy

## Slide 56
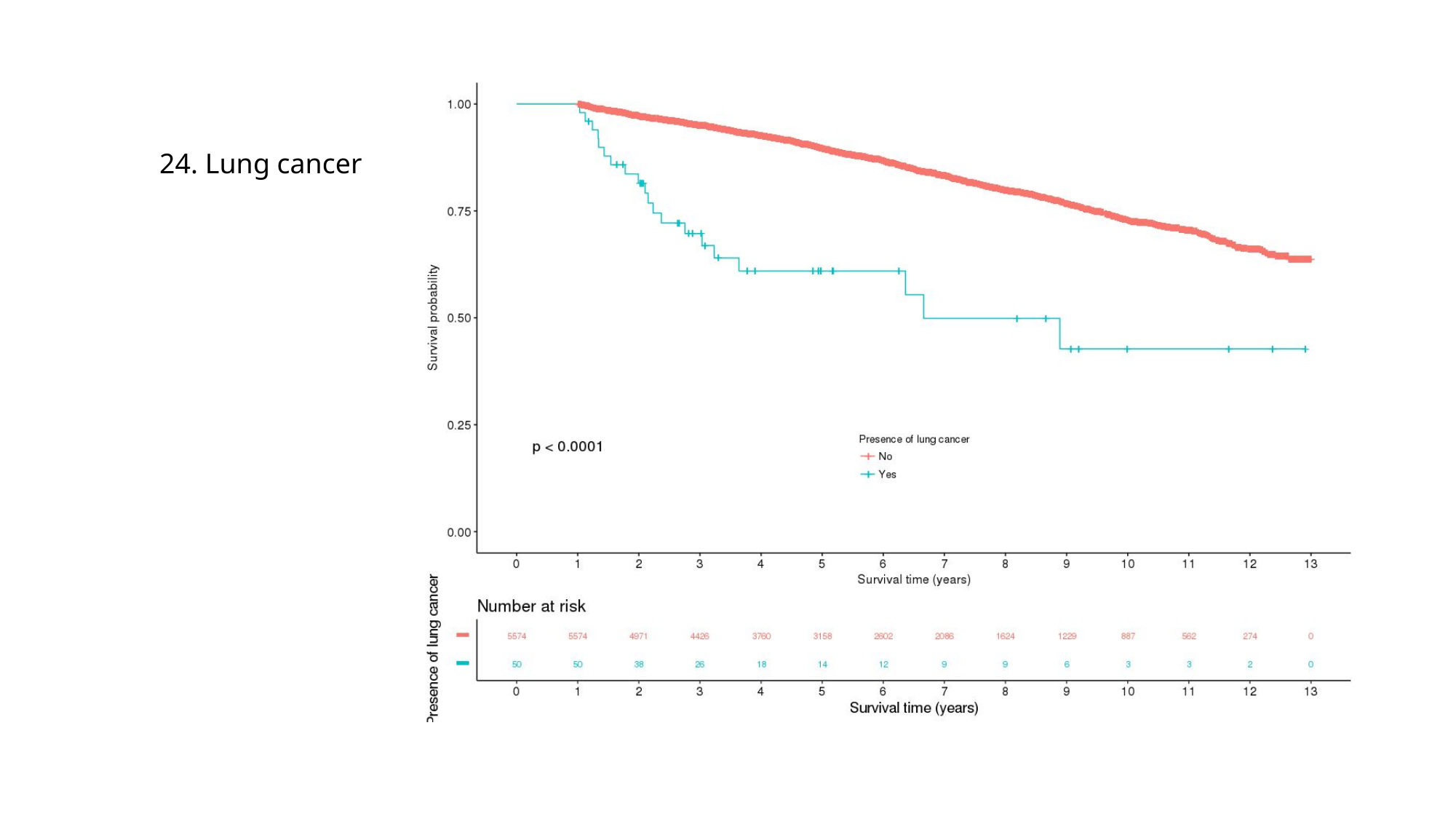

24. Lung cancer

## Slide 57
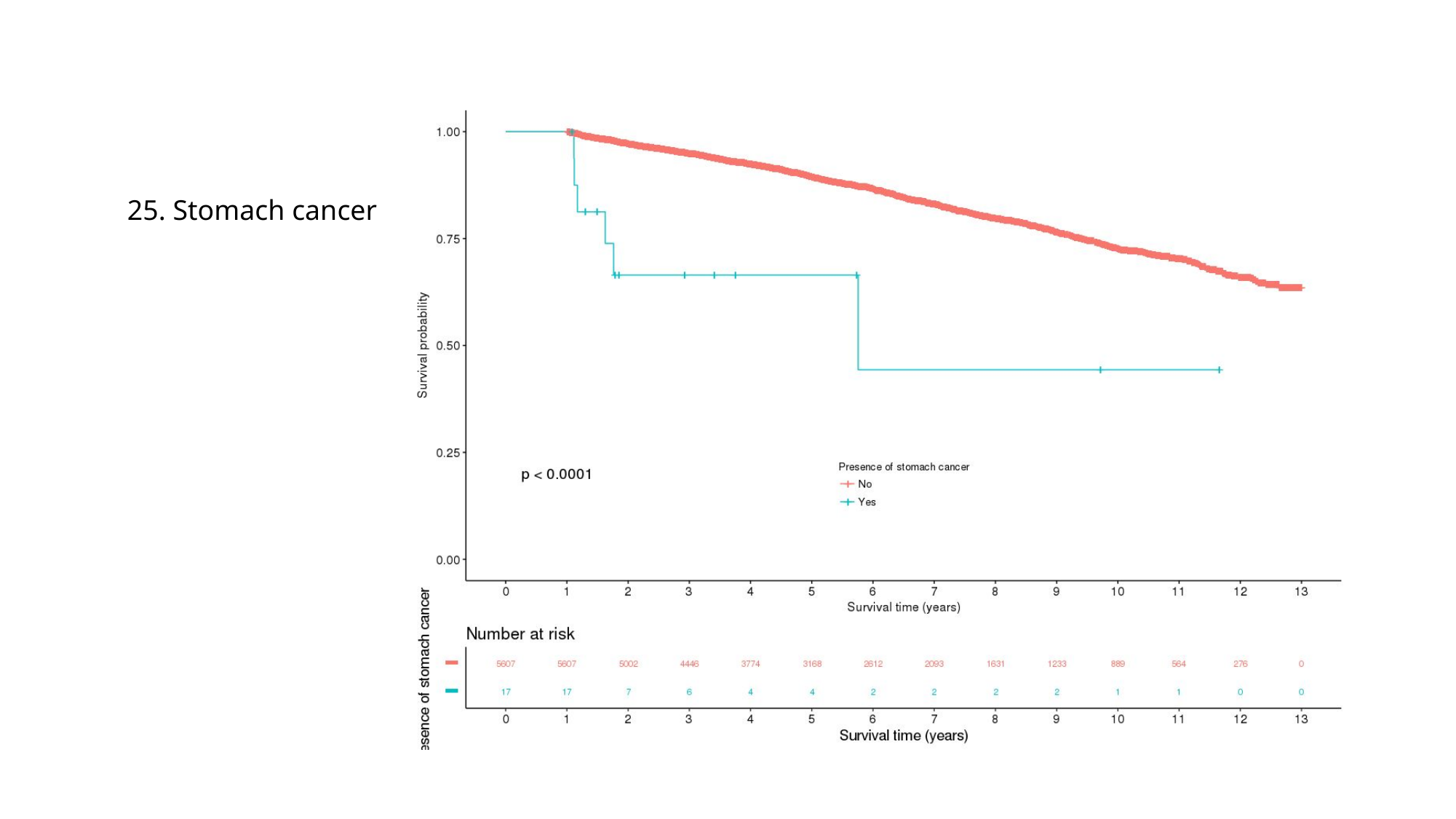

25. Stomach cancer

## Slide 58
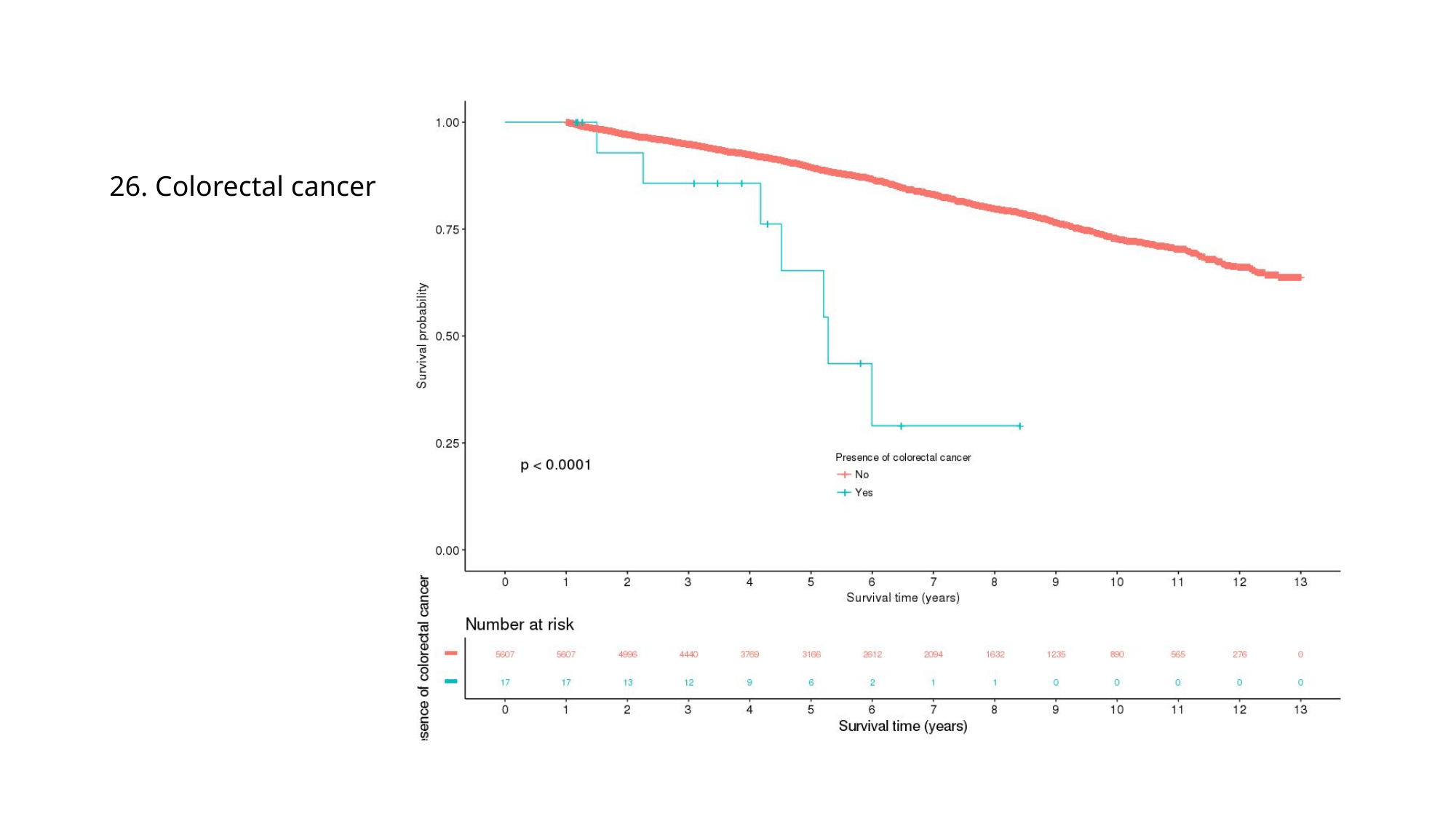

26. Colorectal cancer

## Slide 59
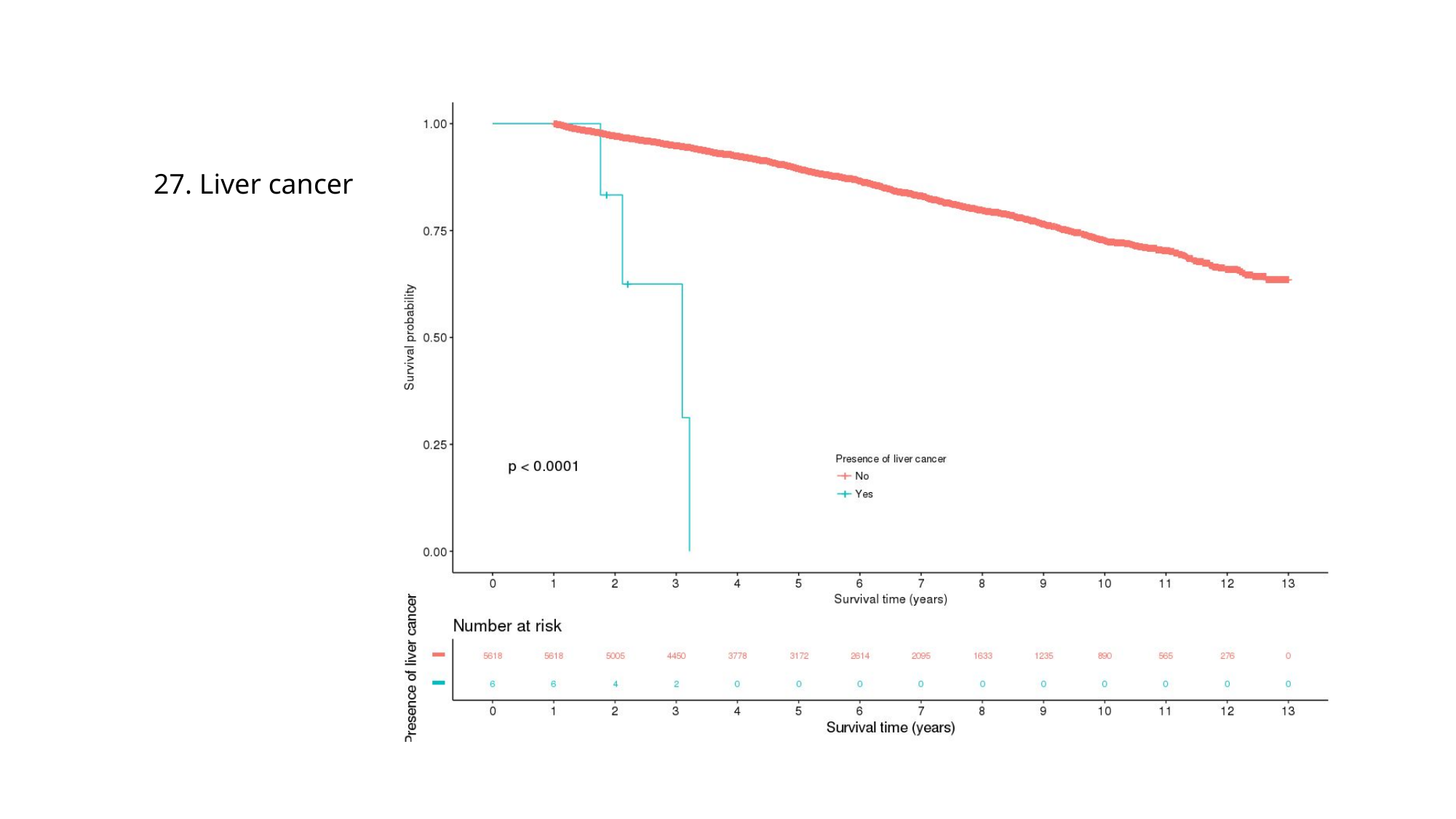

27. Liver cancer

## Slide 60
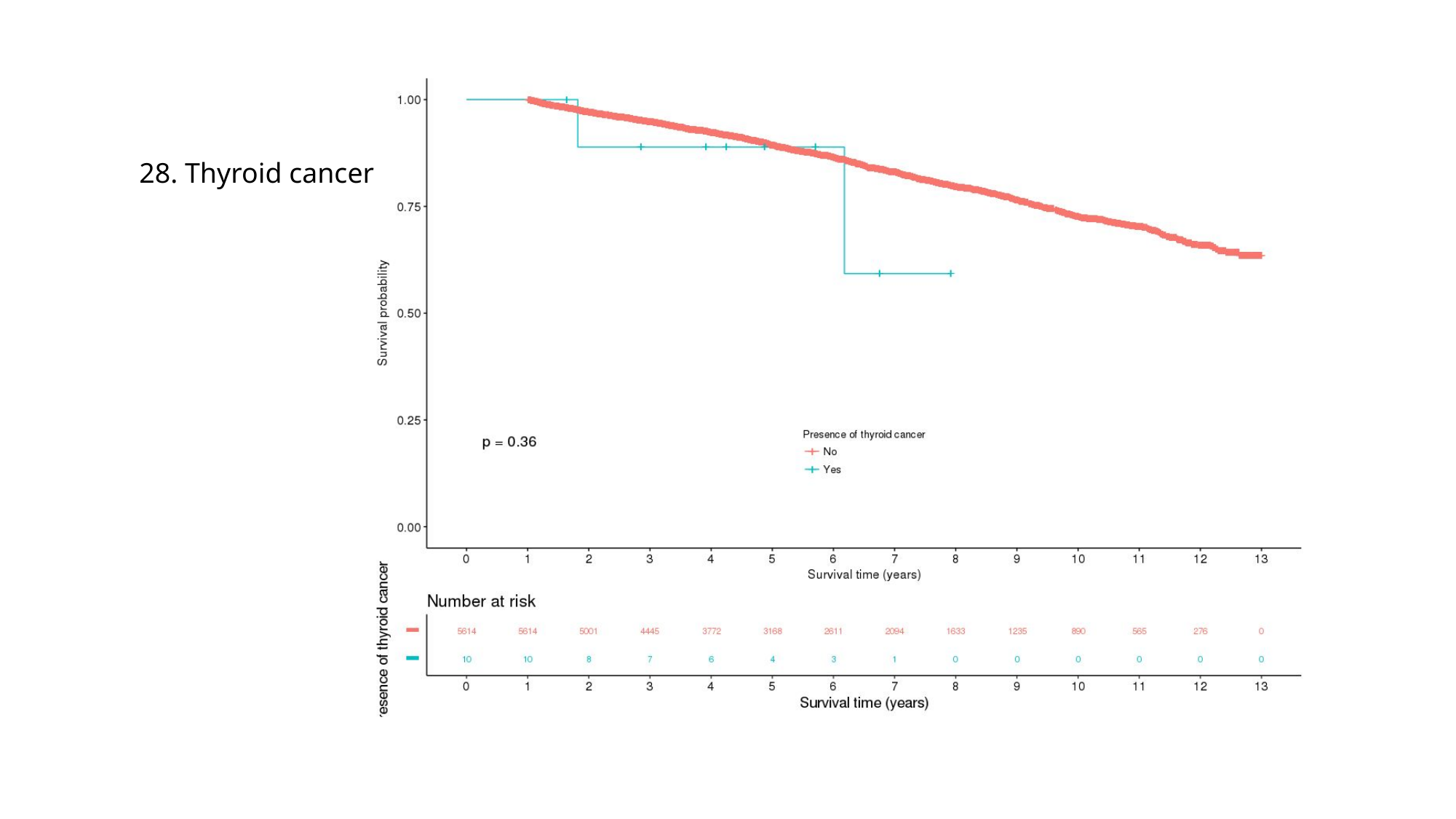

28. Thyroid cancer

## Slide 61
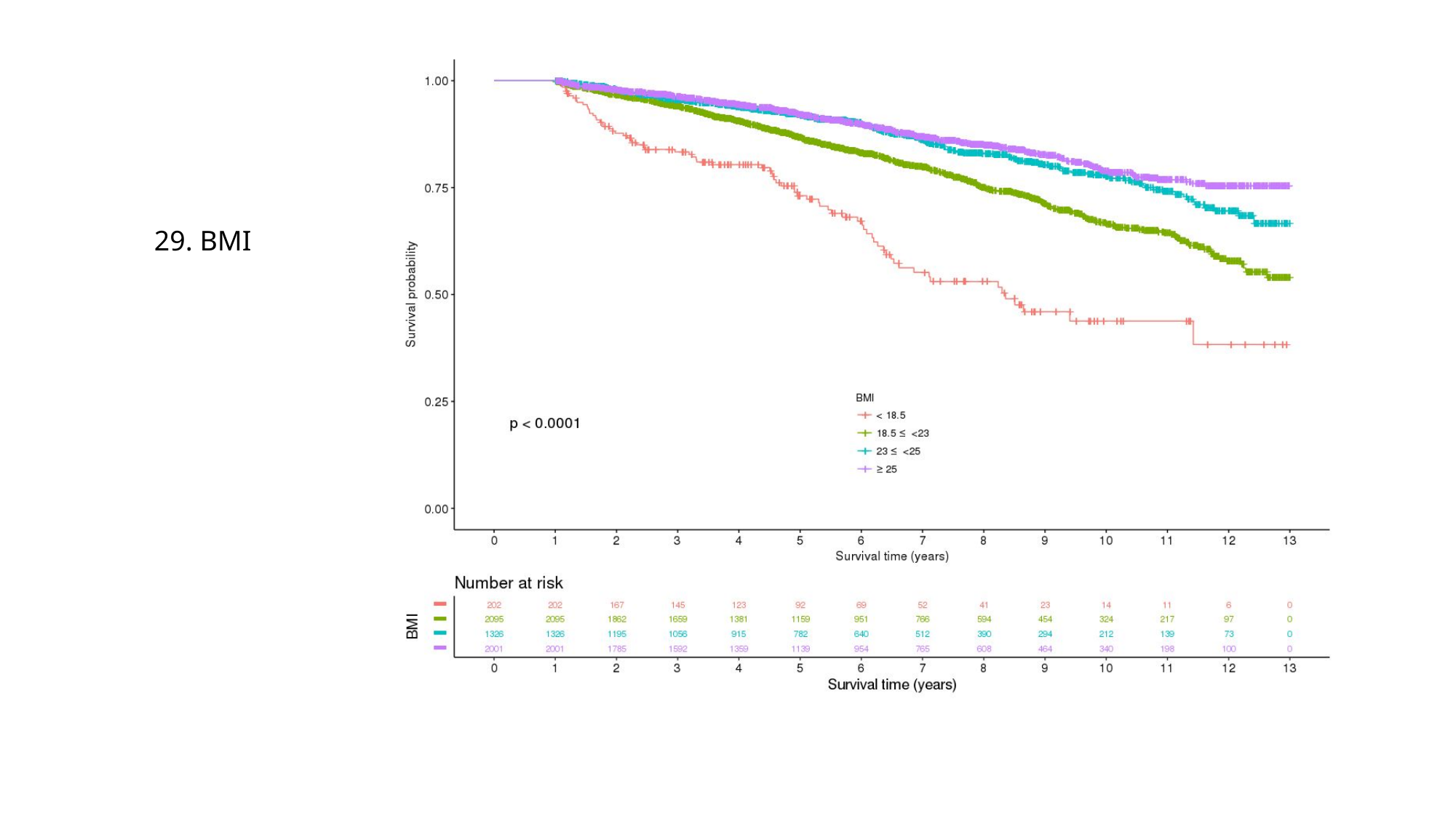

29. BMI

## Slide 62
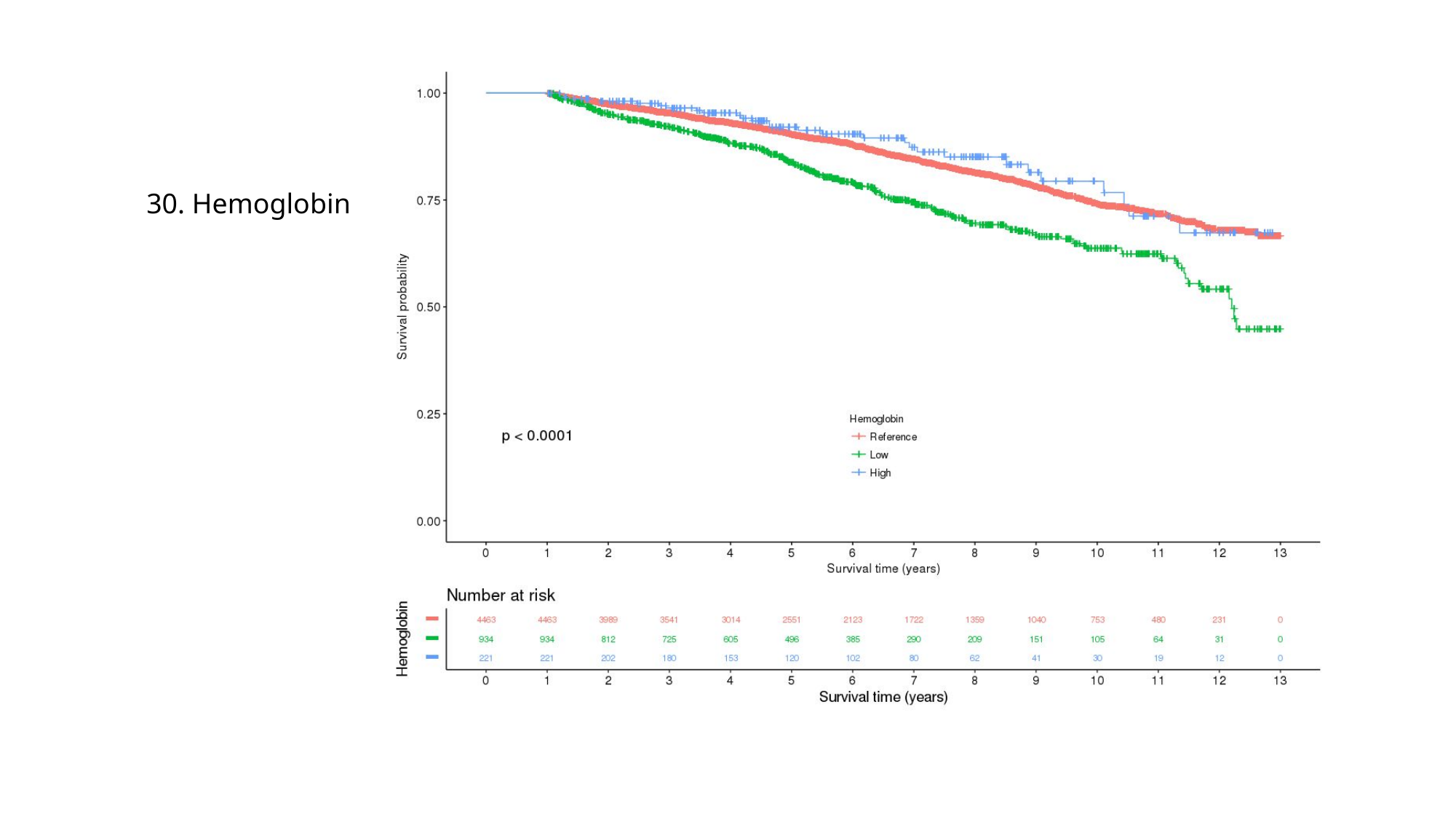

30. Hemoglobin

## Slide 63
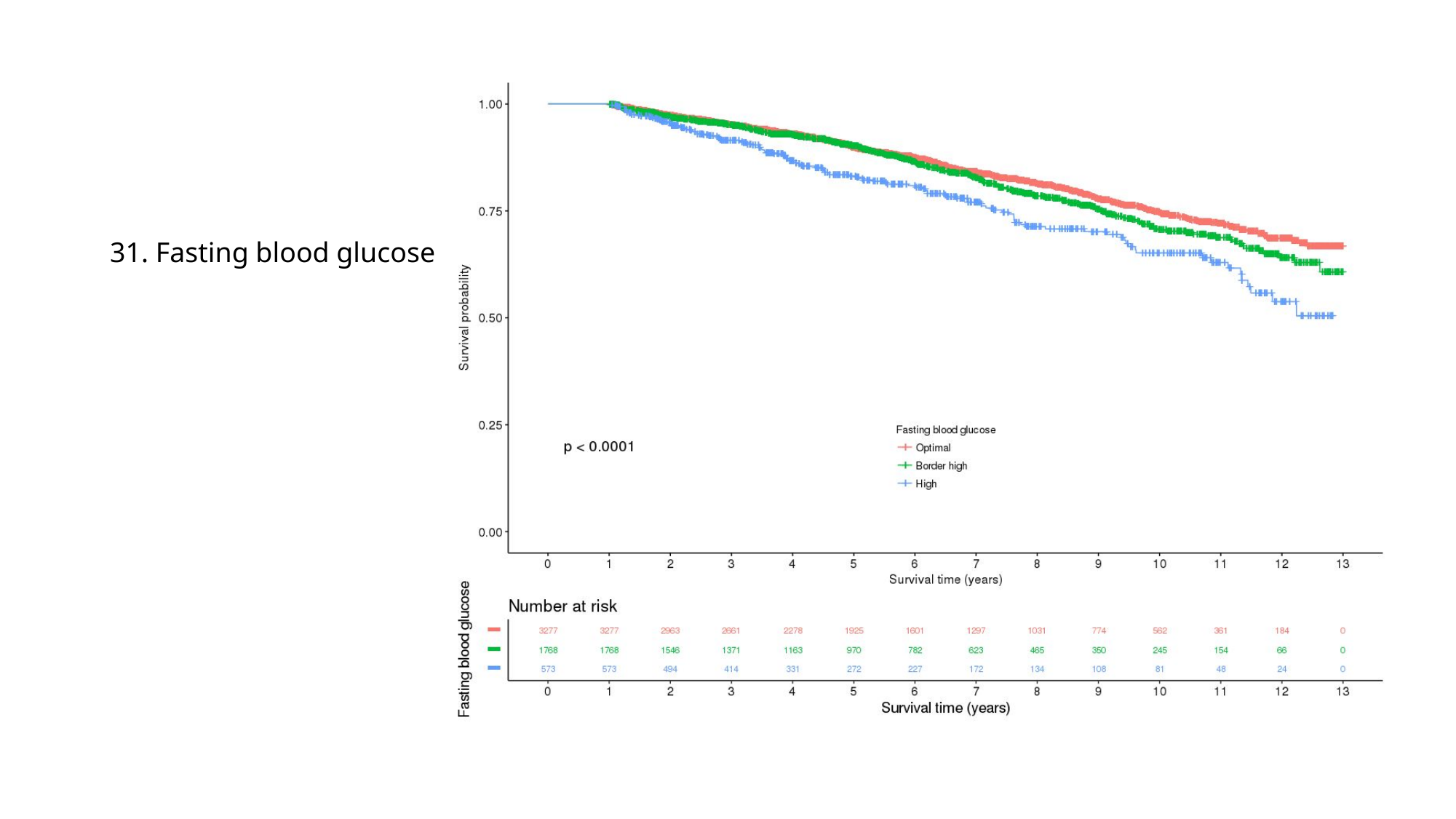

31. Fasting blood glucose

## Slide 64
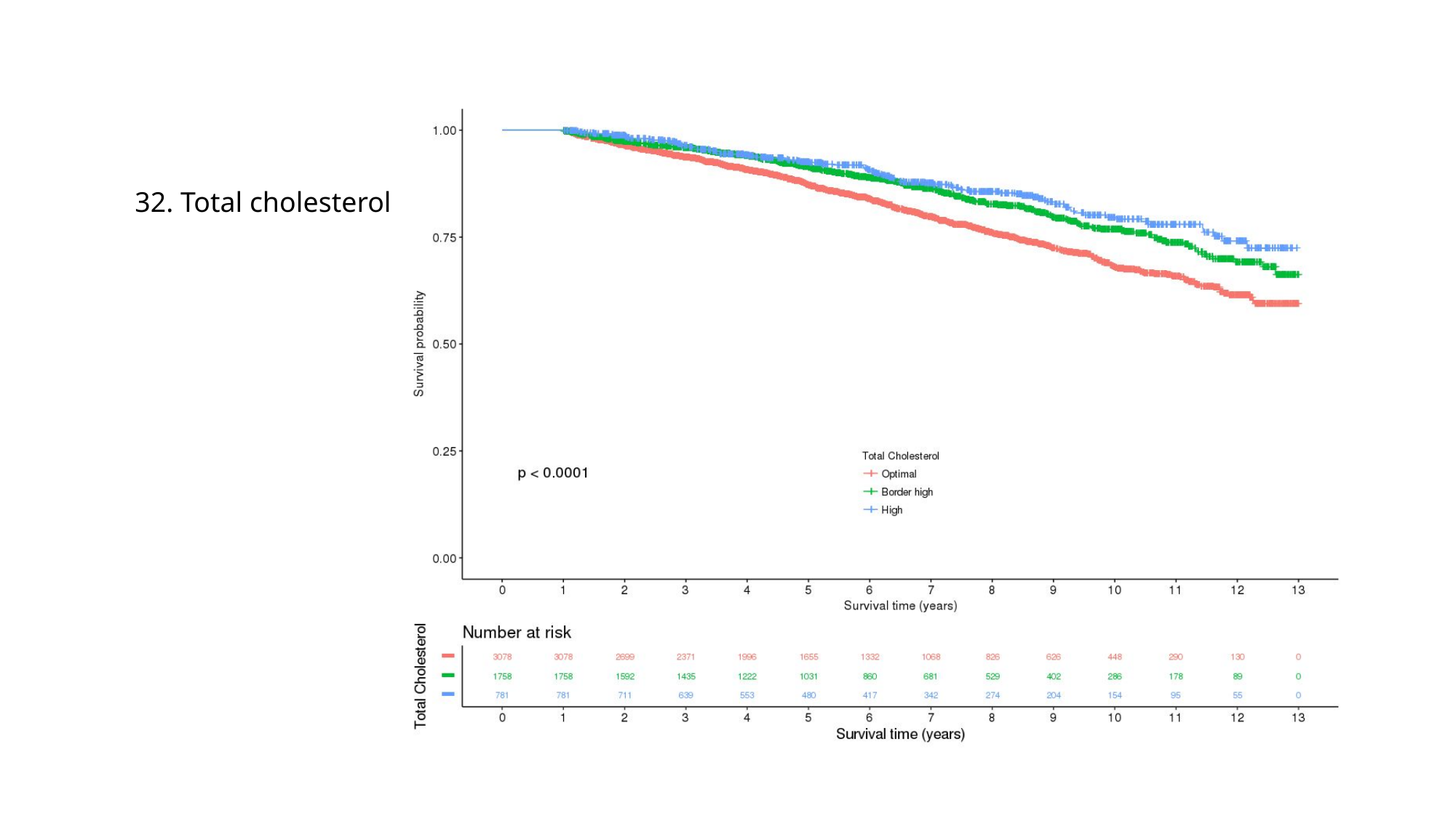

32. Total cholesterol

## Slide 65
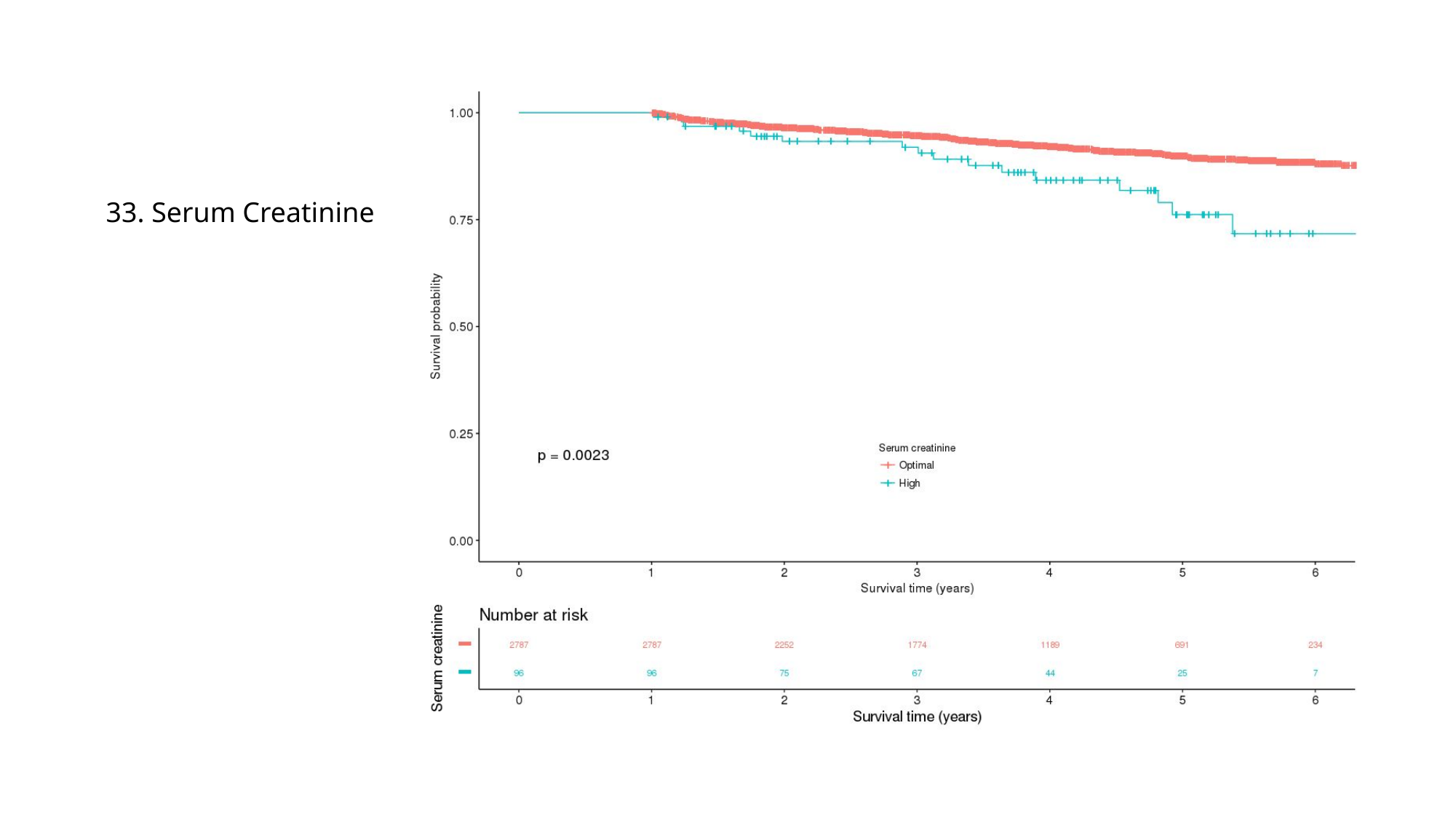

33. Serum Creatinine

## Slide 66
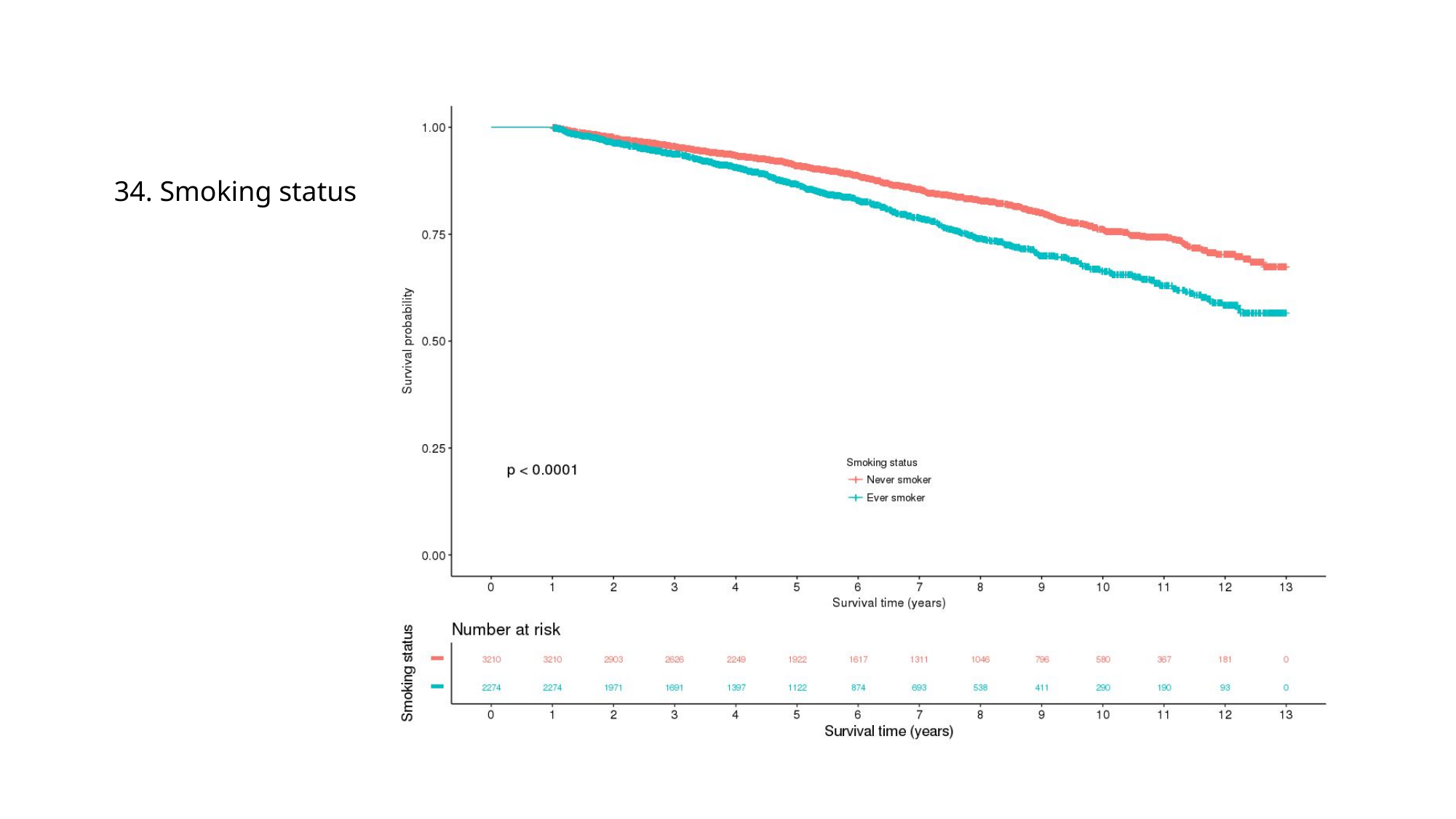

34. Smoking status
