# Supplementary material for: Effect of multiple comorbidities on mortality in chronic obstructive pulmonary disease among Korean population: a nationwide cohort study
Source: BMC Pulm Med. 2021 Feb 11;21:56. doi: 10.1186/s12890-021-01424-7 (PMC7879613; doi:10.1186/s12890-021-01424-7)
Supplement: Supplementary file 3 — Additional file 3: Fig. S2. Kaplan-Meier curves comparing respiratory mortalities in entire cohort according to comorbidities and clinical variables. [file 12890_2021_1424_MOESM3_ESM.pptx]

## Slide 1
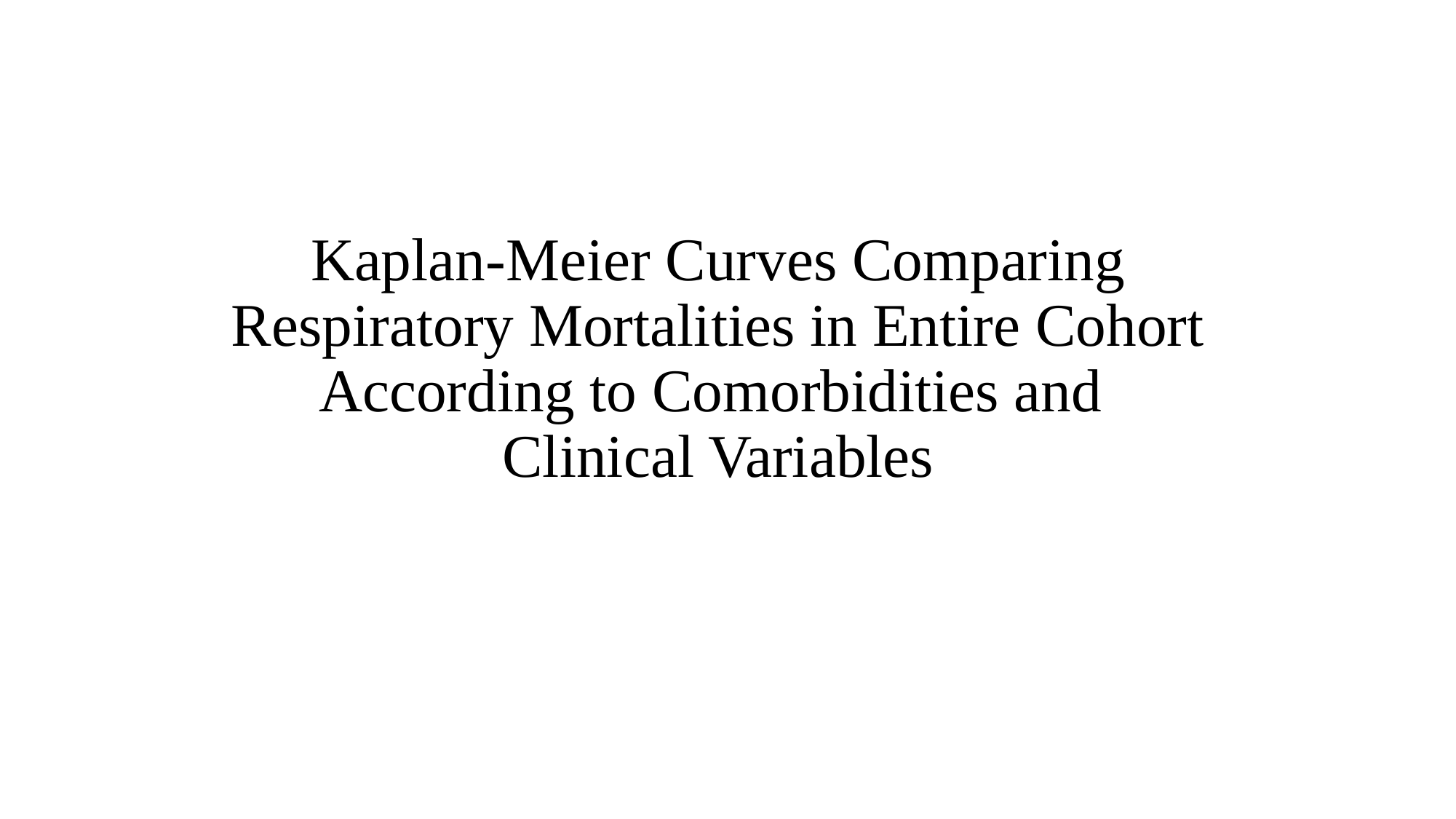

# Kaplan-Meier Curves Comparing Respiratory Mortalities in Entire Cohort According to Comorbidities and Clinical Variables

## Slide 2
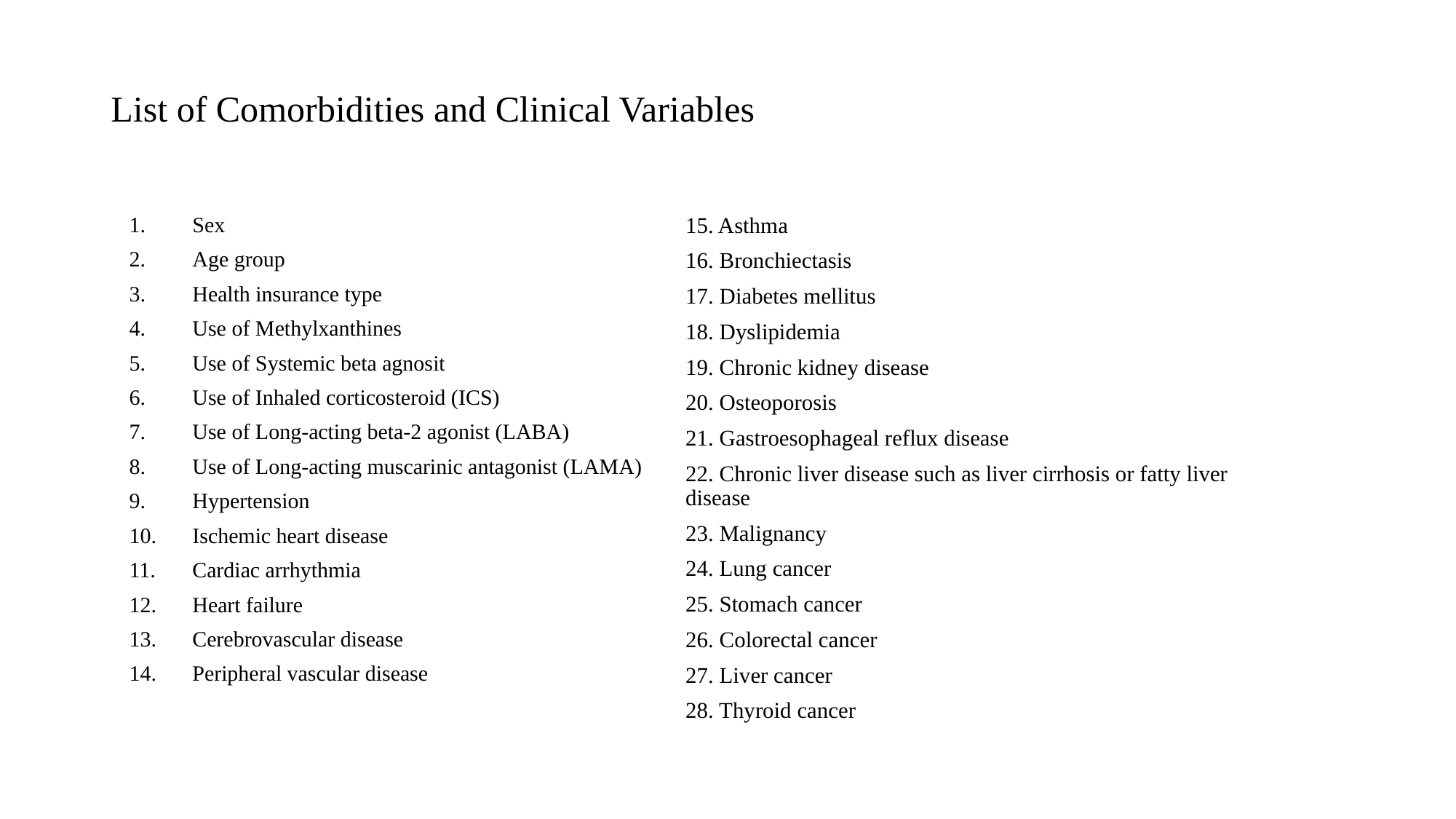

# List of Comorbidities and Clinical Variables
Sex
Age group
Health insurance type
Use of Methylxanthines
Use of Systemic beta agnosit
Use of Inhaled corticosteroid (ICS)
Use of Long-acting beta-2 agonist (LABA)
Use of Long-acting muscarinic antagonist (LAMA)
Hypertension
Ischemic heart disease
Cardiac arrhythmia
Heart failure
Cerebrovascular disease
Peripheral vascular disease
15. Asthma
16. Bronchiectasis
17. Diabetes mellitus
18. Dyslipidemia
19. Chronic kidney disease
20. Osteoporosis
21. Gastroesophageal reflux disease
22. Chronic liver disease such as liver cirrhosis or fatty liver disease
23. Malignancy
24. Lung cancer
25. Stomach cancer
26. Colorectal cancer
27. Liver cancer
28. Thyroid cancer

## Slide 3
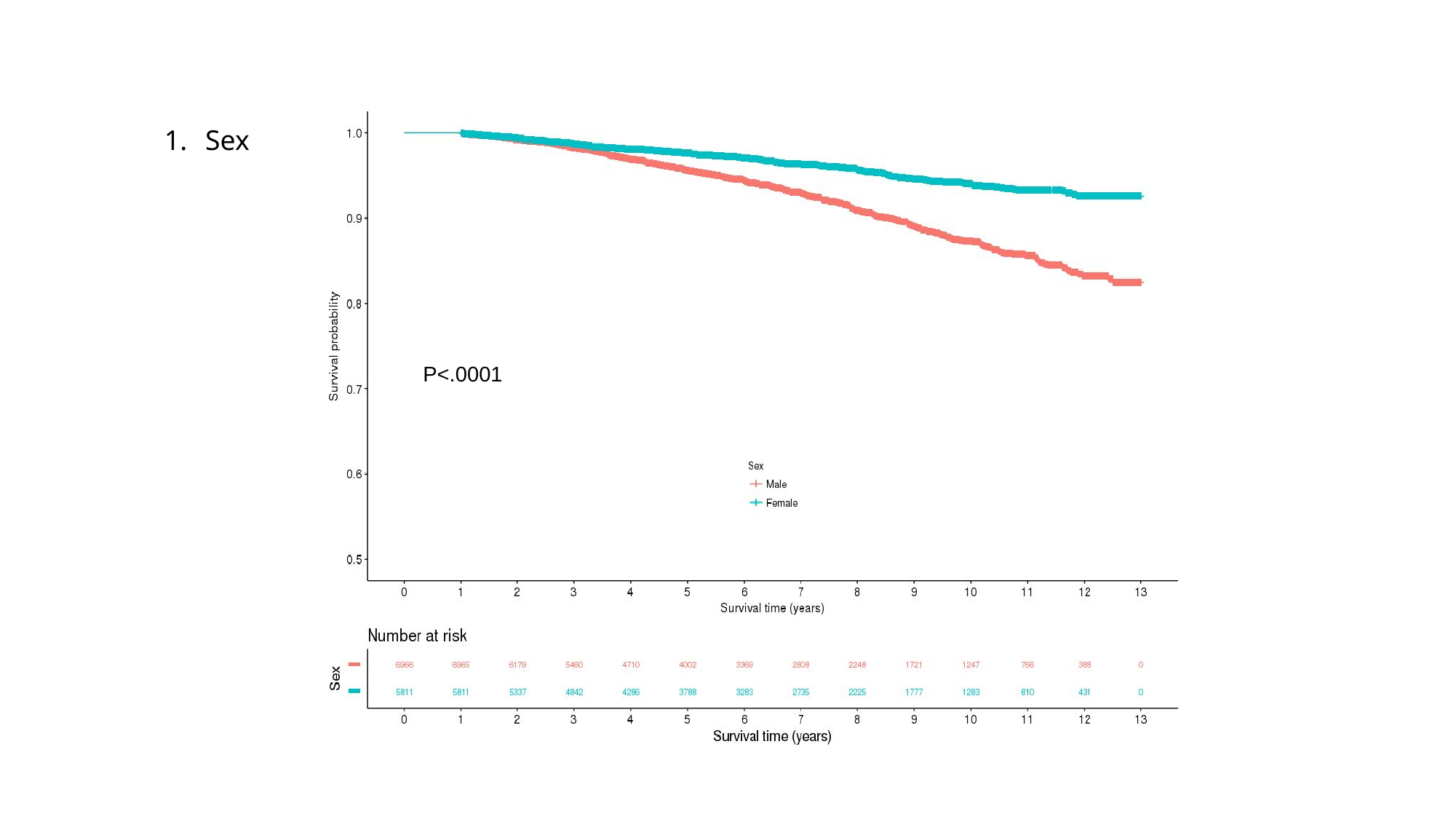

P<.0001
Sex

## Slide 4
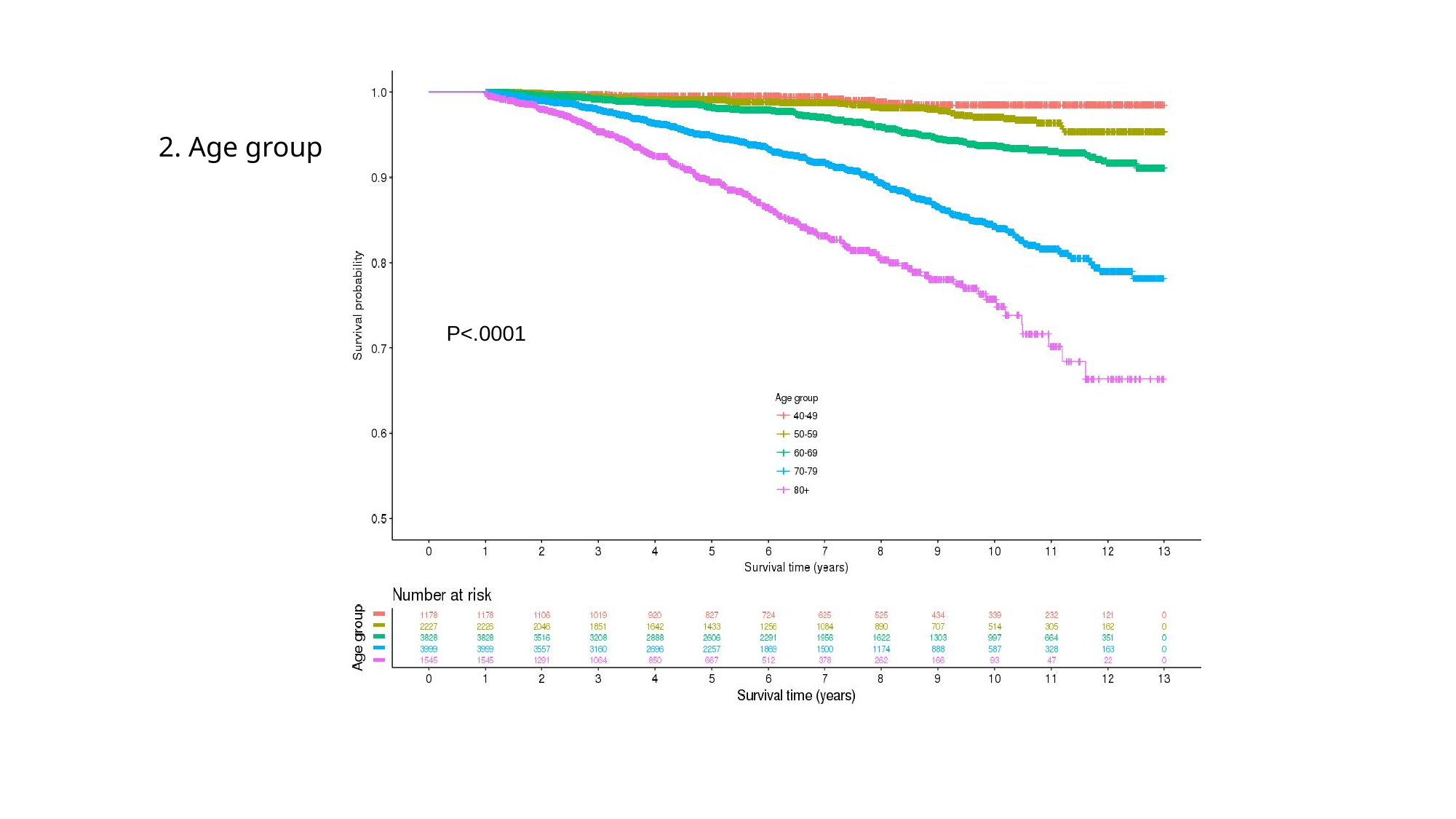

P<.0001
2. Age group

## Slide 5
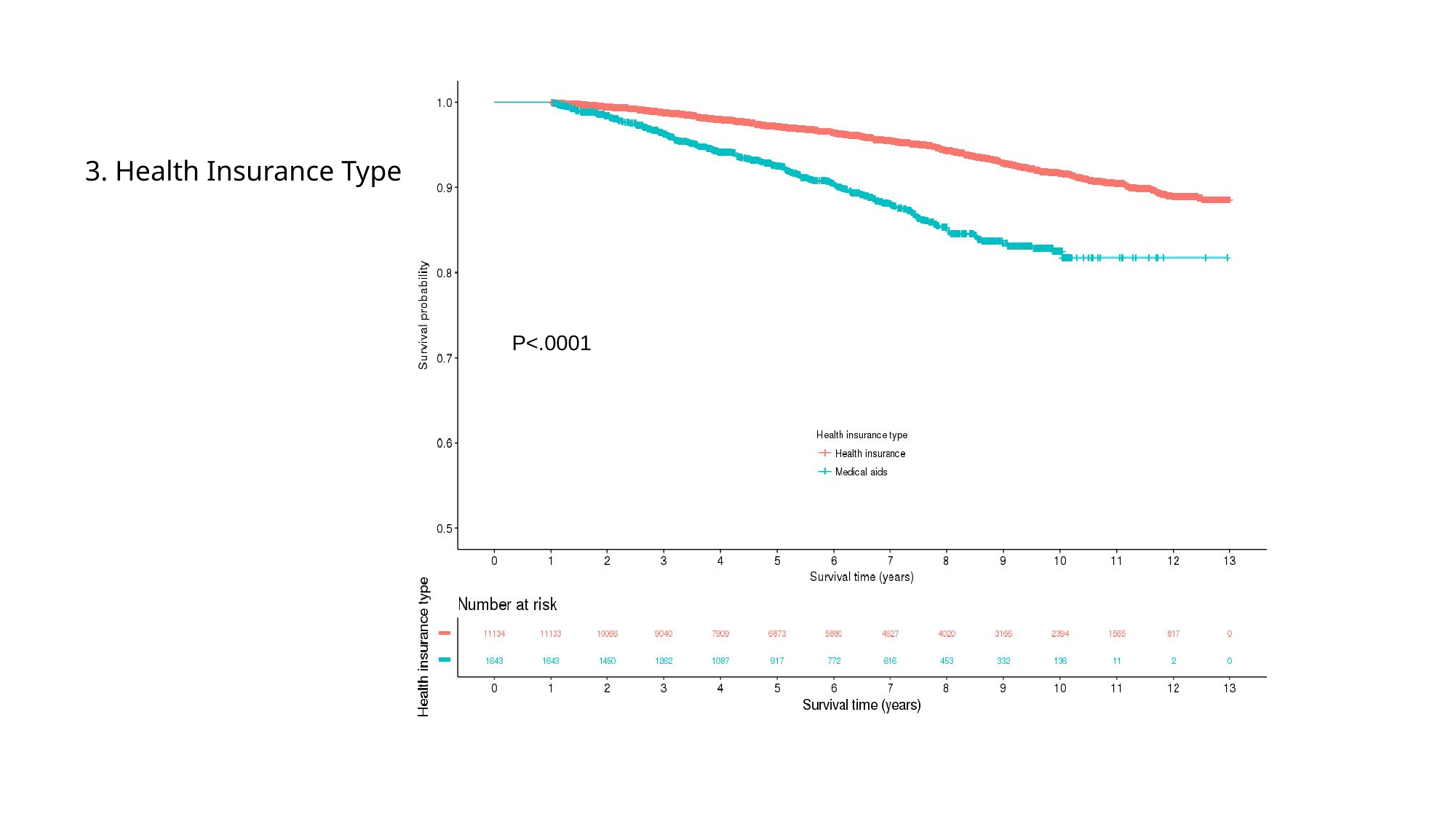

P<.0001
3. Health Insurance Type

## Slide 6
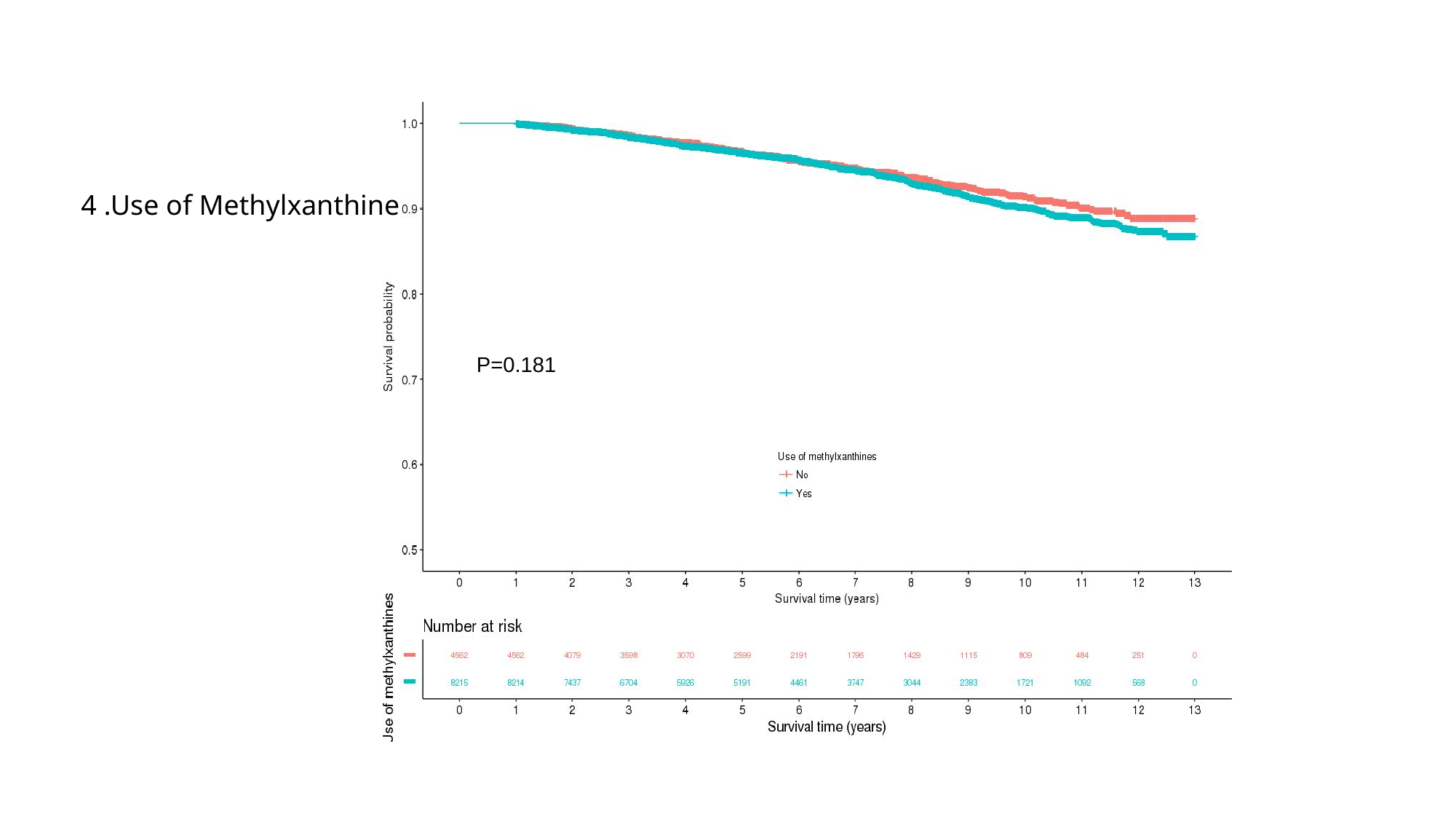

P=0.181
4 .Use of Methylxanthine

## Slide 7
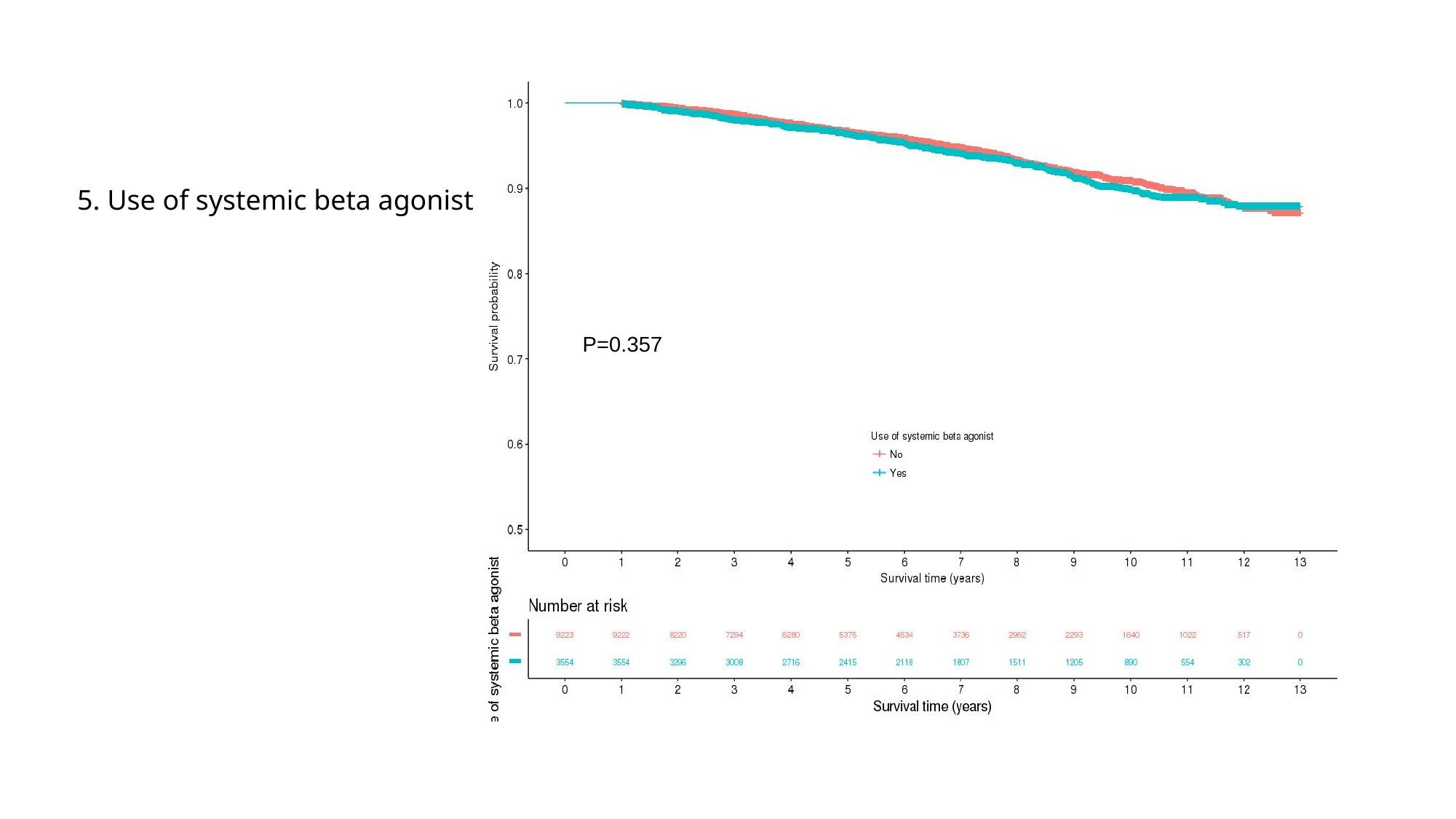

P=0.357
5. Use of systemic beta agonist

## Slide 8
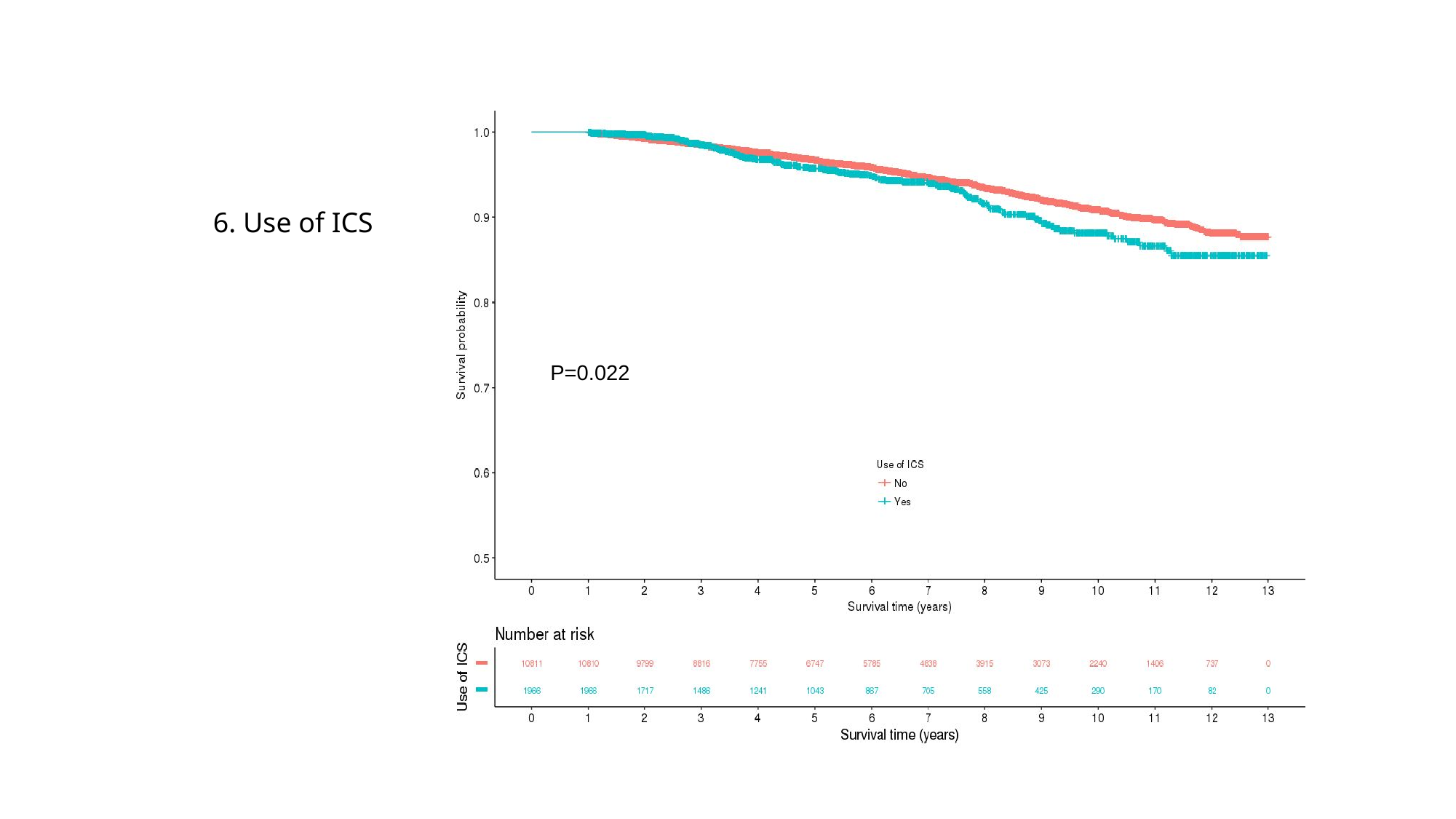

P=0.022
6. Use of ICS

## Slide 9
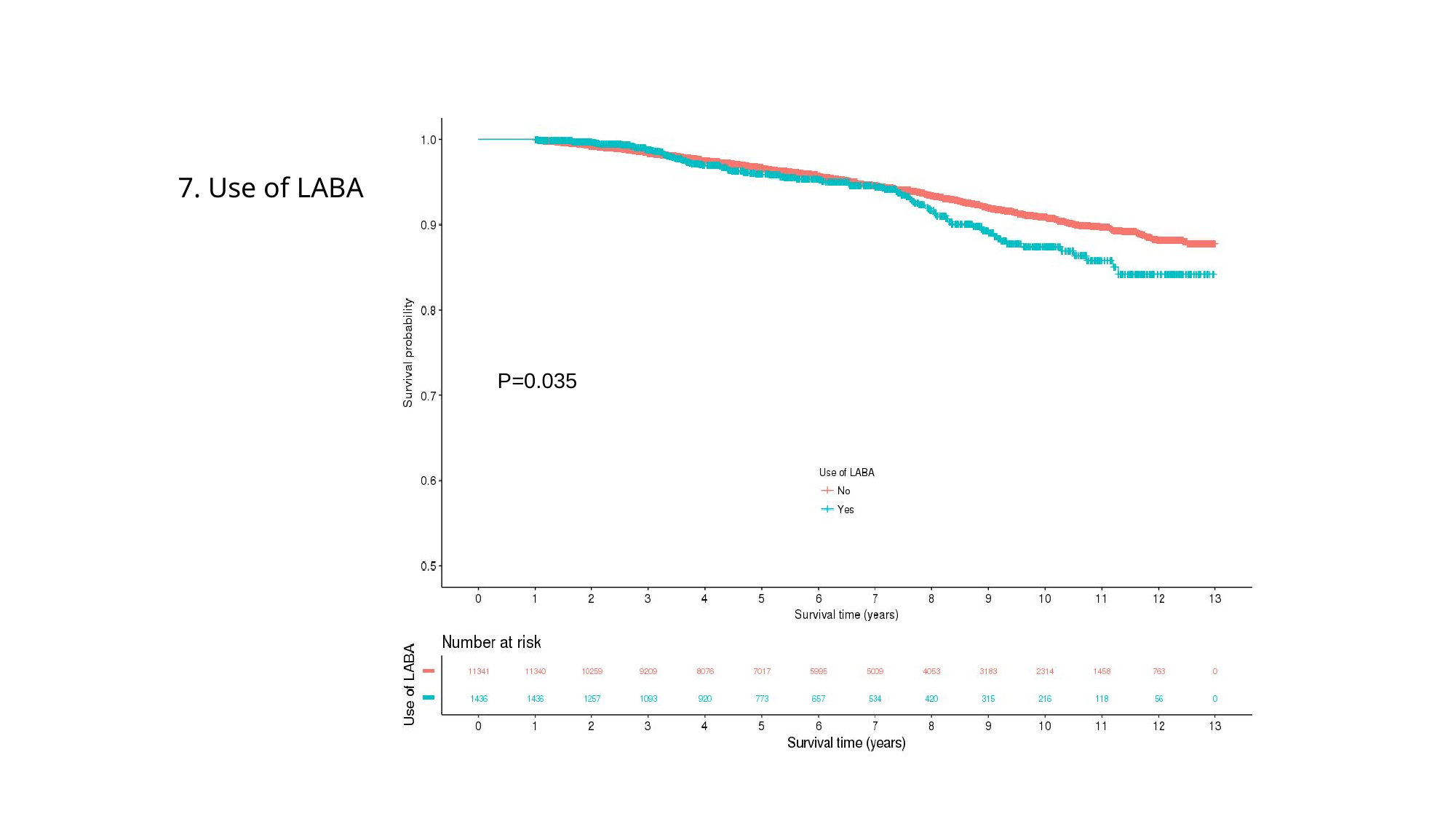

P=0.035
7. Use of LABA

## Slide 10
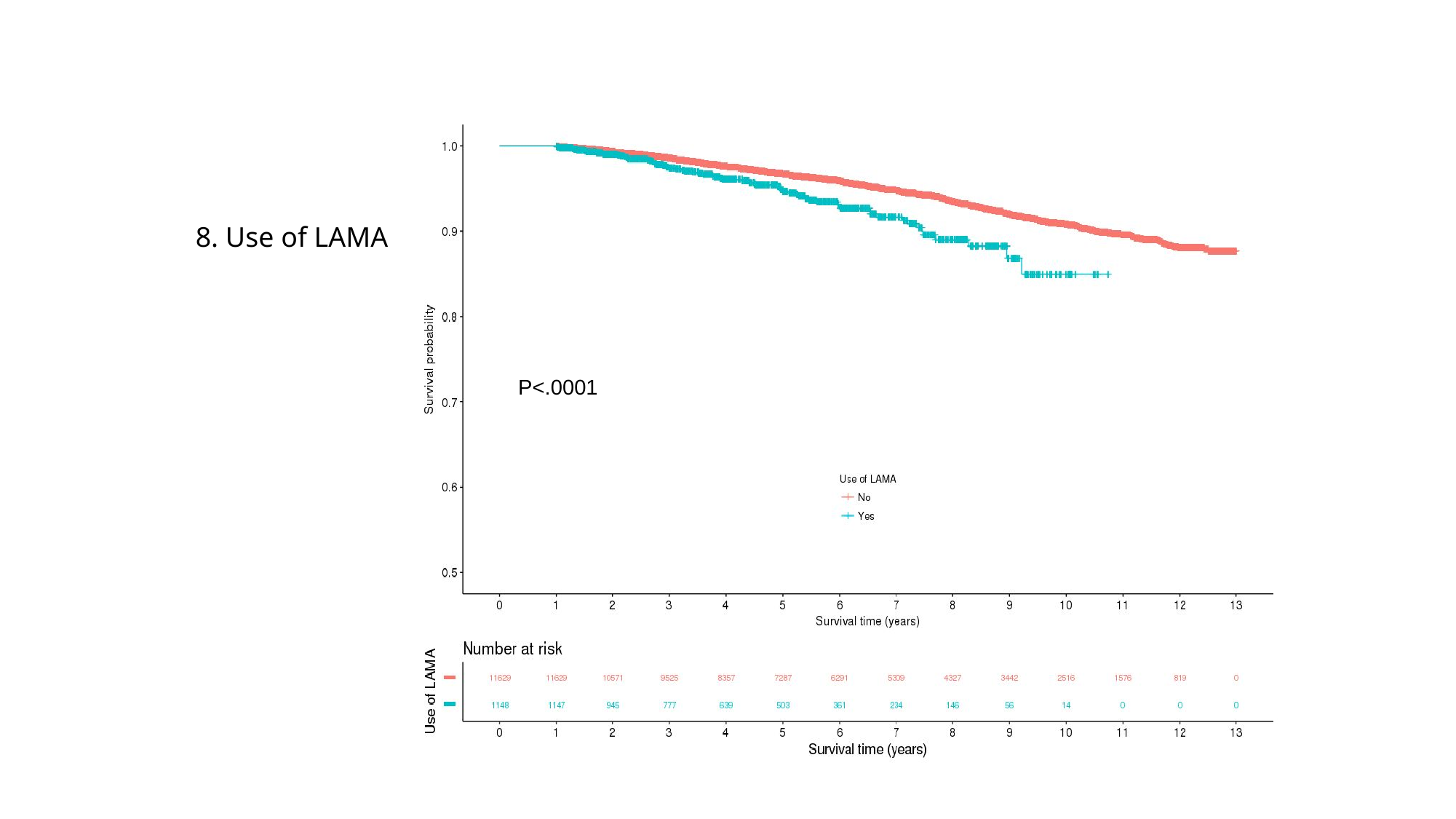

P<.0001
8. Use of LAMA

## Slide 11
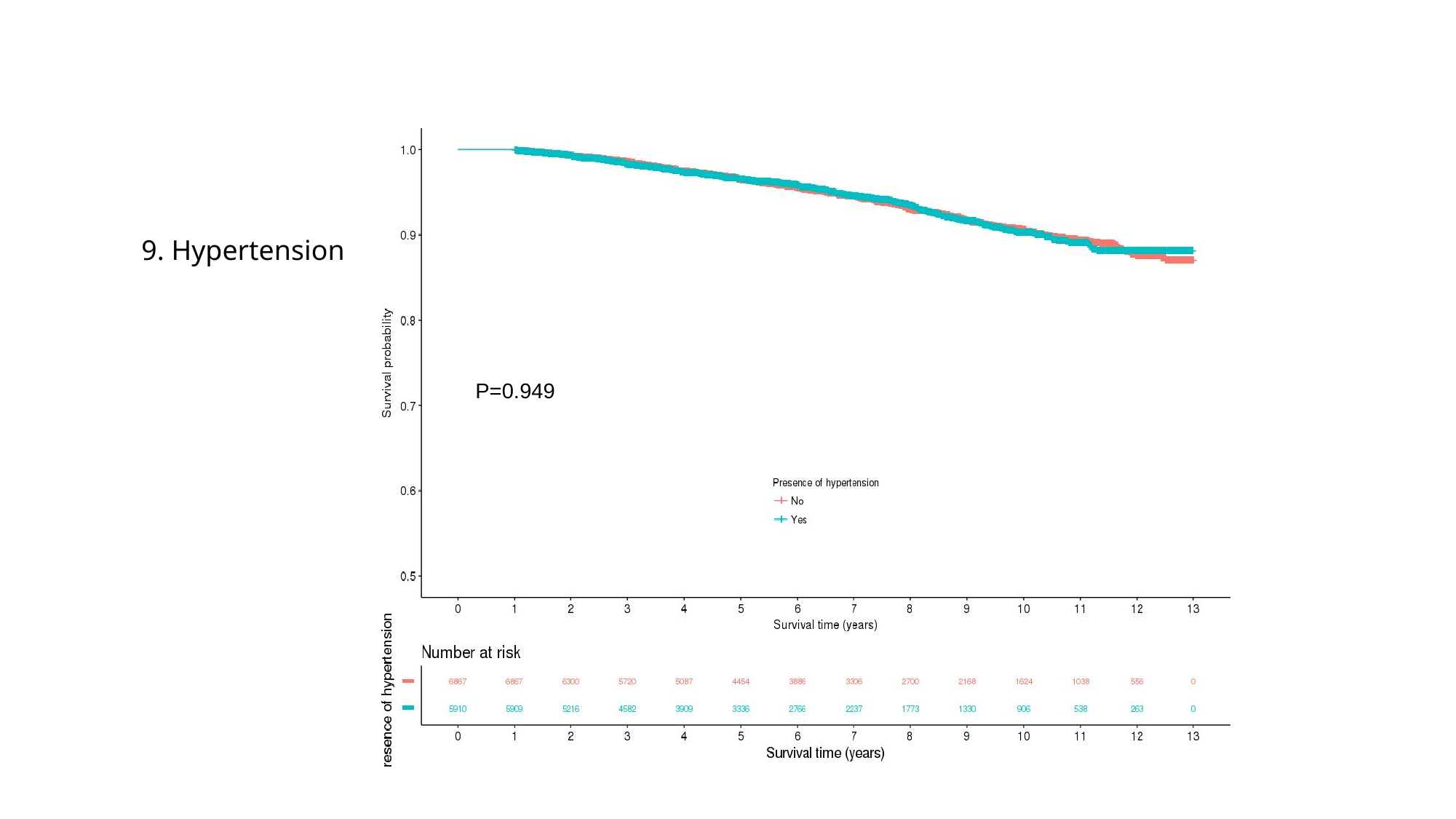

P=0.949
9. Hypertension

## Slide 12
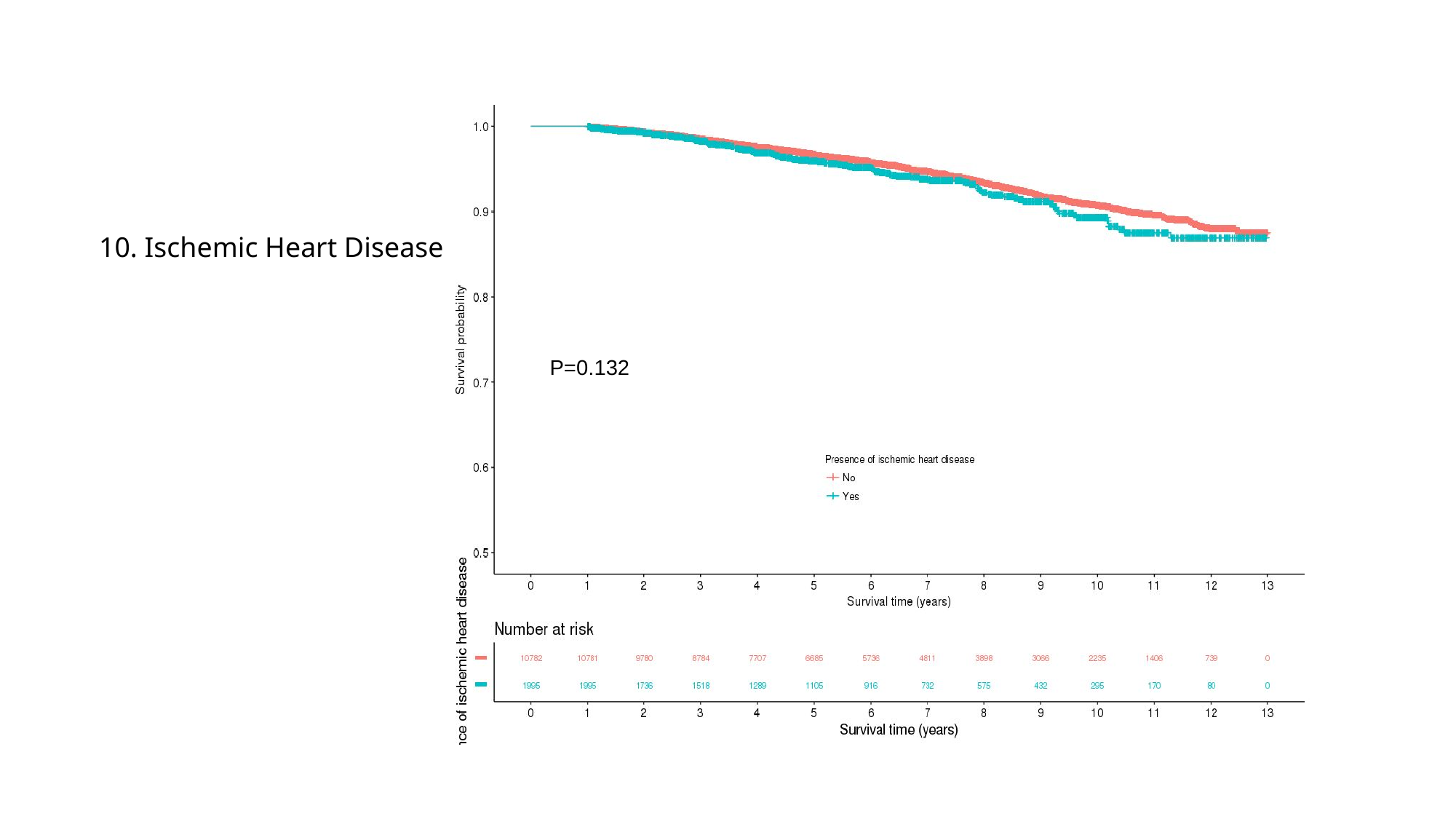

P=0.132
10. Ischemic Heart Disease

## Slide 13
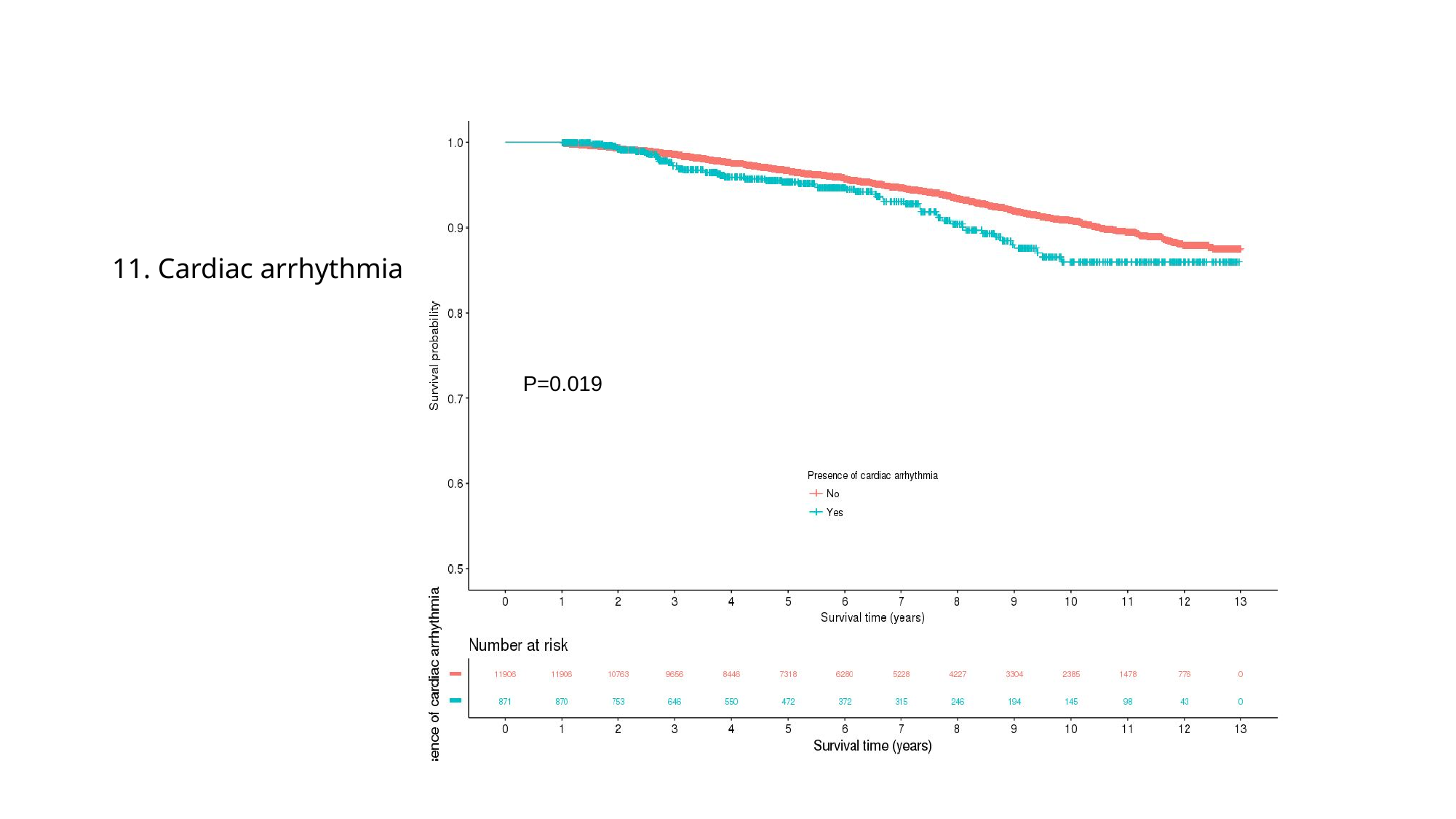

P=0.019
11. Cardiac arrhythmia

## Slide 14
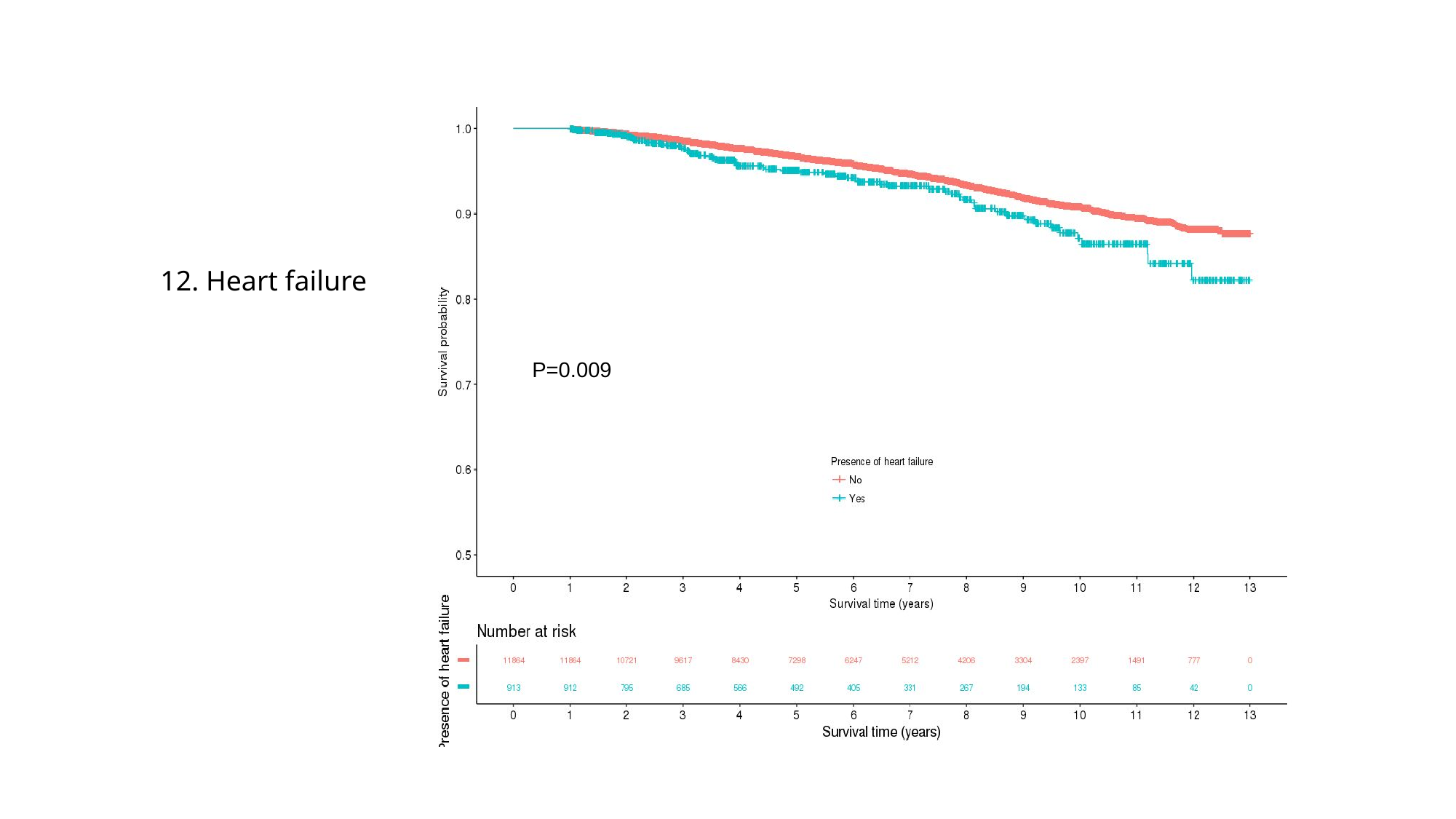

P=0.009
12. Heart failure

## Slide 15
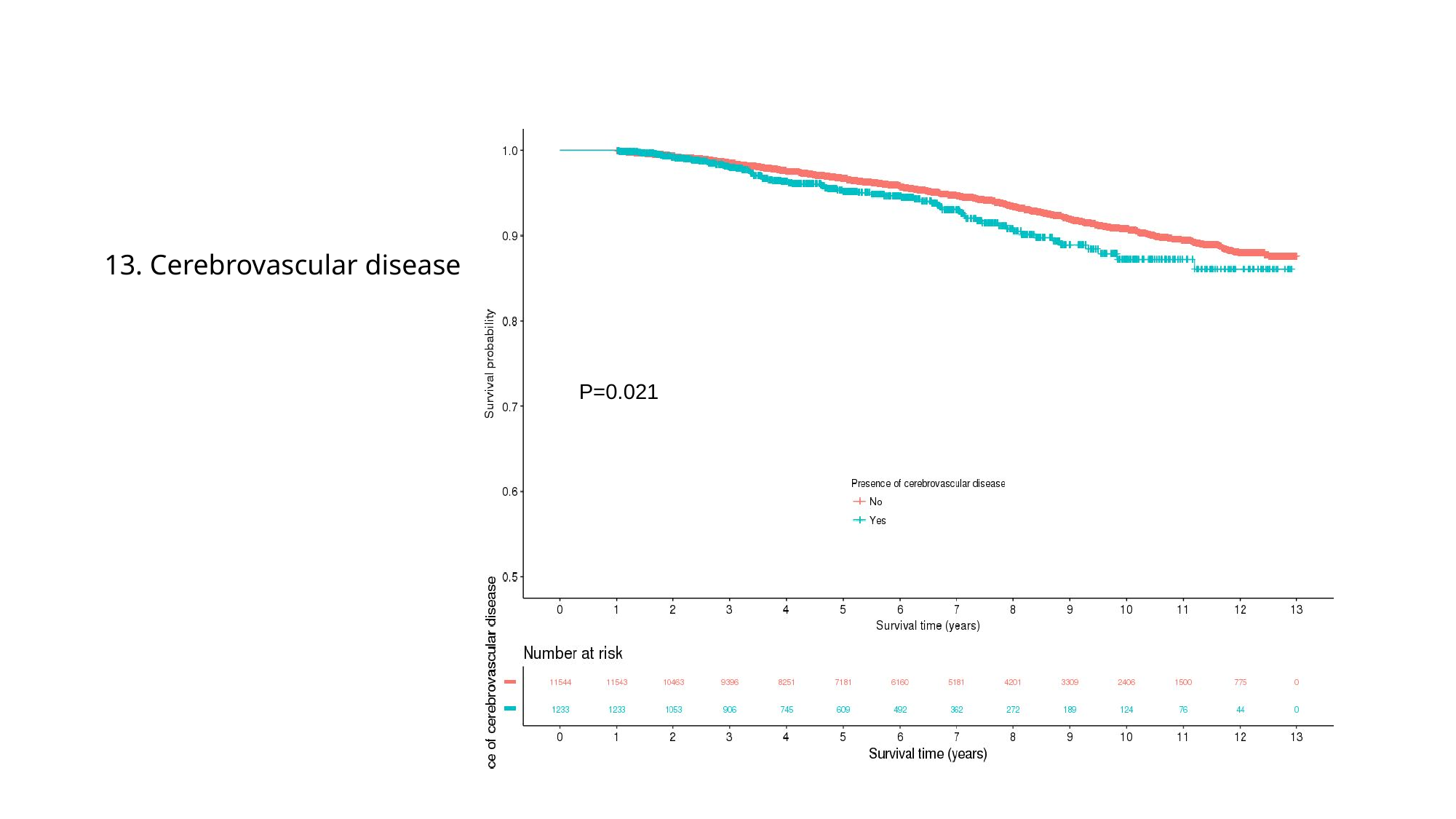

P=0.021
13. Cerebrovascular disease

## Slide 16
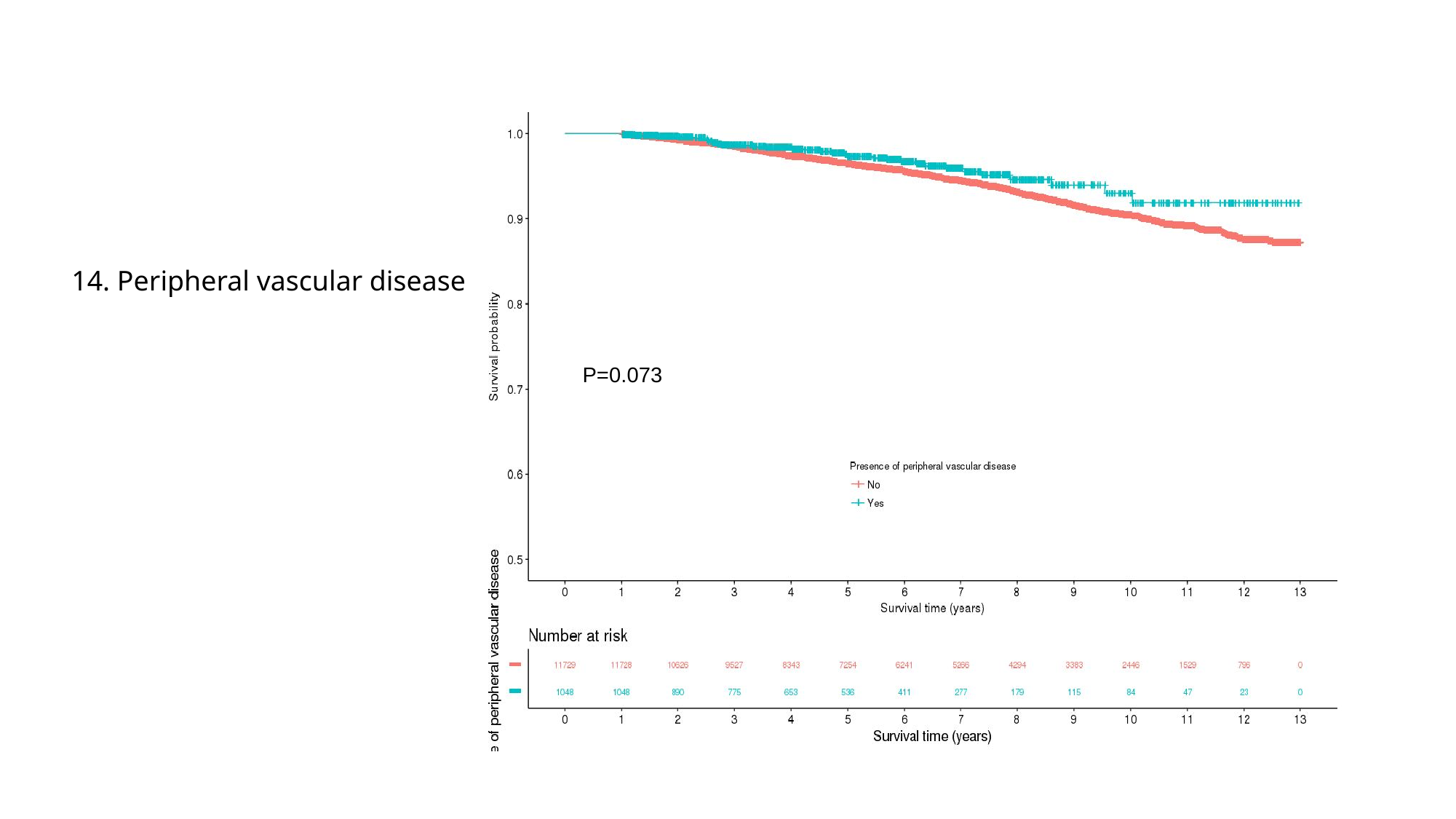

P=0.073
14. Peripheral vascular disease

## Slide 17
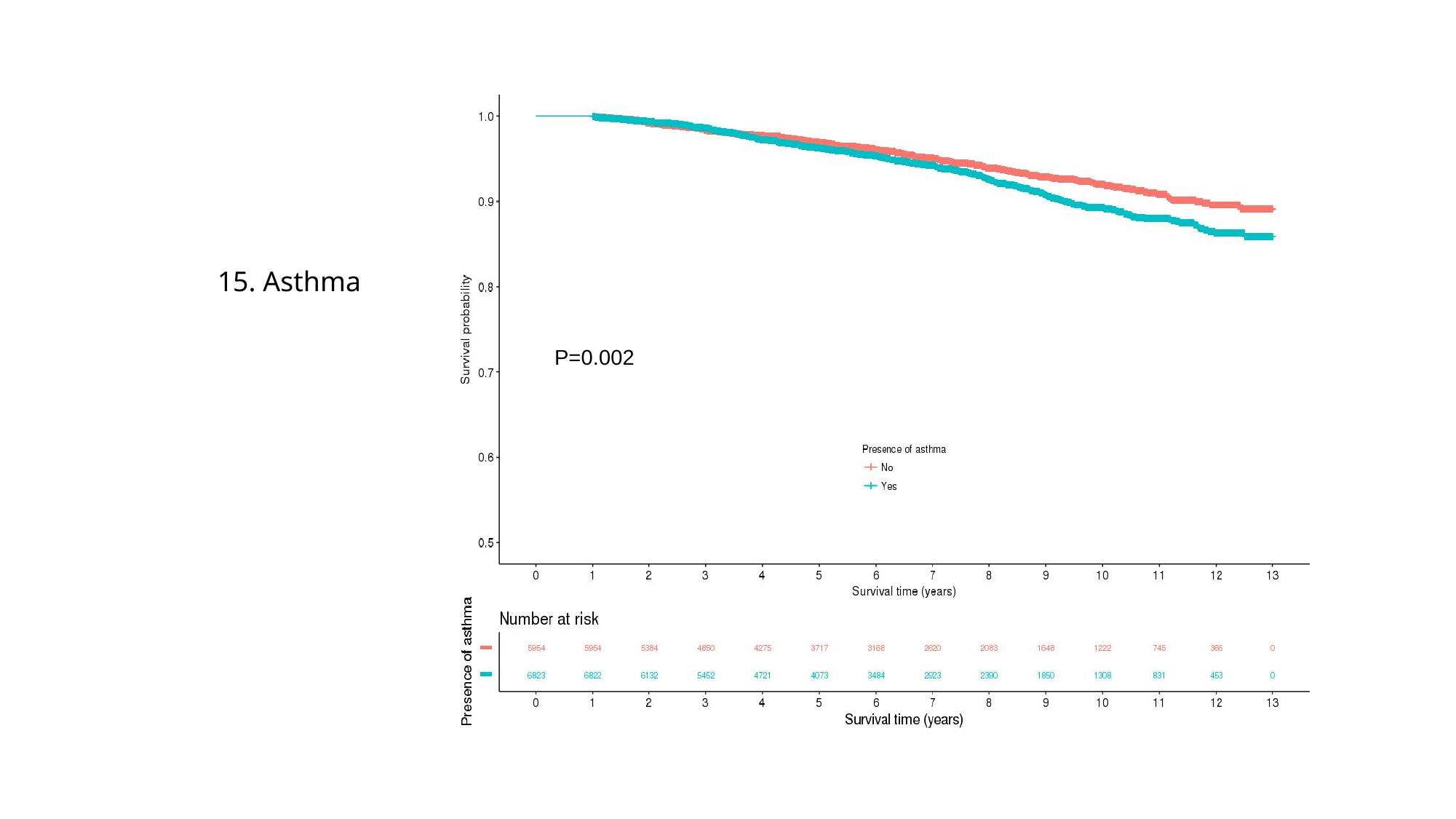

P=0.002
15. Asthma

## Slide 18
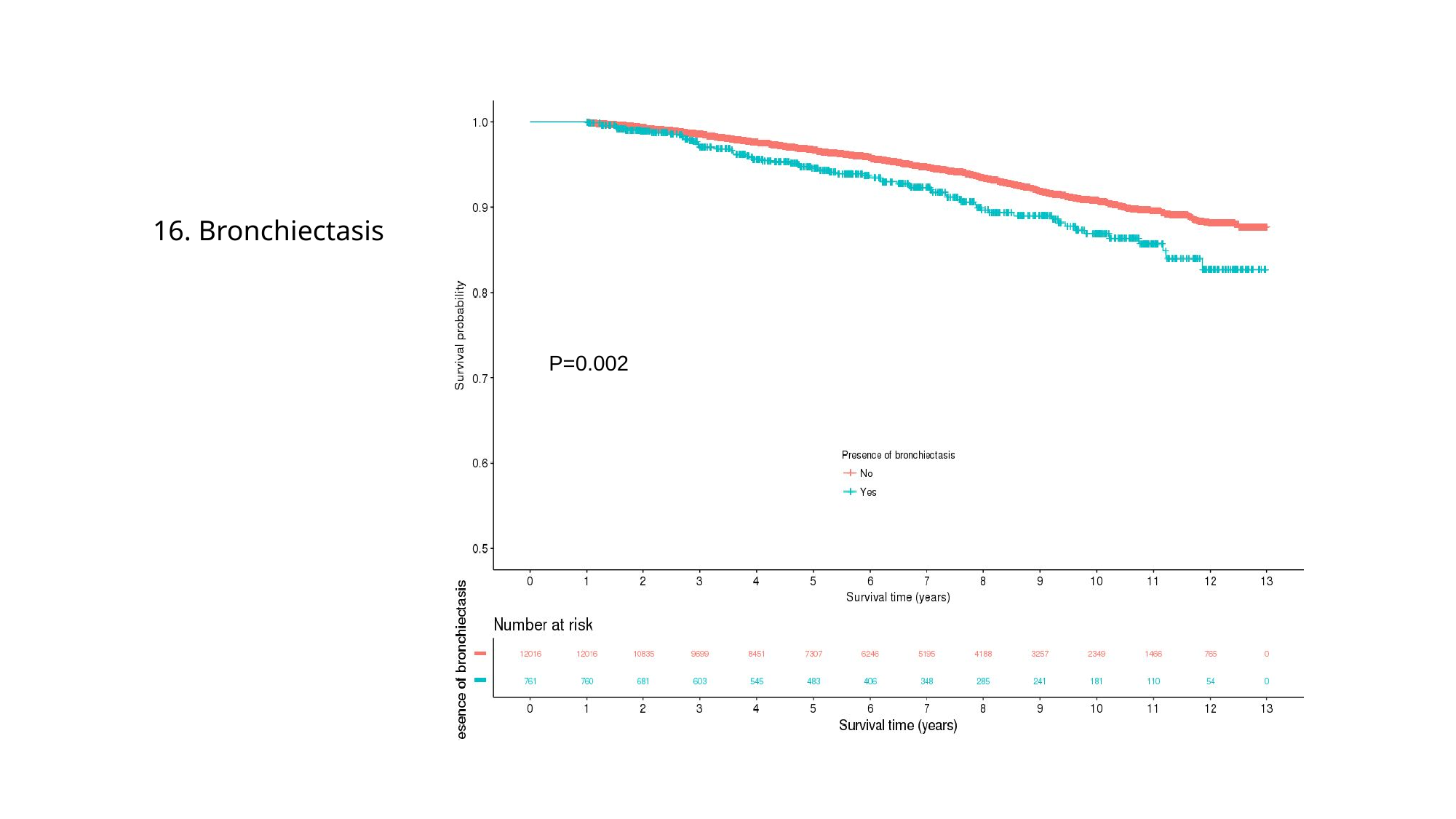

P=0.002
16. Bronchiectasis

## Slide 19
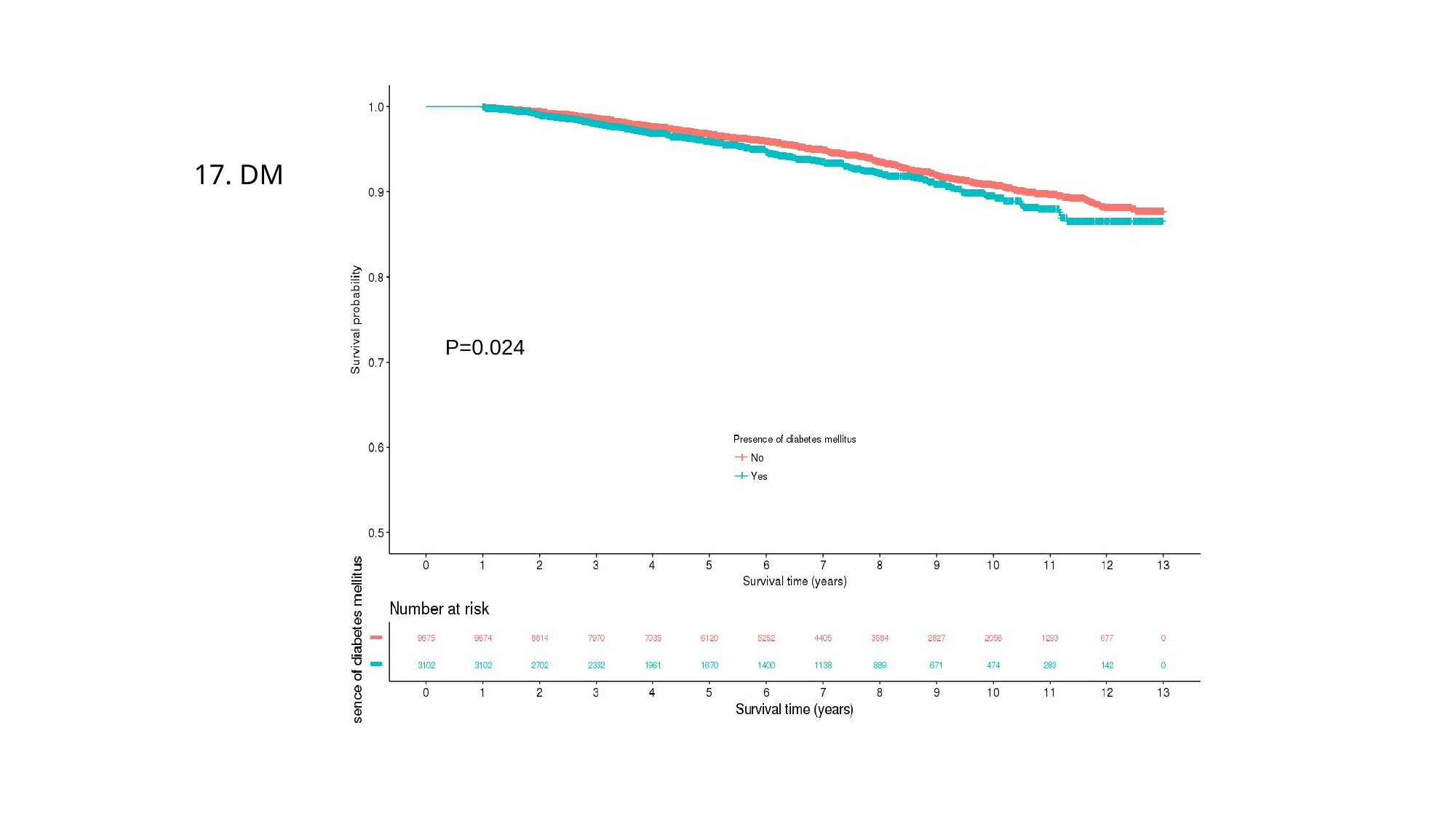

P=0.024
17. DM

## Slide 20
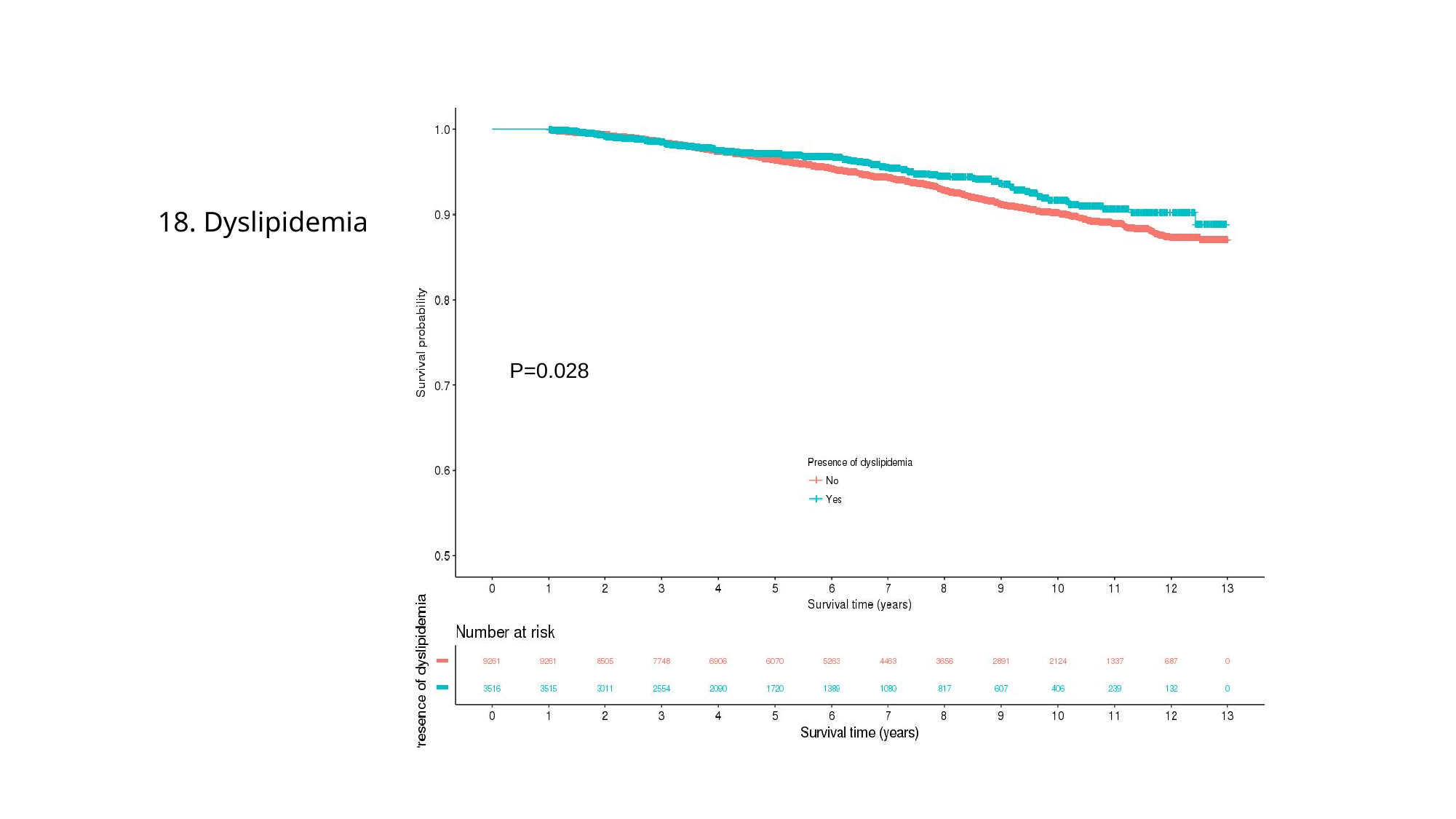

P=0.028
18. Dyslipidemia

## Slide 21
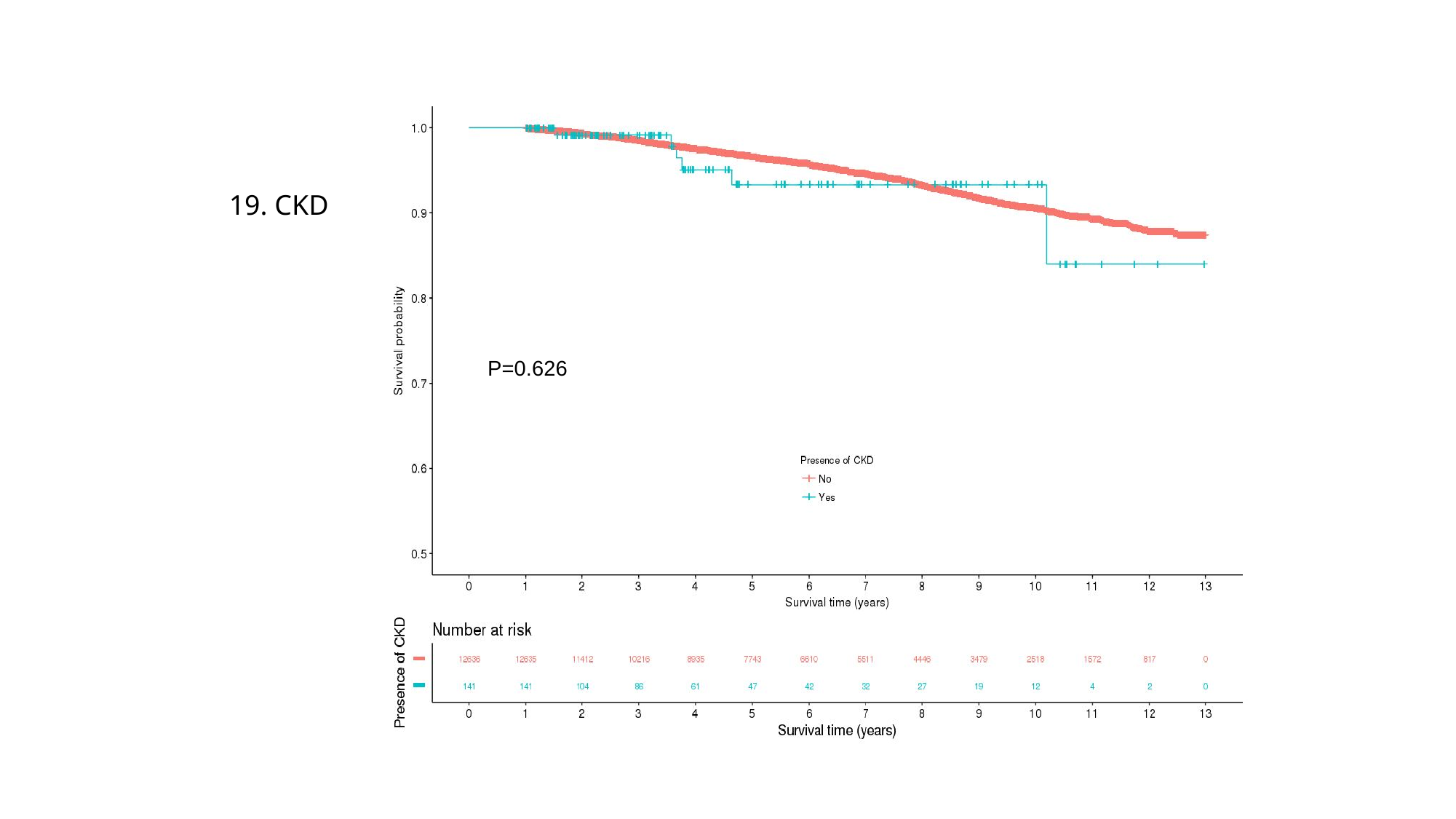

P=0.626
19. CKD

## Slide 22
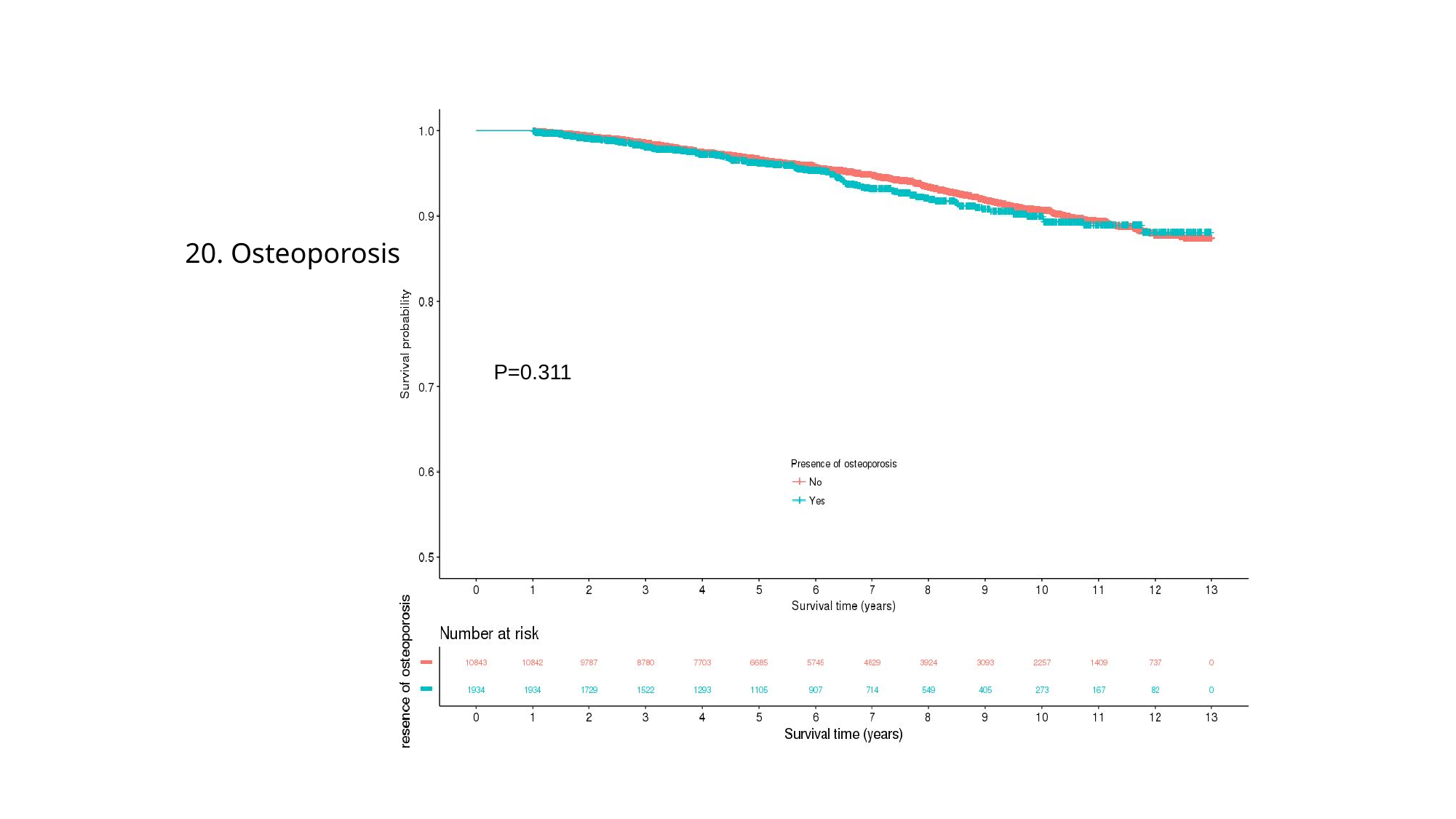

P=0.311
20. Osteoporosis

## Slide 23
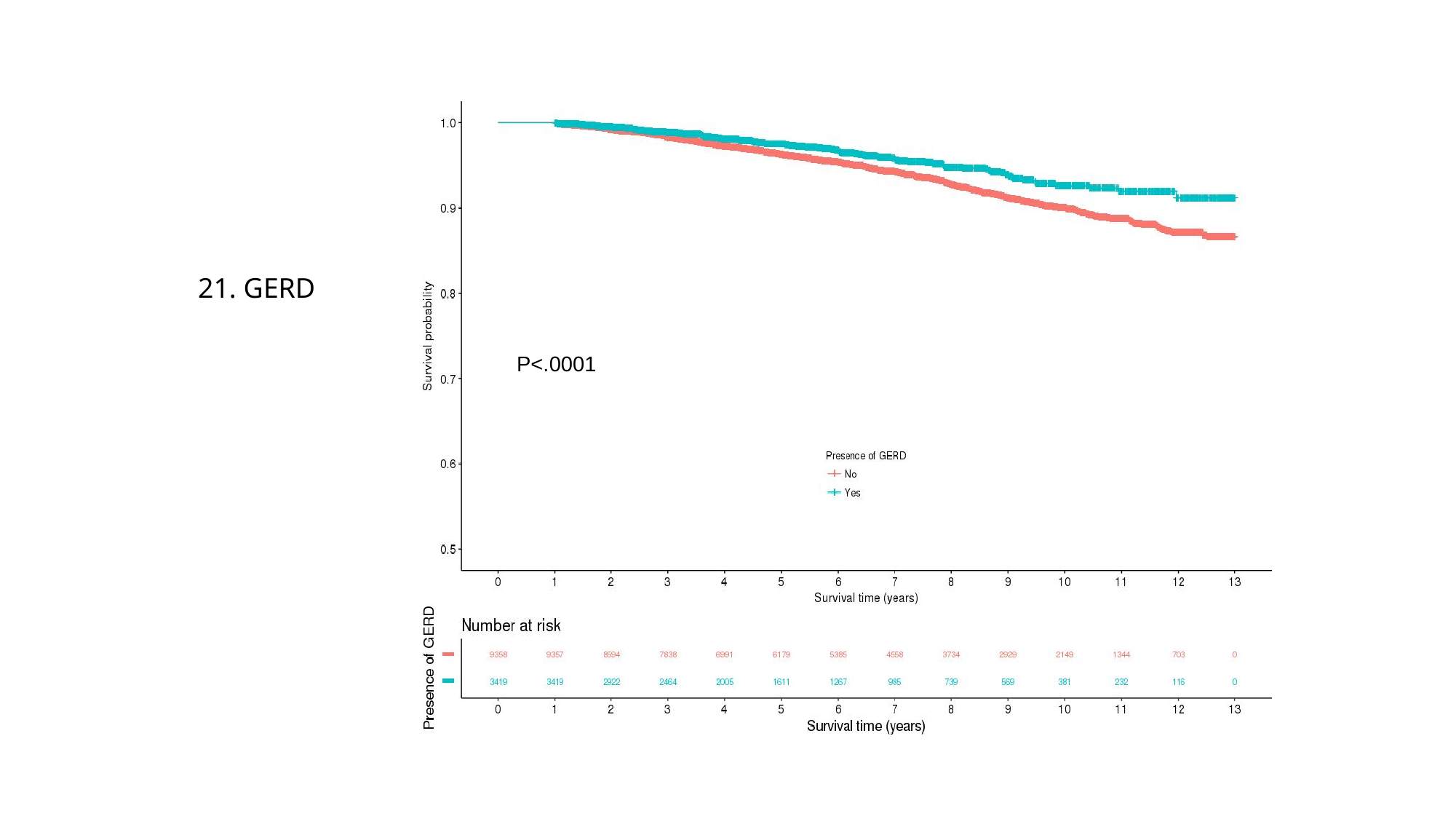

P<.0001
21. GERD

## Slide 24
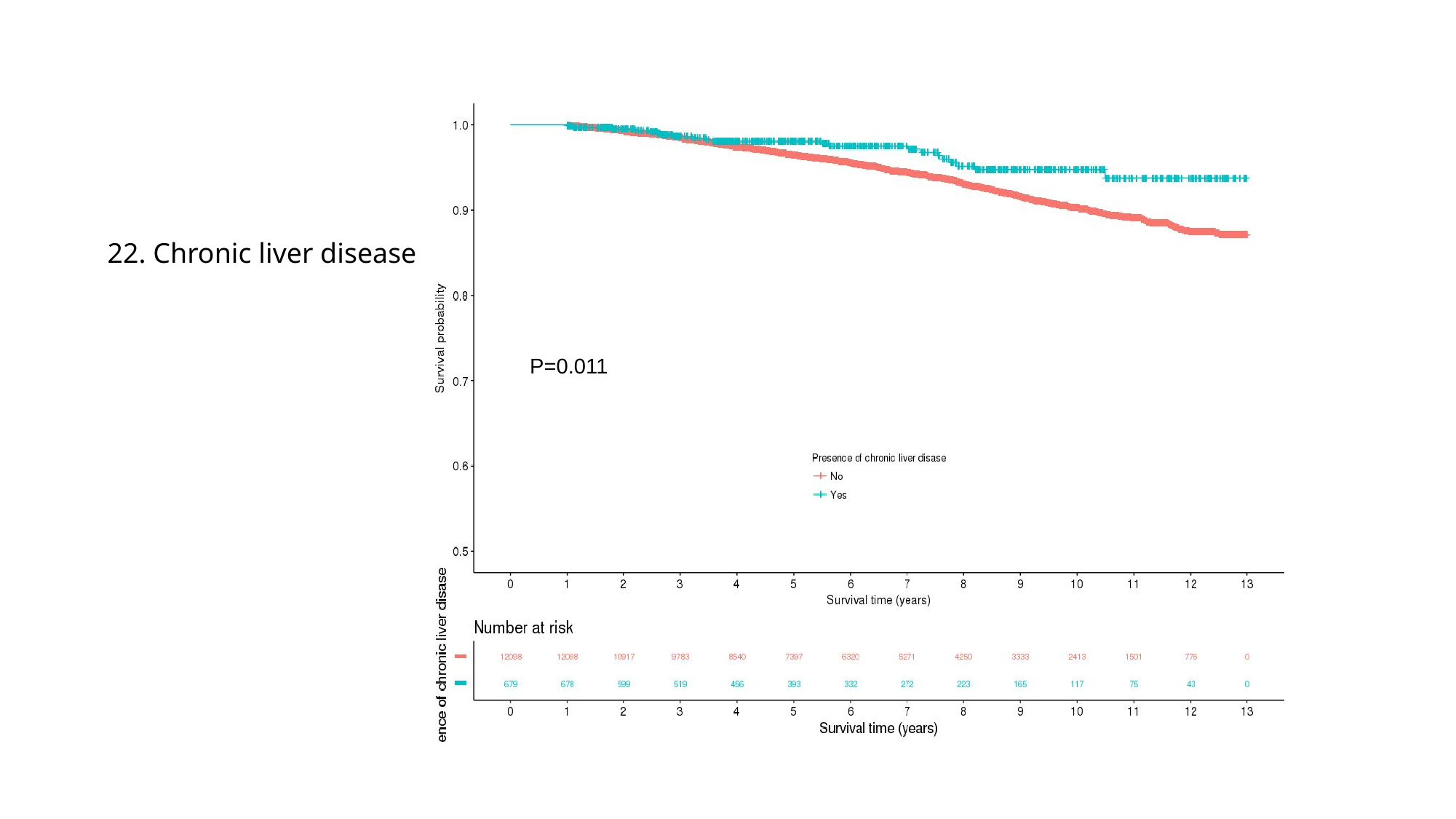

P=0.011
22. Chronic liver disease

## Slide 25
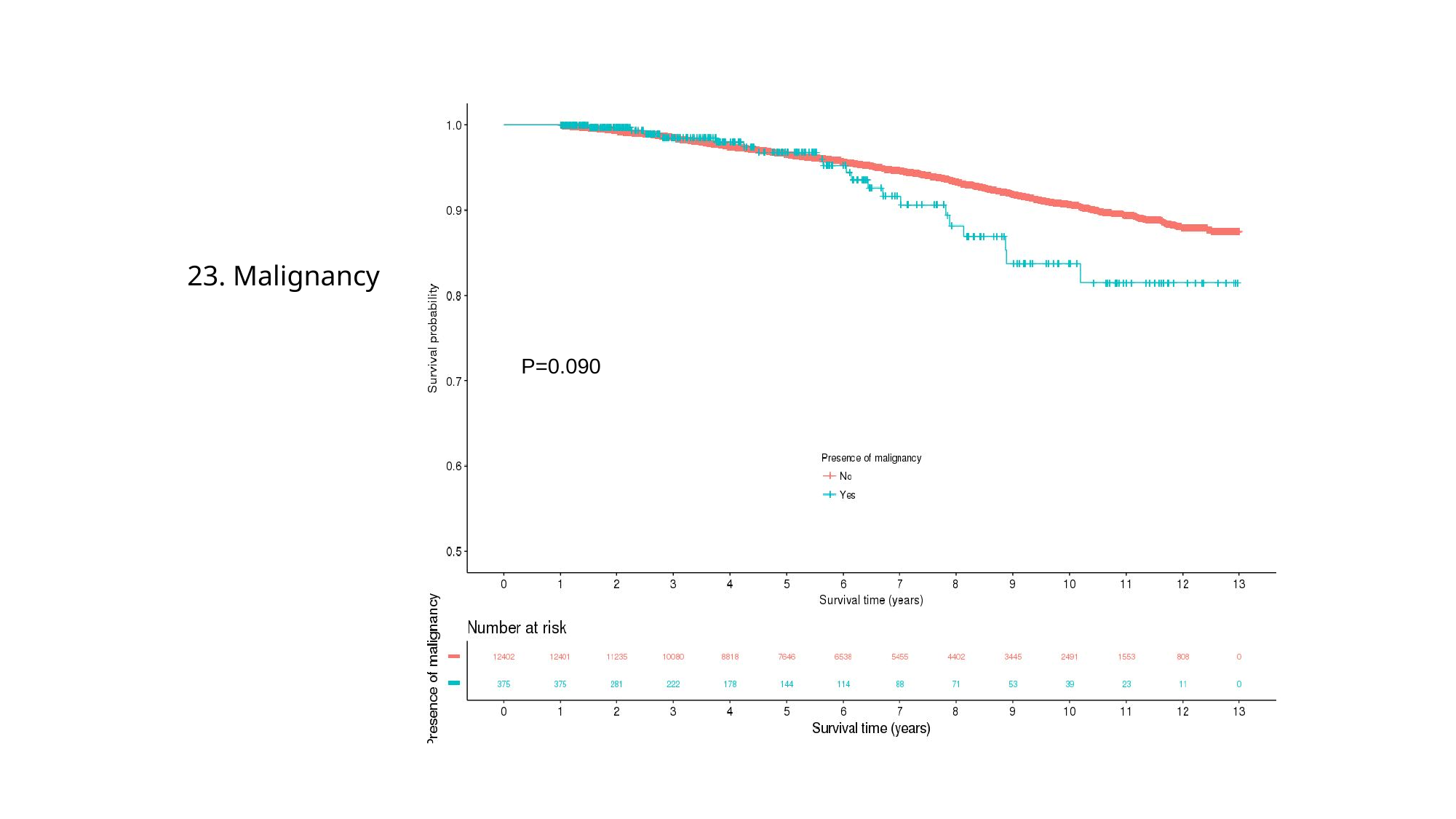

P=0.090
23. Malignancy

## Slide 26
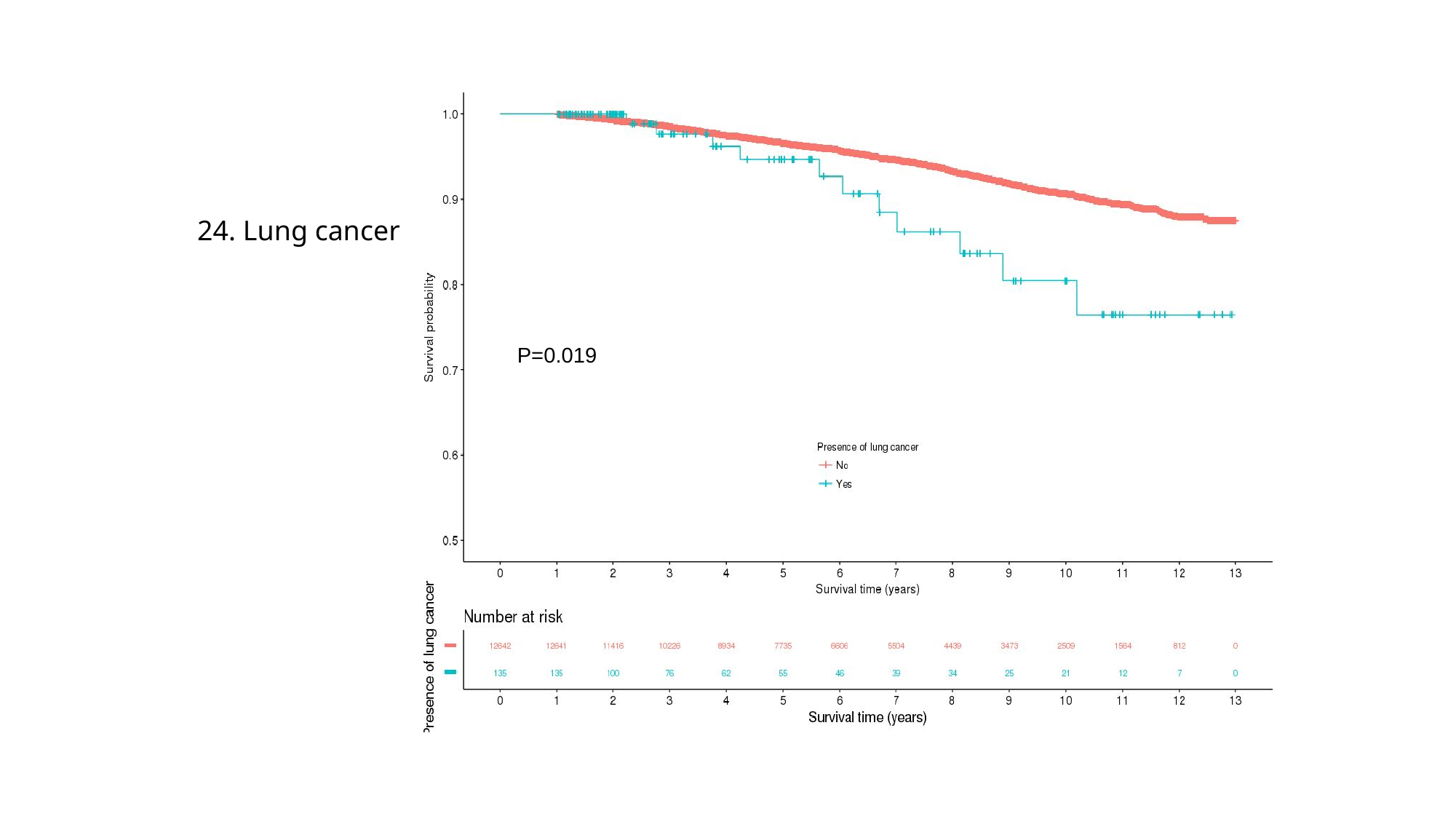

P=0.019
24. Lung cancer

## Slide 27
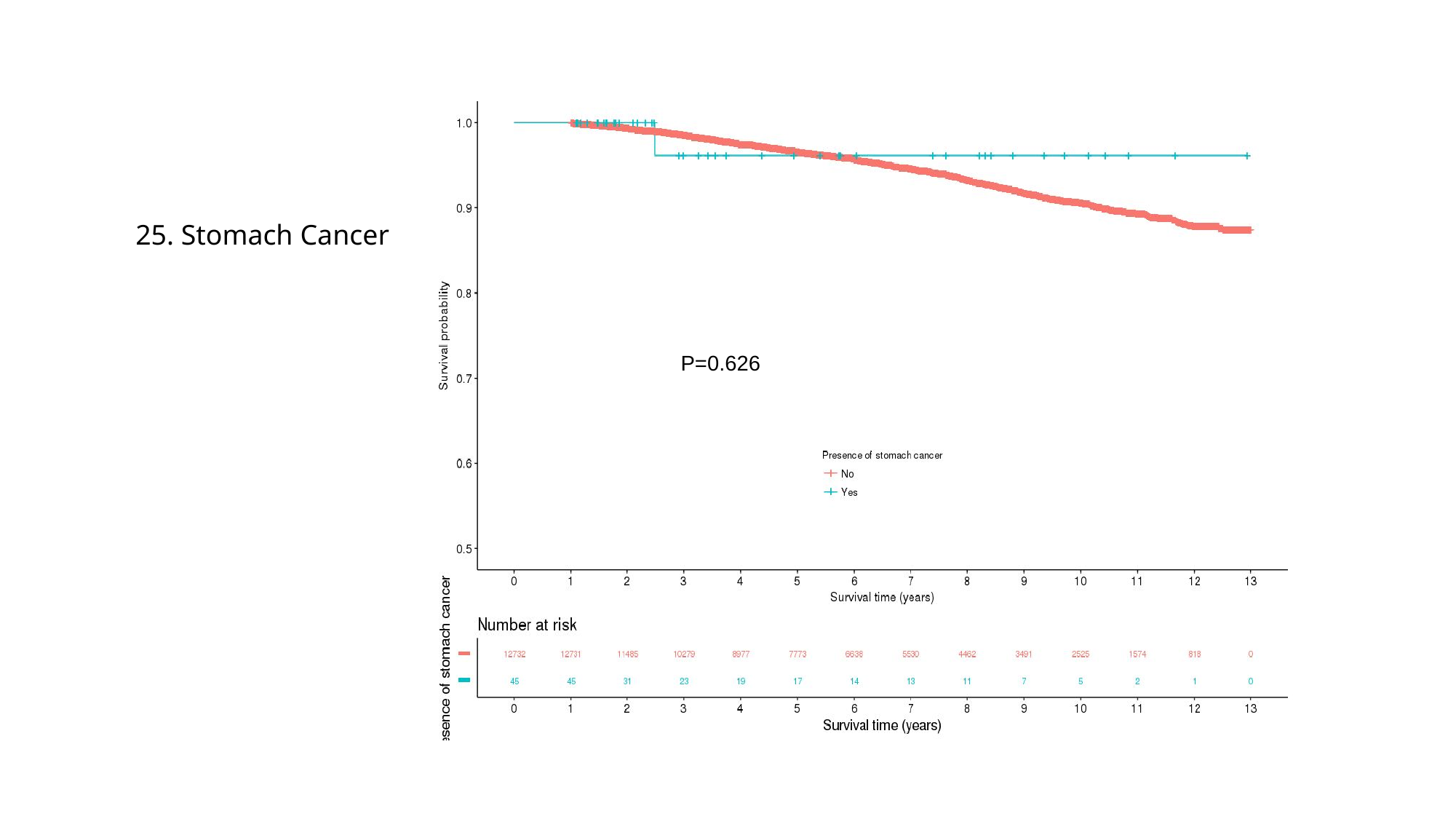

P=0.626
25. Stomach Cancer

## Slide 28
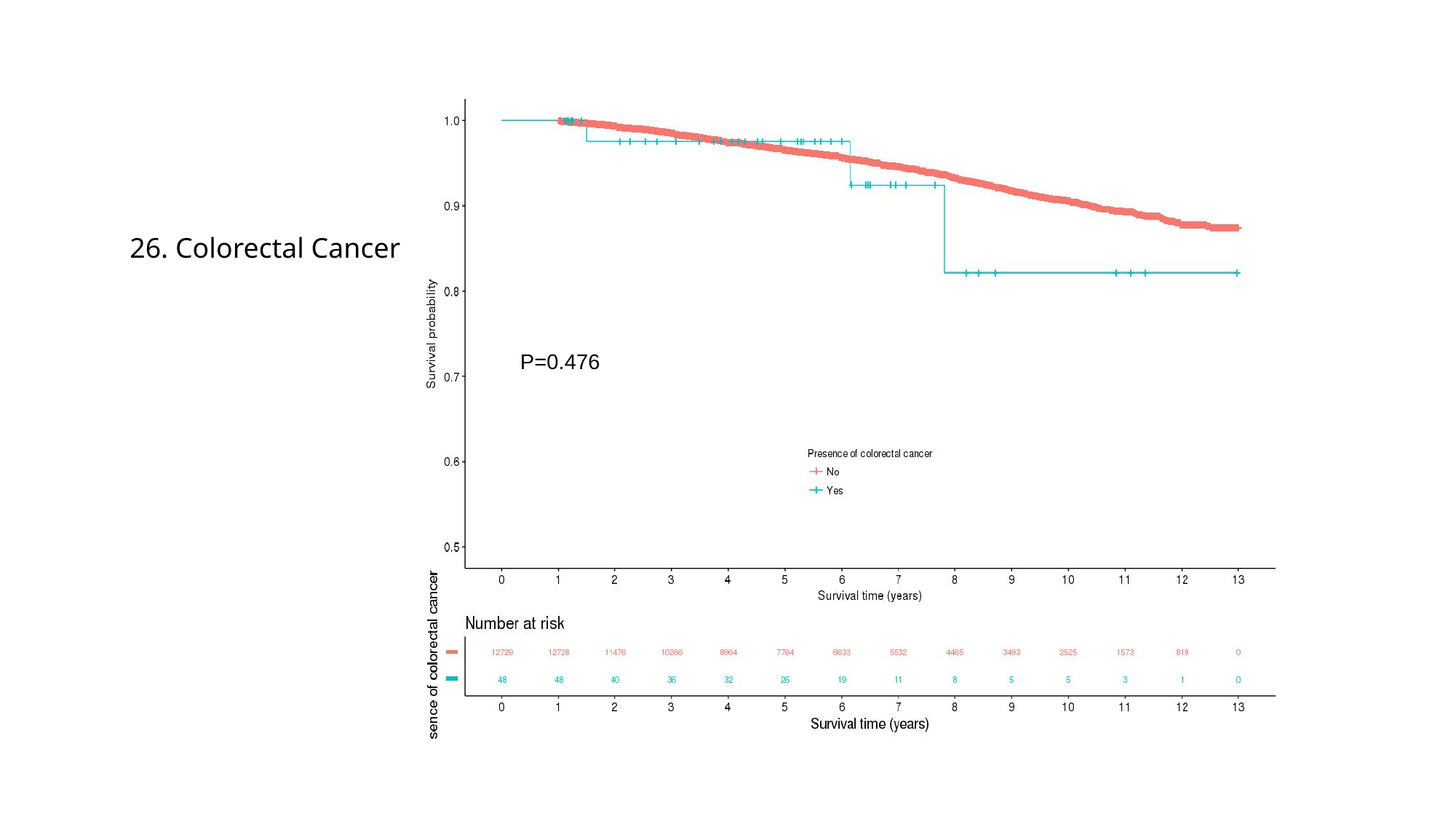

P=0.476
26. Colorectal Cancer

## Slide 29
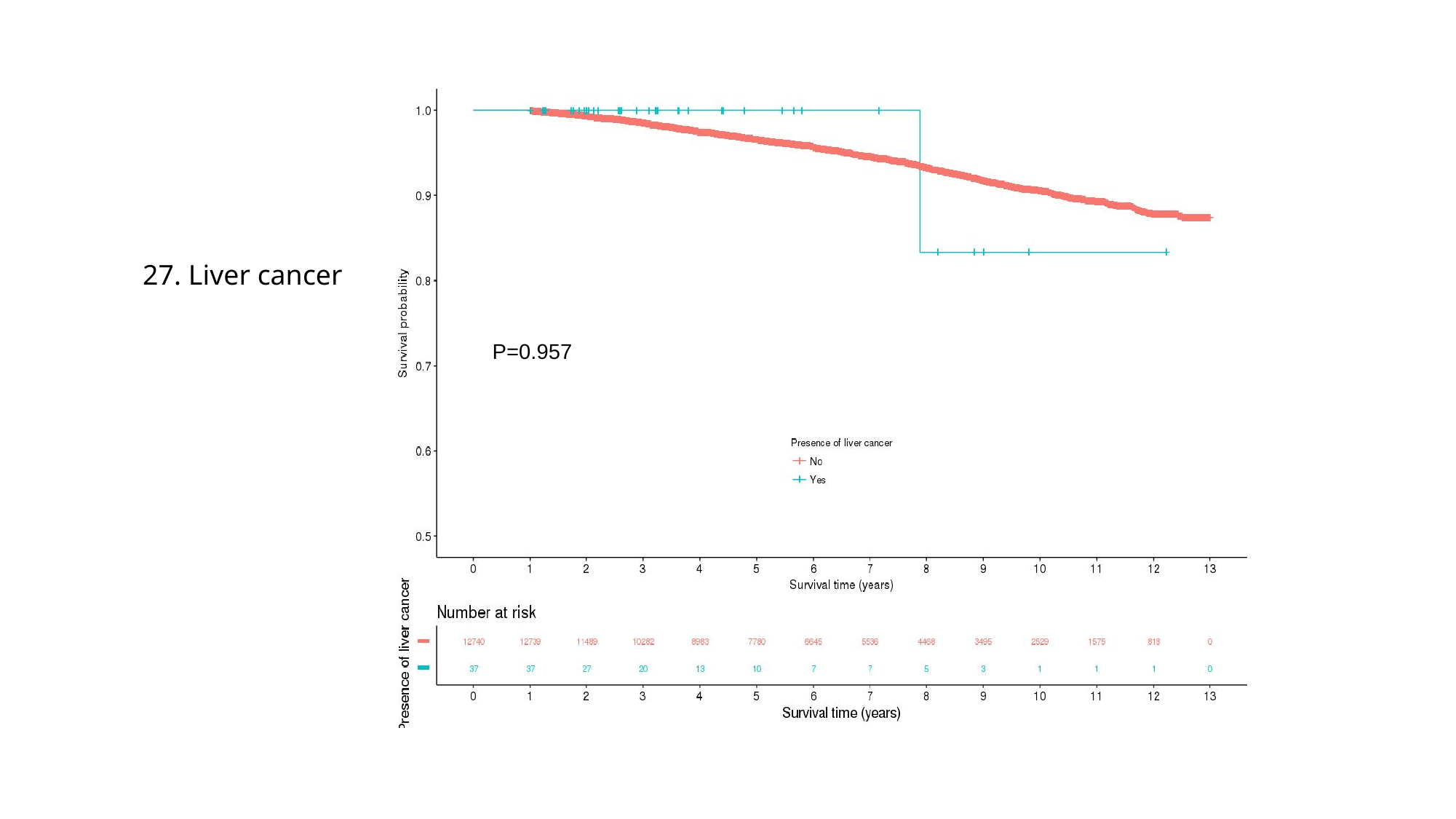

P=0.957
27. Liver cancer

## Slide 30
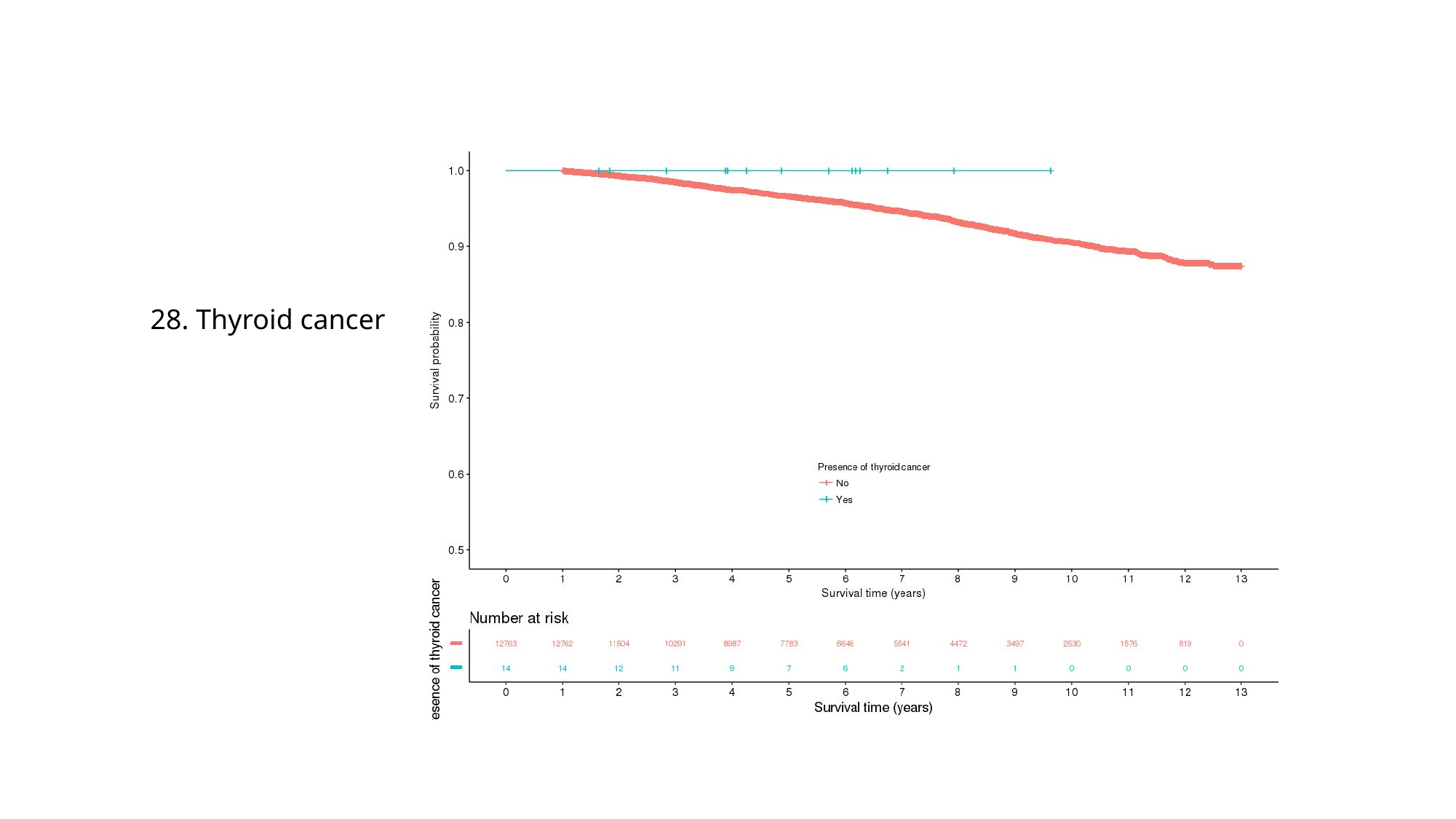

28. Thyroid cancer

## Slide 31
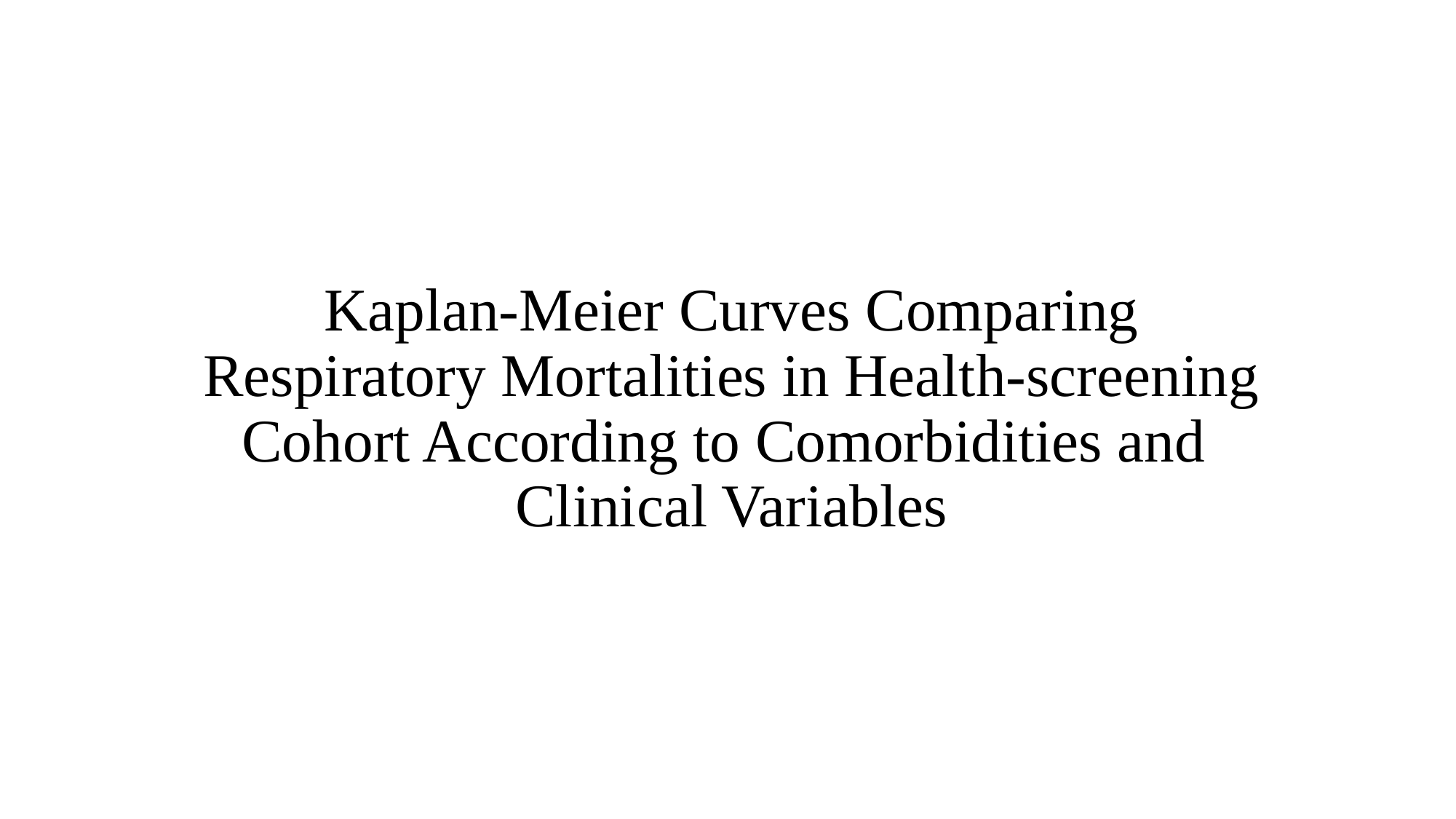

# Kaplan-Meier Curves Comparing Respiratory Mortalities in Health-screening Cohort According to Comorbidities and Clinical Variables

## Slide 32
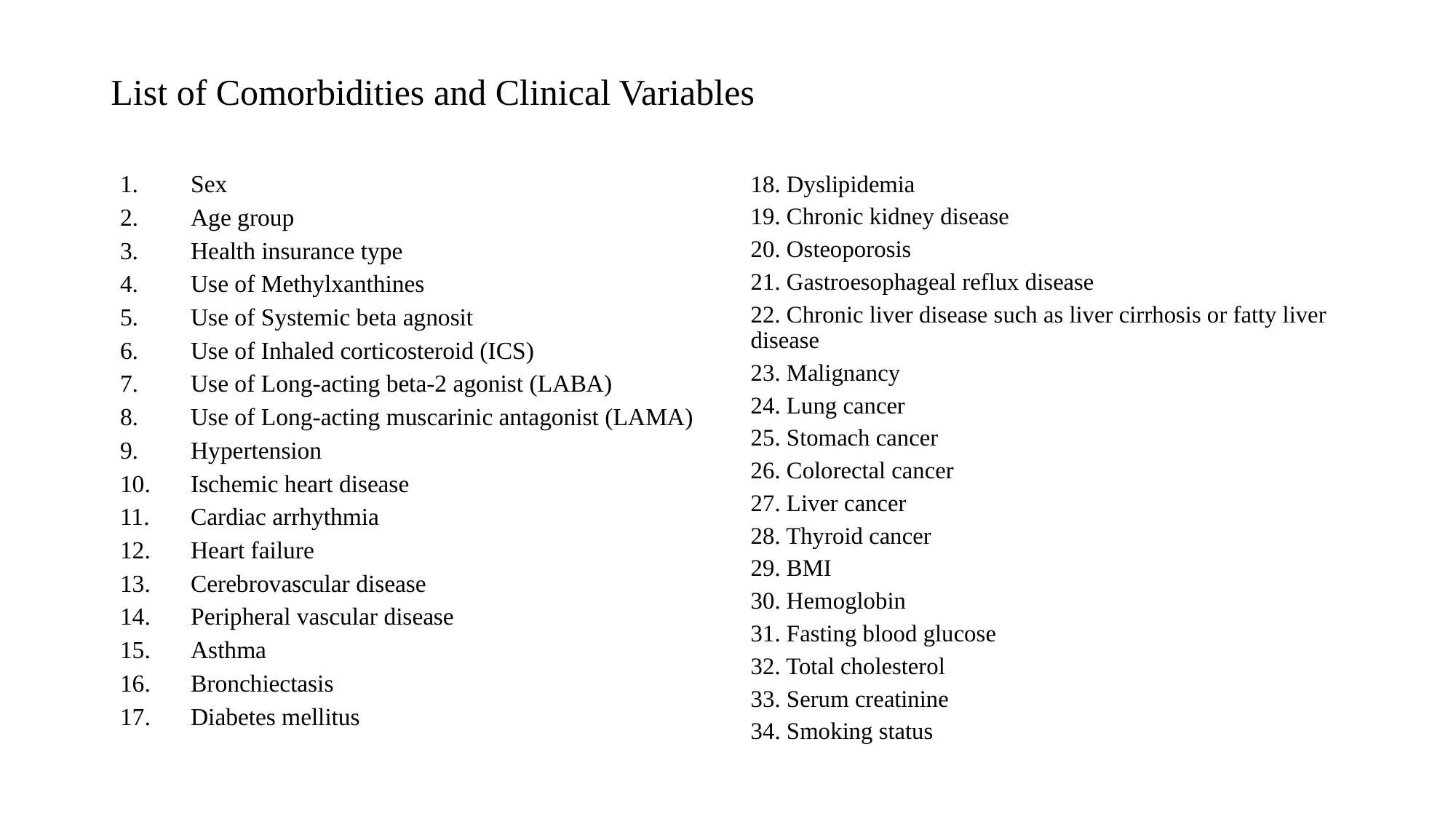

# List of Comorbidities and Clinical Variables
Sex
Age group
Health insurance type
Use of Methylxanthines
Use of Systemic beta agnosit
Use of Inhaled corticosteroid (ICS)
Use of Long-acting beta-2 agonist (LABA)
Use of Long-acting muscarinic antagonist (LAMA)
Hypertension
Ischemic heart disease
Cardiac arrhythmia
Heart failure
Cerebrovascular disease
Peripheral vascular disease
Asthma
Bronchiectasis
Diabetes mellitus
18. Dyslipidemia
19. Chronic kidney disease
20. Osteoporosis
21. Gastroesophageal reflux disease
22. Chronic liver disease such as liver cirrhosis or fatty liver disease
23. Malignancy
24. Lung cancer
25. Stomach cancer
26. Colorectal cancer
27. Liver cancer
28. Thyroid cancer
29. BMI
30. Hemoglobin
31. Fasting blood glucose
32. Total cholesterol
33. Serum creatinine
34. Smoking status

## Slide 33
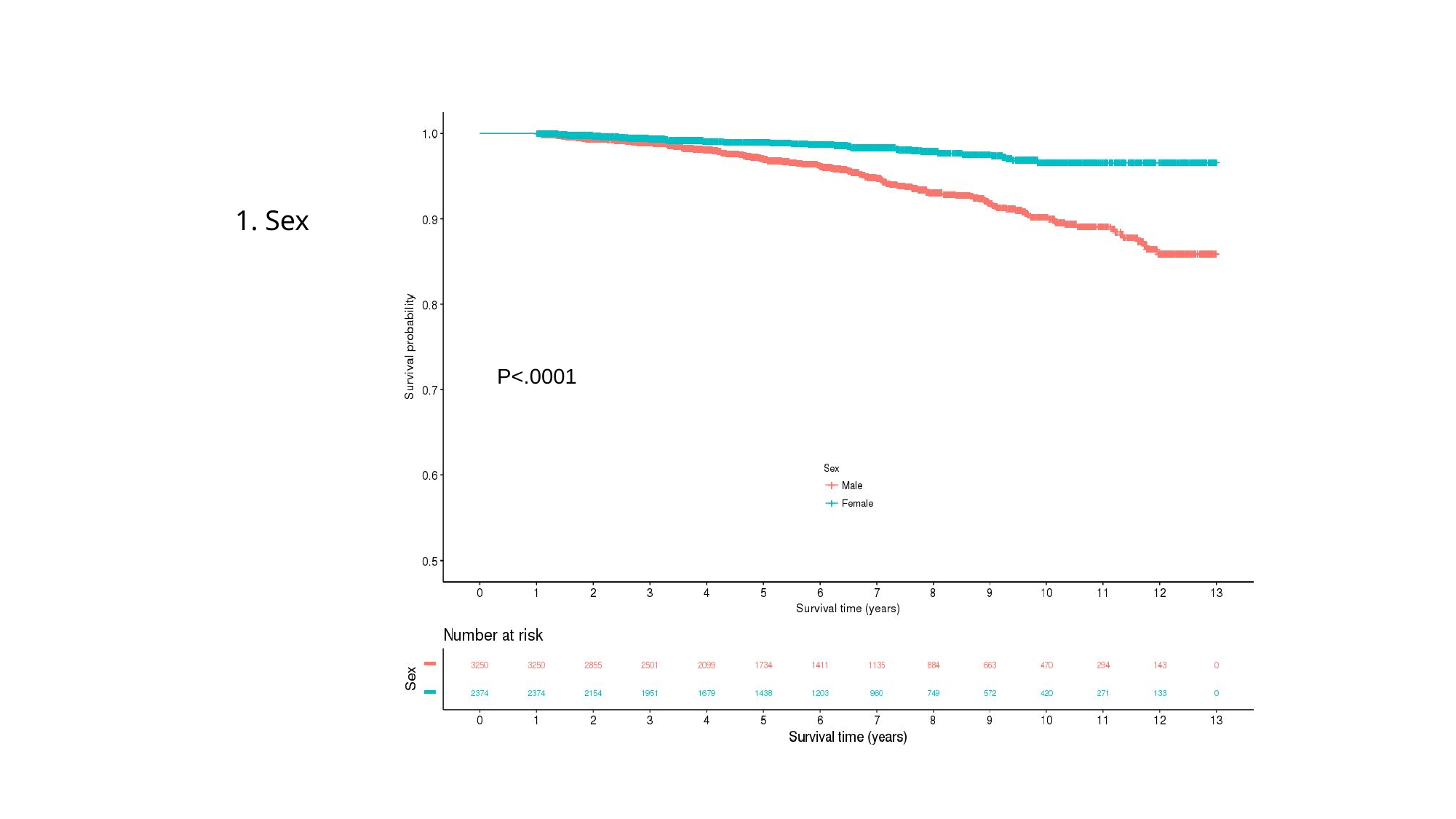

P<.0001
1. Sex

## Slide 34
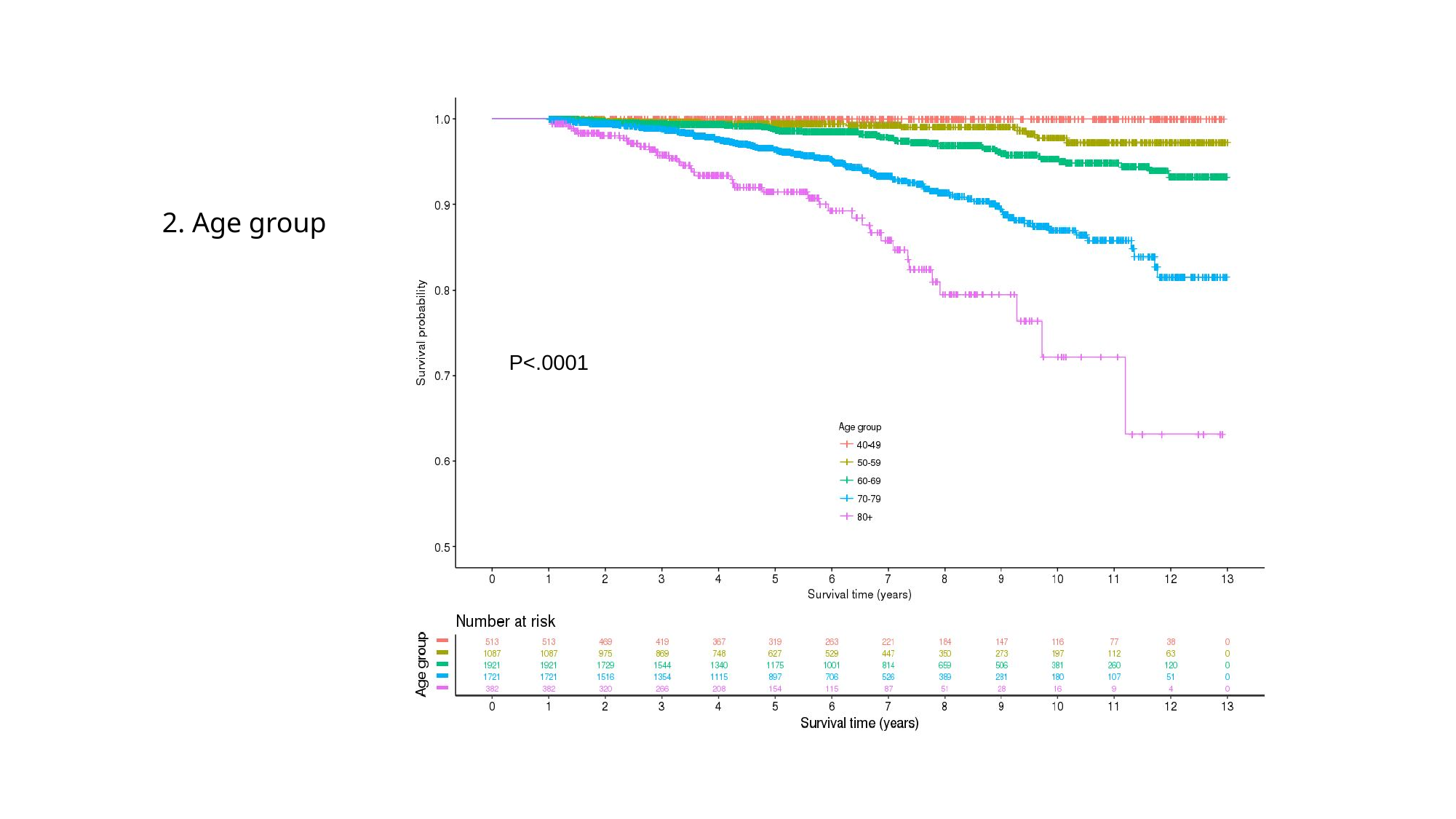

P<.0001
2. Age group

## Slide 35
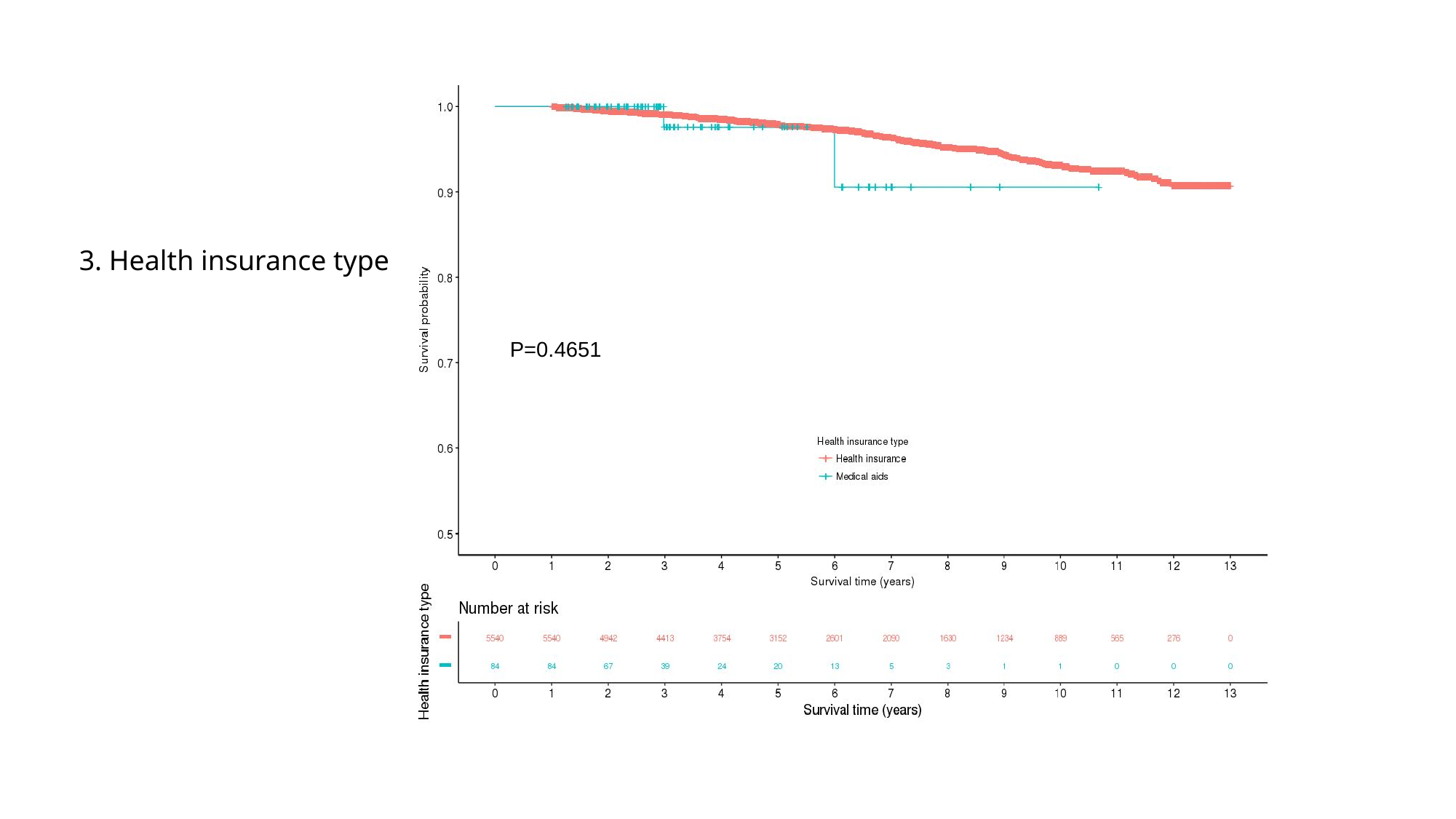

P=0.4651
3. Health insurance type

## Slide 36
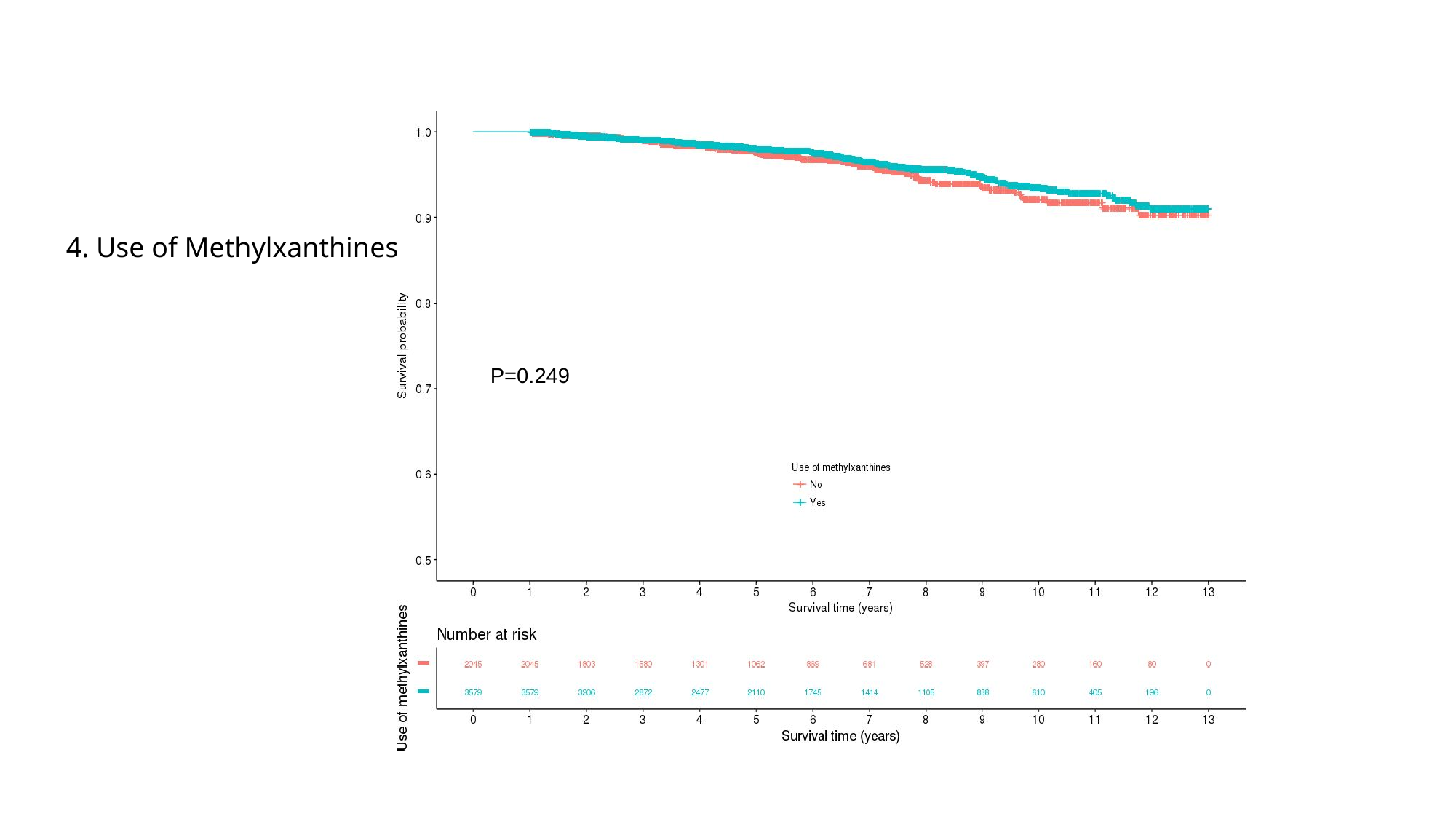

P=0.249
4. Use of Methylxanthines

## Slide 37
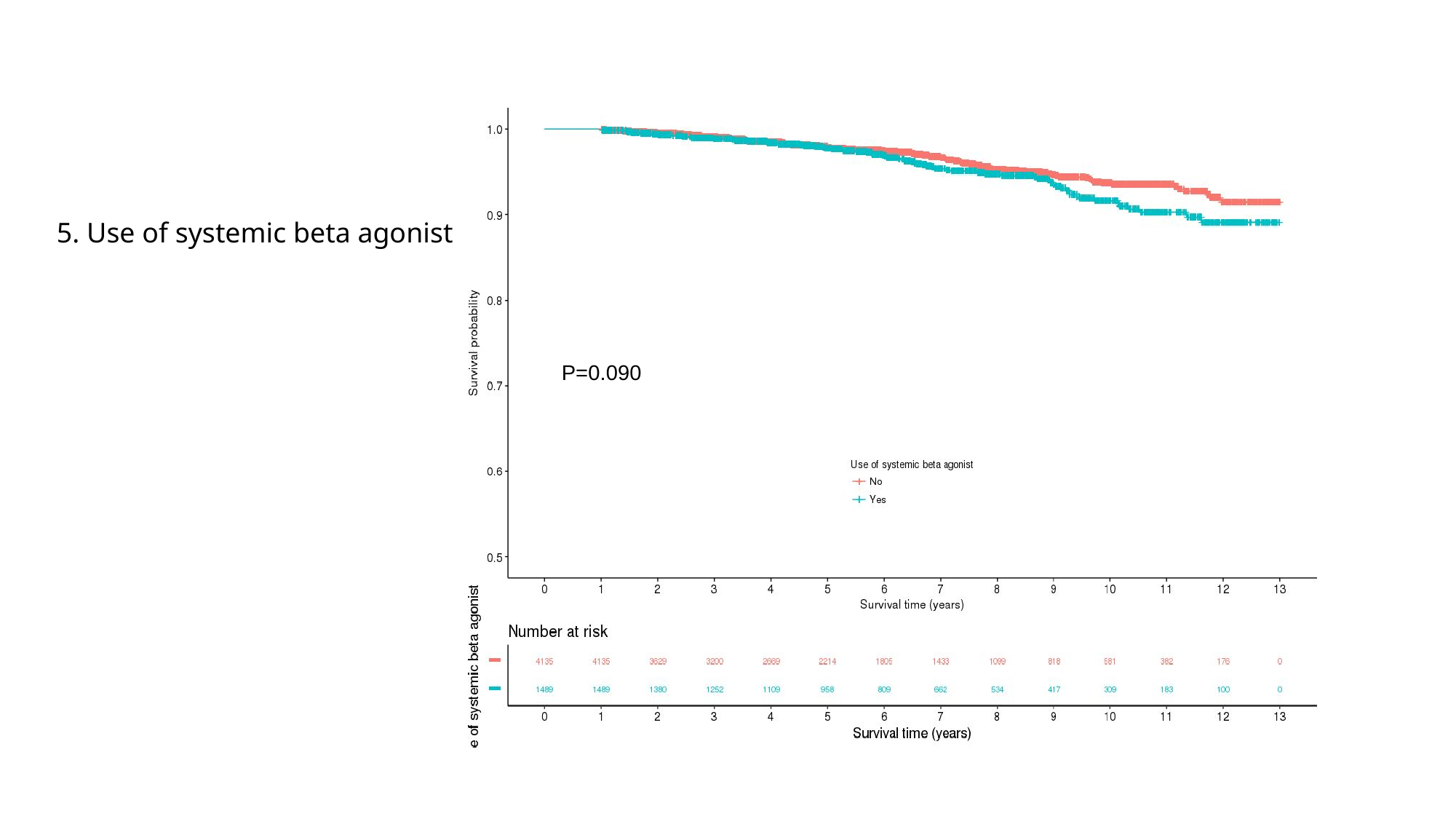

P=0.090
5. Use of systemic beta agonist

## Slide 38
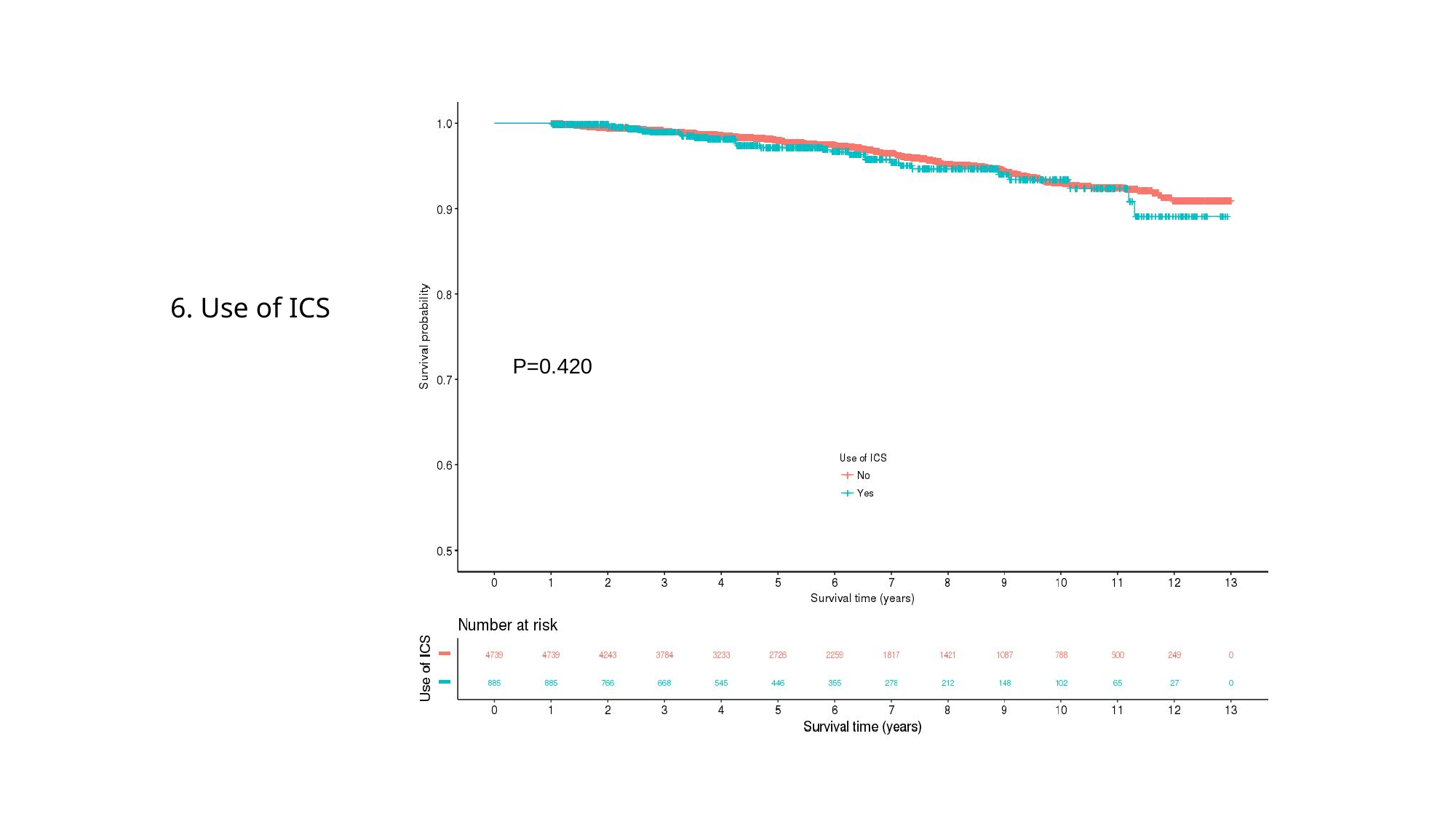

P=0.420
6. Use of ICS

## Slide 39
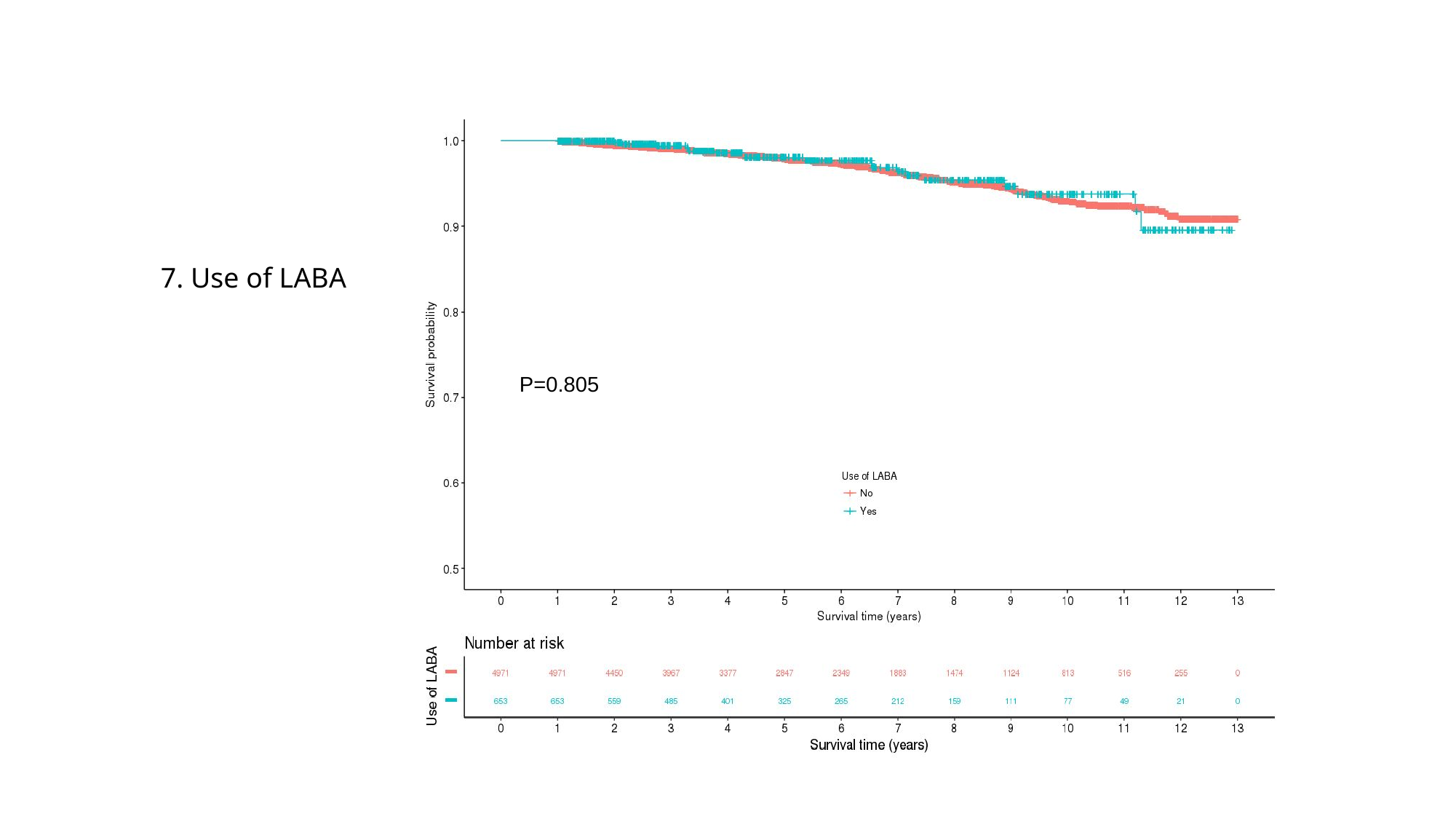

P=0.805
7. Use of LABA

## Slide 40
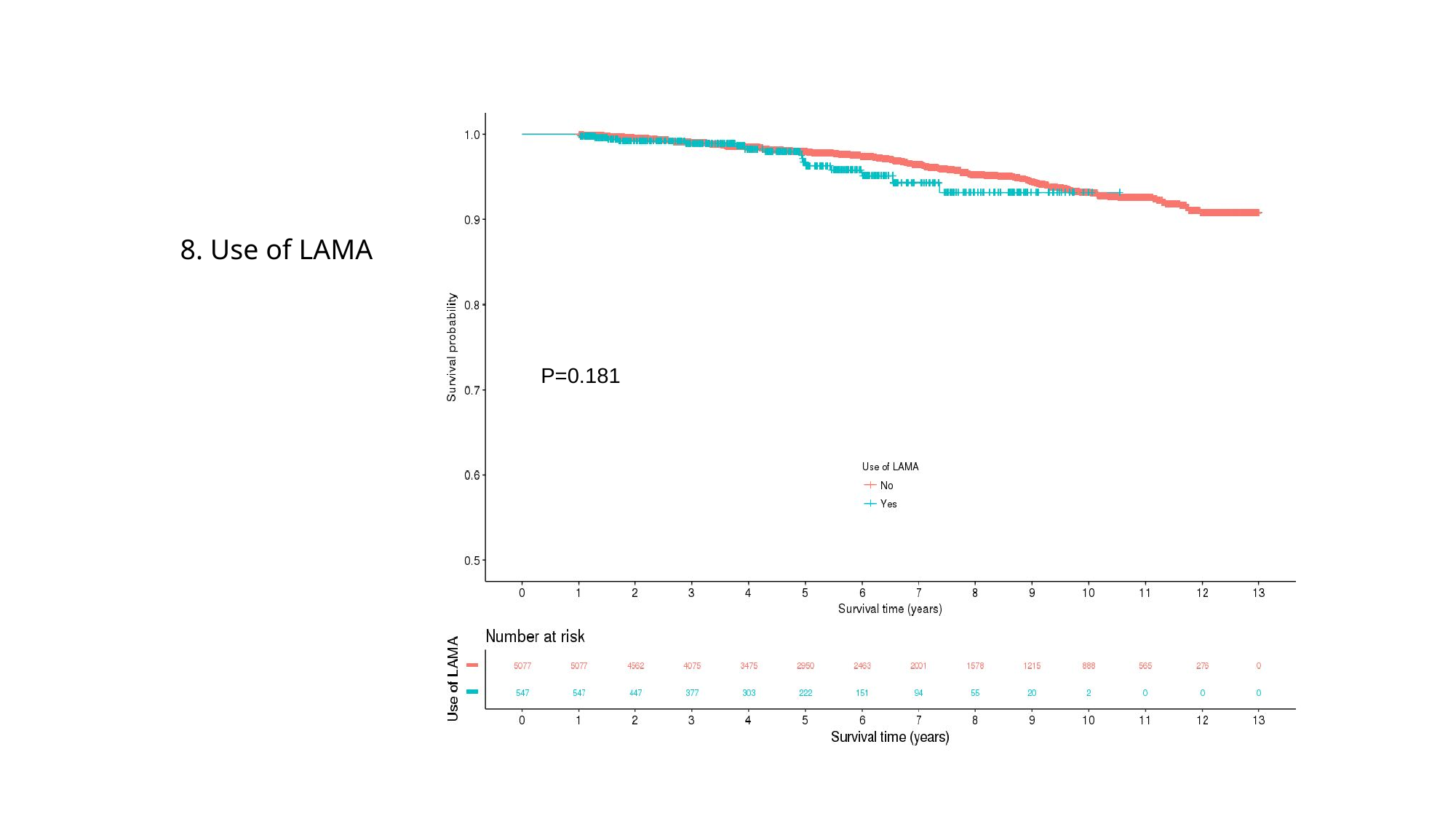

P=0.181
8. Use of LAMA

## Slide 41
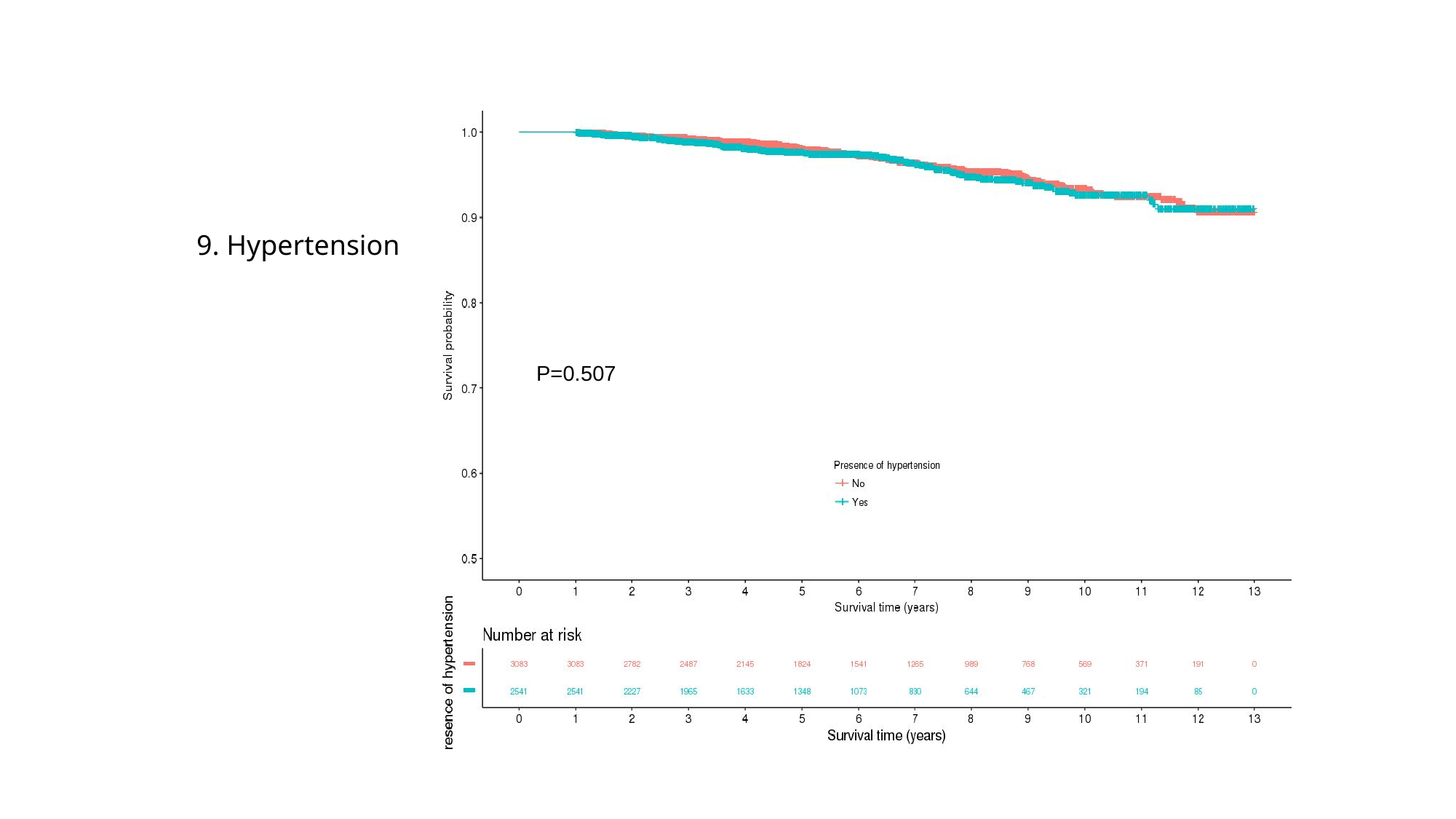

P=0.507
9. Hypertension

## Slide 42
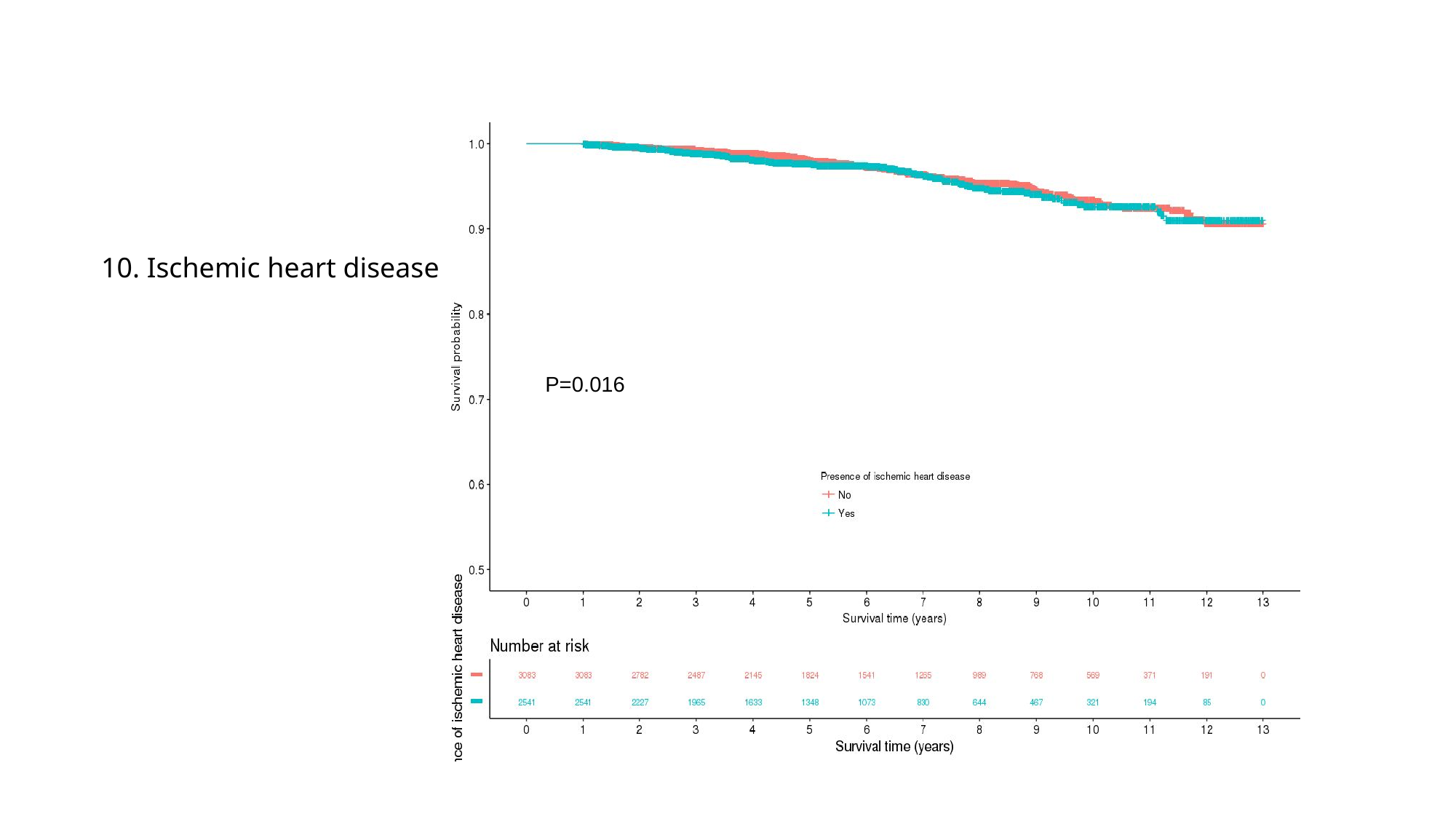

P=0.016
10. Ischemic heart disease

## Slide 43
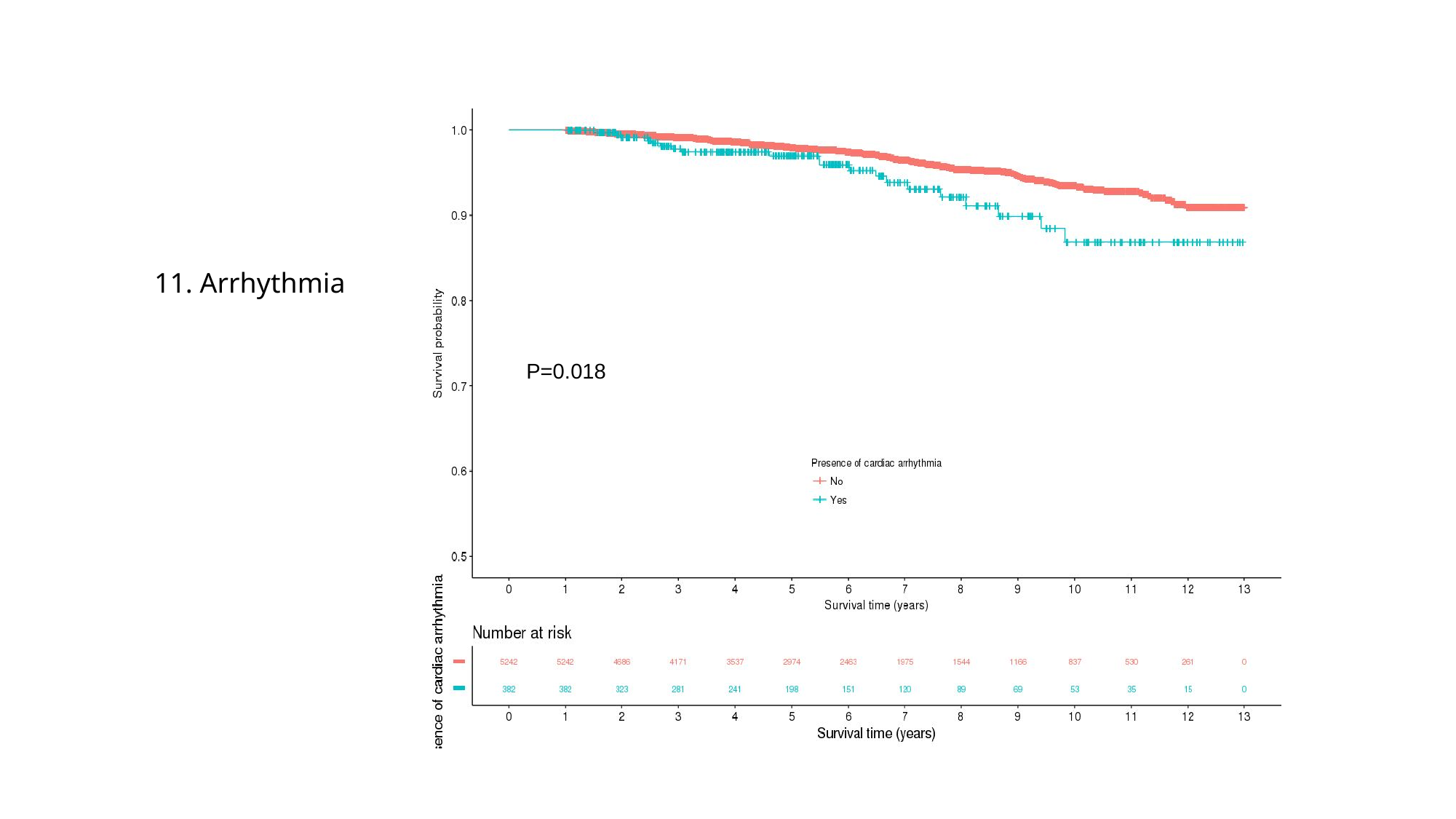

P=0.018
11. Arrhythmia

## Slide 44
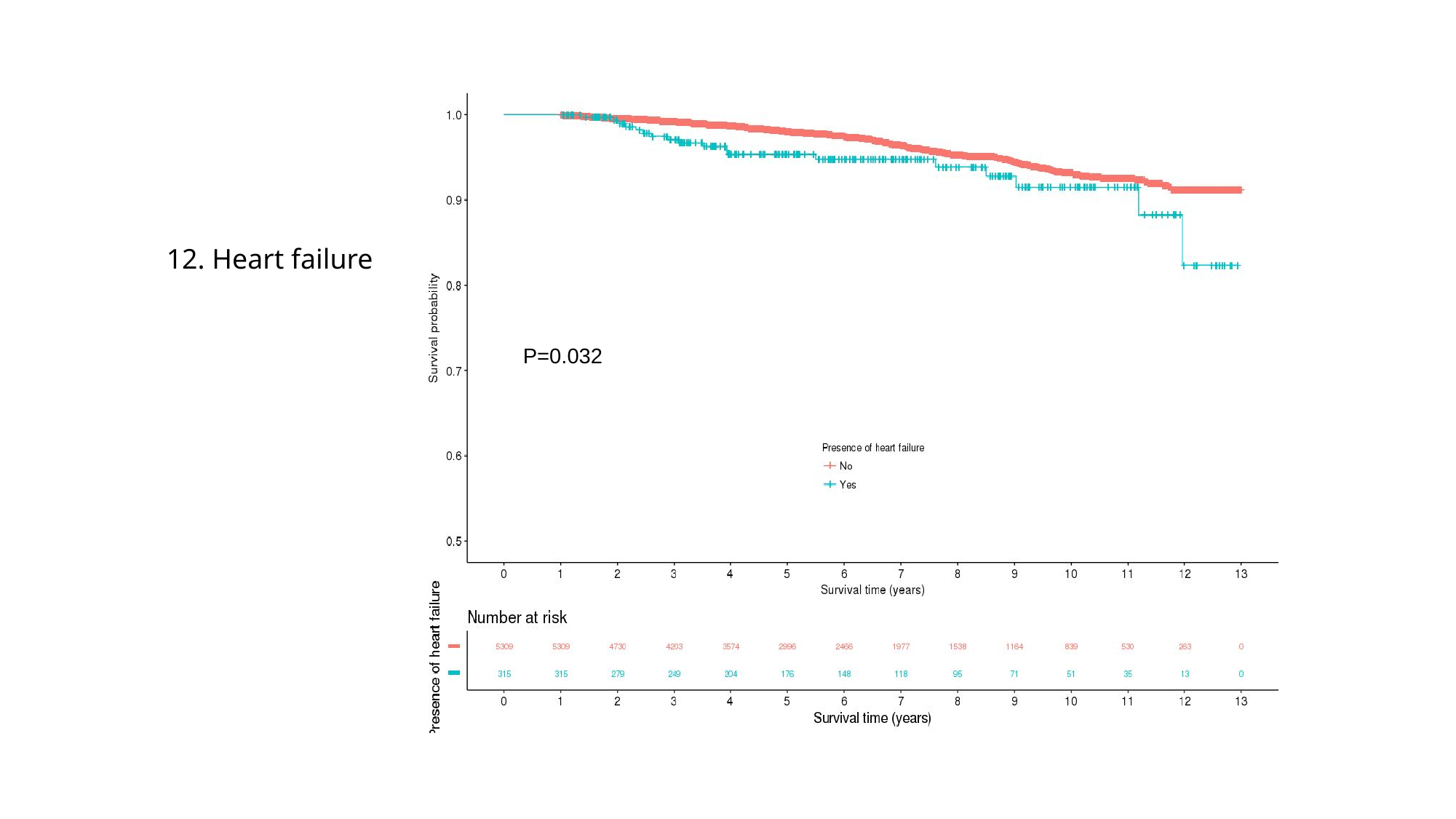

P=0.032
12. Heart failure

## Slide 45
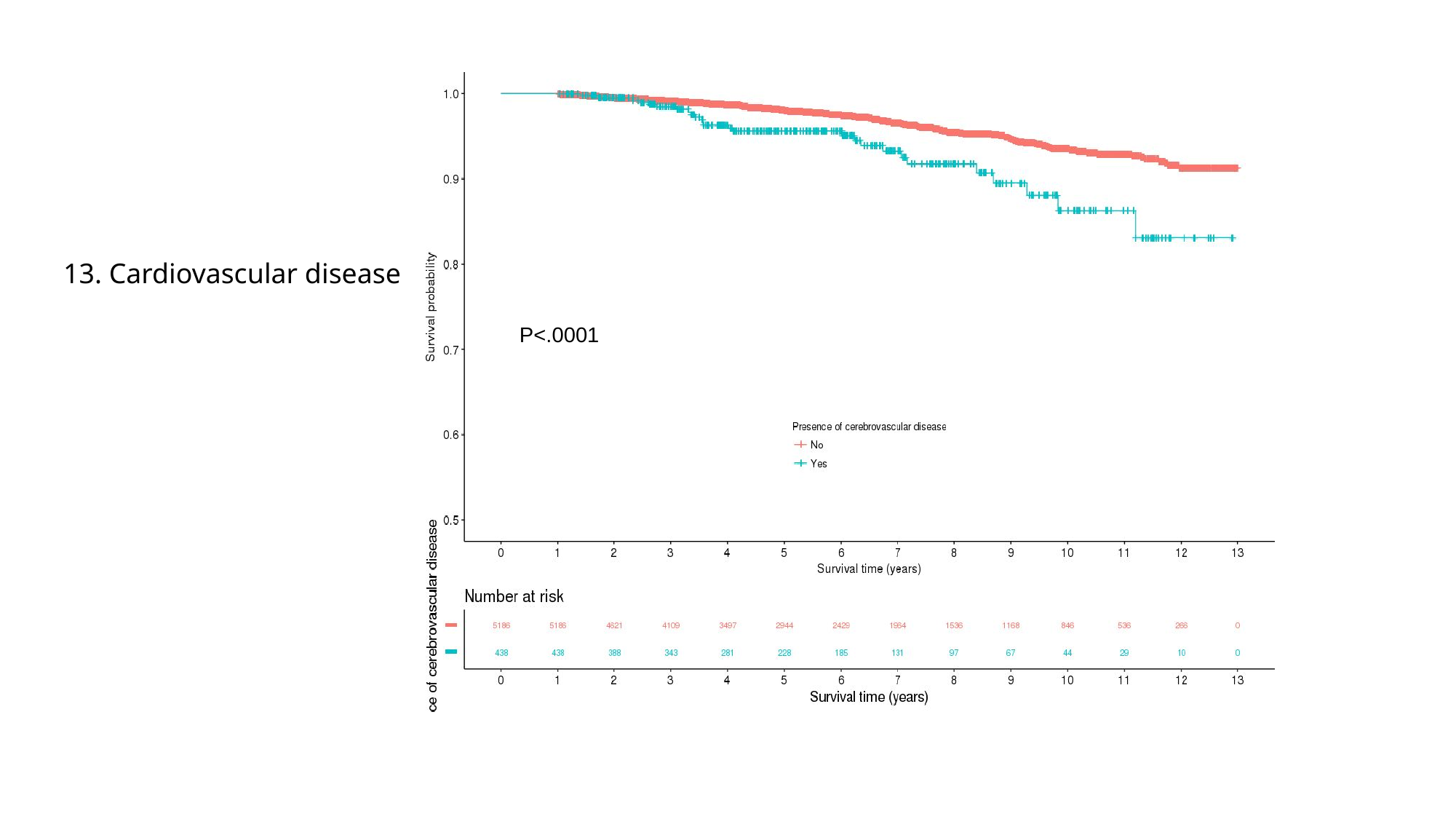

P<.0001
13. Cardiovascular disease

## Slide 46
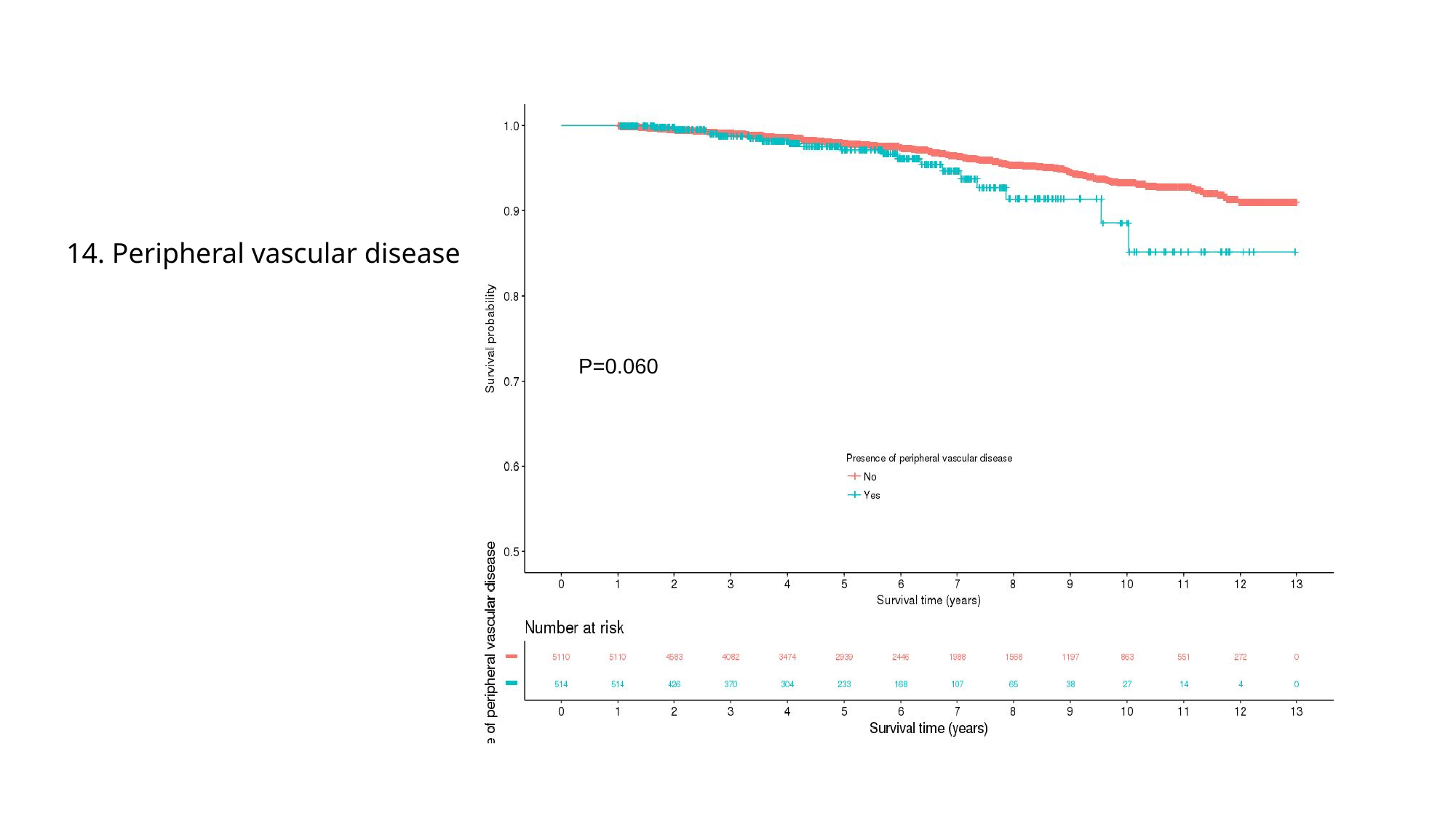

P=0.060
14. Peripheral vascular disease

## Slide 47
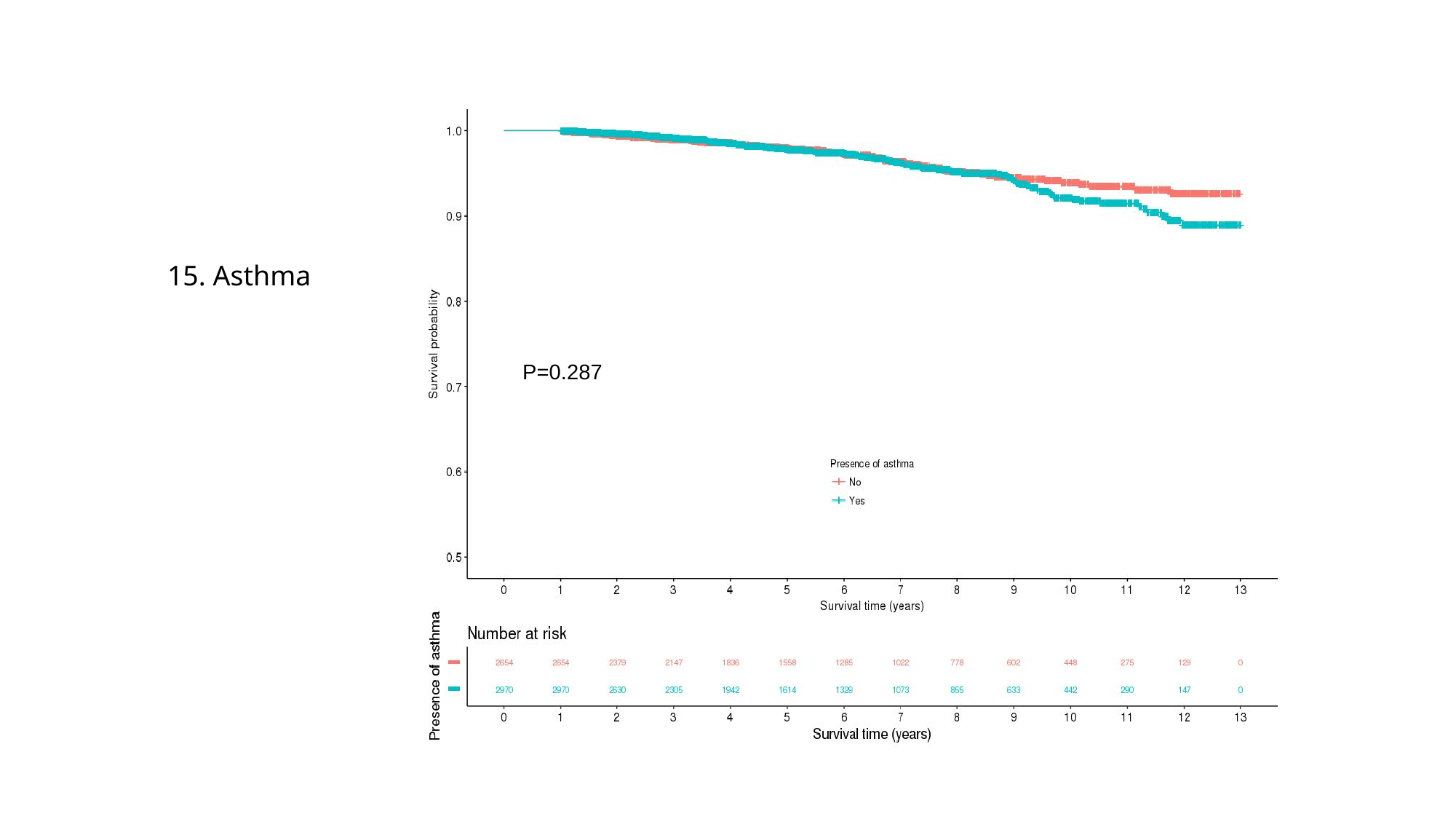

P=0.287
15. Asthma

## Slide 48
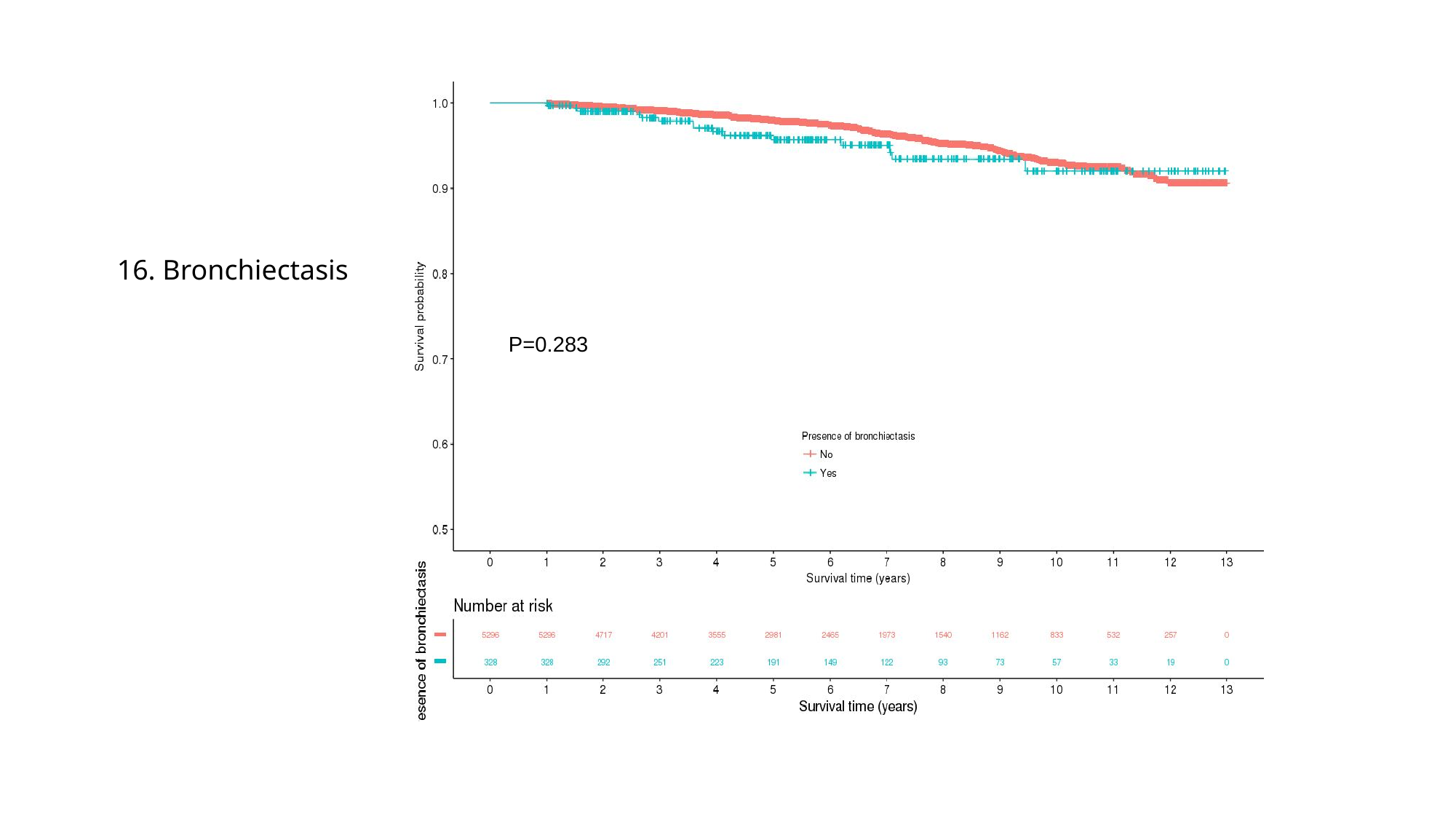

P=0.283
16. Bronchiectasis

## Slide 49
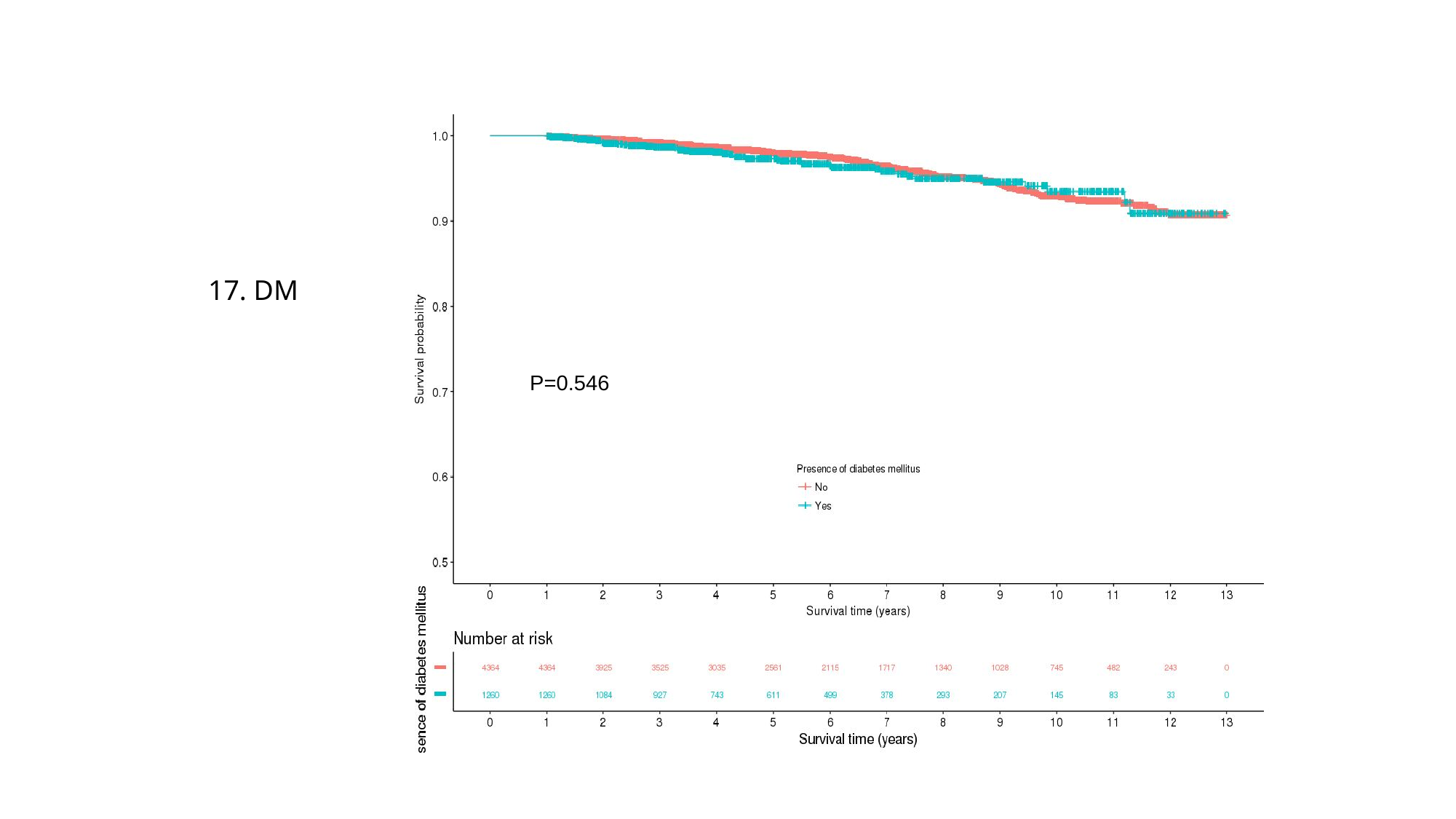

P=0.546
17. DM

## Slide 50
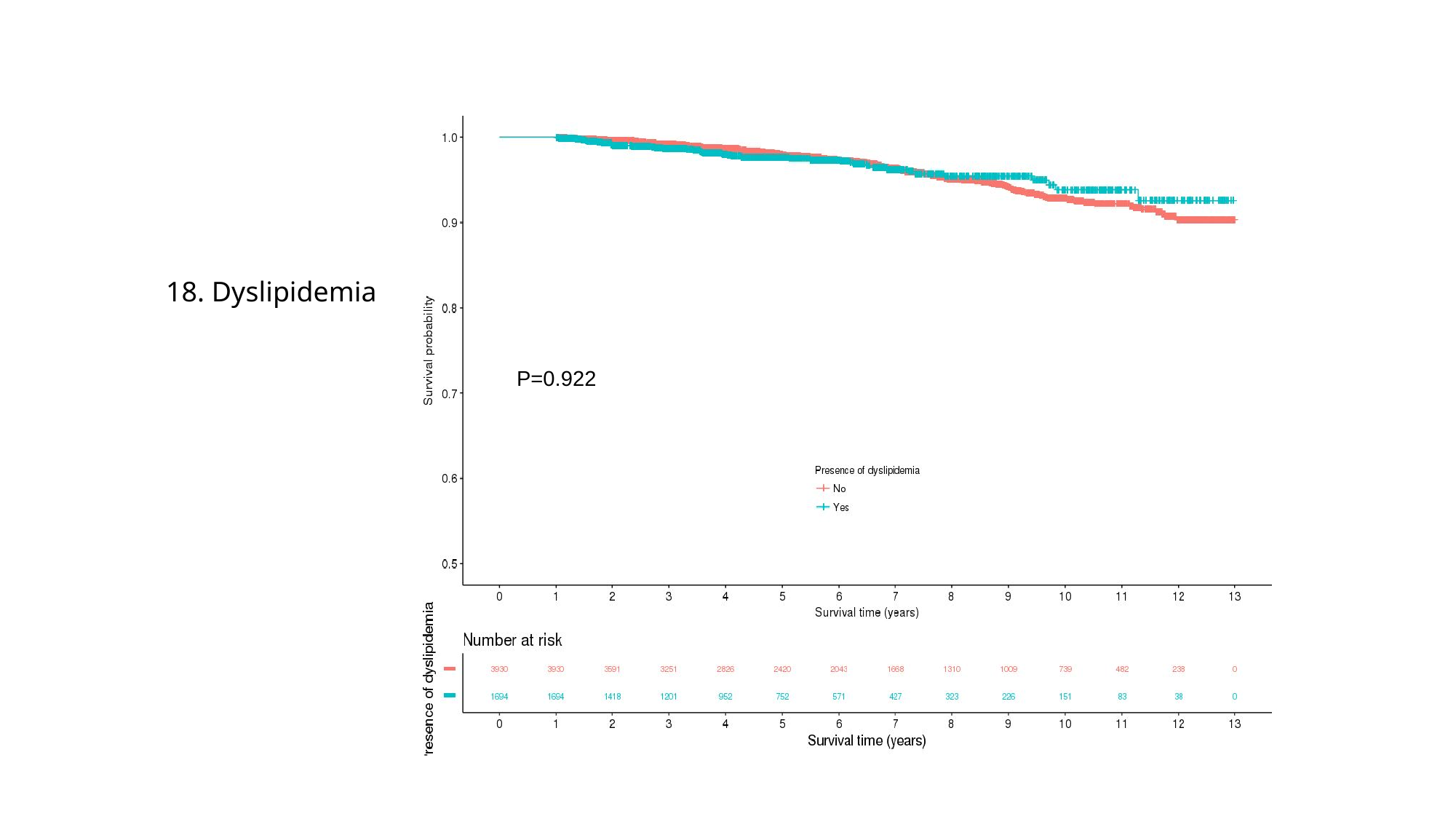

P=0.922
18. Dyslipidemia

## Slide 51
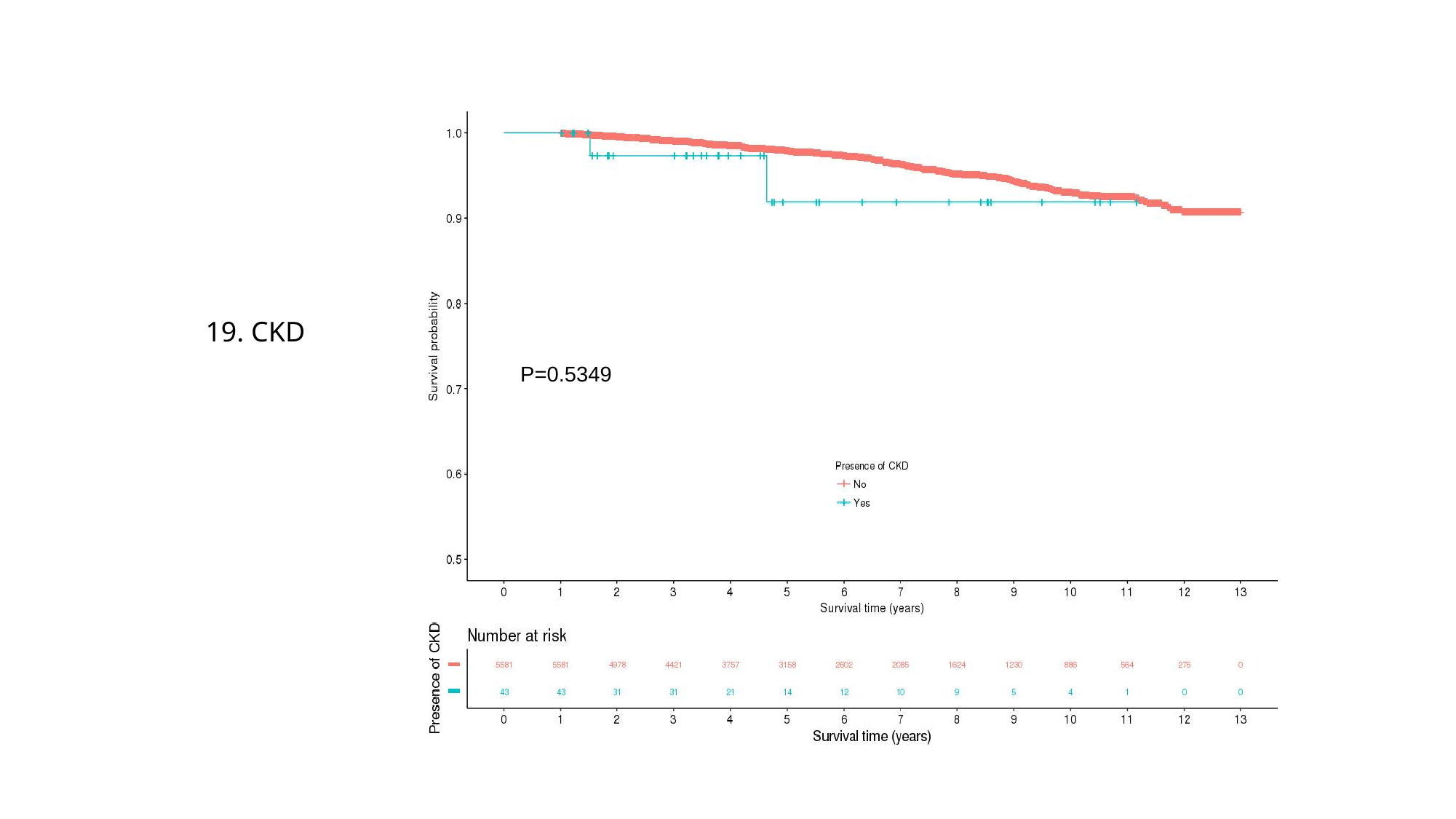

P=0.5349
19. CKD

## Slide 52
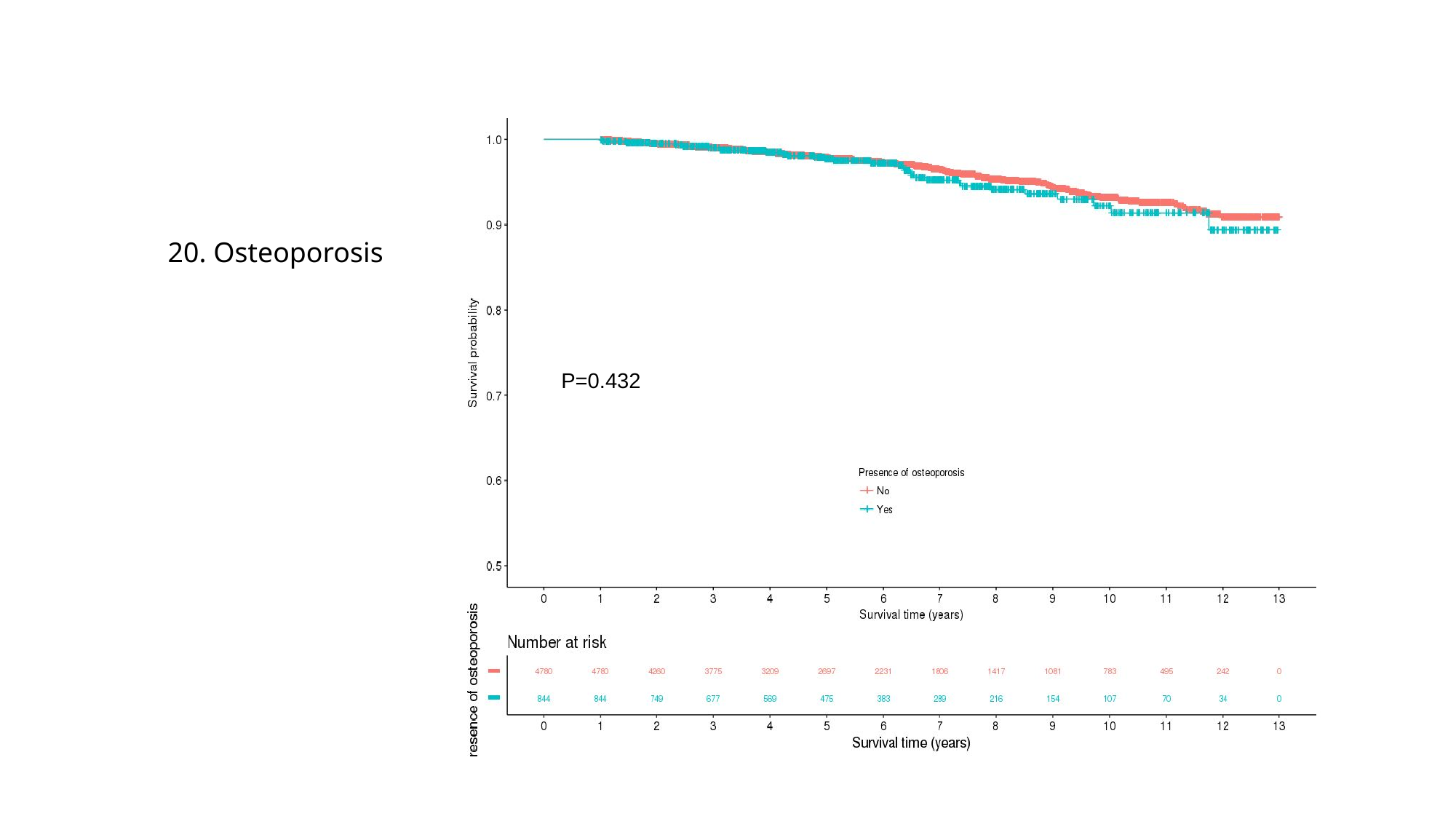

P=0.432
20. Osteoporosis

## Slide 53
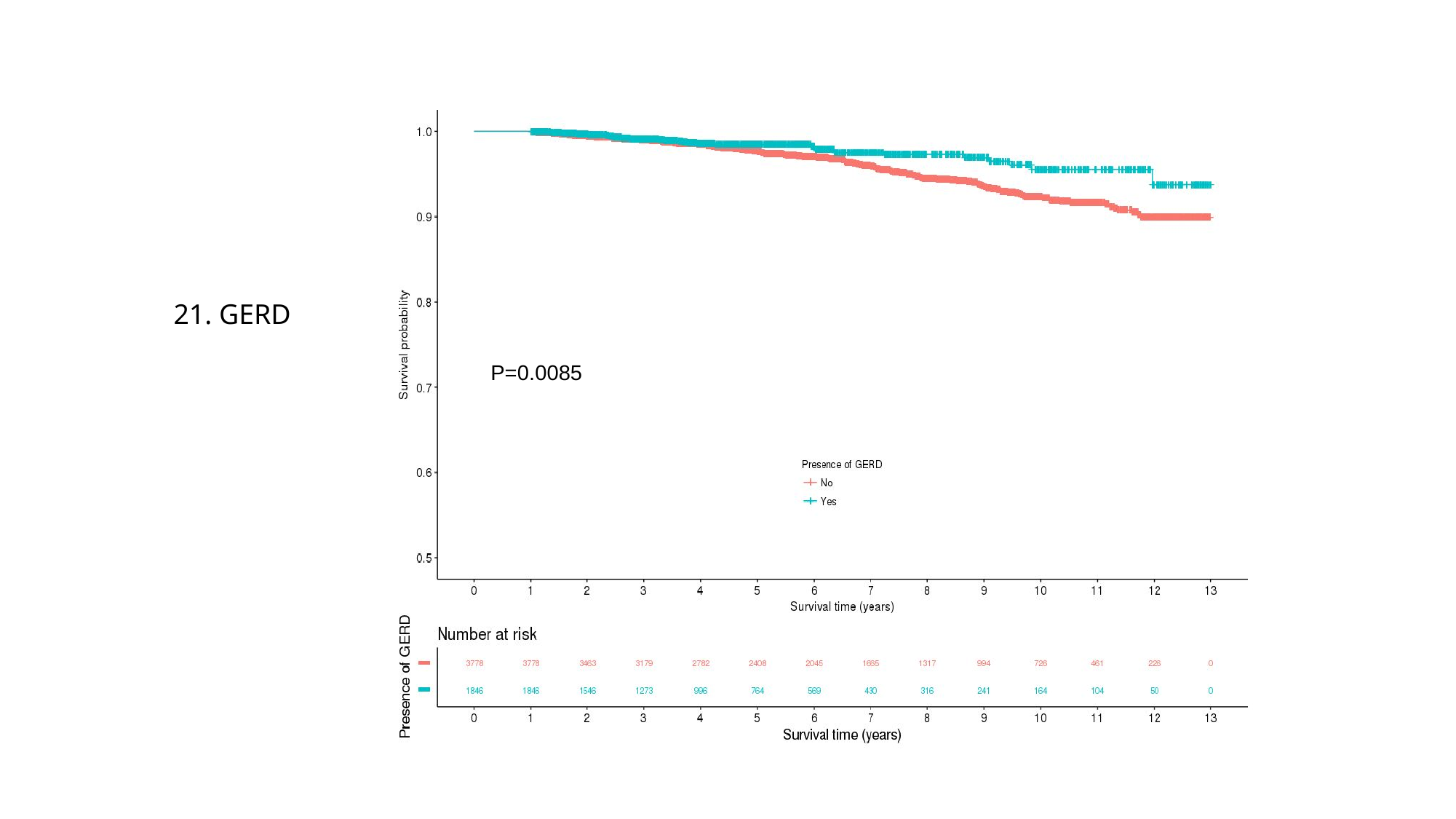

P=0.0085
21. GERD

## Slide 54
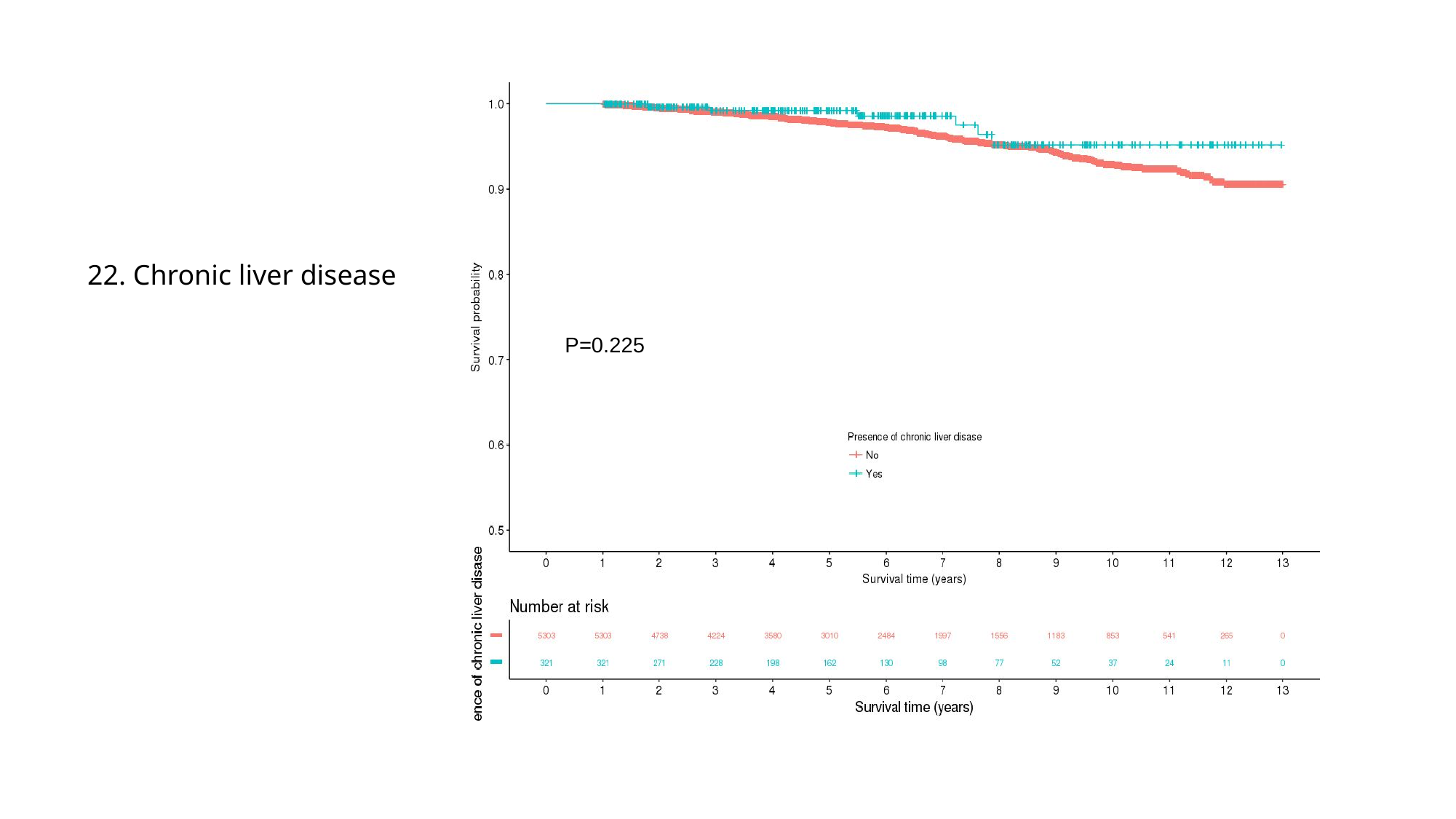

P=0.225
22. Chronic liver disease

## Slide 55
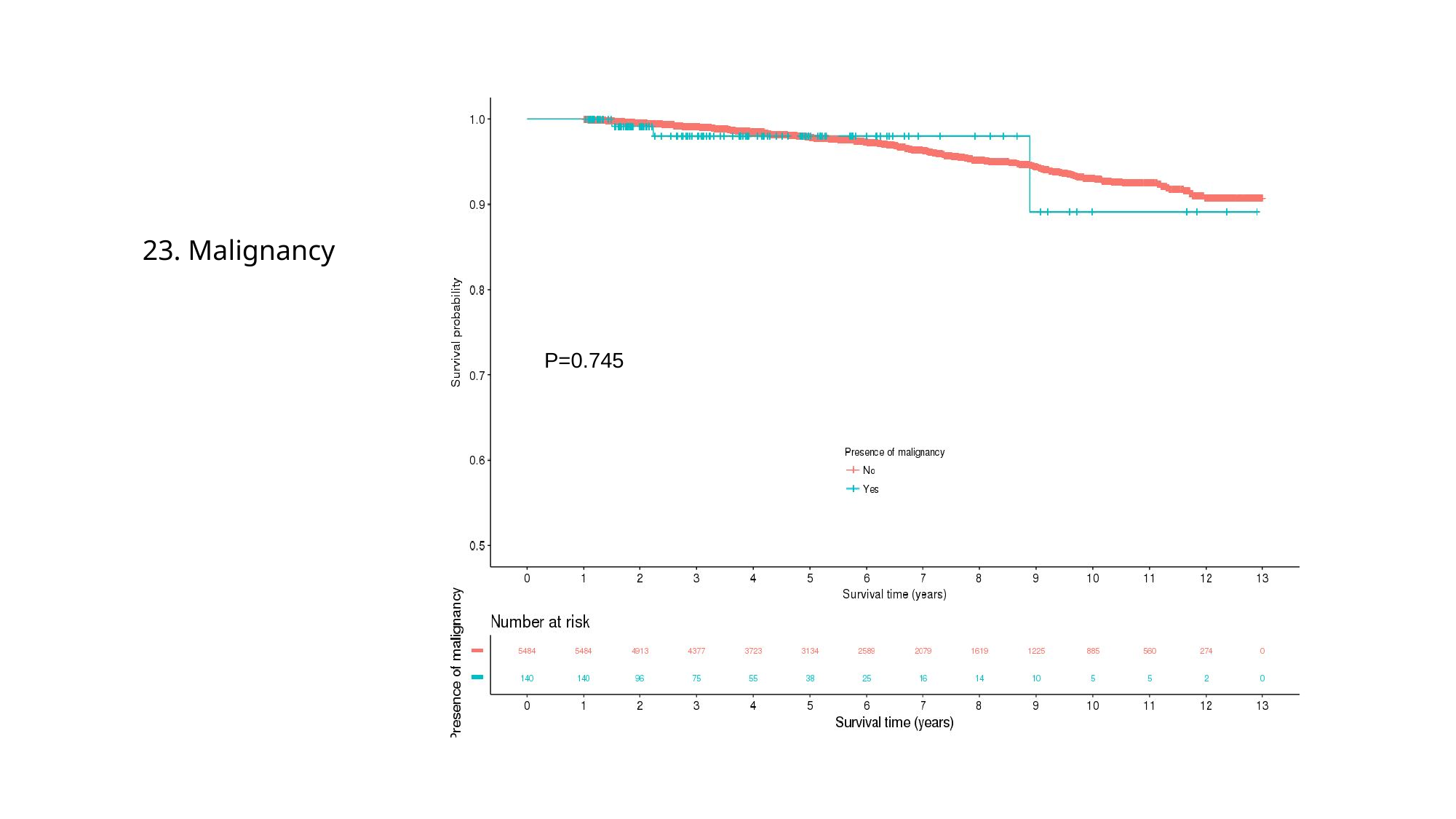

P=0.745
23. Malignancy

## Slide 56
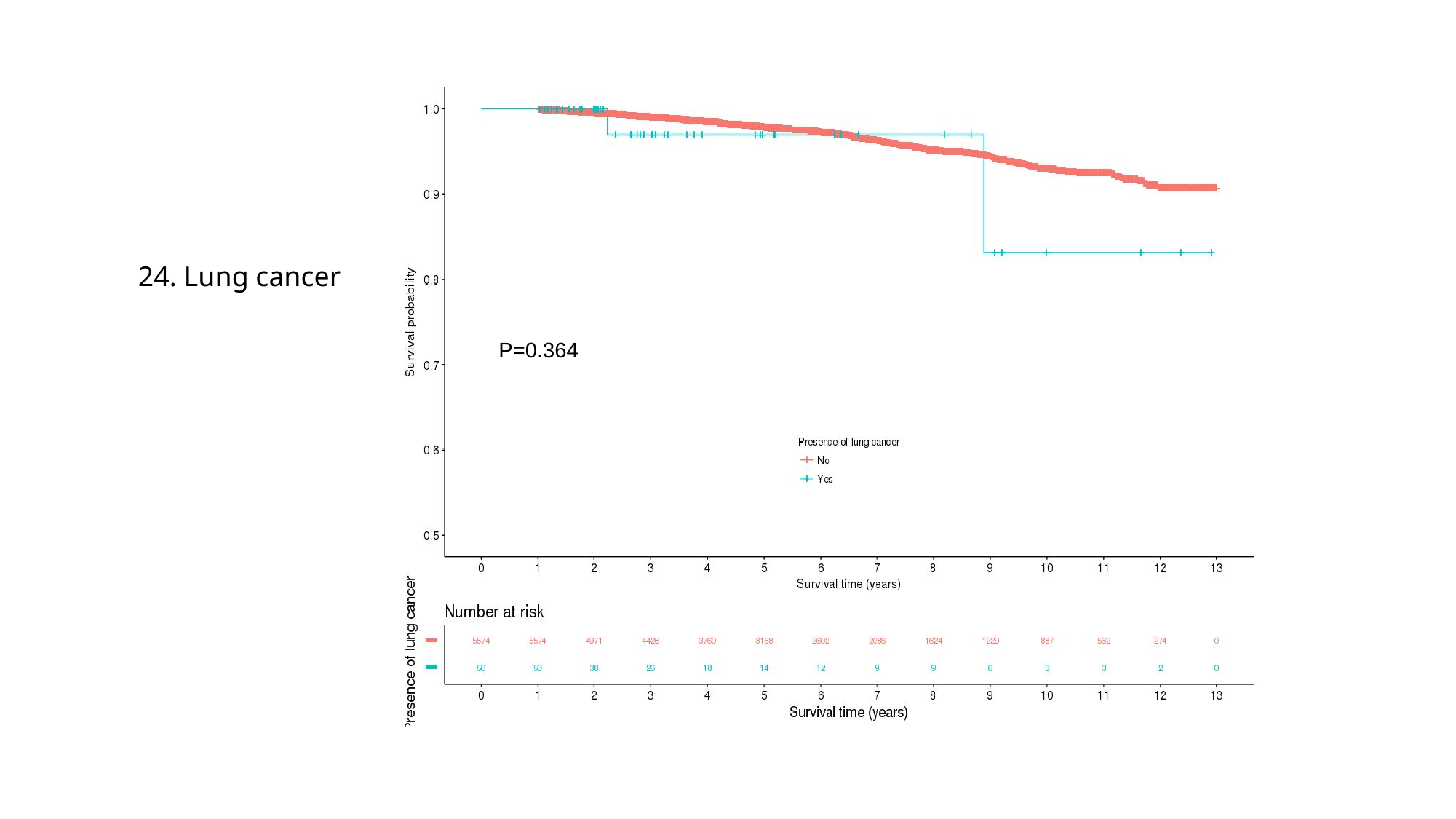

P=0.364
24. Lung cancer

## Slide 57
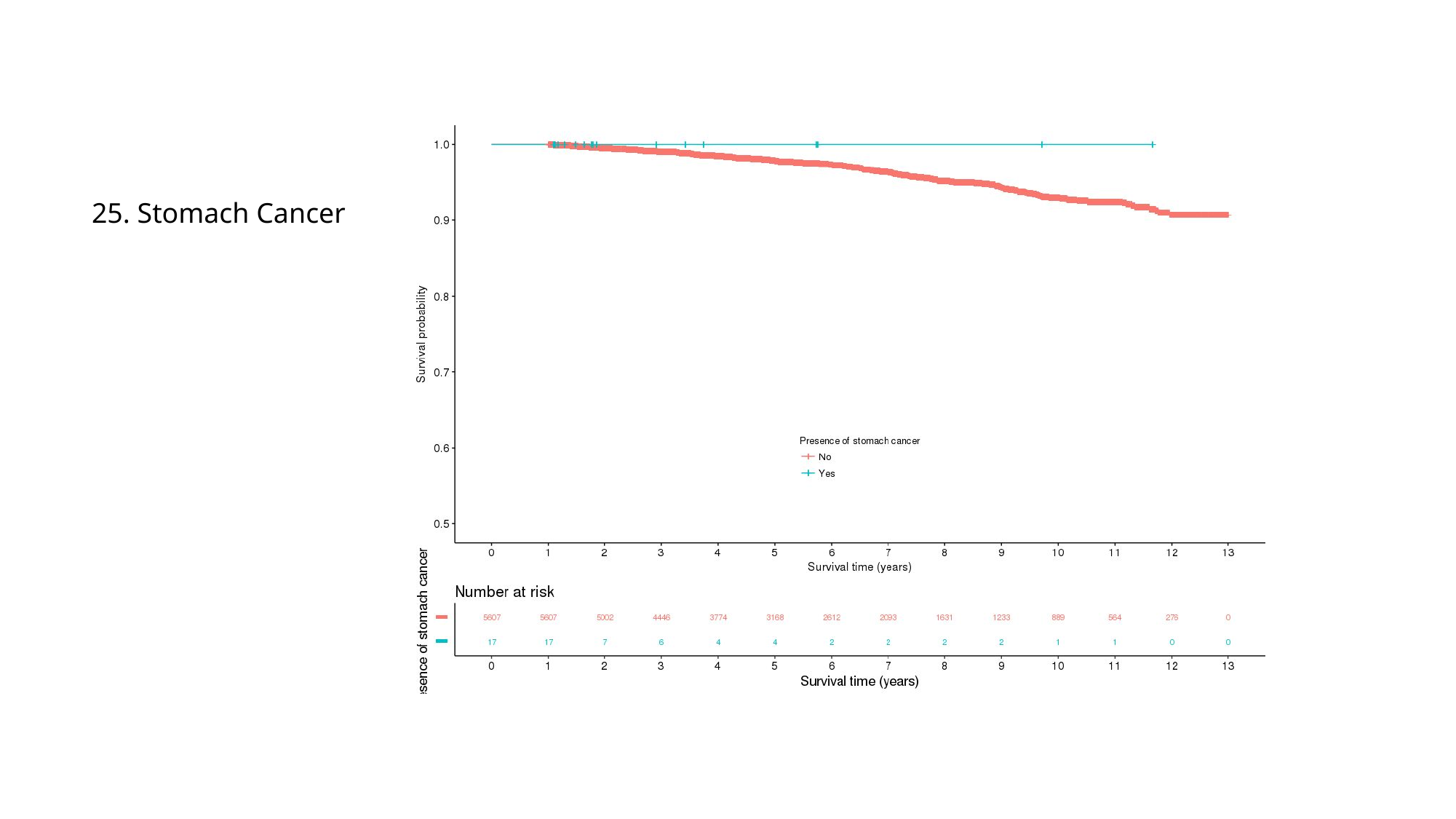

25. Stomach Cancer

## Slide 58
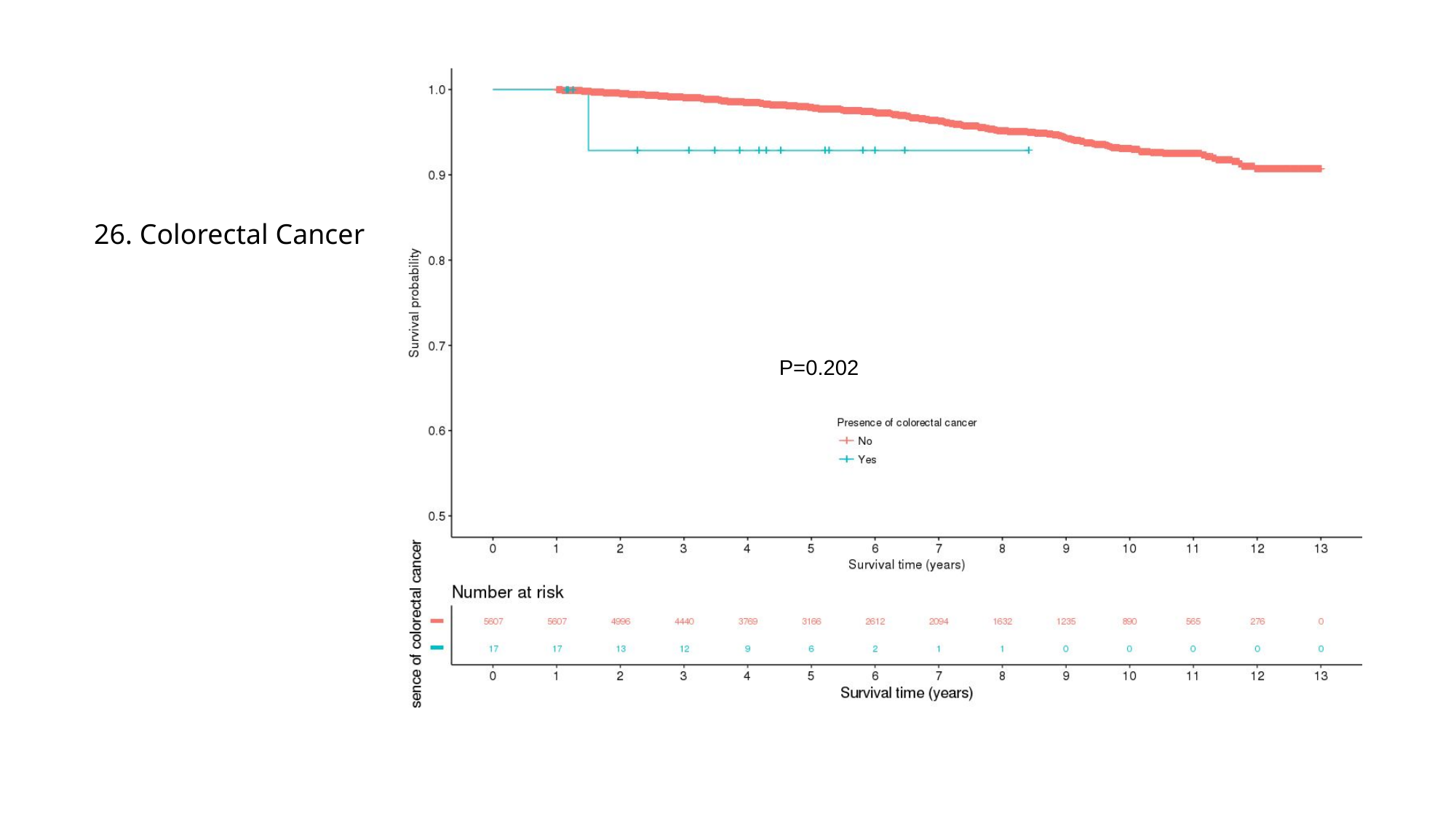

P=0.202
26. Colorectal Cancer

## Slide 59
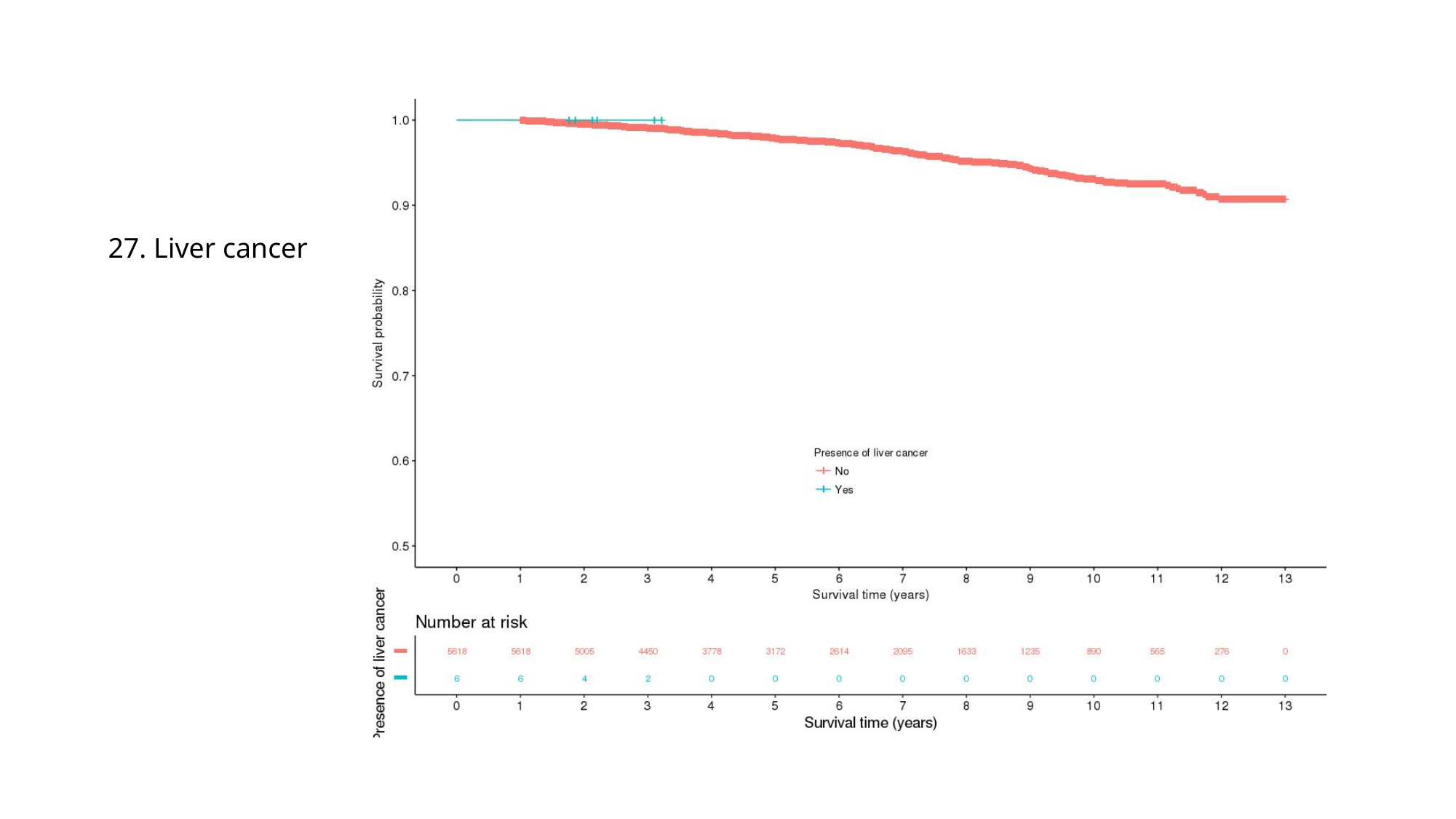

27. Liver cancer

## Slide 60
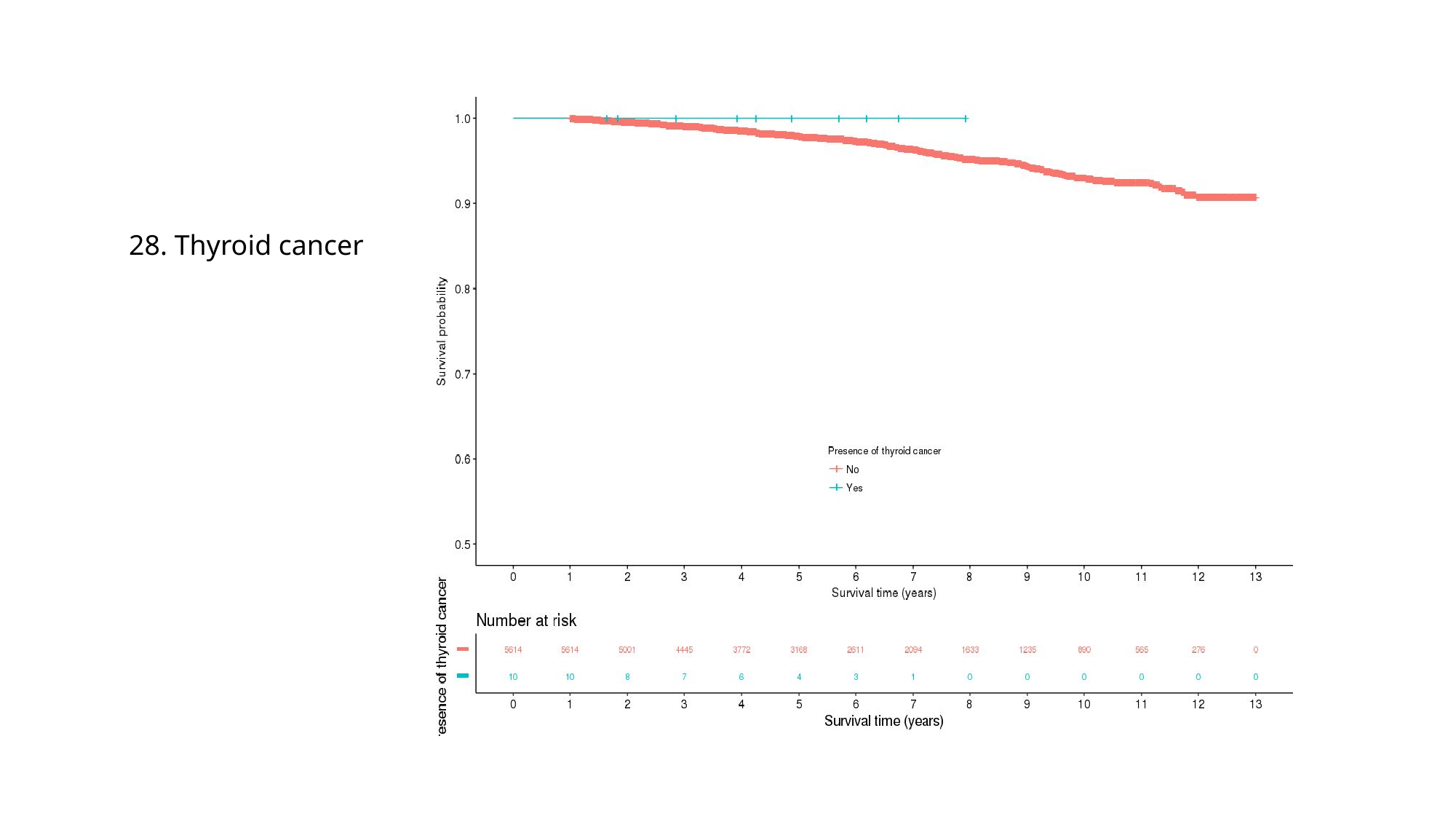

28. Thyroid cancer

## Slide 61
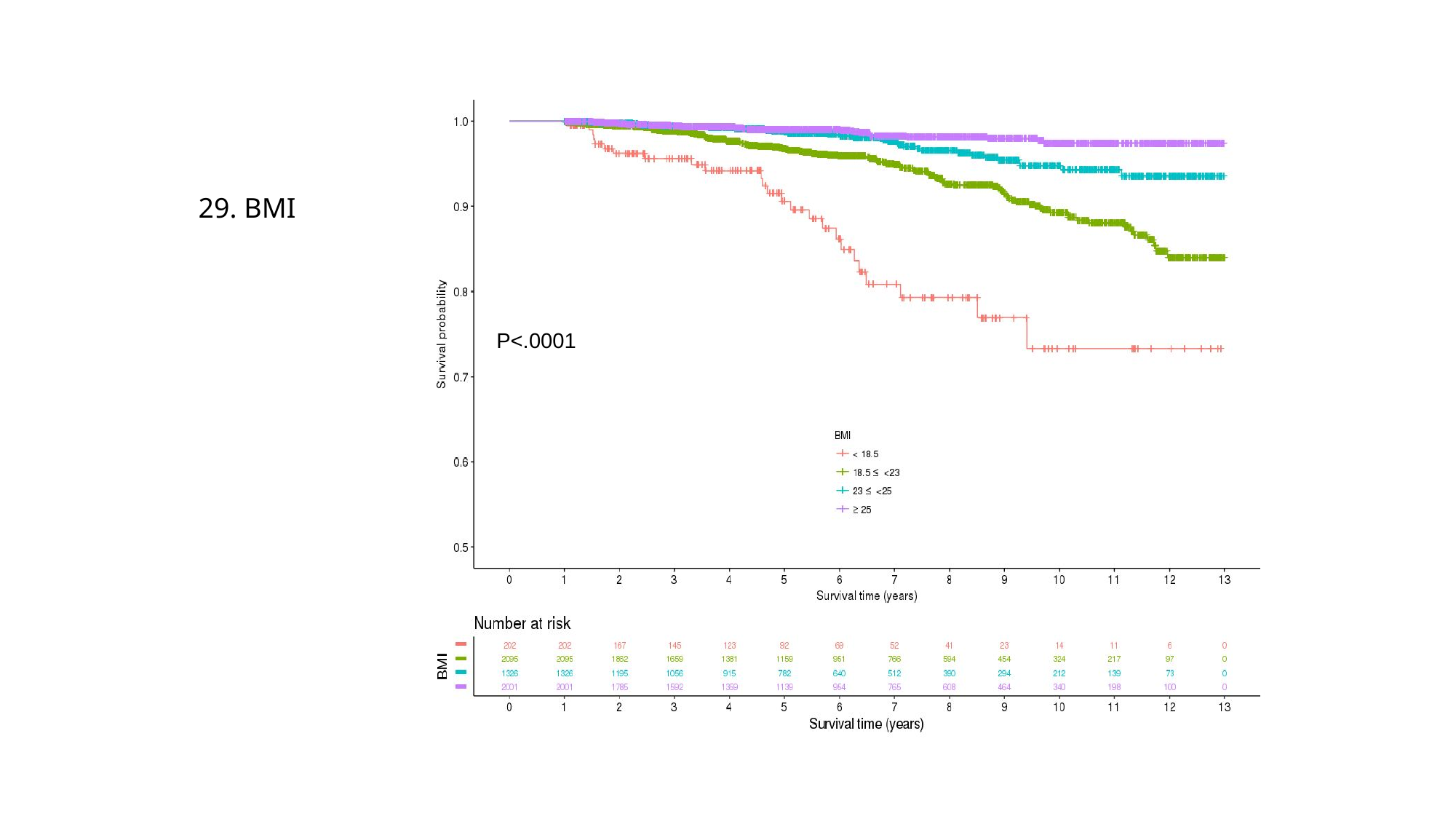

P<.0001
29. BMI

## Slide 62
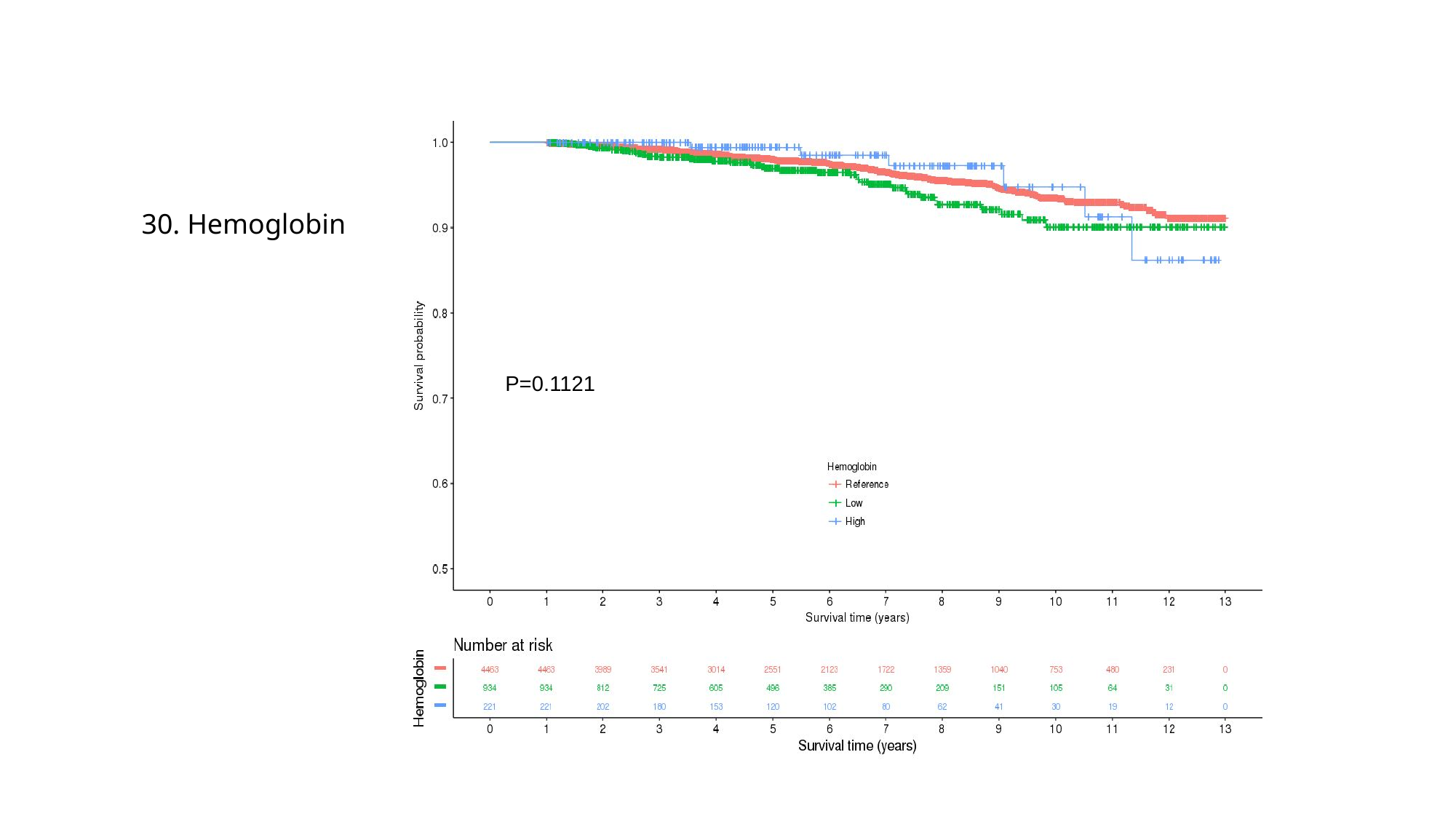

P=0.1121
30. Hemoglobin

## Slide 63
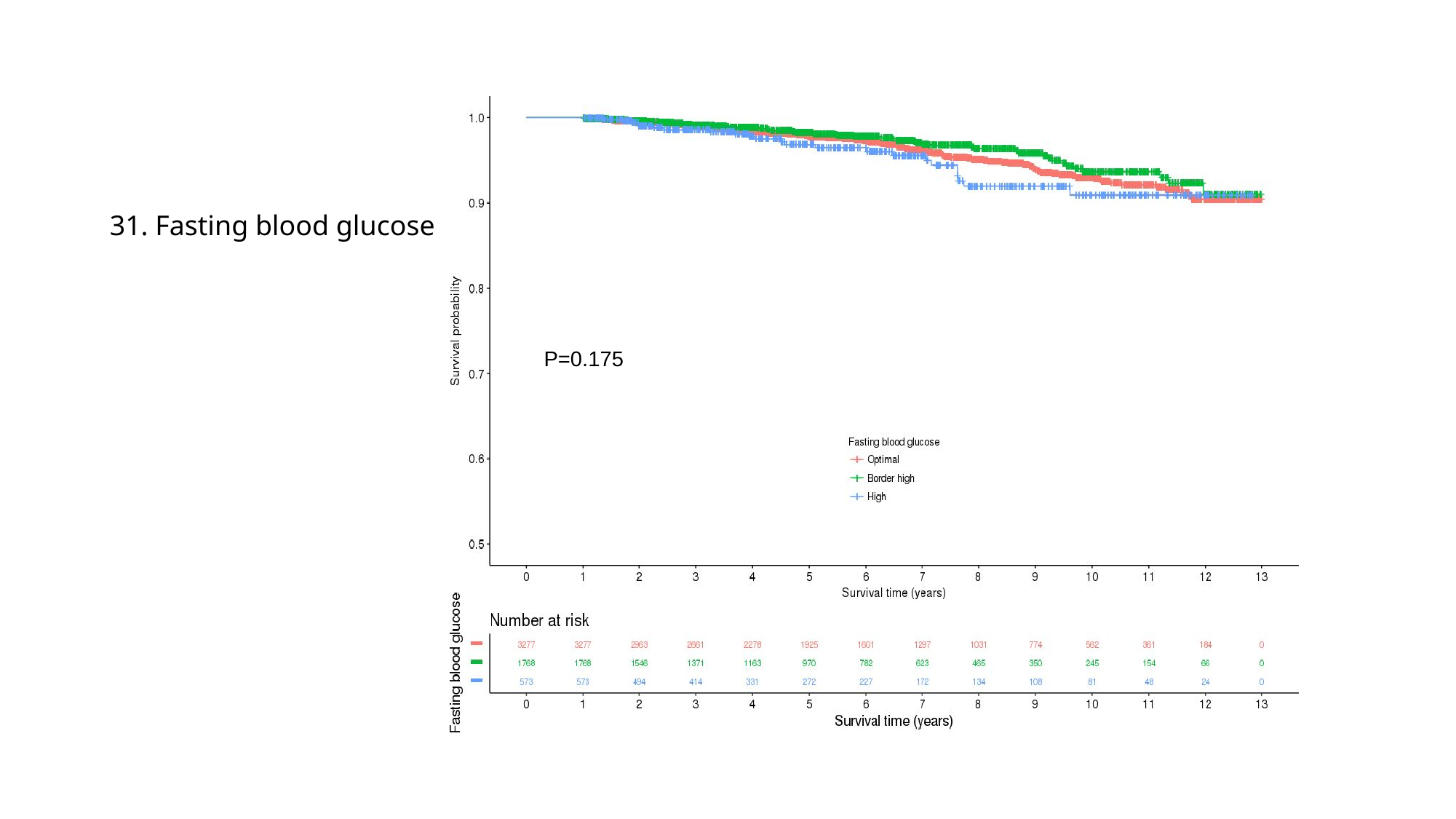

P=0.175
31. Fasting blood glucose

## Slide 64
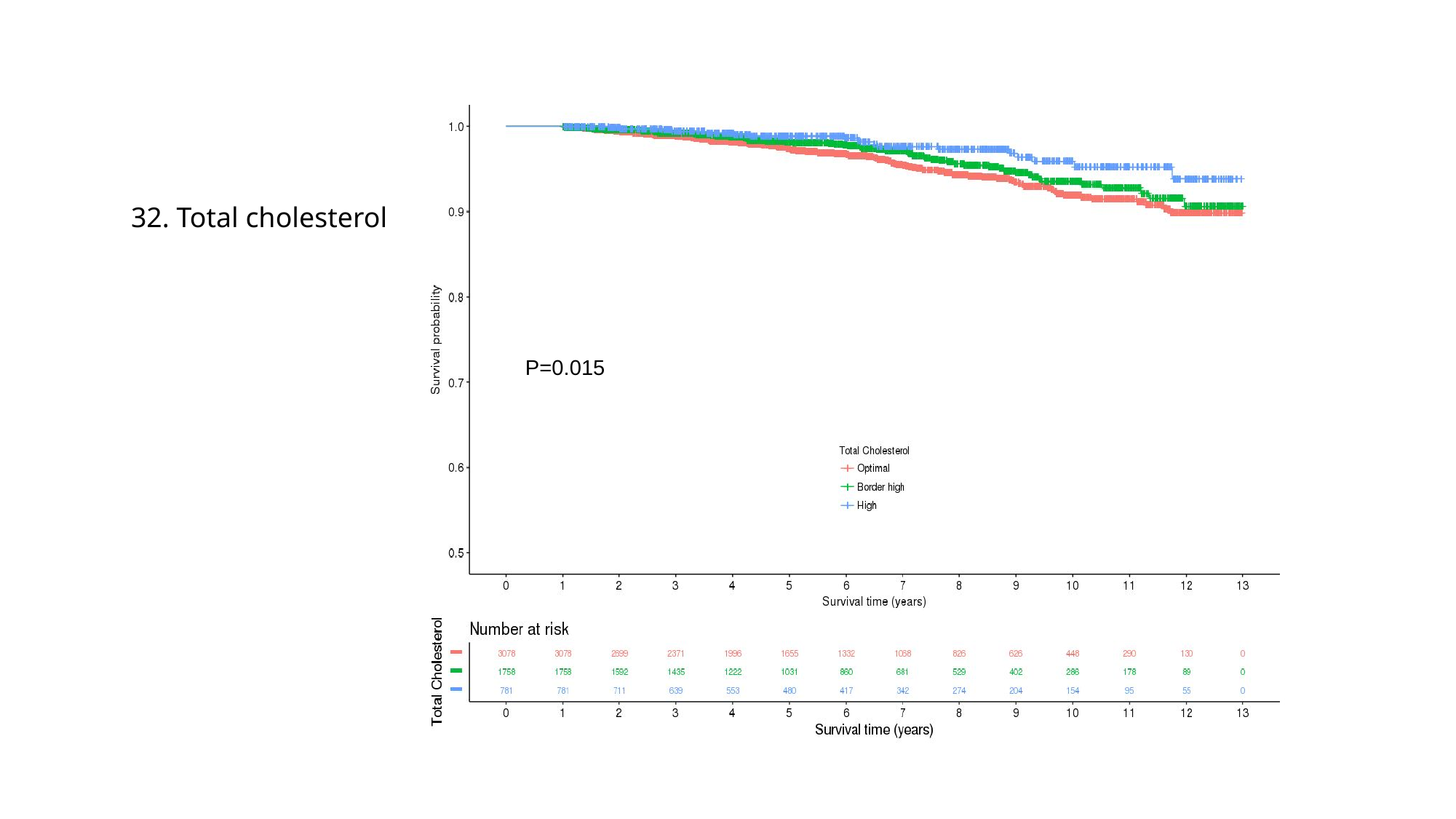

P=0.015
32. Total cholesterol

## Slide 65
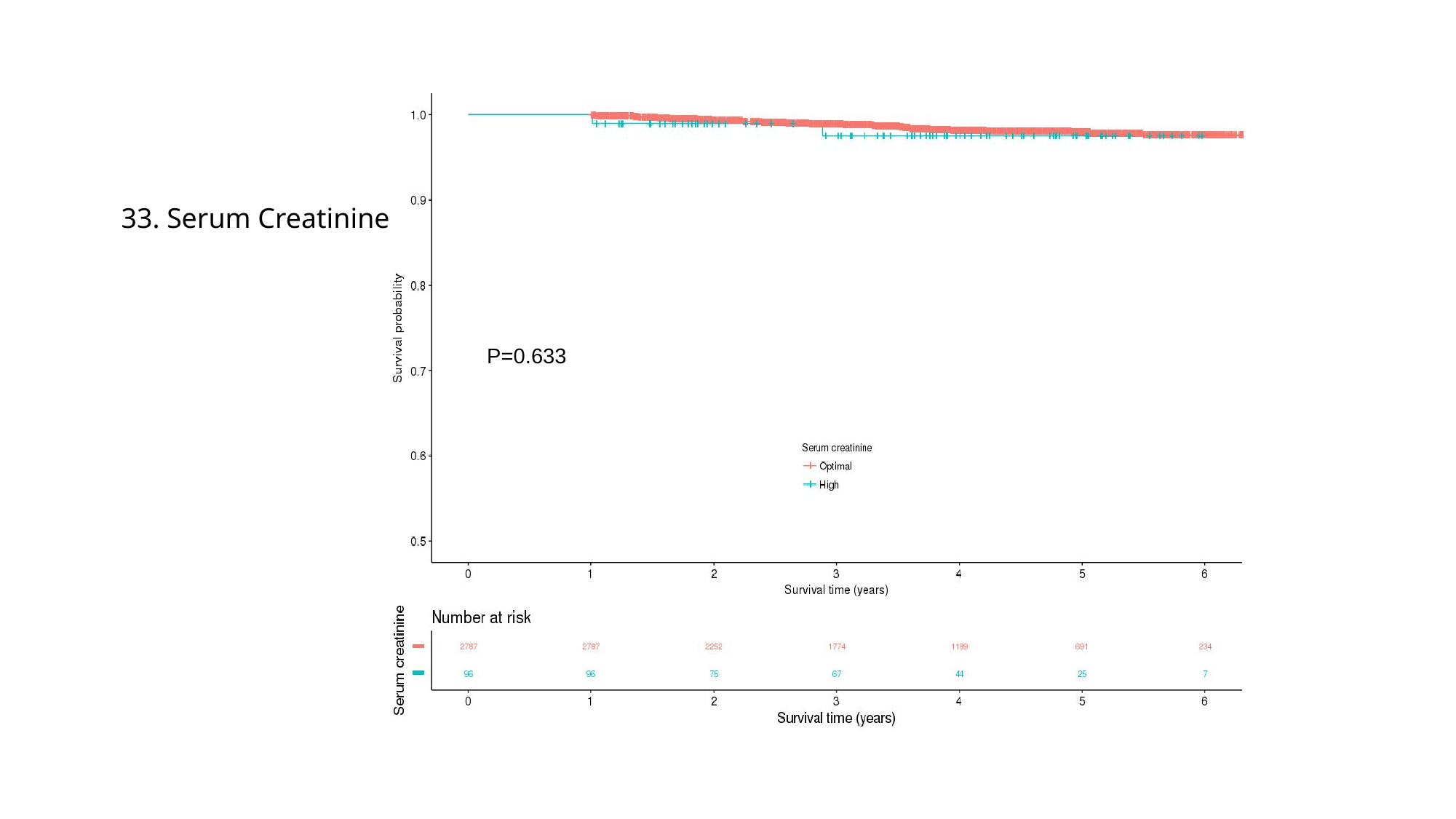

P=0.633
33. Serum Creatinine

## Slide 66
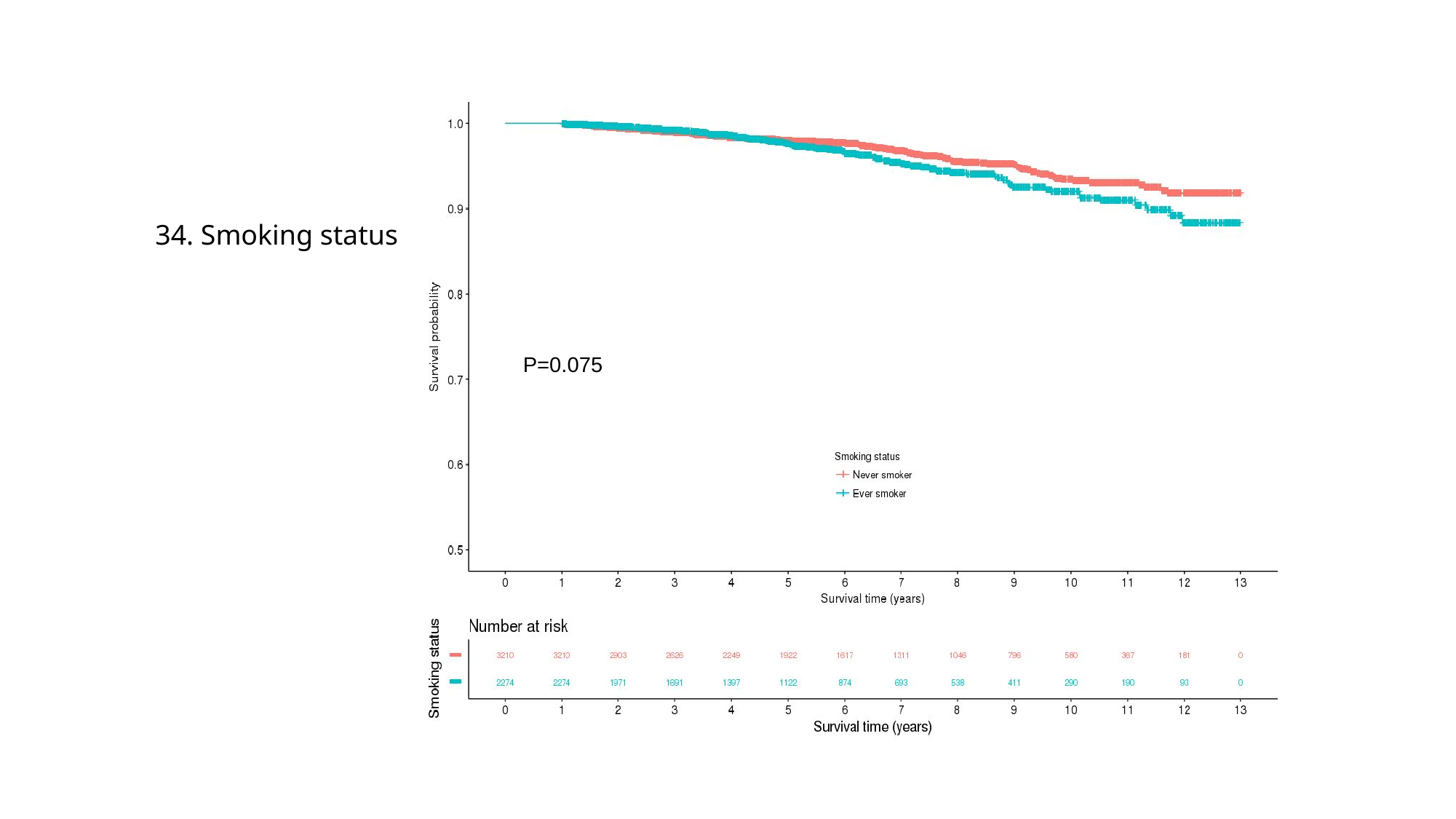

P=0.075
34. Smoking status
